# Providing regular and frequent maps of losses and gains of farmland birds based on European monitoring data

**DOI:** 10.1111/cobi.70268

**Published:** 2026-03-24

**Authors:** Sergi Herrando, Guillem Pocull, Sara Fraixedas, Anna Gamero, David Martí, Oriol Solà, Dani Villero, Verena Keller, Petr Voříšek, Alena Klvaňová, Gabriel Gargallo, Vitalie Ajder, Marc Anton, Ainars Aunins, Dawn Balmer, Mattia Brambilla, Tomasz Chodkiewicz, Przemysław Chylarecki, Cristi Domșa, Vlatka Dumbović Mazal, Virginia Escandell, Néstor Fernández, Ruud Foppen, Juan Gallego‐Zamorano, Carlos Godinho, Irene Guerrero, Christina Ieronymidou, Frédéric Jiguet, Aleksi Lehikoinen, Primož Kmecl, Peter Knaus, Lechosław Kuczyński, Åke Lindström, Qenan Maxhuni, Blas Molina, Jean‐Yves Paquet, Maria Luisa Paracchini, Danae Portolou, Draženko Z. Rajković, Dimitrije Radišić, Jiří Reif, Paul Shimmings, Henk Sierdsema, Jovica Sjeničić, Karel Šťastný, Bård G. Stokke, Nicolas Strebel, Zoltán D. Szabó, Tibor Szép, Norbert Teufelbauer, Nicolas Titeux, Sven Trautmann, Judit Veres‐Szászkac, Thomas Vikstrøm, Lluís Brotons

**Affiliations:** ^1^ European Bird Census Council (EBCC) Prague Czech Republic; ^2^ Catalan Ornithological Institute Natural Science Museum of Barcelona Barcelona Spain; ^3^ CREAF, Cerdanyola del Vallès Barcelona Spain; ^4^ Centre de Ciència i Tecnologia Forestal de Catalunya Solsona Spain; ^5^ Czech Society for Ornithology Prague Czech Republic; ^6^ Swiss Ornithological Institute Sempach Switzerland; ^7^ Institute of Ecology and Geography Moldova State University Chișinău Republic of Moldova; ^8^ Laboratory of Interdisciplinary Research on the Marine Environment and Marine–Terrestrial Atmosphere, ICI RECENT AIR Center, Prof. Dr. Ioan Borcea Marine Biological Station, Alexandru Ioan Cuza University of Iași Agigea Romania; ^9^ Department of Ecology, Faculty of Biology University of Latvia Riga Latvia; ^10^ Latvian Ornithological Society Riga Latvia; ^11^ British Trust for Ornithology Thetford UK; ^12^ Dipartimento di Scienze e Politiche Ambientali Università di Milano Milano Italy; ^13^ Museum and Institute of Zoology Polish Academy of Sciences Warsaw Poland; ^14^ Polish Society for the Protection of Birds (OTOP BirdLife Poland) Marki Poland; ^15^ Romanian Ornithological Society Cluj‐Napoca Romania; ^16^ State Agency for Environment and Nature Zagreb Croatia; ^17^ SEO/BirdLife Madrid Spain; ^18^ German Centre for Integrative Biodiversity Research (iDiv) Leipzig Germany; ^19^ Institute of Biology Martin‐Luther University Halle‐Wittenberg Halle Germany; ^20^ Sovon Dutch Centre for Field Ornithology Nijmegen The Netherlands; ^21^ Radboud Institute for Biology and Environmental Sciences Radboud University Nijmegen The Netherlands; ^22^ Laboratory of Ornithology, Institute of Mediterranean Agricultural and Environmental Sciences (ICAAM) University of Évora Évora Portugal; ^23^ Joint Research Centre European Commission Ispra Italy; ^24^ BirdLife Cyprus Nicosia Cyprus; ^25^ Muséum National d'Histoire Naturelle Paris France; ^26^ The Helsinki Lab of Ornithology, Finnish Museum of Natural History University of Helsinki Helsinki Finland; ^27^ DOPPS – BirdLife Slovenia Ljubljana Slovenia; ^28^ Population Ecology Lab Adam Mickiewicz University in Poznań Poznań Poland; ^29^ Department of Biology Lund University Lund Sweden; ^30^ Kosovo Ornithological Society Pristina Kosovo; ^31^ Aves Natagora, Département Études Namur Belgium; ^32^ Hellenic Ornithological Society/BirdLife Greece Athens Greece; ^33^ Department of Biology and Inland Waters Protection, Institute for Multidisciplinary Research University of Belgrade Belgrade Serbia; ^34^ Department of Biology and Ecology, Faculty of Sciences University of Novi Sad Novi Sad Serbia; ^35^ Institute for Environmental Studies, Faculty of Science Charles University Prague Czech Republic; ^36^ Department of Zoology, Faculty of Science Palacký University Olomouc Czech Republic; ^37^ BirdLife Norge Trondheim Norway; ^38^ Society for Research and Protection of Biodiversity Banja Luka Bosnia and Herzegovina; ^39^ Department of Ecology, Faculty of Environmental Sciences Czech University of Life Sciences Prague Czech Republic; ^40^ Norwegian Institute for Nature Research Trondheim Norway; ^41^ Milvus Group Bird and Nature Protection Association Târgu Mureș Romania; ^42^ Institute of Environmental Sciences University of Nyíregyháza Nyíregyháza Hungary; ^43^ Hungarian Ornithological and Nature Conservation Society (MME/BirdLife Hungary) Budapest Hungary; ^44^ BirdLife Austria Vienna Austria; ^45^ Environmental Research and Innovation Department Luxembourg Institute of Science and Technology, Observatory for Climate, Environment and Biodiversity Belvaux Luxembourg; ^46^ Dachverband Deutscher Avifaunisten e.V. Münster Germany; ^47^ Dansk Ornitologisk Forening/BirdLife Denmark Copenhagen Denmark; ^48^ Centro Superior Investigaciones Científicas (CSIC) Madrid Spain

**Keywords:** change maps, distribution modeling, EU policy, Europe, farmland birds, international monitoring, short‐term dynamics, Aves de tierras agrícolas, Europa, dinámica a corto plazo, mapas de cambio, modelado de distribución, monitoreo internacional, políticas EU, 变化地图, 分布建模, 国际监测, 短期动态, 农田鸟类, 欧盟政策, 欧洲

## Abstract

Knowledge of species distributions is essential for informing policies on nature conservation and restoration. However, updating them on a regular basis and doing so in a harmonized manner at the international level is difficult. The European Bird Census Council integrated national monitoring data covering 5 years to update farmland bird distributions and assessed how they changed. We used these data on 50 farmland bird species to generate 10×10 km maps showing their probability of occurrence from 2018 to 2022. We produced these maps with weighted ensemble species distribution models. We also developed models for the previous 5 years and plotted the differences in probabilities of occurrence per 10 × 10‐km area between the two periods as calibrated change maps. We evaluated model performance at continental and regional levels and interpreted changes in probability of occurrence in relation to known abundance trends. Models showed good predictive performance (mean AUC ≈ 0.84; mean squared error ≈ 0.13). Change estimates were reliable for 43 species (high accuracy, low bias), and distribution changes were positively correlated with independent abundance trends (Pearson's *r* ≈ 0.50). Thus, the distribution maps for the two periods accurately captured species’ distribution patterns and their temporal changes for all species at the European scale and for the majority of species in all regions except southeastern Europe. Among the 43 species with reliable estimates, predicted occurrences declined for 33 species, increased for nine, and were the same for one species. For most species, the direction of change in distribution was consistent with changes in species overall abundance in the same period, except for four species. Overall, our results indicated a recent contraction of farmland bird distributions in Europe, highlighting the strong capacity of existing bird monitoring networks to provide continent‐wide species maps that can be updated regularly.

## Abstract (es)

Aportación de mapas periódicos y frecuentes de las pérdidas y ganancias de aves de tierras agrícolas basados en datos de monitoreo europeos

**Resumen:**

El conocimiento de la distribución de las especies es esencial para informar las políticas de conservación y restauración de la naturaleza. Sin embargo, es difícil actualizarlas periódicamente y hacerlo de manera armonizada a nivel internacional. El Consejo Europeo de Censos de Aves integró los datos nacionales de monitoreo de cinco años para actualizar la distribución de las aves de las zonas agrícolas y evaluó cómo habían cambiado. Utilizamos estos datos para 50 especies de aves de las zonas agrícolas para generar mapas de 10 × 10 km que muestran su probabilidad de ocurrencia entre 2018 y 2022. Elaboramos estos mapas con modelos ponderados de distribución de especies. También desarrollamos modelos para los últimos cinco años y representamos gráficamente las diferencias en las probabilidades de ocurrencia por cada área de 10 × 10 km entre los dos periodos como mapas de cambios calibrados. Evaluamos el rendimiento del modelo a nivel continental y regional e interpretamos los cambios en la probabilidad de ocurrencia en relación con las tendencias de abundancia conocidas. Los modelos mostraron un buen rendimiento predictivo (AUC medio ≈ 0,84; error cuadrático medio ≈ 0,13). Las estimaciones de cambio fueron fiables para 43 especies (alta precisión, bajo sesgo), y los cambios en la distribución se correlacionaron positivamente con las tendencias independientes de abundancia (r de Pearson ≈ 0,50). Por lo tanto, los mapas de distribución de los dos períodos captaron con precisión los patrones de distribución de las especies, así como sus cambios temporales para todas las especies a escala europea y para la mayoría de las especies en todas las regiones, excepto en el sureste de Europa. Entre las 43 especies con estimaciones fiables, las ocurrencias previstas disminuyeron para 33 especies, aumentaron para 9 y se mantuvieron iguales para 1 especie. En la mayoría de las especies, la dirección del cambio en la distribución fue coherente con los cambios en la abundancia general de las especies durante el mismo período, excepto en el caso de cuatro especies. En general, nuestros resultados indicaron una reciente contracción de la distribución de las aves de las zonas agrícolas en Europa, lo que pone de relieve la gran capacidad de las redes de seguimiento de aves existentes para proporcionar mapas de especies a escala continental que pueden actualizarse periódicamente.

## 基于欧洲监测数据频繁定期发布农田鸟类分布增减地图

**【摘要】** 物种分布信息对制定自然保护与恢复政策至关重要。然而, 在国际尺度上定期更新并协调这些信息仍存在困难。欧洲鸟类普查委员会整合了5年的国家监测数据, 以更新农田鸟类分布图并评估其变化。我们利用50种农田鸟类的数据, 生成2018至2022年间10×10公里网格的物种出现概率图, 这些地图通过加权集合物种分布模型生成。我们还构建了前五年的模型, 将两个时间段内每10×10公里网格的物种出现概率差异绘制为校准变化图。我们在大陆和区域尺度上评估了模型表现, 并结合已知的种群丰度趋势来理解物种出现概率的变化。模型表现出良好的预测能力(平均AUC≈0.84;均方误差≈0.13)。分析结果中有43个物种的变化估计较为可靠(高精度、低偏差), 其分布变化与独立的种群丰度变化趋势呈正相关(皮尔逊相关系数≈0.50)。因此, 这两个时间段的分布地图准确捕捉了欧洲尺度上所有农田鸟类物种, 以及除东南欧外各区域尺度上多数物种的分布格局及其时序变化。在43个具有可靠估计的物种中, 33个物种的预测分布范围缩小, 9个物种的范围扩大, 还有1种保持不变。除4个物种外, 多数物种的分布变化趋势与同时期物种总丰度变化相一致。总体而言, 我们的研究结果表明, 欧洲农田鸟类分布范围近期呈现收缩趋势, 强调了现有鸟类监测网络有强大的能力来定期更新大陆尺度上的物种分布地图。**【翻译:胡怡思;审校:聂永刚】**

## INTRODUCTION

Knowledge on the distribution of species is essential to halt the loss of biodiversity. It provides key information for assessing the conservation status of species and can inform area‐based protection and restoration planning (Egoh et al., 2014; Fernández et al., 2020; Kissling et al., 2017; Rathore & Sharma, 2023). Consequently, species distribution is one of the essential biodiversity variables (EBVs) required to support international biodiversity agreements, such as the Kunming–Montreal Global Biodiversity Framework (GEO BON, 2022; Neugarten et al., 2024).

Information on bird distributions and on changes to distributions is included in assessments of international European policies, such as the EU Birds Directive (2009/147/EC) and other reporting processes (Council Directive 92/43/EEC) in the context of Natura2000, the Common Agricultural Policy (Regulation 2021/2115), the EU Biodiversity Strategy for 2030 (COM/2020/380 final), the Nature Restoration Regulation (EU, 2024/1991), and developments under the Bern Convention in non‐EU countries (Council of Europe, ETS 104). However, assembling data to robustly determine this EBV is often hindered by the availability of reliable long‐term, large‐scale information on biodiversity change (Dornelas et al., 2013; Rollinson et al., 2021). A recent analysis of the needs of biodiversity information identified constraints in spatial data as a major factor limiting the reliability of policy assessments (Moersberger et al., 2022).

Bird atlases are a primary source of information on bird distribution. Many European ornithological organizations produce atlases on a regular basis, and research has benefited enormously from these data (Herrando et al., 2019; Pototsky & Cresswell, 2023). However, national atlases are not generated simultaneously, their completion needs several years, and different methodologies and field sampling periods are used, all of which hampers their use in assessing species distribution changes at the international level (Gibbons et al., 2007; Keller, 2017). A significant number of European countries, particularly in the east and southeast, have not yet produced a national atlas (EBCC, 2022). The publication of the second European Breeding Bird Atlas (EBBA2) (Keller et al., 2020) constituted a major leap in knowledge about the distribution and abundance and change in distribution and abundance of breeding birds. Despite this leap, the 30 years that have elapsed since the first EBCC atlas (Hagemeijer & Blair, 1997) remain far longer than the frequency of data updates required for conservation and policy assessments, which aim to track distribution changes every 6–10 years (BirdLife International, 2021; Morán‐Ordóñez et al., 2023).

We developed the first steps in the new EBBA Live project, an initiative of the European Bird Census Council (EBCC) to evaluate the feasibility of updating species distribution maps across Europe every 5 years. Such updates require data on bird distributions to be collected in a consistent manner and to be reliable, balanced, and calibrated across space and time. In this context, we used data from the Pan‐European Common Bird Monitoring Scheme (PECBMS) to generate modeled maps of distribution at a spatial resolution of 10 × 10 km for 2013–2017 and 2018–2022 and maps of change in distribution between these two periods. The PECBMS is the best long‐term, large‐scale, and temporally fine‐grained dataset available (Gregory et al., 2019; PECBMS, 2023).

Because birds are key indicators of environmental change (Fraixedas et al., 2020; Järvinen & Väisänen, 1979), we did not select our study species at random; instead, we focused on farmland species because they can be used to examine specific environmental problems, because their overall conservation status in Europe is unfavorable (Burfield et al., 2023; Donald et al., 2001; Fuller et al., 1995; Reif et al., 2024; Rigal et al., 2023), and because of their role and policy relevance as indicators of the state of agricultural and grassland habitats (Anderle et al., 2024; Gregory et al., 2005). We produced maps to identify the areas where these species have declined, increased, or remained stable over the study period, and we analyzed the consistency between distribution changes and species abundance trends at the European level.

## METHODS

### Mapping approach

**FIGURE 1:**
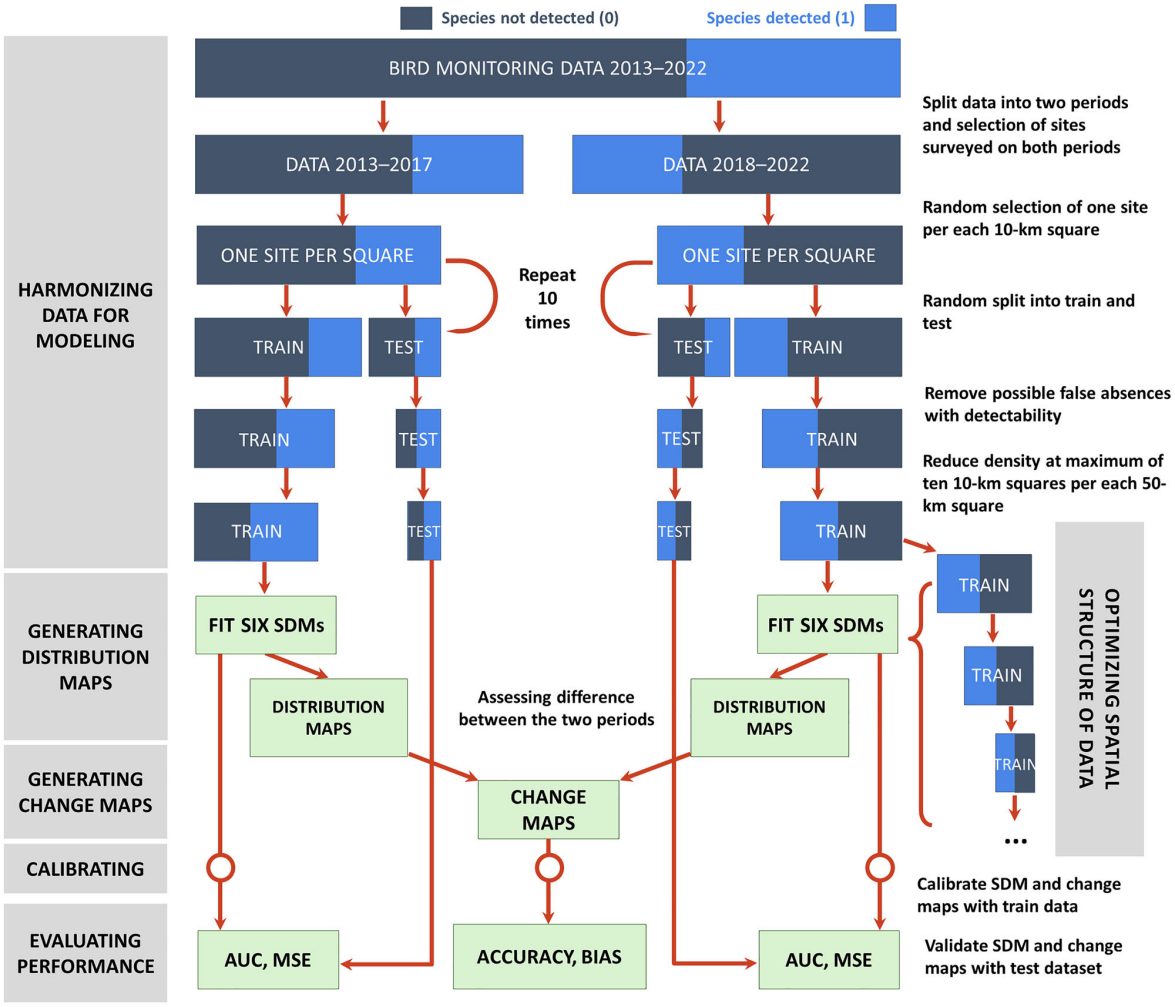
Workflow illustrating the steps (gray boxes) followed for data harmonization and modeling in an examination of farmland bird distributions and changes (blue boxes, data treatment; green boxes, statistical procedures; box size, proportional to the amount of data used; arrows, process order; SDM, species distribution model; AUC, area under the receiver operating characteristic curve; PR‐AUC, precision‐recall area under the curve; MSE, mean squared error). Additional information is in Appendices S1, S5, and S10.

We harmonized data in 10 × 10‐km squares and removed sites that could have represented false absences based on detectability. We modeled bird distribution with detections and reliable nondetections within the species breeding range to produce a map of nearly actual areas occupied during breeding for 2013–2017 and 2018–2022. We then estimated the per cell changes in occupancy probability based on the calibrated difference between the two maps (Figure 1). We included a series of model performance and validation analyses of the results (Figure 1). Finally, we interpreted changes in distribution with respect to changes in abundance and offered advice on the development of strategic sampling strategies. All the processes were executed on the MareNostrum5 Intel CPU cluster (BSC, 2024).

### Bird monitoring data

**FIGURE 2:**
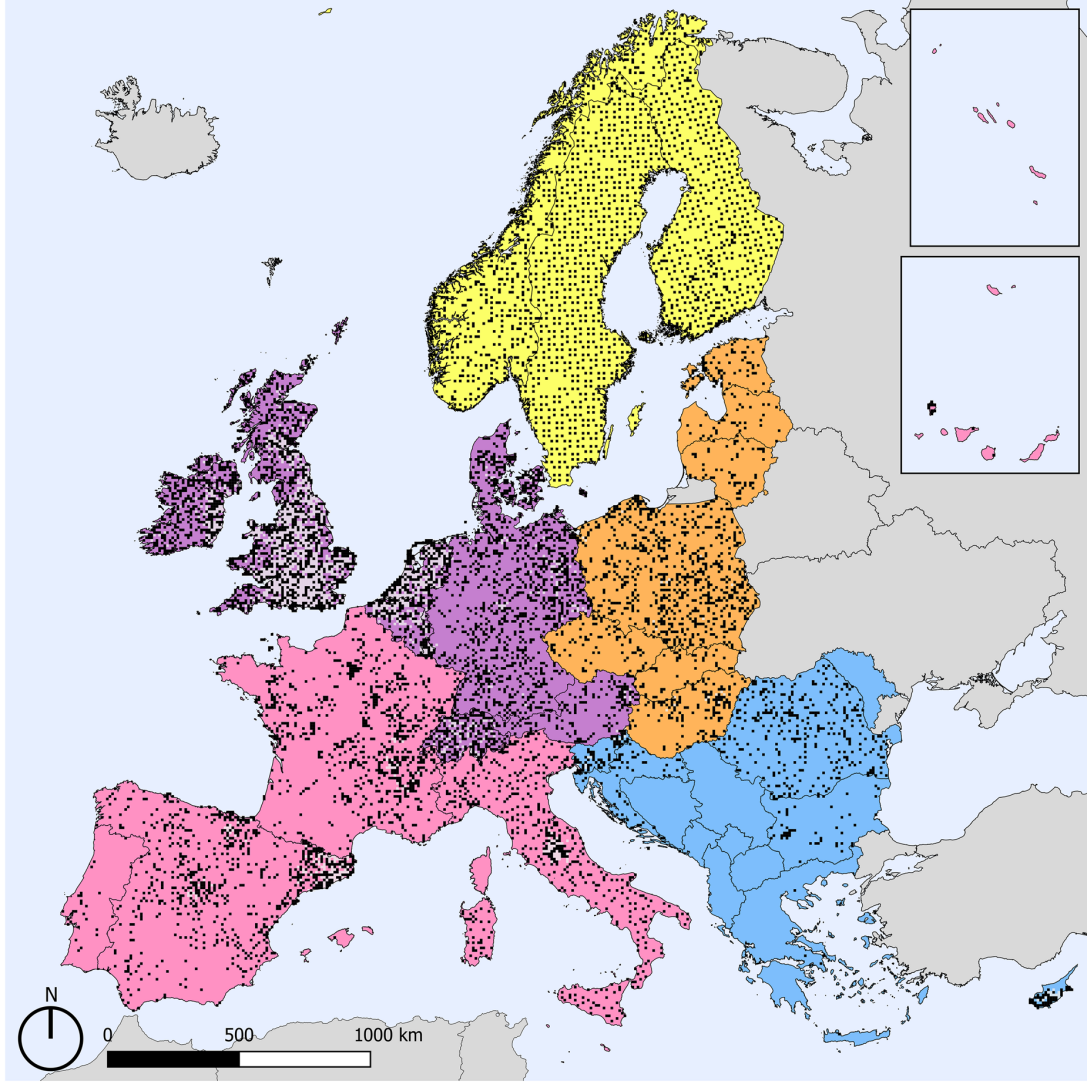
Study area (5,505,200 km^2^) in Europe showing the locations of the 10 × 10‐km grid squares containing all the Pan‐European Common Bird Monitoring Scheme sites surveyed at least once in each period (2013–2017 and 2018–2022) and regions where predicted map performance was evaluated (black, thinned 10 × 10‐km grid squares; semitransparent areas, squares not included in the study; pink, southwest; purple, western; yellow, northern; orange, central eastern; blue, southeast; top inset, Azores and Madeira; bottom inset, Canary Islands).

We extracted bird data from the PECBMS (2023) database. The PECBMS compiles data from 40 national and subnational monitoring schemes across 30 European countries and covers the western half of the continent (Figure 2). Each year, approximately 15,000 fieldworkers use standardized national protocols to count breeding birds (e.g., same sites, time of year, and survey duration). Typically, the same observers conduct counts over multiple years. These standardized observations are validated and analyzed at the national level to produce bird abundance trends. Eventually, data are shared and harmonized under the umbrella of the EBCC and contribute to annual and long‐term indicators of abundance changes at the continental scale (Brlík et al., 2021). The PECBMS site‐level database contains information on more than 450 bird species and 30,000 monitoring sites, primarily from generic, common bird monitoring schemes. Monitoring sites include the centroids of line transects, plots surveyed using territory mapping, or clusters of point counts. Validated data are updated every 1–4 years by national coordinators and further verified at the European level through automated detection of potential errors (e.g., occurrences outside known distributions). Verification is followed by a final revision by the national coordinators. The database contains for each species the estimated number of breeding individuals, pairs, and territories per site and year, site coordinates, and survey effort.

We selected 50 species that breed in agricultural and grassland habitats across Europe (see appendix 2 in Keller et al. [2020]), including all 39 farmland species used to develop the European Farmland Bird Index (Gregory et al., 2019). More information on the selected species is in “DISCUSSION.”

### Harmonizing data for modeling

We aimed to develop distribution maps for 2018–2022 and maps of changes to this metric between this 5‐year period and the previous 5 years (2013–2017) (Figure 1). Aggregating yearly monitoring data over study periods of 5 years allowed us to reduce the potential effect of environmental stochasticity among monitoring seasons (Lehikoinen & Virkkala, 2016). We allocated monitoring sites to corresponding 10 × 10‐km squares in the Lambert Azimuthal Equal‐Area (LAEA) projection of the ETRS89 reference frame.

To produce comparable maps for the two time periods, we harmonized the datasets through several data management steps. First, we selected monitoring sites surveyed in both periods (Figure 1) and randomly chose one unique site per 10 × 10‐km square. Next, we accounted for differences in effort by modeling detectability with site‐level data from multiple years (Briscoe et al., 2021) (details in “Modeling detectability”). We then aggregated site‐level data by period and 10 × 10‐km square, counting a detection if the species was observed in the square in at least 1 year and a nondetection if it was not observed in any year of the period. To mitigate false‐absence biases due to imperfect detection, we excluded squares in which the species was not detected and had a detection probability <0.25 (Strimas‐Mackey et al., 2023) (details in “Modeling detectability”). We randomly split this dataset and used 70% for training the species distribution models (SDMs) and 30% for testing performance (Figure 1). We limited the number of sampled squares per 50‐km area extracted from EBBA2 to a maximum of 10 (Appendix S1) and addressed class imbalance by selecting five random squares for each class. When either class had fewer than five squares, additional squares from the opposite class were added, up to a maximum of 10 per 50‐km area. This procedure produced a final selection of squares with a more homogeneous density than the original dataset (Figure 2; Appendices S2 & S3). All analyses were conducted in R 4.1.2 (R Core Team, 2021) and relied on the tidyverse package (Wickham et al., 2019).

### Site covariates

We selected site covariates to produce predicted maps that could be regularly updated and compared robustly. We used 35 predictors at the 10 × 10‐km grid resolution. We produced static covariates (i.e., those exhibiting minimal variation over short periods, including distance to the coastline, centroid longitude, mean elevation, and mean slope). We used dynamic covariates that could vary substantially over time and be regularly updated in the future. These were generated by computing the 5‐year mean values for each period. They included land‐cover percentages derived from the European Space Agency, the Climate Change Initiative data, the Shannon habitat diversity index (calculated from land cover), climatic variables, and normalized difference vegetation index (NDVI) values for the breeding season (April to July). We treated highway road density as a static variable due to the lack of updated data, but it could be included as a dynamic variable in the future if new data become available (Meijer et al., 2018). Detailed descriptions of the static and dynamic predictors and their sources are provided in Appendix S4.

We fitted the SDMs with all site covariates because such models perform better with many covariates (Briscoe et al., 2021; Merow et al., 2014). For static covariates, models for both periods were fitted with the same values, whereas dynamic covariates were assigned values for each period. All spatial analyses used to build the site covariates were conducted with R packages terra (Hijmans, 2023) and sf (Pebesma & Bivand, 2023).

### Modeling detectability

We modeled the detection probability with an occupancy model to identify and remove potential false absences and thus ensure more robust data for model fitting (Johnston et al., 2021; Strimas‐Mackey et al., 2023). We did not explicitly estimate the probability of occupancy with an occupancy model. In “DISCUSSION,” we provide the rationale for choosing correlative, neural networks and decision tree models over occupancy models to estimate and map the distribution.

To estimate detection probability, we used two observational covariates to account for species detectability in the field: the duration of field surveys and the specific method birdwatchers used to detect birds. The duration was calculated as the sum of the time spent in different surveys per site and year. The method covariate encompassed three categories: point counts (observations made from a fixed location), line transects (observations collected while walking on a fixed route), and territory mapping (area thoroughly surveyed to determine the territories of breeding birds) (Voříšek et al., 2008).

We modeled yearly detection probability as a function of the method covariate and the interaction between method and duration of the field survey. We conducted this analysis with the occu function from the R package unmarked (Kellner et al., 2023; Kéry & Royle, 2016). Finally, we calculated the cumulative estimated detectability per site in each 5‐year period as 1 − the product of the detection probabilities for the surveyed years in each period (Johnston et al., 2021; Kéry & Royle, 2016).

### Generating distribution maps

There is no consensus on the best models to use to produce species distribution maps (Qiao et al., 2015). Therefore, following the previous work done in EBBA2, we used seven types of statistical models to produce the SDMs: artificial neural network (ANN) from the R package nnet (Venables & Ripley, 2002), boosted regression trees (BRT) from the dismo package (Hijmans et al., 2023), flexible discriminant analysis (FDA) from the mda package (Leisch et al., 2023), generalized additive models (GAM) from the gam package (Hastie, 2023), generalized linear models (GLM) from the stats package (R Core Team, 2023), multivariate adaptive regression splines (MARS) from the earth package (Hastie & Tibshirani, 2023), and random forests (RF) from the ranger package (Wright & Ziegler, 2017).

Before building the SDMs, we fitted a model relating occurrence to yearly detection probability with data that included potential false absences. Next, we built GLM and GAM models with occurrence data that excluded potential false absences, adding the estimated coefficients from the previous model as offsets. We used these two SDMs to predict the probability of occurrence across the entire distribution range of the species, standardizing the yearly detection probability to 1. However, because models do not always align with observed frequencies (Vaughan & Ormerod, 2005), we fitted a new binomial GAM with training dataset observations as the response variable and the modeled probability of occurrence as the explanatory variable (Strimas‐Mackey et al., 2023). For this purpose, we employed the scam function from the R package scam (Pya, 2024) with the following parameters: gamma = 2, a smooth function with *k* = 6, and bs = mpi. Because calibration curves can produce probabilities outside the 0–1 range, we constrained these artifact values to the proper range. The same calibration method has been applied in modeled distribution maps in eBird (Strimas‐Mackey et al., 2023).

We built the five remaining models (ANN, BRT, FDA, MARS, and RF) and predicted the probability of occurrence without incorporating detectability (Figure 1). To adjust for detectability bias, we used the calibration procedure described in the previous paragraph to calibrate the probability of occurrence with the GAM‐derived occurrence probability map. Thus, we adjusted them jointly for detectability and occurrence gradient bias. Species with lower detection probabilities exhibited a greater increase in occurrence estimates, whereas species with high detection probabilities (close to one) showed minimal increases.

Finally, we generated the final distribution map with the weighted ensemble prediction procedure (Keller et al., 2020). This procedure consists of averaging the occurrence probabilities of the seven SDMs with weights based on their performance on the training data. Artifact SDMs (without predicted values) were discarded from the ensemble. Although the PECBMS scheme does not currently cover some countries in southeastern Europe (notably the western Balkans and Moldova) and poorly covers Greece, we took into account the entire region in our predictions (Figure 2).

We developed and calibrated SDMs with observational data and predictor values exclusively within the geographical breeding range of each species, rather than across the entire study area. We considered that fitting models within the range provided two main advantages. First, it reduced the number of absences without valuable information, potentially enhancing the fit of covariates to actual occupancy and mitigating class imbalance (Johnston et al., 2021; Strimas‐Mackey et al., 2023). Second, prediction maps were less likely to be extrapolated into ecologically inaccessible areas or into regions constrained by historical, dispersal, or biological factors that the models ignore, thereby producing maps with greater ecological meaning. However, there is no clear consensus on the best approach (Bergman et al., 2024). We then set the predicted probability of occurrence to zero in squares in the range where water bodies covered more than 75% of the area or permanent ice extended over 50% of the area because these were poorly sampled regions that introduced artifacts in the prediction maps of some species. Moreover, farmland species are highly unlikely to breed in such habitats.

We assumed species ranges were identical for both periods. We defined them with EBBA2 50‐km presence data from the 2013–2017 period and included the 50‐km squares with presence data from the second period (2018–2022) to extend the range with a 50‐km gap‐filling buffer. We applied a smoothing process to the species range with the ksmooth method (smoothness = 6), implemented with the R package smoothr (Strimas‐Mackey, 2023). The species range was then downscaled to the 10 × 10‐km grid, and we used this range consistently for both periods.

We cross‐validated the modeled maps 10 times with refined test datasets across the entire study area and at the regional level (Figure 1). The regional analyses were applied to western Europe, northern Europe, southeastern Europe, southwestern Europe, and central eastern Europe (Figure 1). To validate the spatial predictions, we employed three widely used metrics: area under the receiver operating characteristic curve (AUC), precision‐recall area under the curve (PR‐AUC), and mean squared error (MSE) (Briscoe et al., 2021; Johnston et al., 2021; Saito & Rehmsmeier, 2015; Strimas‐Mackey et al., 2023). We calculated the average intraspecies standard deviation (AISD) for AUC, PR‐AUC, and MSE across the entire study area to evaluate how much of the performance error was attributable to variability among the multiple models for each species.

### Generating change maps

We generated a predicted change map at 10 × 10‐km resolution by calculating the difference in the probability of occurrence between the second (2018–2022) and first (2013–2017) periods (Figure 1). Predicted change values ranged from –1 to 1. Negative values indicated a reduction in distribution, whereas positive values indicated an increase in this metric.

Predicted change maps might have inherent potential biases. Biases might emerge from variations in sampling effort or from other factors not captured by the site covariates (e.g., biological competition, predation, or disturbances). To address these biases, we applied the same calibration method as the one we used for the distribution maps (see “Generating distribution maps”). In this case, the calibration aligned with the observed gains, losses, and stable squares with the predicted changes via a GAM function. We performed model selection to evaluate whether models including calibrated change were better supported by the data than models without calibration. We then produced modeled change maps as the calibrated differences between the two periods (details on the explanatory power of calibrated vs. noncalibrated maps in Appendices S10–S13).

The utility of change predictions depends on how much they can be trusted (Piirainen et al., 2023). Following the species‐level method developed by Rapacciuolo et al. (2014), we tested the performance of the predicted changes by weighting the predicted change values by the probability of occurrence in the first period. We then used the accuracy and bias metrics (Rapacciuolo et al., 2014) to evaluate the predicted change through the same cross‐validation methodology. The accuracy metric quantifies the deviation between the ideal observation–prediction line and the actual weighted change predicted at each observation site. The bias metric measures the difference in the area below and above the ideal observation–prediction curve. A negative bias indicated that the predicted change maps tended to underestimate gains or overestimate losses, whereas a positive bias indicated the opposite. We considered accuracy values >0.7 good indicators of change performance and bias values from –0.25 to 0.25 as indicating no bias.

To standardize the measure of change in distribution, we calculated the percentage of change in distribution as the sum of the change in occurrence probability for all the 10 × 10‐km squares in each species’ range divided by the total number of squares of the species range.

### Changes in distribution versus changes in abundance

According to the EBV framework (GEO BON, 2022), one of the six EBV classes is species populations, which includes species distributions and species abundances. Changes in distribution should not be directly interpreted as changes in abundance (Koleček & Reif, 2011). We aimed to explore the nature and strength of any potential correlation between these two types of changes, as has been attempted in previous national‐level studies (Briscoe et al., 2021).

To do that, we studied the relationship between the predicted change in occurrence as a proxy for change in distribution for the periods 2013–2017 and 2018–2022 and the annual change in species population indices as a proxy for abundance over the same 10‐year period (2013–2022) at the European level (PECBMS, 2023). This analysis included 35 species for which abundance trends were available and the model produced robust change maps (see “Generating change maps”). For the abundance data, annual change was expressed as the additive slopes and their 95% confidence interval (CI) for the 10‐year period (PECBMS, 2023).

For the change in distribution, we used the percentage of change in distribution (explained at in “Generating change maps”). We then conducted a nonparametric bootstrap with 1000 replicates with replacement to obtain the 95% CI for predicted change in occurrence probability. Finally, we classified the species according to their predicted change in probability of occurrence. Species with CIs below zero were classified as significant losses, those with CIs above 0 as significant gains, and those species with CIs overlapping zero as stable (i.e., no significant change). We then compared the species that had the same trend direction for distribution and abundance.

## RESULTS

We produced distribution maps for 50 farmland bird species for 2018–2022 (Appendices S5 & S9). All models performed well (AUC > 0.70 and MSE < 0.25 at the European level) (Appendices S6–S8). The models had a mean AUC of 0.840 (AISD = 0.004), a mean PR‐AUC of 0.539 (AISD = 0.006), and a mean MSE of 0.133 (AISD = 0.005). At the regional level, the model of the southwest had the best mean performance (AUC = 0.841, PR‐AUC = 0.550, MSE = 0.117). In contrast, the model of the southeast performed the worst (AUC = 0.714, PR‐AUC = 0.475, MSE = 0.164) (Appendices S6–S8).

**FIGURE 3:**
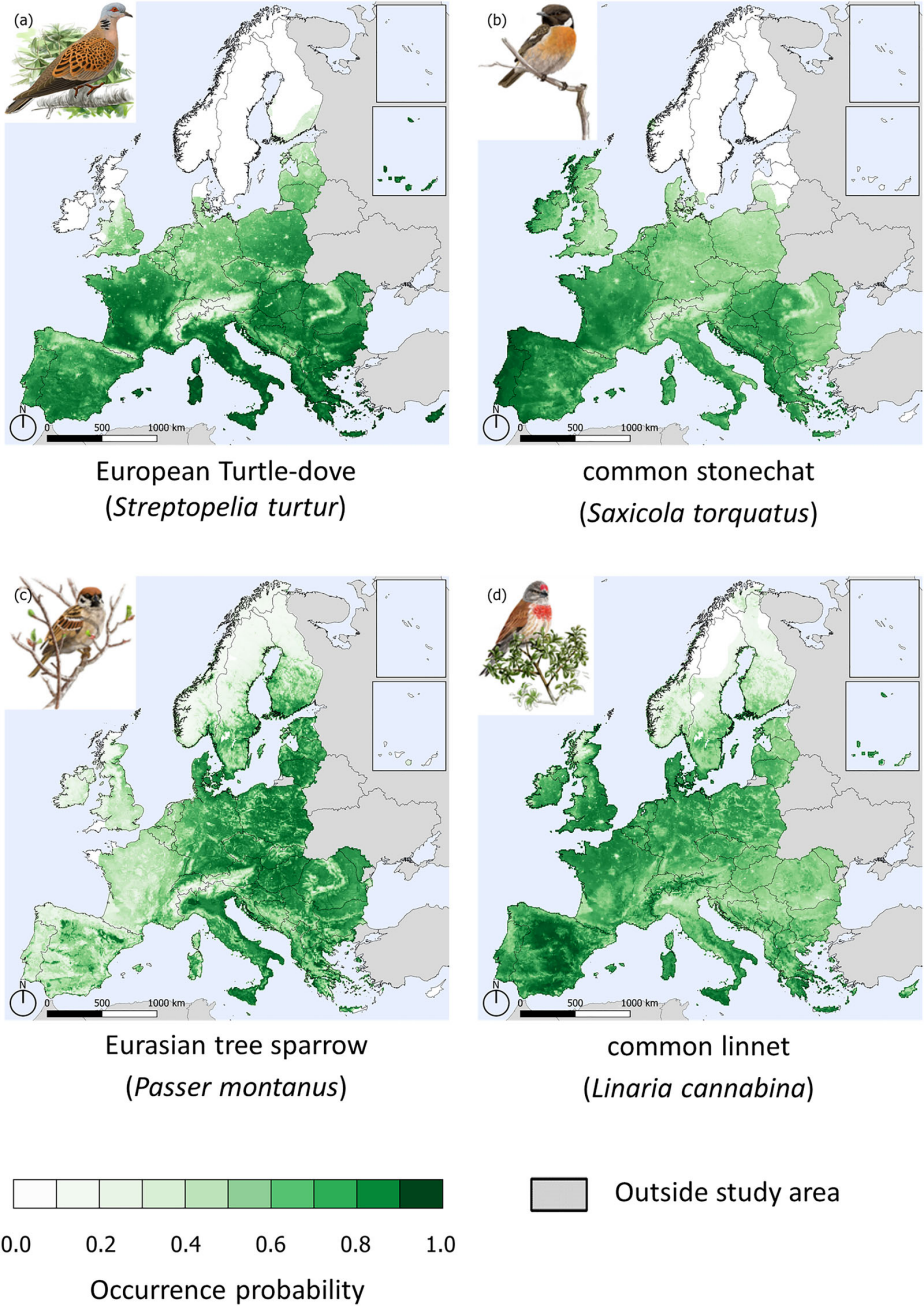
Occurrence distribution in Europe from 2018 to 2022 of (a) European turtle‐dove (*Streptopelia turtur*) (area under the receiver operating characteristic curve [AUC] = 0.847, precision‐recall area under the curve [PR‐AUC] = 0.640, mean squared error [MSE] = 0.192); (b) common stonechat (*Saxicola torquatus*) (AUC = 0.767, PR‐AUC = 0.543, MSE = 0.203); (c) Eurasian tree sparrow (*Passer montanus*) (AUC = 0.841, PR‐AUC = 0.606, MSE = 0.158); and (d) common linnet (*Linaria cannabina*) (AUC = 0.776, PR‐AUC = 0.623, MSE = 0.200). Map resolution is 10 × 10 km. Illustrations of birds by Toni Llobet.

**FIGURE 4:**
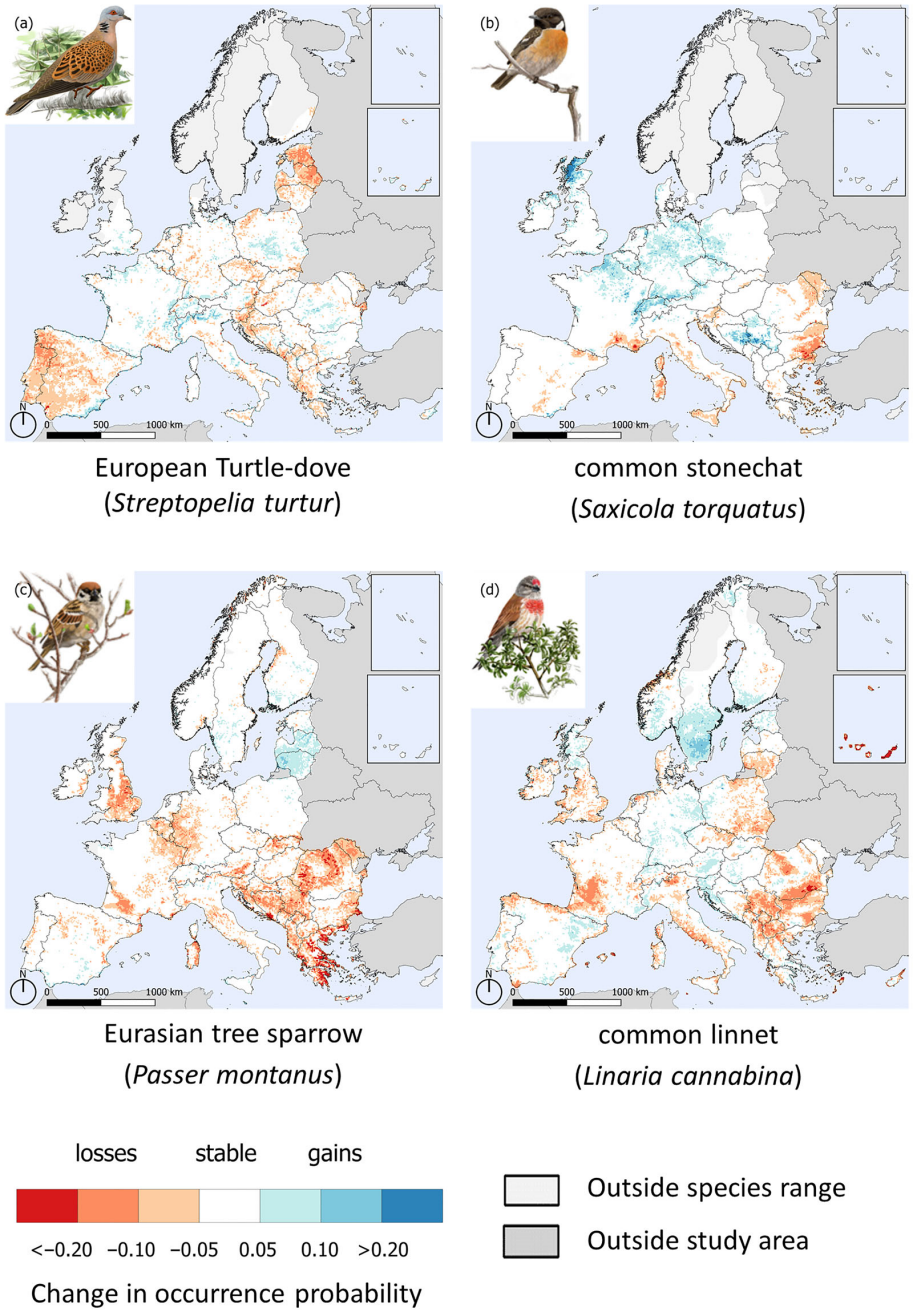
Predicted change in occurrence for (a) European turtle‐dove (*Streptopelia turtur*) (prediction accuracy, 0.867; bias, −0.099; overall change in distribution, −1.525% to −1.437%), (b) common Stonechat (*Saxicola torquatus*) (accuracy, 0.884; bias, 0.099; overall change in distribution, 0.021% to 0.109%), (c) Eurasian tree sparrow (*Passer montanus*) (accuracy, 0.882; bias, 0.124; overall change in distribution, −1.919% to −1.828%), and (d) common linnet (*Linaria cannabina*) (accuracy, 0.906; bias, −0.004; overall change in distribution across the entire range of the species, −0.906% to −0.814%). Gains or losses determined based on a minimum change threshold of 0.05. Map resolution is 10 × 10 km. Illustrations of birds by Toni Llobet.

We selected four species as examples, European turtle‐dove (*Streptopelia turtur*; Linnaeus, 1758), common stonechat (*Saxicola torquatus*; Linnaeus, 1758), Eurasian tree sparrow (*Passer montanus*; Linnaeus, 1758), and common linnet (*Linaria cannabina*; Linnaeus, 1758), because of their widespread and complementary patterns of distribution across Europe (Figure 3), and their contrasting occupancy changes, increasing in some regions while declining in others (Figure 4). Models performed well across the entire study area and in the five analyzed regions, but the model of the southeast consistently showed the worst performance (Appendix S9).

Distribution maps for the 50 farmland species for 2013–2017 performed well (AUC > 0.7 and MSE < 0.25). The mean AUC was 0.838 (AISD = 0.004), the mean PR‐AUC was 0.551 (AISD = 0.006), and the mean MSE was 0.135 (AISD = 0.004). The models used to produce the change maps performed well for 43 species (models converged). Accuracy was >0.7 and bias was –0.25 to 0.25 (full set of change maps and statistics in Appendix S11). For these 43 species, the mean accuracy was 0.832 (SD 0.064) and the mean bias was 0.076 (0.122). The model of the southwestern region had the best mean change performance (accuracy = 0.826 [0.067], bias = 0.049 [0.110]), followed by the central eastern region (accuracy = 0.820 [0.069], bias = 0.041 [0.075]). The southeastern region showed the lowest mean performance (accuracy = 0.756 [0.083], bias = 0.034 [0.075]).

Among the 43 species that had accurate and unbiased change maps, 42 had a significant change in distribution across the study area. Thirty‐three species (77%) showed significant losses, whereas nine (21%) showed significant gains (Table 1). The 43 species together lost on average 0.79% of their distribution across the Europe between 2013–2017 and 2018–2022. The two species with the largest losses were the black‐eared wheatear (*Oenanthe hispanica*; Linnaeus, 1758), which lost an average of 3.7%, and the woodchat shrike (*Lanius senator*; Linnaeus, 1758), which lost 2.3%. The two species with the largest gains were the lesser kestrel (*Falco naumanni*; Fleischer, 1818), which increased by an average of 1.4%, and the Spanish sparrow (*Passer hispaniolensis*; Temminck, 1820), which increased by 1.1% (Table 1).

**TABLE 1:** Mean percent change (95% CI) in distribution of 43 farmland birds in Europe versus European regions for models that performed well (Appendix S10).

Scientific name, change direction^a^	Europe (95% CI)	Central eastern (95% CI)	Northern (95% CI)	Southeastern (95% CI)	Southwestern (95% CI)	Western (95% CI)
*Alauda arvensis*, decrease	0.37 (−0.40 to 0.35)	−0.66 (−0.71 to −0.61)	1.43 (1.39 to 1.47)	−2.26 (−2.33 to −2.18)	−1.00 (−1.05 to −0.96)	0.02 (−0.03 to 0.06)
*Alectoris rufa*, decrease	−1.32 (−1.39 to −1.26)	–	–	−0.46 (−0.81 to −0.11)	−1.59 (−1.67 to −1.53)	−0.28 (−0.41 to −0.16)
*Anthus campestris*, increase	0.14 (0.10 to 0.18)	−0.76 (−0.82 to −0.70)	−0.33 (−0.49 to −0.18)	1.21 (1.11 to 1.32)	−0.12 (−0.18 to −0.06)	0.22 (0.15 to 0.29)
*Anthus pratensis*, decrease	−1.01 (−1.05 to −0.96)	−2.27 (−2.39 to −2.16)	−1.22 (−1.34 to −1.11)	−0.32 (−0.52 to −0.11)	−0.76 (−0.82 to −0.69)	−0.03 (−0.09 to 0.03)
*Athene noctua*, decrease	−1.21 (−1.26 to −1.16)	1.10 (1.06 to 1.14)	–	−0.18 (−0.29 to −0.06)	−3.15 (−3.25 to −3.06)	−0.27 (−0.36 to −0.18)
*Bubulcus ibis*, increase	0.79 (0.69 to 0.88)	−0.2 (−0.28 to −0.12)	–	−0.58 (−0.76 to −0.41)	1.00 (0.89 to 1.10)	0.80 (0.69 to 0.92)
*Burhinus oedicnemus*, increase	0.44 (0.36 to 0.51)	−2.16 (−2.34 to −1.98)	–	−0.90 (−0.99 to −0.80)	0.70 (0.61 to 0.79)	3.90 (3.58 to 4.22)
*Calandrella brachydactyla*, decrease	−0.54 (−0.62 to −0.45)	−0.04 (−0.26 to 0.16)	–	−0.01 (−0.18 to 0.16)	−0.84 (−0.94 to −0.76)	–
*Ciconia ciconia*, decrease	−1.27 (−1.32 to −1.22)	−3.17 (−3.27 to −3.06)	1.31 (1.00 to 1.64)	−0.98 (−1.16 to −0.79)	−1.09 (−1.16 to −1.02)	0.07 (0.03 to 0.11)
*Coracias garrulus*, decrease	−1.08 (−1.17 to −1.00)	0.5 (0.39 to 0.62)	–	−2.28 (−2.44 to −2.11)	−0.54 (−0.64 to −0.44)	1.47 (1.33 to 1.59)
*Corvus frugilegus*, decrease	−0.16 (−0.20 to −0.11)	−1.99 (−2.06 to −1.91)	1.22 (1.12 to 1.33)	0.44 (0.33 to 0.54)	1.69 (1.51 to 1.88)	−0.86 (−0.93 to −0.8)
*Coturnix coturnix*, decrease	−0.82 (−0.86 to −0.78)	−2.49 (−2.57 to −2.4)	−2.25 (−2.30 to −2.21)	−1.95 (−2.07 to −1.83)	0.56 (0.45 to 0.66)	0.57 (0.5 to 0.64)
*Crex crex*, decrease	−2.22 (−2.28 to −2.16)	−3.19 −3.31 to −3.06)	−1.68 (−1.77 to −1.58)	−5.75 (−5.97 to −5.55)	−0.44 (−0.47 to −0.41)	0.30 (0.26 to 0.34)
*Curruca communis*, decrease	−0.95 (−1.01 to −0.90)	−0.52 (−0.62 to −0.43)	−0.71 (−0.79 to −0.63)	0.41 (0.26 to 0.56)	−2.82 (−2.93 to −2.71)	−0.12 (−0.21 to −0.03)
*Emberiza calandra*, decrease	−0.37 (−0.41 to −0.34)	−0.68 (−0.74 to −0.62)	1.09 (0.93 to 1.25)	0.82 (0.75 to 0.89)	−1.43 (−1.5 to −1.36)	0.48 (0.43 to 0.52)
*Emberiza cirlus*, increase	0.66 (0.61 to 0.72)	6.92 (6.22 to 7.69)	–	2.49 (2.36 to 2.63)	−0.30 (−0.35 to −0.24)	1.57 (1.51 to 1.62)
*Emberiza citrinella*, decrease	−1.27 (−1.31 to −1.24)	0.30 (0.26 to 0.34)	−0.61 (−0.69 to −0.54)	−2.48 (−2.56 to −2.39)	−1.74 (−1.82 to −1.66)	−1.83 (−1.91 to −1.76)
*Emberiza hortulana*, decrease	−0.28 (−0.33 to −0.23)	−1.80 (−1.89 to −1.72)	−1.09 (−1.15 to −1.04)	2.05 (1.9 to 2.2)	−0.91 (−0.98 to −0.83)	0.12 (0.05 to 0.20)
*Falco naumanni*, increase	1.37 (1.22 to 1.52)	–	–	3.46 (3.14 to 3.80)	1.25 (1.09 to 1.40)	–
*Falco tinnunculus*, increase	0.84 (0.80 to 0.88)	3.30 (3.19 to 3.40)	1.73 (1.64 to 1.82)	2.15 (2.04 to 2.26)	−0.47 (−0.53 to −0.41)	−1.19 (−1.28 to −1.11)
*Galerida cristata*, decrease	−0.82 (−0.88 to −0.76)	−0.39 (−0.44 to −0.34)	–	−0.23 (−0.39 to −0.09)	−1.44 (−1.52 to −1.37)	−0.55 (−0.60 to −0.50)
*Galerida theklae*, ns	−0.13 (−0.34 to 0.07)	–	–	–	−0.13 (−0.34 to 0.08)	–
*Hirundo rustica*, decrease	−0.88 (−0.92 to −0.84)	−3.90 (−3.99 to −3.81)	0.17 (0.07 to 0.28)	−2.16 (−2.26 to −2.06)	0.70 (0.65 to 0.75)	−1.19 (−1.28 to −1.10)
*Lanius collurio*, decrease	−1.26 (−1.31 to −1.23)	−0.10 (−0.16 to −0.03)	−2.93 (−3.04 to −2.83)	−1.40 (−1.49 to −1.30)	−1.40 (−1.49 to −1.32)	0.04 (−0.06 to 0.16)
*Lanius meridionalis*, decrease	−2.32 (−2.49 to −2.14)	–	–	–	−2.32 (−2.5 to −2.13)	–
*Lanius minor*, decrease	−2.24 (−2.48 to −2.00)	−0.93 (−1.15 to −0.70)	–	−3.15 (−3.48 to −2.82)	−0.07 (−0.42 to 0.26)	0.21 (−0.15 to 0.56)
*Lanius senator*, decrease	−2.33 (−2.43 to −2.23)	–	–	−2.79 (−2.99 to −2.59)	−2.13 (−2.23 to −2.01)	−2.15 (−2.54 to −1.75)
*Linaria cannabina*, decrease	−0.86 (−0.91 to −0.81)	−1.57 (−1.67 to −1.47)	2.61 (2.52 to 2.69)	−3.65 (−3.77 to −3.51)	−1.62 (−1.70 to −1.54)	0.09 (0.01 to 0.18)
*Melanocorypha calandra*, decrease	−0.97 (−1.11 to −0.84)	–	–	−2.15 (−2.43 to −1.87)	−0.17 (−0.31 to −0.04)	–
*Motacilla flava*, decrease	−0.17 (−0.2 to −0.13)	−2.53 (−2.64 to −2.42)	−0.09 (−0.16 to −0.02)	1.71 (1.61 to 1.80)	−0.11 (−0.16 to −0.05)	−0.17 (−0.23 to −0.12)
*Oenanthe hispanica*, decrease	−3.74 (−3.89 to −3.60)	–	–	−6.36 (−6.68 to −6.07)	−2.24 (−2.38 to −2.11)	–
*Passer hispaniolensis*, increase	1.08 (0.95 to 1.22)	0.43 (0.14 to 0.71)		0.10 (−0.10 to 0.29)	2.13 (1.97 to 2.30)	–
*Passer montanus*, decrease	−1.87 (−1.92 to −1.83)	0.67 (0.58 to 0.77)	0.47 (0.42 to 0.52)	−7.35 (−7.49 to −7.22)	−1.51 (−1.57 to −1.45)	−2.47 (−2.56 to −2.38)
*Perdix perdix*, decrease	−2.27 (−2.33 to −2.20)	−4.00 (−4.12 to −3.88)	−0.64 (−0.82 to −0.47)	−5.09 (−5.26 to −4.92)	−0.06 (−0.14 to 0.01)	−1.22 (−1.30 to −1.13)
*Petronia petronia*, decrease	−0.58 (−0.64 to −0.53)	–	–	1.25 (1.07 to 1.47)	−0.82 (−0.88 to −0.76)	–
*Saxicola rubetra*, decrease	−1.30 (−1.35 to −1.24)	−4.17 (−4.30 to −4.05)	0.21 (0.12 to 0.31)	−4.96 (−5.14 to −4.78)	−0.80 (−0.87 to −0.72)	1.28 (1.18 to 1.39)
*Saxicola torquatus*, increase	0.07 (0.02 to 0.11)	0.81 (0.74 to 0.88)	0.58 (0.36 to 0.77)	−2.33 (−2.44 to −2.21)	−1.22 (−1.28 to −1.16)	3.64 (3.56 to 3.72)
*Serinus serinus*, decrease	−0.22 (−0.27 to −0.17)	−0.64 (−0.72 to −0.56)	2.40 (2.23 to 2.56)	−1.87 (−2.02 to −1.75)	0.51 (0.43 to 0.60)	−0.08 (−0.17 to 0.02)
*Streptopelia turtur*, decrease	−1.48 (−1.52 to −1.44)	−2.45 (−2.56 to −2.34)	−1.03 (−1.25 to −0.80)	−1.62 (−1.71 to −1.53)	−1.67 (−1.75 to −1.59)	0.00 (−0.08 to 0.09)
*Sturnus vulgaris*, decrease	−0.49 (−0.54 to −0.45)	1.01 (0.94 to 1.08)	−0.07 (−0.15 to 0.01)	−2.62 (−2.74 to −2.49)	1.29 (1.21 to 1.37)	−2.25 (−2.34 to −2.15)
*Tetrax tetrax*, decrease	−1.22 (−1.36 to −1.08)	–	–	–	−1.21 (−1.35 to −1.06)	–
*Upupa epops*, increase	0.20 (0.13 to 0.28)	1.01 (0.88 to 1.13)	5.02 (4.37 to 5.73)	2.61 (2.42 to 2.77)	−1.49 (−1.63 to −1.35)	−0.43 (−0.58 to −0.28)
*Vanellus vanellus*, decrease	−1.33 (−1.36 to −1.30)	−3.10 (−3.19 to −3.03)	−0.06 (−0.11 to −0.02)	−3.17 (−3.29 to −3.06)	0.05 (0.00 to 0.10)	−1.95 (−2.02 to −1.88)
Mean (SD)^b^	−0.79 (1.04)	−0.81 (2.21)	−0.22 (1.69)	−1.26 (2.53)	−0.64 (1.19)	−0.07 (1.40)

^a^ Decrease or increase for statistically significant changes in Europe (ns, not significant). Scientific names follow the taxonomy of the Handbook of the Birds of the World and BirdLife International (2024) and are shown in alphabetical order.

^b^ Mean change in distribution.

In the four example cases, the pattern of change was predominantly negative, although both the magnitude and direction of change varied spatially and some areas experienced gains. The European turtle‐dove exemplified the overall decrease in distribution during the study period; losses were significant in most European regions. The common stonechat showed a minor overall gain, but we observed contrasting positive and negative changes. The negative changes were concentrated in the southern regions, where the species had the highest occurrences (Figure 3). Amid an overall trend of loss, the Eurasian tree sparrow showed minor gains only in the central eastern and northern regions. Finally, for the common linnet, losses predominantly occurred in the southwestern, central eastern, and southeastern regions, whereas gains were concentrated in the north and west. However, unlike the rather homogeneous pattern of the previous species, the regions exhibited noticeable variation.

**FIGURE 5:**
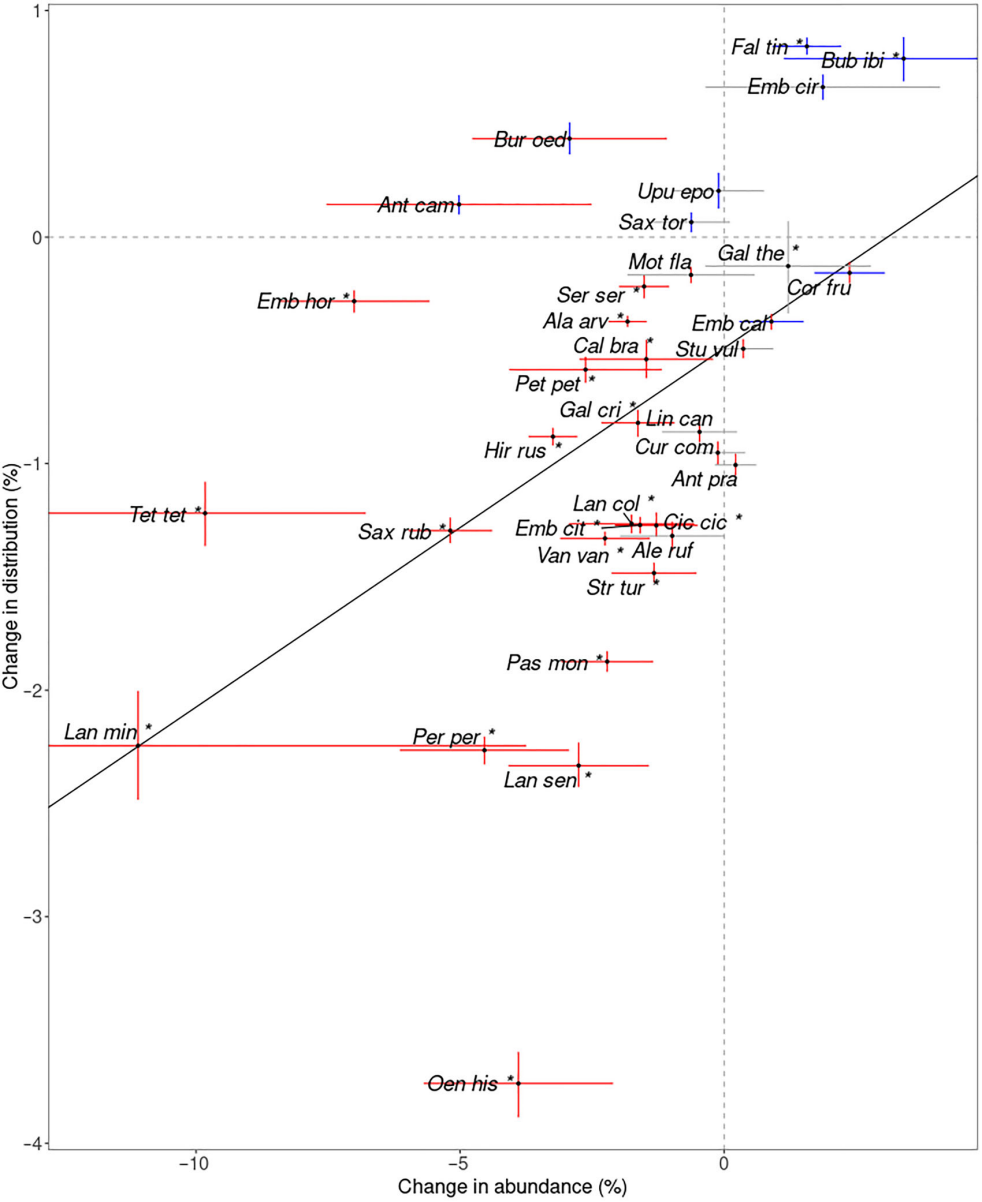
Comparison of the average estimated percent change in distribution in farmland bird species ranges from 2013 to 2017 and from 2018 to 2022 estimated for this study (Appendix S12) and the average annual percent change in abundance across Europe for 2013–2022 estimated from abundance trends (PECBMS, 2023) (red, losses; blue, gains; gray, no significant change; vertical lines, 95% CI from the bootstrapping procedure on calibrated predicted change in distribution; horizontal lines, same statistic for percent annual abundance change; black line, linear model [intercept = −0.490 to slope = 0.158]). Only distribution models that performed well in both spatial and change evaluations and for which Pan‐European Common Bird Monitoring Scheme abundance trends were available are shown (*n* = 35).

A comparison between the distribution change (2013–2017 vs. 2018–2022) and the annual abundance changes (2013–2022) showed the same direction of change for most species (Figure 5). The two parameters were correlated (Pearson's *r* = 0.499), and a linear model yielded an intercept of –0.489 and a slope of 0.158. Among the 35 species analyzed, 19 exhibited a significant decline in distribution and abundance (i.e., CI did not include zero), two showed a significant increase in both parameters, 10 displayed changes either in distribution or abundance that were uncertain, and in four the two types of change had a different direction (Figure 5).

## DISCUSSION

### Tracking changes in distribution of farmland birds

In contrast to nonspatial species abundance trends such as the common bird index, fine‐grained distribution changes have been used rarely in policy‐relevant international assessments due to their scarcity and low reliability (EEA, 2024; IPBES, 2018). Our study contributes to filling this knowledge gap by successfully predicting the distribution of 50 bird species across Europe for the periods 2013–2017 and 2018–2022 and, more importantly, by determining changes in distribution between these two periods for most of the study species. Together with recent work developed in North America (Johnston et al., 2025), these are currently the most comprehensive high‐resolution maps of change available at a continental scale, and they have great potential to contribute to understanding distribution dynamics and guide conservation and restoration efforts at large spatial scales.

Our results showed recent losses of farmland birds across Europe that seemed to indicate a continuation of the overall loss in distribution found from the 1980s to the 2010s (Keller et al., 2020). Our change maps revealed information on the general decline of farmland birds in Europe in only 5 years at a 10 × 10‐km resolution (Appendix S12). A total of 77% of the species for which change was reliably estimated exhibited a statistically significant overall loss in distribution (Table 1). Some species experienced more than 2% loss in distribution, which represents a noticeable loss given the short period analyzed. In our opinion, tracking changes every few years could be used as an alert system to help improve policy reporting and trigger early action through its spatially explicit dimension, a feature that is entirely new in Europe.

Identifying key drivers of change in distribution and quantifying their effect is crucial in ecology and conservation (Briscoe et al., 2021; Kelleher et al., 2025; Kéry et al., 2013; Zurell et al., 2023). However, understanding and predicting changes in the distribution of breeding birds at European level is complex and mediated by multiple driving forces (Howard et al., 2023; Marjakangas et al., 2023). The patterns of change we found were heterogeneous across Europe; 65% of species exhibited contrasting positive and negative trends across different regions (Table 1). In general, although in the northern and western regions losses were moderate, they were more dominant in the southwest, southeast, and central east. This suggests that there could be different underlying drivers of change in different parts of species ranges or different responses of local abundances to the same drivers. We argue that the continental‐scale and fine spatiotemporal patterns we found offer great potential to identify plausible drivers of change as done at national scale (Cabodevilla et al., 2022; Malte et al., 2020; Sharps et al., 2023).

Analyzing the causes of change in farmland birds was beyond the scope of this work. Nevertheless, it is well established that agricultural intensification and farmland abandonment are the key drivers of abundance changes in these species (e.g., Gregory et al., 2005; Reif & Hanzelka, 2020; Rigal et al., 2023; Wretenberg et al., 2006). At the European scale, the species we considered are largely dependent on farmland and serve as indicators of farmland ecosystems (Keller et al., 2020). However, some of these species also occur in other habitat types. For example, a large fraction of the northern Europe population of the meadow pipit (*Anthus pratensis*; Linnaeus, 1758) and the western yellow wagtail (*Motacilla flava*; Linnaeus, 1758) breed in mountain tundra, mire, and bog (Stjernman et al., 2013), and many tawny pipits (*Anthus campestris*; Linnaeus, 1758) and black‐eared wheatears breed in Mediterranean low shrublands (Herrando et al., 2014). There are similar examples across Europe (Keller et al., 2020). Consequently, changes in certain areas cannot be directly attributed to agricultural activity in some species. Although we predicted distributions and changes inside and outside farmland areas, we calculated the percentage of predicted distribution (2018–2022) occurring in farmland to facilitate interpretation of the farmland bird maps (Appendices S15 & S16).

Modeling counts to generate maps of absolute numbers of birds has proven useful at national levels (e.g., Gedeon et al., 2014; Knaus et al., 2018; Nabias et al., 2024; Sovon Vogelonderzoek Nederland, 2018). However, harmonizing abundance data at the European scale is challenging because different field and data processing protocols are used. At finer resolution, occupancy probability gets closer to local abundance, but their relation tends to be less clear at coarser resolution (Brambilla et al., 2024). Analyzing and interpreting changes in abundance and distribution has been done at national levels (e.g., Brambilla et al., 2024; Heldbjerg et al., 2025), but to our knowledge, ours is the first study that explores these patterns at continental level. For the great majority of species, we found that the direction of change in distribution across Europe was the same as the change in abundance for the same period (Figure 5). This suggests that, in general, a loss in distribution across a species range could be used as a spatial metric associated with a parallel abundance decline. Four species (11%) showed overall losses in distribution, despite a general increase in abundance, or the opposite pattern (Figure 5). However, this is not necessarily an inconsistent result, as reported for some species at the national level (e.g., Franch et al., 2021; Lardelli et al., 2022; Mihelič et al., 2019; Šťastný et al., 2021). Careful interpretation is thus recommended for understanding species‐specific patterns of change.

We were unable to evaluate change in distribution for several species (Appendix S14). General breeding bird surveys do not always provide enough data for some species, and either greater fieldwork effort or species‐specific monitoring schemes may be needed for modeling the distribution for particular species. Some of the limitations could be partially overcome by relaxing the needs of temporal precision and with wider time windows for each study period (e.g., 10 years instead of 5).

Despite the value of its high level of standardization, the monitoring data are always limited in terms of quantity, and this could certainly limit the extension of our approach to some bird species. This could be at least partially overcome by integrating non‐monitoring data into this modeling process, as has been done for North America (Johston et al., 2025). Advancements in citizen science and technology have been used to implement international online recording platforms, such as eBird, Observation, and Ornitho. Although the use of these data has limitations, such as spatiotemporal or species reporting bias (Robinson et al., 2022; Zhang, 2020), scientists have used them widely in recent studies to determine species abundance, distribution, and change (Callaghan et al., 2021; Fink et al., 2023; Johnston et al., 2021, 2025; Pflüger et al., 2024; Sullivan et al., 2014). Combining data from standardized and unstandardized projects has proven successful in mapping species distribution and distribution change at national levels (e.g., Knaus et al., 2018; Lardelli et al., 2022; Sovon Vogelonderzoek Nederland, 2018; Strebel et al., 2022). We therefore consider that incorporating data from the EuroBirdPortal (EBP, 2024), the project that collects data from all online bird recording portals in Europe, represents a promising source for improving the modeling of species distribution and its change on this continent.

Beyond bird populations, information gaps still persist across most taxonomic groups in Europe, highlighting the need to reinforce sustainable monitoring structures (Bresadola & Bjärhall, 2025; Santana et al., 2025). However, our approach could be applied to other biological groups, such as butterflies, which have a very well‐structured monitoring scheme in Europe (eBMS, 2025). The importance of improving the taxonomic and geographic representation of monitoring data has also been reported globally (Ledger et al., 2023), but no fundamental obstacles prevent use of available data to map changes in occurrence in a manner similar to that employed in this study.

### Methodological considerations

The methodology implemented in EBBA2 (Keller et al., 2020) provided the core framework underpinning our approach. However, we introduced several improvements to reduce data and modeling biases, enhance model performance, and ensure a high comparability between the two periods. The main upgrades were refining modeling detectability with site covariates, removing possible false absences, fitting SDMs, predicting distribution within species range, and calibrating occurrence and change maps. Regarding the removal of suspected false absences, it may likely result in models that better reflect true environmental conditions or occupancy associations, thus making predictions more reliable and interpretable.

In terms of detectability, we implemented a hierarchical approach in which both observational and environmental covariates were included in a site occupancy model. This approach allowed us to separate the observation process (detectability) from the biological process (true occupancy), leading to more accurate and ecologically meaningful inferences. We did not address detectability bias with hierarchical models (i.e., static and dynamic occupancy frameworks), which are specifically designed to account for such bias (Kéry & Royle, 2021). This decision was shaped by the use of PECBMS site‐level data, which lacked disaggregated information on repeated surveys for each breeding season, a requirement for occupancy models due to the closure assumption, where a species’ state must remain constant during the study period (Kelleher et al., 2025; Kéry & Royle, 2016). In addition, predicting distribution only within a species’ known range reduces commission errors (i.e., predicting presence where the species does not occur) and improves model calibration. Finally, related to calibration, we made use of a novel method developed by eBird (Strimas‐Mackey et al., 2023) that aligns model predictions with actual observations by accounting for variation in observer effort and detectability. This helped ensure that predicted distributions were consistent with the actual conditions under which observations were made.

Change maps were constrained within the plausible ecological thresholds of each species. At the general level, this involved calibrating the change in distribution values within the species‐specific limits of possible change. At the local level, the calibration was further refined by constraining the range of change to the predicted distribution values from the initial time period. By doing so, we found that, compared with preliminary analyses (not shown), models of distribution change for a greater number of species performed well. Predicted changes in distribution also aligned more closely with observed changes (Figure 5) and better reflected annual abundance trends.

We suggest that additional improvements include incorporation of variables containing information on soil properties, farmland intensity, small road density, and the effects of disturbances, such as wind, flooding, or fire, that might explain the distribution of species and its change at a finer scale. Future studies should aim to increase the set of covariates available for modeling detectability by, for instance, incorporating data on survey dates, meteorological conditions, and seasonal land‐cover dynamics (Kelleher et al., 2025).

The contribution of site covariates in SDMs may have varied between the two periods due to differences in the statistical relationship between species occurrence and these covariates, which could affect predictions of distributional change. This could be particularly important for highly influential site covariates, such as longitude, for which patterns of change in some species could be generated by relatively small differences in their contribution in the two periods. Therefore, gaining a deeper understanding of the extent to which environmental covariates influence model outputs would improve local predictions, offer insights into the drivers of distributional change, and support proper interpretation of the estimated changes.

Further studies may consider novel methodologies that could improve modeling distribution and change. These include applying spatially varying coefficient models, which address biases arising from wide‐ranging species whose habitats differ significantly along latitudinal gradients (Doser et al., 2024; Martin et al., 2019); applying adaptive spatiotemporal exploratory models (Fink et al., 2013, 2020); and explicitly modeling spatial autocorrelation (Anderson et al., 2022). Finally, model performance and validation procedures could be improved in the future, for example, by using spatial accuracy measures (Carl & Kuhn, 2017).

### Policy implications

Information on the spatial dimension of change is gaining interest in the context of biodiversity loss. In Europe, this has been addressed mostly at the national level, but harmonized information among countries is increasingly required (Morán‐Ordóñez et al., 2023). A good example is the recently approved EU Nature Restoration Regulation (EU, 2024), which asks member states to identify farmland areas in need of restoration and monitor the potential recovery of farmland bird populations. We believe that initiatives such as the EBBA Live project could contribute to robustly determining where farmland birds are declining in Europe, therefore complementing existing tools such as the farmland bird index (Gregory et al., 2005).

Our results showed that by applying advanced methodologies, the European partnership on bird monitoring (EBCC, 2024) can provide crucial information on short‐term spatiotemporal dynamics of populations. Rigorous and reliable maps in distribution and change maps can be generated at short intervals and at relatively fine spatial resolution, thanks to standardized data collection procedures applied by thousands of skilled ornithologists (PECBMS, 2023). However, it is important to improve coverage in areas with monitoring gaps in the Balkan countries and Eastern Europe to successfully cover the entire continent, as in EBBA2.

We addressed recent calls for developing EBVs for enhancing nature policy assessments in Europe (Liquete et al., 2024). Our findings could be used to establish a new foundation for tracking the state of biodiversity, based on reinforcing the role of long‐term, large‐scale, and site‐level monitoring data. We hope our results will improve the targeting of conservation and restoration measures and inform the development of restoration plans.

## Supporting information



Additional supporting information may be found in the online version of the article at the publisher's website.

## Data Availability

The data used in this study are stored and curated by the European Bird Census Council (EBCC) through its Pan‐European Common Bird Monitoring Scheme (PECBMS). The data are owned by EBCC partner organizations that coordinate monitoring schemes in their respective countries. Access to these data is available on request to the EBCC, which coordinates permission from the multiple data owners and thus facilitates continental‐wide data mobilization. Detailed instructions and contact information for requesting access are available at https://pecbms.info/use‐of‐the‐results/data‐access‐policy/.
