# Supplementary material for: Providing regular and frequent maps of losses and gains of farmland birds based on European monitoring data
Source: Conserv Biol. 2026 Mar 24;40(4):e70268. doi: 10.1111/cobi.70268 (PMC13392801; doi:10.1111/cobi.70268)
Supplement: Supplementary file 1 — Additional supporting information may be found in the online version of the article at the publisher's website. [file COBI-40-e70268-s001.docx]

**Supporting Information for the article “Providing regular and frequent maps of losses and gains of farmland birds based on European monitoring data”**

Appendix S1. Optimising the spatial structure of the data…..…………………….. 2

Appendix S2. Impact of reducing number of squares in model performance….….. 3

Appendix S3. Visual representation of reducing number of squares….…………… 5

Appendix S4. Covariates used in the correlative models…..……………………… 6

Appendix S5. Distribution maps…………………………………………………… 8

Appendix S6. AUC performance of distribution models ………………………….. 9

Appendix S7. PR-AUC performance of distribution models….…………………... 11

Appendix S8. MSE performance of distribution models....………………………... 13

Appendix S9. Distribution maps for the period 2018–2022…..…………………… 15

Appendix S10. Change in distribution maps……………………….……………….28

Appendix S11. Accuracy and bias metrics of change maps…..……..…………….. 29

Appendix S12. Change maps….…………………………………………………… 31

Appendix S13. Observed change vs predicted change…………………………….. 42

Appendix S14. Performance on sample size……………………………………….. 43

Appendix S15. Percentage of species distribution in farmland habitats…...………. 44

Appendix S16. Map of farmland areas…..………………………………………… 45

Appendix S17. List of participating countries and organizations…...……………... 46

References………………………………………………………………………….. 48

**Appendix S1. Optimising the spatial structure of the data.** Despite efforts to harmonize data, bird monitoring surveys remained unevenly distributed across Europe (see main Fig. 2). Highly sampled regions (e.g., the Netherlands, Belgium, and Great Britain) may increase models’ spatial autocorrelation, reducing the reliability to infer from the final models (Johnston et al. 2021; Moudrý et al. 2024; Strimas-Mackey et al. 2023). Conversely, excessively reducing sampling density, and consequently the sample size, can negatively affect model performance. To address this trade-off and optimize data density, we evaluated spatial performance by progressively reducing sampling density, from a maximum of 24 sampled 10-km squares per 50-km grid square to a single 10-km square. We evaluated performance using the cross-validation test datasets for the period 2018–2022, repeating the process 10 times.

We found that the Area Under the Receiver Operating Characteristic Curve (AUC) metric remained robust with a density of 10 10-km squares per 50-km grid, whereas this value could be reduced to four for the Mean Squared Error (MSE) validation statistic (Appendix S2). To be conservative, we selected the number derived from the AUC analysis. A visual example of the reduced sampling density of 10-km squares is shown in Appendix S3.

**Appendix S2**. **Impact of reducing number of squares in model performance.** Impact of reducing the number of 10-km squares within each 50-km square on model performance, evaluated using AUC (top), Precision-Recall AUC (middle), and MSE (bottom).


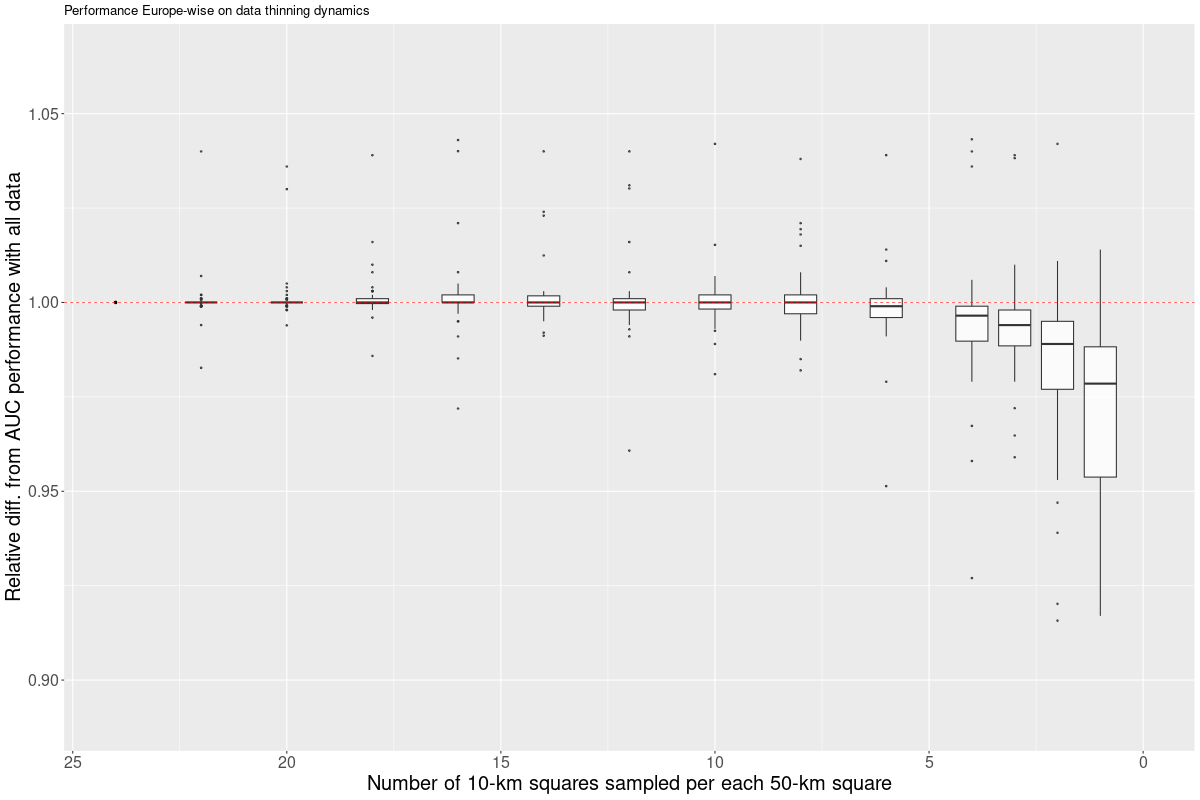


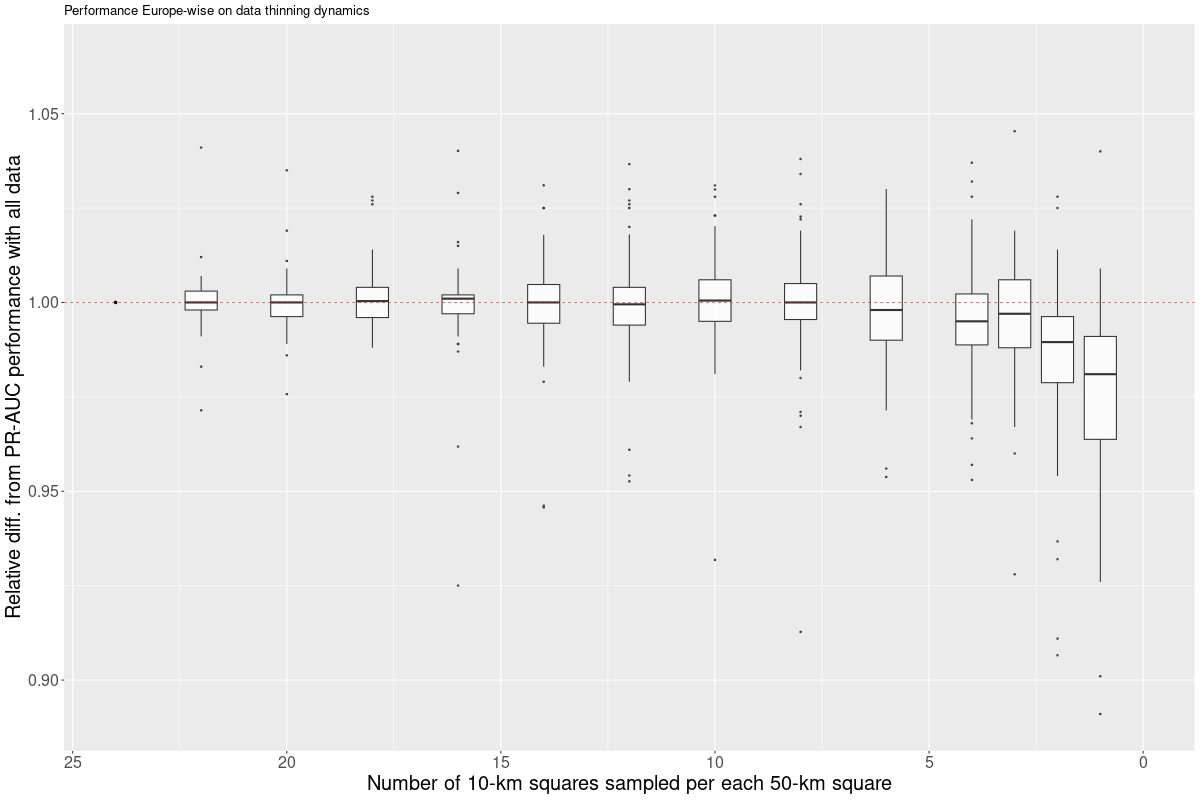


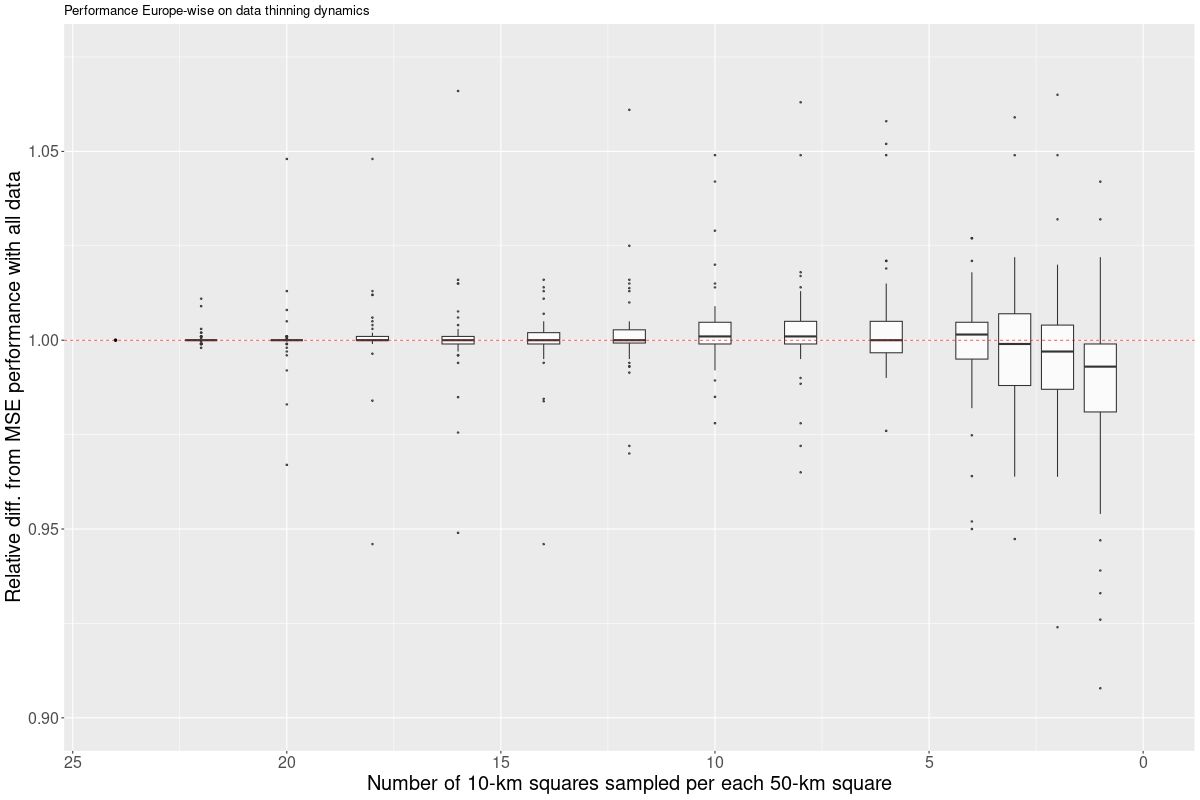


**Appendix S3**. **Visual representation of reducing number of squares.** Visualization of the local effects of reducing the number of sampled 10-km squares in Western and Central Europe, an area characterized by heterogeneous densities of monitoring sites. The map shows 10-km squares with shared sampled sites from both periods (2013–2017 and 2018–2022; off-white squares), 10-km squares retained after the thinning process to homogenize spatial density (maximum of 10 10-km squares per 50-km grid cell; black squares), and the 50-km grid squares (blue squares).

**
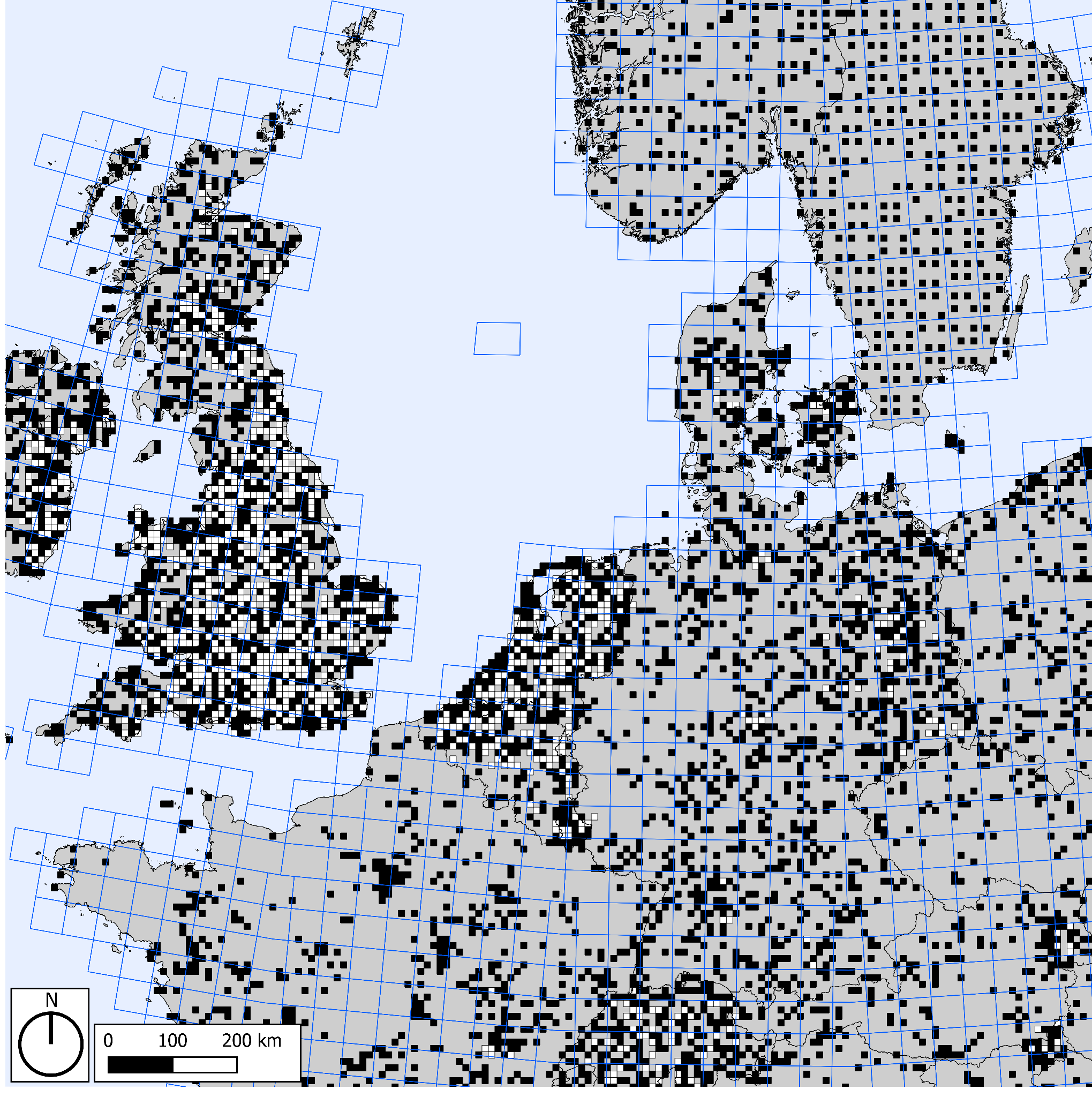
**

**Appendix S4. Covariates used in the correlative models.** List of the 35 static and dynamic covariates used in correlative models. No asterisk refers to static predictors. An asterisk means that the site covariate is dynamic (could change over relatively short periods of time). We did not include latitude because this covariate was highly correlated with mean temperature, following the procedure of EBBA2. Note that some covariates were calculated for the breeding period (considered from April to July). Covariates are sorted from higher to lower importance for all species in both periods (2013–2017 and 2018–2022). EEA: European Environmental Agency; ERA5-Land: ECMWF ReAnalysis v.5 Land, Copernicus; MODIS: Moderate Resolution Imaging Spectroradiometer from NASA; FAO: Food and Agriculture Organization of the United Nations; ESA CCI: European Spatial Agency Climate Change Initiative; EBBA2: European Breeding Bird Atlas 2; EBBALF: European Breeding Bird Atlas Live Farmland.

| **Category** | **Name of site covariate** | **Units** | **Sources** |
| --- | --- | --- | --- |
| Geography | Longitude (center of 10-km square) | m | EEA |
| Climate | Mean temperature of the warmest month * | K | ERA5-Land |
| Climate | Annual evapotranspiration * | ET/PET | MODIS |
| Climate | Surface net solar radiation * | J /m^2^ | ERA5-Land |
| Climate | Mean temperature in the breeding period * | K | ERA5-Land |
| Climate | Mean annual temperature * | K | ERA5-Land |
| Climate | Mean temperature of the coldest month * | K | ERA5-Land |
| Climate | Total annual precipitation * | mm | ERA5-Land |
| Climate | Total precipitation in the breeding period * | mm | ERA5-Land |
| Habitats struct. | Minimum NDVI in the breeding period * | DHI min | MODIS13 Vegetation Index |
| Habitats struct. | Accumulated NDVI in the breeding period * | DHI cum | MODIS13 Vegetation Index |
| Topography | Mean elevation | m | FAO; Fischer et al. 2008 |
| Land cover | Herbaceous cover * | % | ESA CCI Land Cover |
| Anthropogenic | Human population density * | Ind./km^2^ | Gridded Pop. of the World |
| Habitats struct. | Seasonality NDVI in the breeding period * | DHI var | MODIS13 Vegetation Index |
| Land cover | Rainfed cropland * | % | ESA CCI Land Cover |
| Topography | Mean slope | % | FAO; Fischer et al. 2008 |
| Land cover | Urban areas * | % | ESA CCI Land Cover |
| Land cover | Mosaic cropland-natural vegetation * | % | ESA CCI Land Cover |
| Land cover | Coniferous forests * | % | ESA CCI Land Cover |
| Land cover | Mosaic natural vegetation * | % | ESA CCI Land Cover |
| Habitats struct. | Shannon habitat diversity index * | - | EBBA2 and EBBALF |
| Land cover | Broadleaved forests * | % | ESA CCI Land Cover |
| Land cover | Grassland * | % | ESA CCI Land Cover |
| Land cover | Mixed broadleaved and coniferous forests * | % | ESA CCI Land Cover |
| Anthropogenic | Highway road density | m/km^2^ | Meijer et al. 2018 |
| Land cover | Irrigated crops * | % | ESA CCI Land Cover |
| Land cover | Rainfed tree crop * | % | ESA CCI Land Cover |
| Land cover | Continental water bodies * | % | ESA CCI Land Cover |
| Geography | Distance to the coastline | m | EBBALF |
| Land cover | Wetlands * | % | ESA CCI Land Cover |
| Land cover | Shrublands * | % | ESA CCI Land Cover |
| Land cover | Sparse vegetation * | % | ESA CCI Land Cover |
| Land cover | Bare areas * | % | ESA CCI Land Cover |
| Land cover | Permanent ice * | % | ESA CCI Land Cover |

**Appendix S5. Distribution maps.** The SDMs implemented in this study allowed us to robustly predict the species distribution at 10-km resolution for 50 farmland bird species (Appendices S6-S8). The resulting maps (Appendix S9) have had a very good acceptance across the community of European coordinators of bird monitoring schemes. However, for some species national data suggests that modeled distributions could be underrepresented. For example, this could be the case of the White Stork (*Ciconia ciconia*), the Little Owl (*Athene noctua*)*,* and the Tawny Pipit (*Anthus campestris*) in countries like Germany, Belgium and Poland, respectively (Appendix S9). One possible explanation is that data harmonization and the SDMs architecture do not fully reduce some occurrence biases, which could be different among countries (e.g., due to different sampling methods). Another plausible reason is that while covariates were measured at a 10-km resolution, species presence was collected at much finer scale at site level (usually 1 or 2 km but depending on the country) and SDMs could not always predict occurrences accurately in some mosaic landscapes.

In general, model validations were high or acceptable, even in low-sampled regions, such as Southeastern Europe but certainly validation statistics were usually lower in that region (Appendices S6-S8). Fortunately, ornithological organizations in North Macedonia, Albania, Serbia, Bosnia and Herzegovina, Kosovo, Montenegro, and Moldova have recently initiated bird monitoring efforts that are quickly improving this situation (EBCC 2024). We consider this study to provide valuable guidance on coverage goals for monitoring projects across Europe, not only in the Southeast but also in other regions (see Appendix S1).

**Appendix S6. AUC performance of distribution models.** Area Under the Curve (AUC) performance of distribution models for the 50 study species in the two study periods. The AUC value for 2013–2017 appears before the slash, and the value for 2018–2022 appears after the slash. AUCs are shown across the whole species range and by regions.

| **Scientific name** | **AUC Europe** | **AUC Central- Eastern** | **AUC North** | **AUC Southeast** | **AUC Southwest** | **AUC West** |
| --- | --- | --- | --- | --- | --- | --- |
| *Alauda arvensis* | 0.86 / 0.84 | 0.78 / 0.77 | 0.93 / 0.92 | 0.84 / 0.79 | 0.83 / 0.84 | 0.76 / 0.76 |
| *Alectoris rufa* | 0.87 / 0.87 | ー | ー | ー | 0.89 / 0.89 | 0.86 / 0.84 |
| *Anthus campestris* | 0.78 / 0.81 | 0.64 / 0.78 | ー / 0.41 | 0.78 / 0.78 | 0.82 / 0.81 | 0.88 / 0.81 |
| *Anthus pratensis* | 0.87 / 0.87 | 0.78 / 0.78 | 0.88 / 0.89 | ー / 0.44 | 0.72 / 0.68 | 0.87 / 0.87 |
| *Athene noctua* | 0.83 / 0.85 | 0.87 / 0.89 | ー | 0.68 / 0.62 | 0.81 / 0.82 | 0.89 / 0.83 |
| *Bubulcus ibis* | 0.88 / 0.85 | ー | ー | 0.98 / 0.96 | 0.89 / 0.86 | ー |
| *Burhinus oedicnemus* | 0.88 / 0.90 | ー | ー | 0.58 / 0.56 | 0.90 / 0.90 | ー |
| *Calandrella brachydactyla* | 0.92 / 0.91 | ー | ー | 0.87 / 0.89 | 0.94 / 0.93 | ー |
| *Ciconia ciconia* | 0.88 / 0.85 | 0.77 / 0.75 | ー / 0.88 | 0.65 / 0.59 | 0.90 / 0.90 | 0.71 / 0.67 |
| *Circus pygargus* | 0.77 / 0.79 | 0.72 / 0.7 | 0.75 / 0.95 | 0.75 / 0.58 | 0.83 / 0.86 | 0.76 / 0.74 |
| *Coracias garrulus* | 0.81 / 0.86 | 0.8 / 0.9 | ー | 0.82 / 0.85 | 0.84 / 0.88 | 0.44 / 0.57 |
| *Corvus frugilegus* | 0.84 / 0.84 | 0.72 / 0.68 | 0.92 / 0.92 | 0.81 / 0.80 | 0.69 / 0.70 | 0.90 / 0.89 |
| *Coturnix coturnix* | 0.83 / 0.84 | 0.7 / 0.69 | 0.90 / 0.95 | 0.86 / 0.82 | 0.80 / 0.82 | 0.79 / 0.78 |
| *Crex crex* | 0.88 / 0.86 | 0.76 / 0.78 | 0.89 / 0.85 | 0.75 / 0.57 | 0.96 / ー | 0.77 / 0.73 |
| *Curruca communis* | 0.84 / 0.83 | 0.72 / 0.68 | 0.93 / 0.93 | 0.64 / 0.60 | 0.85 / 0.84 | 0.82 / 0.80 |
| *Emberiza calandra* | 0.86 / 0.86 | 0.74 / 0.72 | ー | 0.82 / 0.77 | 0.85 / 0.85 | 0.82 / 0.83 |
| *Emberiza cirlus* | 0.85 / 0.85 | ー | ー | 0.92 / 0.86 | 0.80 / 0.80 | 0.71 / 0.69 |
| *Emberiza citrinella* | 0.09 / 0.89 | 0.84 / 0.81 | 0.92 / 0.93 | 0.82 / 0.84 | 0.86 / 0.85 | 0.91 / 0.89 |
| *Emberiza hortulana* | 0.78 / 0.81 | 0.82 / 0.78 | 0.58 / 0.74 | 0.78 / 0.78 | 0.80 / 0.83 | 0.84 / 0.81 |
| *Emberiza melanocephala* | 0.84 / 0.79 | ー | ー | 0.80 / 0.76 | 0.88 / 0.94 | ー |
| *Falco naumanni* | 0.83 / 0.85 | ー | ー | ー | 0.84 / 0.87 | ー |
| *Falco tinnunculus* | 0.76 / 0.77 | 0.75 / 0.77 | 0.54 / 0.61 | 0.69 / 0.65 | 0.69 / 0.68 | 0.72 / 0.73 |
| *Galerida cristata* | 0.92 / 0.91 | 0.88 / 0.91 | ー | 0.74 / 0.71 | 0.93 / 0.93 | 0.77 / 0.7 |
| *Galerida theklae* | 0.83 / 0.83 | ー | ー | ー | 0.84 / 0.84 | ー |
| *Hirundo rustica* | 0.83 / 0.81 | 0.68 / 0.64 | 0.88 / 0.87 | 0.68 / 0.72 | 0.8 / 0.78 | 0.82 / 0.79 |
| *Lanius collurio* | 0.80 / 0.79 | 0.61 / 0.61 | 0.86 / 0.85 | 0.56 / 0.58 | 0.79 / 0.79 | 0.76 / 0.77 |
| *Lanius excubitor* | 0.76 / 0.75 | 0.60 / 0.64 | 0.77 / 0.59 | 0.62 / 0.55 | 0.54 / 0.60 | 0.83 / 0.75 |
| *Lanius meridionalis* | 0.77 / 0.80 | ー | ー | ー | 0.78 / 0.82 | ー |
| *Lanius minor* | 0.79 / 0.78 | 0.85 / 0.91 | ー | 0.68 / 0.60 | 0.82 / 0.85 | ー |
| *Lanius senator* | 0.85 / 0.85 | ー | ー | 0.74 / 0.74 | 0.87 / 0.87 | ー |
| *Limosa limosa* | 0.74 / 0.75 | 0.64 / 0.67 | 1.00 / ー | 0.86 / 0.80 | ー | 0.85 / 0.81 |
| *Linaria cannabina* | 0.78 / 0.78 | 0.70 / 0.69 | 0.91 / 0.92 | 0.55 / 0.62 | 0.77 / 0.79 | 0.73 / 0.72 |
| *Melanocorypha calandra* | 0.91 / 0.90 | ー | ー | 0.88 / 0.80 | 0.93 / 0.92 | ー |
| *Motacilla flava* | 0.83 / 0.83 | 0.80 / 0.79 | 0.85 / 0.87 | 0.86 / 0.84 | 0.83 / 0.84 | 0.80 / 0.80 |
| *Oenanthe hispanica* | 0.82 / 0.84 | ー | ー | 0.68 / 0.66 | 0.85 / 0.87 | ー |
| *Passer hispaniolensis* | 0.82 / 0.81 | ー | ー | 0.75 / 0.74 | 0.87 / 0.86 | ー |
| *Passer montanus* | 0.84 / 0.84 | 0.75 / 0.73 | 0.90 / 0.90 | 0.69 / 0.69 | 0.84 / 0.84 | 0.85 / 0.85 |
| *Perdix perdix* | 0.77 / 0.78 | 0.75 / 0.73 | 0.91 / 0.88 | 0.68 / 0.64 | 0.85 / 0.88 | 0.77 / 0.79 |
| *Petronia petronia* | 0.82 / 0.84 | ー | ー | ー | 0.83 / 0.85 | ー |
| *Pterocles alchata* | 0.87 / 0.92 | ー | ー | ー | 0.87 / 0.92 | ー |
| *Pterocles orientalis* | 0.88 / 0.88 | ー | ー | ー | 0.88 / 0.88 | ー |
| *Saxicola rubetra* | 0.86 / 0.85 | 0.66 / 0.68 | 0.76 / 0.77 | 0.71 / 0.68 | 0.82 / 0.86 | 0.81 / 0.78 |
| *Saxicola torquatus* | 0.78 / 0.77 | 0.76 / 0.76 | 1.00 / 1.00 | 0.72 / 0.74 | 0.72 / 0.74 | 0.75 / 0.73 |
| *Serinus serinus* | 0.86 / 0.86 | 0.71 / 0.70 | ー / 0.14 | 0.88 / 0.88 | 0.85 / 0.86 | 0.79 / 0.78 |
| *Streptopelia turtur* | 0.83 / 0.85 | 0.72 / 0.72 | 0.13 / ー | 0.70 / 0.71 | 0.76 / 0.78 | 0.79 / 0.80 |
| *Sturnus unicolor* | 0.93 / 0.88 | ー | ー | ー | 0.94 / 0.90 | ー |
| *Sturnus vulgaris* | 0.88 / 0.88 | 0.81 / 0.80 | 0.92 / 0.92 | 0.83 / 0.73 | 0.90 / 0.92 | 0.79 / 0.78 |
| *Tetrax tetrax* | 0.90 / 0.90 | ー | ー | ー | 0.93 / 0.92 | ー |
| *Upupa epops* | 0.86 / 0.86 | 0.76 / 0.77 | ー | 0.71 / 0.61 | 0.84 / 0.85 | 0.82 / 0.82 |
| *Vanellus vanellus* | 0.83 / 0.82 | 0.72 / 0.73 | 0.86 / 0.88 | 0.77 / 0.73 | 0.84 / 0.84 | 0.80 / 0.78 |
| **Mean** | **0.84 / 0.84** | **0.74 / 0.75** | **0.83 / 0.81** | **0.75 / 0.71** | **0.83 / 0.84** | **0.79 / 0.78** |

**Appendix S7. PR-AUC performance of distribution models.** Precision-Recall Area Under the Curve (PR-AUC) performance of distribution models for the 50 study species in the two study periods. The PR-AUC value for 2013–2017 appears before the slash, and the value for 2018–2022 appears after the slash. PR-AUCs are shown across the whole species range and by regions.

| **Scientific name** | **PR-AUC Europe** | **PR-AUC Central- Eastern** | **PR-AUC North** | **PR-AUC Southeast** | **PR-AUC Southwest** | **PR-AUC West** |
| --- | --- | --- | --- | --- | --- | --- |
| *Alauda arvensis* | 0.75 / 0.73 | 0.91 / 0.90 | 0.62 / 0.61 | 0.89 / 0.87 | 0.72 / 0.71 | 0.74 / 0.72 |
| *Alectoris rufa* | 0.67 / 0.64 | ー | ー | ー | 0.73 / 0.73 | 0.56 / 0.52 |
| *Anthus campestris* | 0.33 / 0.32 | 0.27 / 0.31 | ー / 0.06 | 0.54 / 0.51 | 0.35 / 0.30 | 0.13 / 0.04 |
| *Anthus pratensis* | 0.64 / 0.64 | 0.52 / 0.48 | 0.78 / 0.76 | ー / 0.05 | 0.09 / 0.04 | 0.66 / 0.66 |
| *Athene noctua* | 0.46 / 0.40 | 0.07 / 0.01 | ー | 0.41 / 0.24 | 0.52 / 0.45 | 0.29 / 0.10 |
| *Bubulcus ibis* | 0.42 / 0.42 | ー | ー | 0.06 / 0.06 | 0.45 / 0.46 | ー |
| *Burhinus oedicnemus* | 0.45 / 0.43 | ー | ー | 0.11 / 0.04 | 0.47 / 0.48 | ー |
| *Calandrella brachydactyla* | 0.60 / 0.58 | ー | ー | 0.52 / 0.62 | 0.64 / 0.61 | ー |
| *Ciconia ciconia* | 0.52 / 0.47 | 0.64 / 0.57 | ー / 0.07 | 0.52 / 0.36 | 0.59 / 0.56 | 0.07 / 0.06 |
| *Circus pygargus* | 0.38 / 0.36 | 0.29 / 0.22 | 0.27 / 0.17 | 0.63 / 0.30 | 0.47 / 0.49 | 0.12 / 0.05 |
| *Coracias garrulus* | 0.26 / 0.32 | 0.22 / 0.44 | ー | 0.32 / 0.35 | 0.27 / 0.32 | 0.07 / 0.08 |
| *Corvus frugilegus* | 0.58 / 0.56 | 0.40 / 0.32 | 0.21 / 0.22 | 0.60 / 0.62 | 0.37 / 0.38 | 0.73 / 0.69 |
| *Coturnix coturnix* | 0.54 / 0.51 | 0.52 / 0.43 | 0.32 / 0.18 | 0.86 / 0.76 | 0.60 / 0.64 | 0.25 / 0.22 |
| *Crex crex* | 0.36 / 0.24 | 0.51 / 0.40 | 0.42 / 0.19 | 0.40 / 0.11 | 0.07 / ー | 0.04 / 0.02 |
| *Curruca communis* | 0.74 / 0.72 | 0.86 / 0.84 | 0.72 / 0.73 | 0.75 / 0.73 | 0.70 / 0.67 | 0.76 / 0.75 |
| *Emberiza calandra* | 0.65 / 0.66 | 0.62 / 0.66 | ー | 0.82 / 0.82 | 0.77 / 0.76 | 0.32 / 0.33 |
| *Emberiza cirlus* | 0.68 / 0.69 | ー | ー | 0.52 / 0.45 | 0.72 / 0.74 | 0.14 / 0.05 |
| *Emberiza citrinella* | 0.81 / 0.79 | 0.94 / 0.94 | 0.81 / 0.80 | 0.72 / 0.69 | 0.66 / 0.62 | 0.84 / 0.81 |
| *Emberiza hortulana* | 0.32 / 0.27 | 0.43 / 0.26 | 0.14 / 0.07 | 0.50 / 0.50 | 0.26 / 0.21 | 0.29 / 0.24 |
| *Emberiza melanocephala* | 0.51 / 0.41 | ー | ー | 0.58 / 0.52 | 0.13 / 0.18 | ー |
| *Falco naumanni* | 0.51 / 0.52 | ー | ー | ー | 0.54 / 0.53 | ー |
| *Falco tinnunculus* | 0.67 / 0.64 | 0.53 / 0.54 | 0.29 / 0.30 | 0.73 / 0.70 | 0.81 / 0.78 | 0.60 / 0.55 |
| *Galerida cristata* | 0.62 / 0.60 | 0.41 / 0.44 | ー | 0.35 / 0.36 | 0.77 / 0.75 | 0.12 / 0.06 |
| *Galerida theklae* | 0.55 / 0.57 | ー | ー | ー | 0.56 / 0.58 | ー |
| *Hirundo rustica* | 0.83 / 0.81 | 0.87 / 0.84 | 0.72 / 0.71 | 0.88 / 0.84 | 0.94 / 0.93 | 0.84 / 0.81 |
| *Lanius collurio* | 0.65 / 0.63 | 0.80 / 0.78 | 0.58 / 0.56 | 0.82 / 0.80 | 0.60 / 0.57 | 0.53 / 0.53 |
| *Lanius excubitor* | 0.28 / 0.22 | 0.28 / 0.26 | 0.44 / 0.15 | 0.68 / 0.40 | 0.01 / 0.01 | 0.18 / 0.12 |
| *Lanius meridionalis* | 0.55 / 0.53 | ー | ー | ー | 0.55 / 0.57 | ー |
| *Lanius minor* | 0.34 / 0.27 | 0.45 / 0.59 | ー | 0.38 / 0.27 | 0.30 / 0.14 | ー |
| *Lanius senator* | 0.54 / 0.52 | ー | ー | 0.42 / 0.31 | 0.56 / 0.55 | ー |
| *Limosa limosa* | 0.32 / 0.33 | 0.33 / 0.06 | 0.50 / ー | 0.08 / 0.07 | ー | 0.39 / 0.28 |
| *Linaria cannabina* | 0.64 / 0.62 | 0.66 / 0.62 | 0.46 / 0.53 | 0.37 / 0.37 | 0.72 / 0.75 | 0.64 / 0.64 |
| *Melanocorypha calandra* | 0.64 / 0.62 | ー | ー | 0.53 / 0.41 | 0.70 / 0.68 | ー |
| *Motacilla flava* | 0.55 / 0.55 | 0.70 / 0.68 | 0.67 / 0.65 | 0.74 / 0.72 | 0.46 / 0.48 | 0.39 / 0.38 |
| *Oenanthe hispanica* | 0.42 / 0.42 | ー | ー | 0.58 / 0.30 | 0.42 / 0.45 | ー |
| *Passer hispaniolensis* | 0.52 / 0.53 | ー | ー | 0.44 / 0.43 | 0.58 / 0.60 | ー |
| *Passer montanus* | 0.62 / 0.61 | 0.76 / 0.73 | 0.50 / 0.50 | 0.67 / 0.58 | 0.62 / 0.61 | 0.64 / 0.61 |
| *Perdix perdix* | 0.30 / 0.25 | 0.38 / 0.30 | 0.75 / 0.32 | 0.32 / 0.16 | 0.49 / 0.41 | 0.27 / 0.23 |
| *Petronia petronia* | 0.53 / 0.56 | ー | ー | ー | 0.56 / 0.59 | ー |
| *Pterocles alchata* | 0.59 / 0.66 | ー | ー | ー | 0.59 / 0.66 | ー |
| *Pterocles orientalis* | 0.32 / 0.35 | ー | ー | ー | 0.32 / 0.35 | ー |
| *Saxicola rubetra* | 0.57 / 0.52 | 0.68 / 0.61 | 0.64 / 0.63 | 0.59 / 0.45 | 0.29 / 0.31 | 0.28 / 0.25 |
| *Saxicola torquatus* | 0.58 / 0.54 | 0.50 / 0.48 | 0.18 / 0.18 | 0.64 / 0.55 | 0.69 / 0.70 | 0.38 / 0.40 |
| *Serinus serinus* | 0.71 / 0.70 | 0.50 / 0.45 | ー / 0.01 | 0.63 / 0.63 | 0.88 / 0.88 | 0.50 / 0.45 |
| *Streptopelia turtur* | 0.65 / 0.64 | 0.51 / 0.43 | 0.02 / ー | 0.63 / 0.61 | 0.76 / 0.76 | 0.30 / 0.27 |
| *Sturnus unicolor* | 0.93 / 0.91 | ー | ー | ー | 0.94 / 0.93 | ー |
| *Sturnus vulgaris* | 0.84 / 0.83 | 0.94 / 0.94 | 0.64 / 0.65 | 0.92 / 0.88 | 0.89 / 0.90 | 0.83 / 0.81 |
| *Tetrax tetrax* | 0.48 / 0.50 | ー | ー | ー | 0.55 / 0.53 | ー |
| *Upupa epops* | 0.64 / 0.67 | 0.45 / 0.52 | ー | 0.66 / 0.63 | 0.76 / 0.78 | 0.26 / 0.19 |
| *Vanellus vanellus* | 0.50 / 0.46 | 0.62 / 0.59 | 0.54 / 0.56 | 0.48 / 0.41 | 0.29 / 0.28 | 0.47 / 0.41 |
| **Mean** | **0.56 / 0.54** | **0.55 / 0.52** | **0.49 / 0.40** | **0.56 / 0.48** | **0.55 / 0.55** | **0.41 / 0.37** |

**Appendix S8. MSE performance of distribution models.** Mean squared error (MSE) performance of distribution models for the 50 study species in the two study periods. The MSE value for 2013–2017 appears before the slash, and the value for 2018–2022 appears after the slash. MSEs are shown across the whole species range and by regions.

| **Scientific name** | **MSE Europe** | **MSE Central- Eastern** | **MSE North** | **MSE Southeast** | **MSE Southwest** | **MSE West** |
| --- | --- | --- | --- | --- | --- | --- |
| *Alauda arvensis* | 0.15 / 0.16 | 0.10 / 0.12 | 0.09 / 0.10 | 0.13 / 0.16 | 0.17 / 0.17 | 0.19 / 0.19 |
| *Alectoris rufa* | 0.13 / 0.14 | ー | ー | ー | 0.13 / 0.14 | 0.12 / 0.13 |
| *Anthus campestris* | 0.12 / 0.10 | 0.17 / 0.10 | ー / 0.09 | 0.19 / 0.17 | 0.11 / 0.10 | 0.03 / 0.03 |
| *Anthus pratensis* | 0.14 / 0.13 | 0.17 / 0.15 | 0.14 / 0.14 | ー / 0.07 | 0.04 / 0.03 | 0.14 / 0.14 |
| *Athene noctua* | 0.12 / 0.12 | 0.03 / 0.03 | ー | 0.17 / 0.14 | 0.16 / 0.16 | 0.07 / 0.04 |
| *Bubulcus ibis* | 0.09 / 0.11 | ー | ー | 0.04 / 0.05 | 0.09 / 0.11 | ー |
| *Burhinus oedicnemus* | 0.08 / 0.08 | ー | ー | 0.10 / 0.04 | 0.08 / 0.08 | ー |
| *Calandrella brachydactyla* | 0.09 / 0.08 | ー | ー | 0.12 / 0.11 | 0.08 / 0.08 | ー |
| *Ciconia ciconia* | 0.11 / 0.12 | 0.20 / 0.21 | ー / 0.08 | 0.25 / 0.22 | 0.09 / 0.09 | 0.04 / 0.04 |
| *Circus pygargus* | 0.14 / 0.12 | 0.14 / 0.13 | 0.18 / 0.13 | 0.25 / 0.17 | 0.12 / 0.11 | 0.06 / 0.05 |
| *Coracias garrulus* | 0.08 / 0.07 | 0.06 / 0.07 | ー | 0.11 / 0.09 | 0.07 / 0.06 | 0.12 / 0.12 |
| *Corvus frugilegus* | 0.15 / 0.16 | 0.18 / 0.17 | 0.03 / 0.03 | 0.17 / 0.19 | 0.18 / 0.18 | 0.13 / 0.14 |
| *Coturnix coturnix* | 0.15 / 0.15 | 0.22 / 0.23 | 0.05 / 0.03 | 0.16 / 0.18 | 0.18 / 0.18 | 0.10 / 0.10 |
| *Crex crex* | 0.08 / 0.07 | 0.17 / 0.15 | 0.08 / 0.07 | 0.16 / 0.13 | 0.01 / ー | 0.02 / 0.02 |
| *Curruca communis* | 0.16 / 0.17 | 0.14 / 0.15 | 0.11 / 0.11 | 0.19 / 0.20 | 0.16 / 0.17 | 0.17 / 0.18 |
| *Emberiza calandra* | 0.15 / 0.16 | 0.21 / 0.22 | ー | 0.16 / 0.17 | 0.16 / 0.16 | 0.10 / 0.11 |
| *Emberiza cirlus* | 0.16 / 0.17 | ー | ー | 0.09 / 0.14 | 0.18 / 0.18 | 0.04 / 0.07 |
| *Emberiza citrinella* | 0.13 / 0.13 | 0.08 / 0.08 | 0.11 / 0.11 | 0.17 / 0.17 | 0.15 / 0.15 | 0.12 / 0.14 |
| *Emberiza hortulana* | 0.11 / 0.09 | 0.12 / 0.10 | 0.09 / 0.04 | 0.17 / 0.17 | 0.08 / 0.06 | 0.08 / 0.08 |
| *Emberiza melanocephala* | 0.13 / 0.14 | ー | ー | 0.17 / 0.20 | 0.05 / 0.04 | ー |
| *Falco naumanni* | 0.14 / 0.13 | ー | ー | ー | 0.13 / 0.12 | ー |
| *Falco tinnunculus* | 0.20 / 0.23 | 0.20 / 0.23 | 0.20 / 0.21 | 0.20 / 0.22 | 0.17 / 0.18 | 0.22 / 0.22 |
| *Galerida cristata* | 0.09 / 0.10 | 0.07 / 0.07 | ー | 0.16 / 0.18 | 0.10 / 0.11 | 0.03 / 0.03 |
| *Galerida theklae* | 0.16 / 0.16 | ー | ー | ー | 0.15 / 0.15 | ー |
| *Hirundo rustica* | 0.13 / 0.15 | 0.13 / 0.15 | 0.14 / 0.16 | 0.11 / 0.14 | 0.08 / 0.08 | 0.16 / 0.19 |
| *Lanius collurio* | 0.18 / 0.19 | 0.17 / 0.19 | 0.15 / 0.15 | 0.16 / 0.18 | 0.19 / 0.19 | 0.20 / 0.20 |
| *Lanius excubitor* | 0.13 / 0.11 | 0.18 / 0.18 | 0.21 / 0.10 | 0.42 / 0.24 | 0.01 / 0.02 | 0.04 / 0.05 |
| *Lanius meridionalis* | 0.20 / 0.18 | ー | ー | ー | 0.19 / 0.17 | ー |
| *Lanius minor* | 0.13 / 0.11 | 0.14 / 0.12 | ー | 0.20 / 0.17 | 0.06 / 0.04 | ー |
| *Lanius senator* | 0.15 / 0.16 | ー | ー | 0.16 / 0.16 | 0.14 / 0.15 | ー |
| *Limosa limosa* | 0.13 / 0.12 | 0.20 / 0.07 | 0.31 / ー | 0.08 / 0.07 | ー | 0.09 / 0.09 |
| *Linaria cannabina* | 0.19 / 0.20 | 0.22 / 0.22 | 0.08 / 0.10 | 0.22 / 0.22 | 0.19 / 0.19 | 0.21 / 0.22 |
| *Melanocorypha calandra* | 0.10 / 0.10 | ー | ー | 0.10 / 0.10 | 0.09 / 0.09 | ー |
| *Motacilla flava* | 0.15 / 0.15 | 0.19 / 0.19 | 0.15 / 0.14 | 0.16 / 0.17 | 0.13 / 0.13 | 0.14 / 0.13 |
| *Oenanthe hispanica* | 0.14 / 0.12 | ー | ー | 0.26 / 0.21 | 0.13 / 0.10 | ー |
| *Passer hispaniolensis* | 0.14 / 0.15 | ー | ー | 0.17 / 0.16 | 0.12 / 0.14 | ー |
| *Passer montanus* | 0.16 / 0.16 | 0.19 / 0.20 | 0.10 / 0.10 | 0.23 / 0.25 | 0.16 / 0.15 | 0.16 / 0.15 |
| *Perdix perdix* | 0.12 / 0.10 | 0.16 / 0.14 | 0.29 / 0.11 | 0.17 / 0.09 | 0.08 / 0.06 | 0.11 / 0.09 |
| *Petronia petronia* | 0.16 / 0.15 | ー | ー | ー | 0.15 / 0.14 | ー |
| *Pterocles alchata* | 0.14 / 0.05 | ー | ー | ー | 0.14 / 0.05 | ー |
| *Pterocles orientalis* | 0.07 / 0.06 | ー | ー | ー | 0.07 / 0.06 | ー |
| *Saxicola rubetra* | 0.14 / 0.13 | 0.22 / 0.22 | 0.19 / 0.19 | 0.22 / 0.21 | 0.05 / 0.04 | 0.09 / 0.09 |
| *Saxicola torquatus* | 0.19 / 0.20 | 0.17 / 0.18 | 0.11 / 0.12 | 0.22 / 0.22 | 0.20 / 0.20 | 0.16 / 0.20 |
| *Serinus serinus* | 0.19 / 0.16 | 0.21 / 0.20 | ー / 0.04 | 0.14 / 0.16 | 0.14 / 0.12 | 0.17 / 0.17 |
| *Streptopelia turtur* | 0.17 / 0.19 | 0.21 / 0.22 | 0.04 / ー | 0.22 / 0.26 | 0.18 / 0.18 | 0.11 / 0.14 |
| *Sturnus unicolor* | 0.09 / 0.12 | ー | ー | ー | 0.08 / 0.11 | ー |
| *Sturnus vulgaris* | 0.12 / 0.13 | 0.07 / 0.08 | 0.10 / 0.10 | 0.10 / 0.12 | 0.12 / 0.11 | 0.14 / 0.15 |
| *Tetrax tetrax* | 0.08 / 0.07 | ー | ー | ー | 0.07 / 0.06 | ー |
| *Upupa epops* | 0.15 / 0.16 | 0.18 / 0.18 | ー | 0.21 / 0.24 | 0.16 / 0.15 | 0.05 / 0.06 |
| *Vanellus vanellus* | 0.15 / 0.14 | 0.22 / 0.21 | 0.13 / 0.12 | 0.18 / 0.18 | 0.07 / 0.07 | 0.16 / 0.15 |
| **Mean** | **0.14 / 0.14** | **0.16 / 0.16** | **0.13 / 0.11** | **0.17 / 0.16** | **0.12 / 0.12** | **0.11 / 0.12** |

**Appendix S9. Distribution maps for the period 2018–2022.** Distribution maps for the 50 farmland bird species included in this study for the period 2018–2022. Greener values show a higher probability of occurrence, while whiter values show a lower probability of occurrence. All species maps performed well for the whole of the study area, but in some cases, the performance was less reliable at the regional level (see Appendices S6-S8).

| 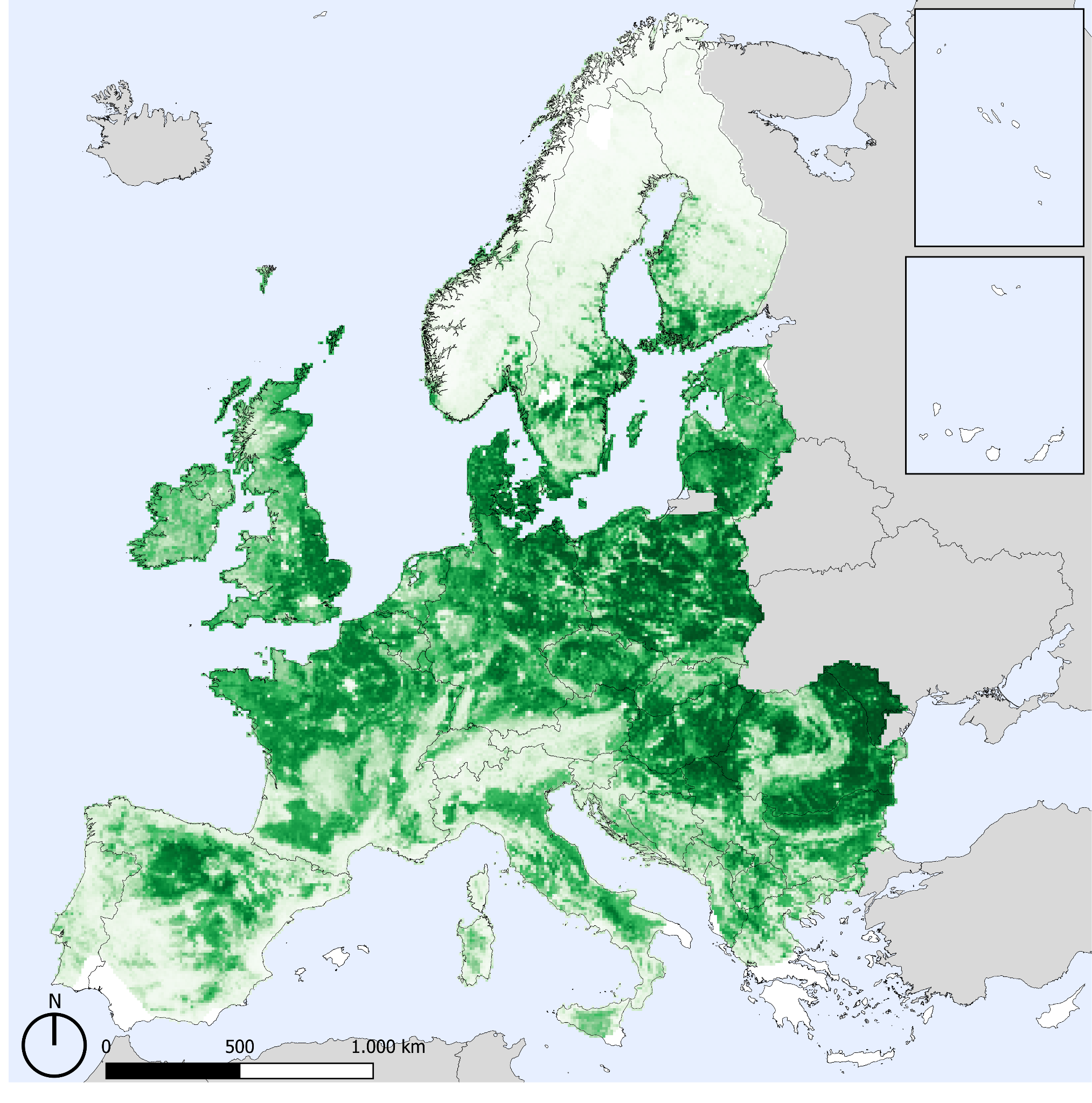 | 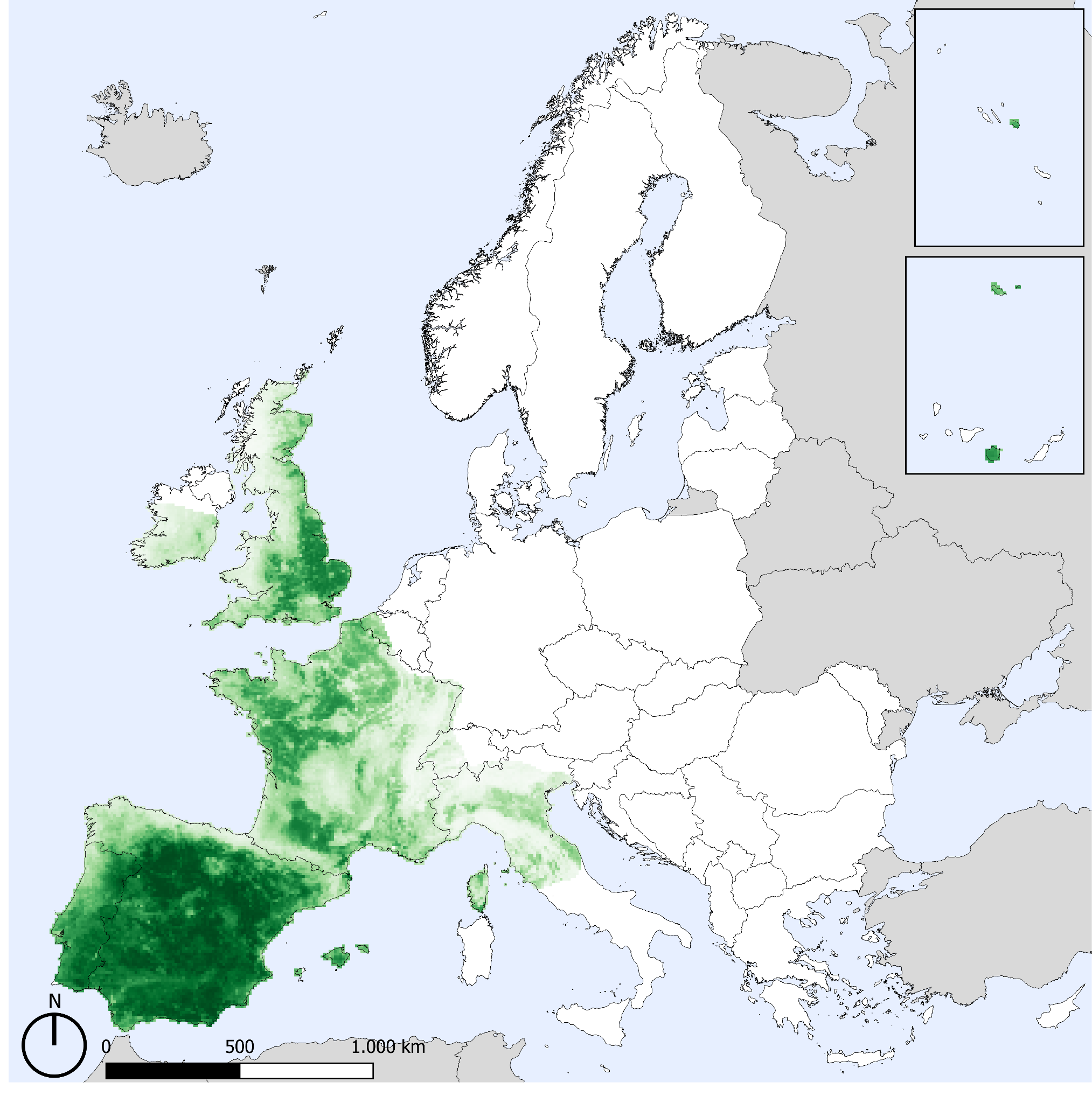 |
| --- | --- |
| *Alauda arvensis* | *Alectoris rufa* |
| 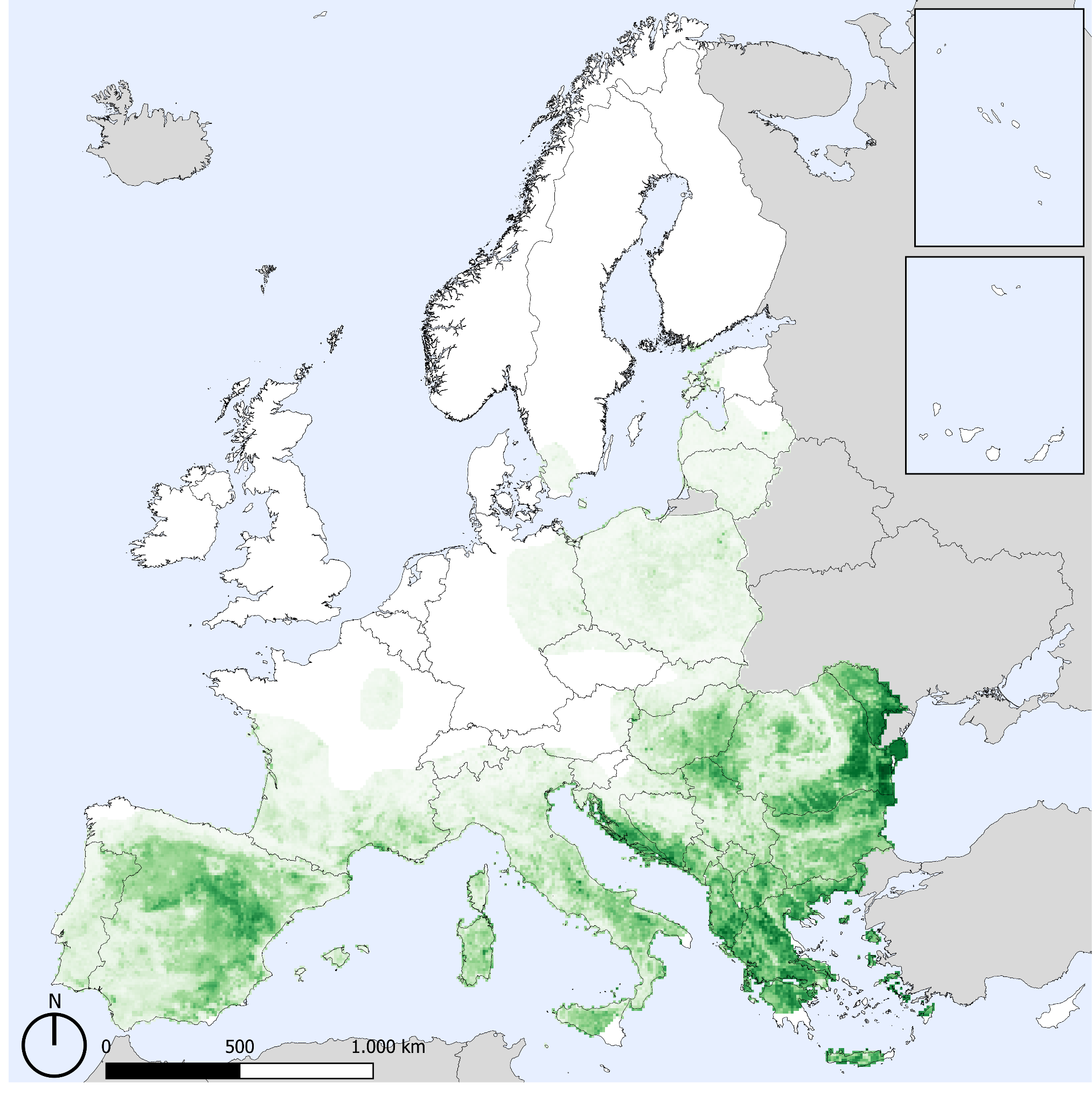 | 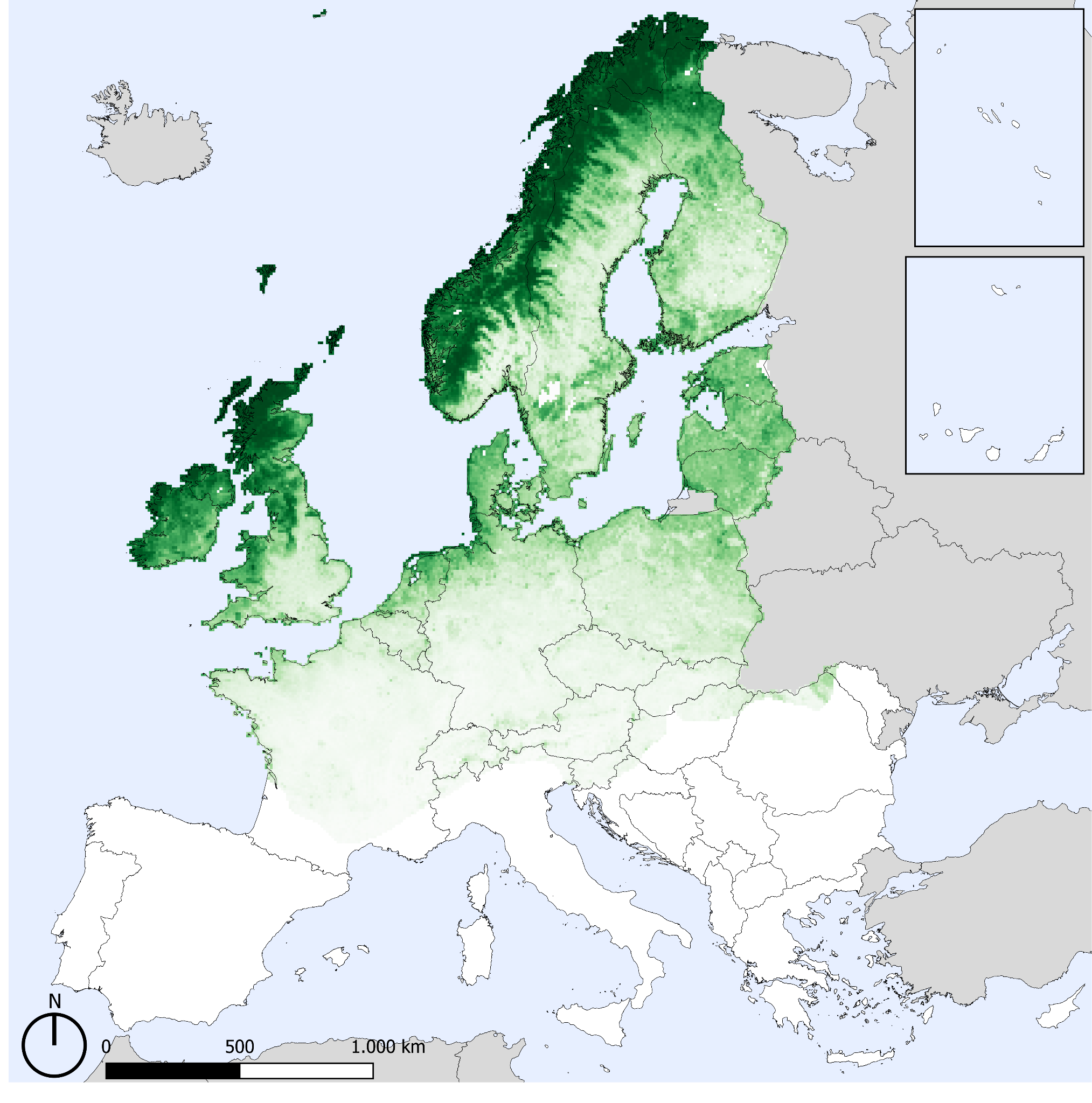 |
| *Anthus campestris* | *Anthus pratensis* |
| 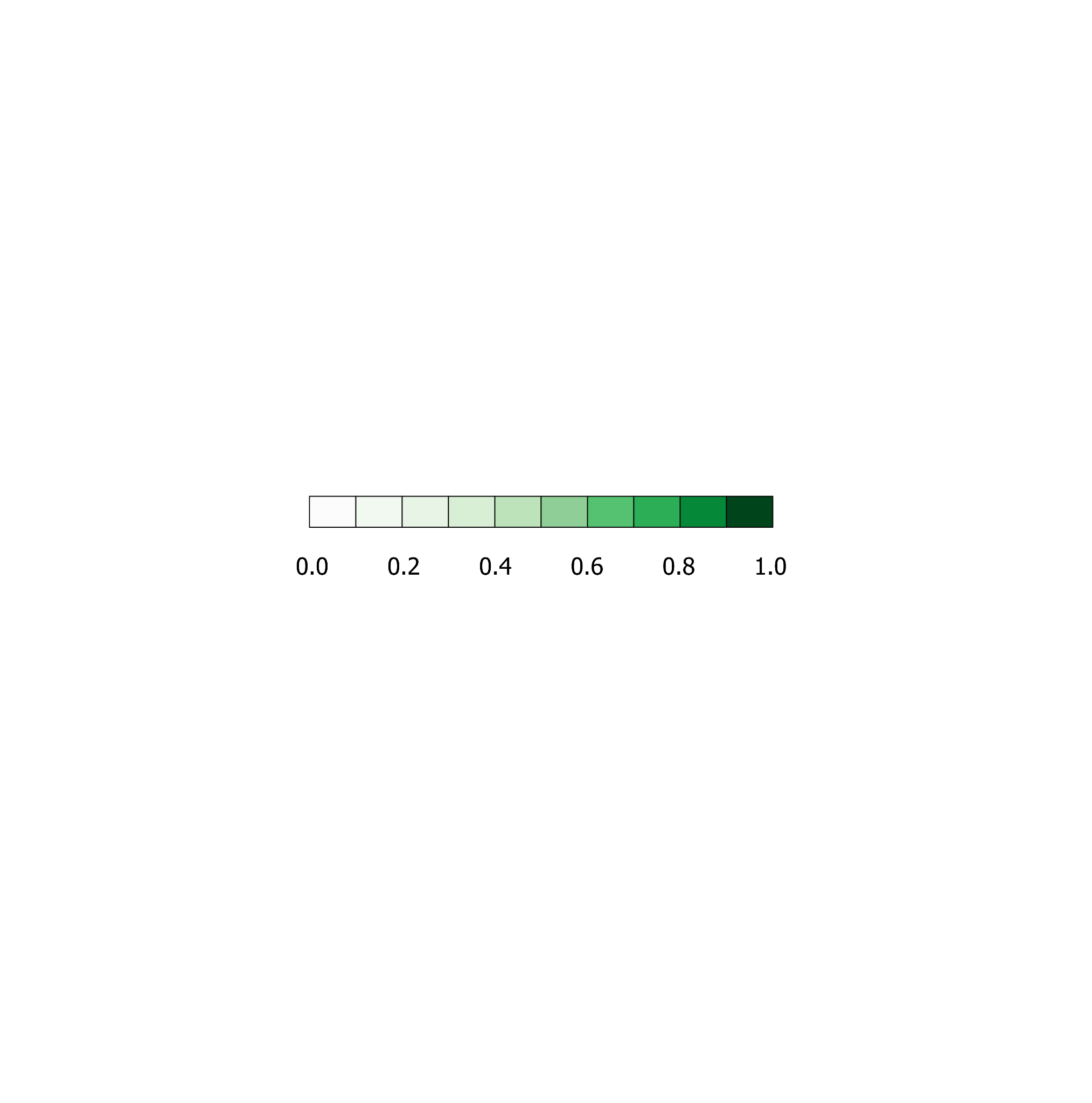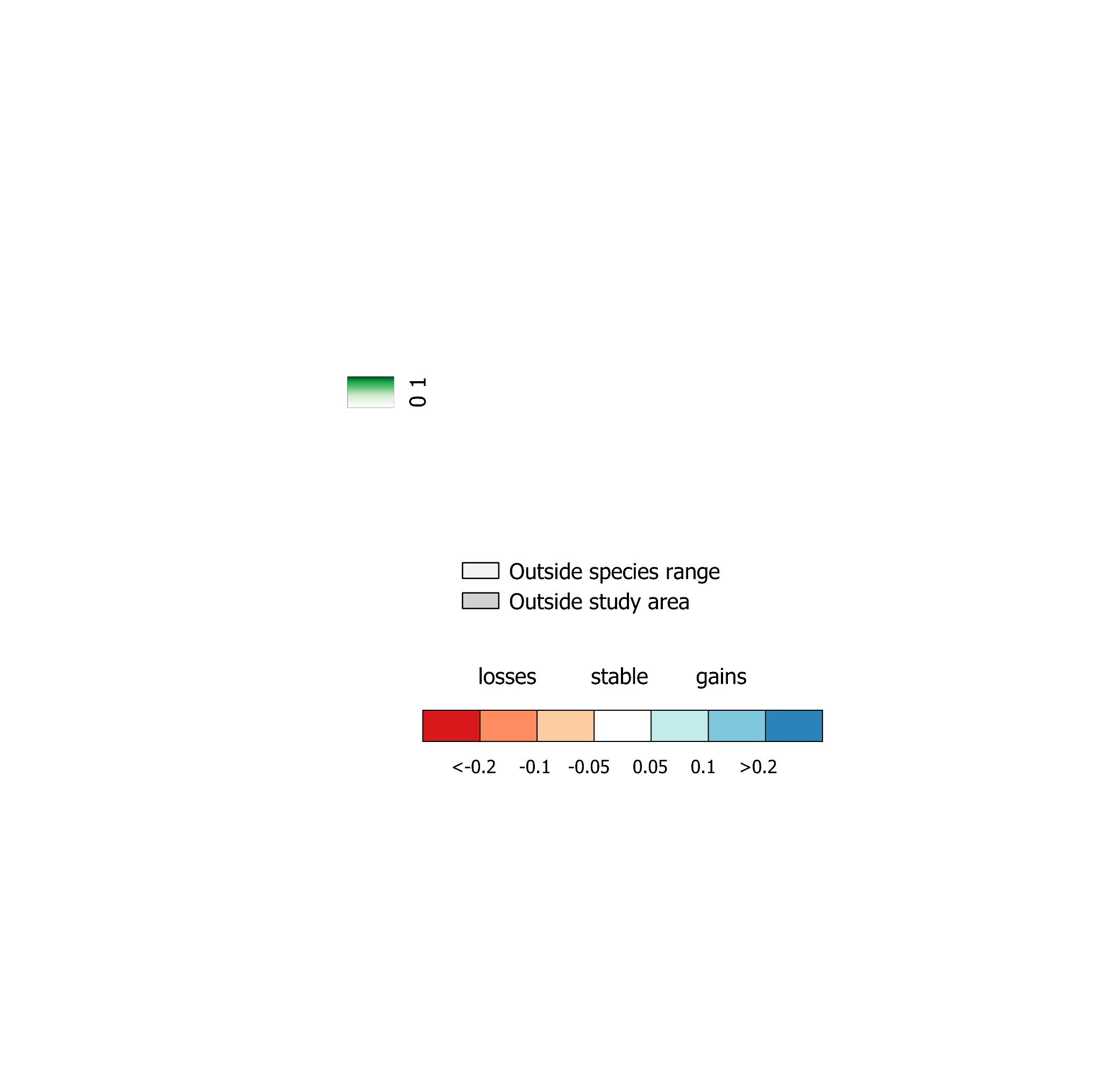  Occurrence probability | |
| 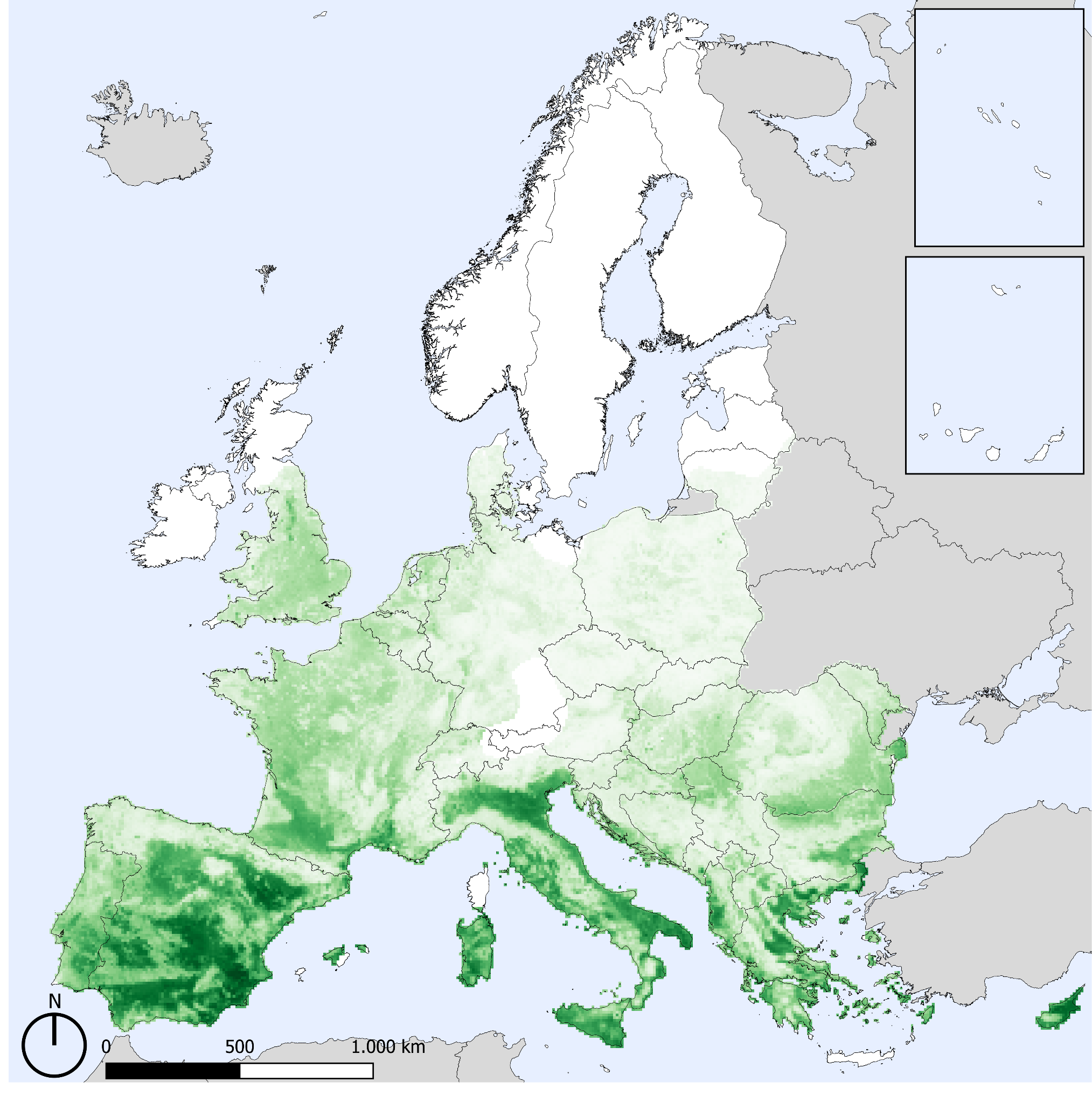 | 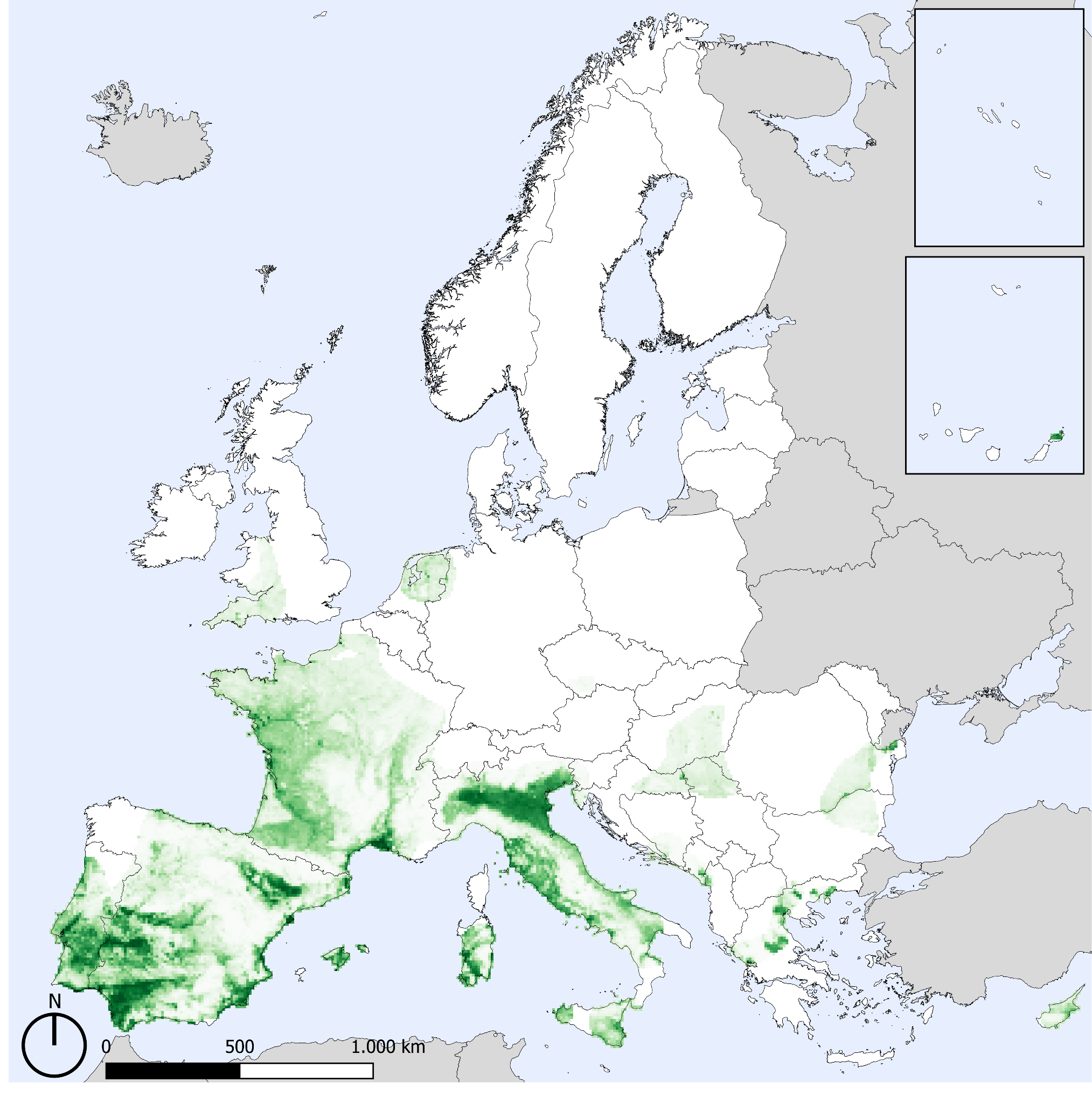 |
| *Athene noctua* | *Bubulcus ibis* |
| 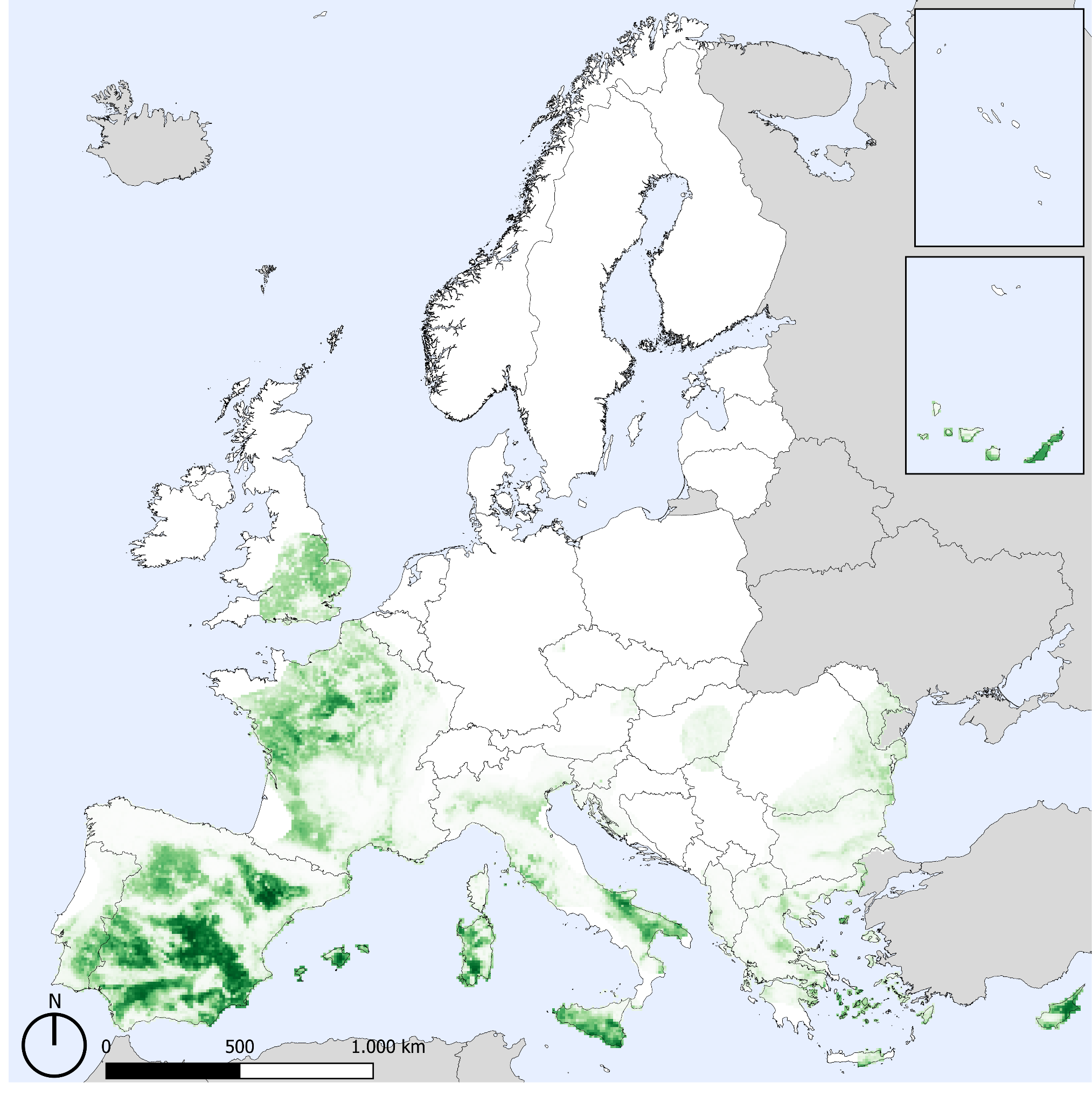 | 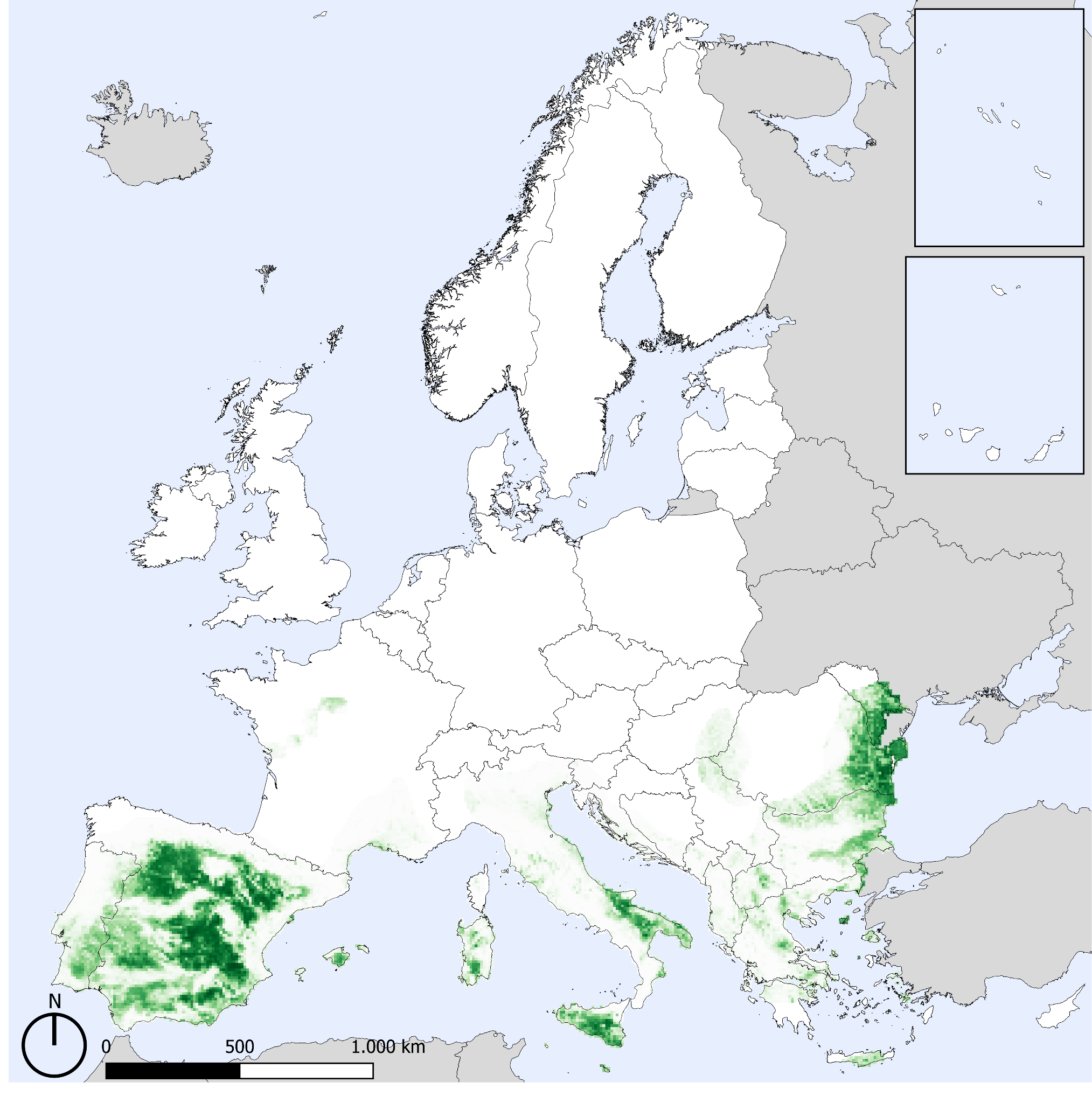 |
| *Burhinus oedicnemus* | *Calandrella brachydactyla* |
| 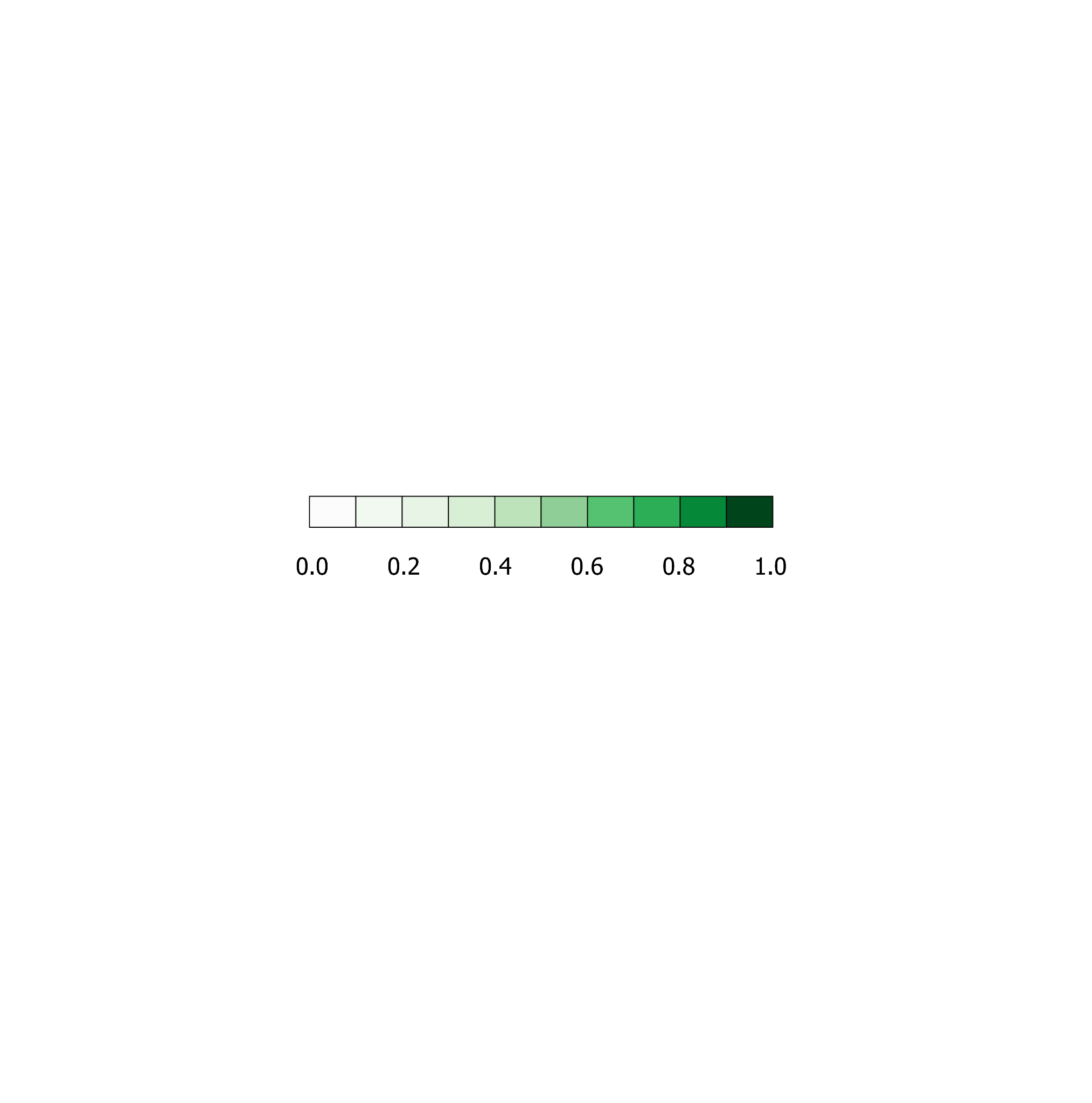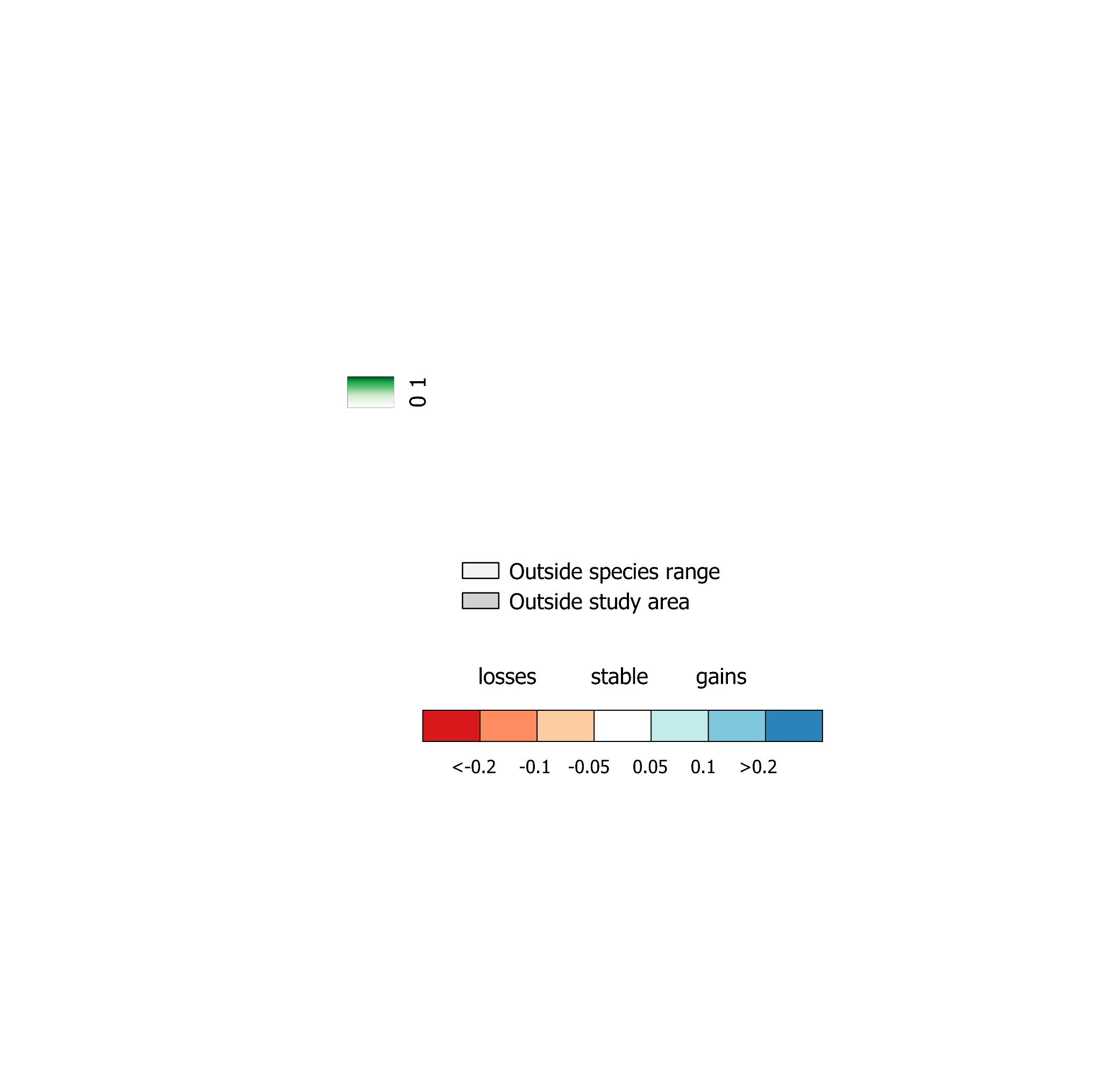  Occurrence probability | |
| *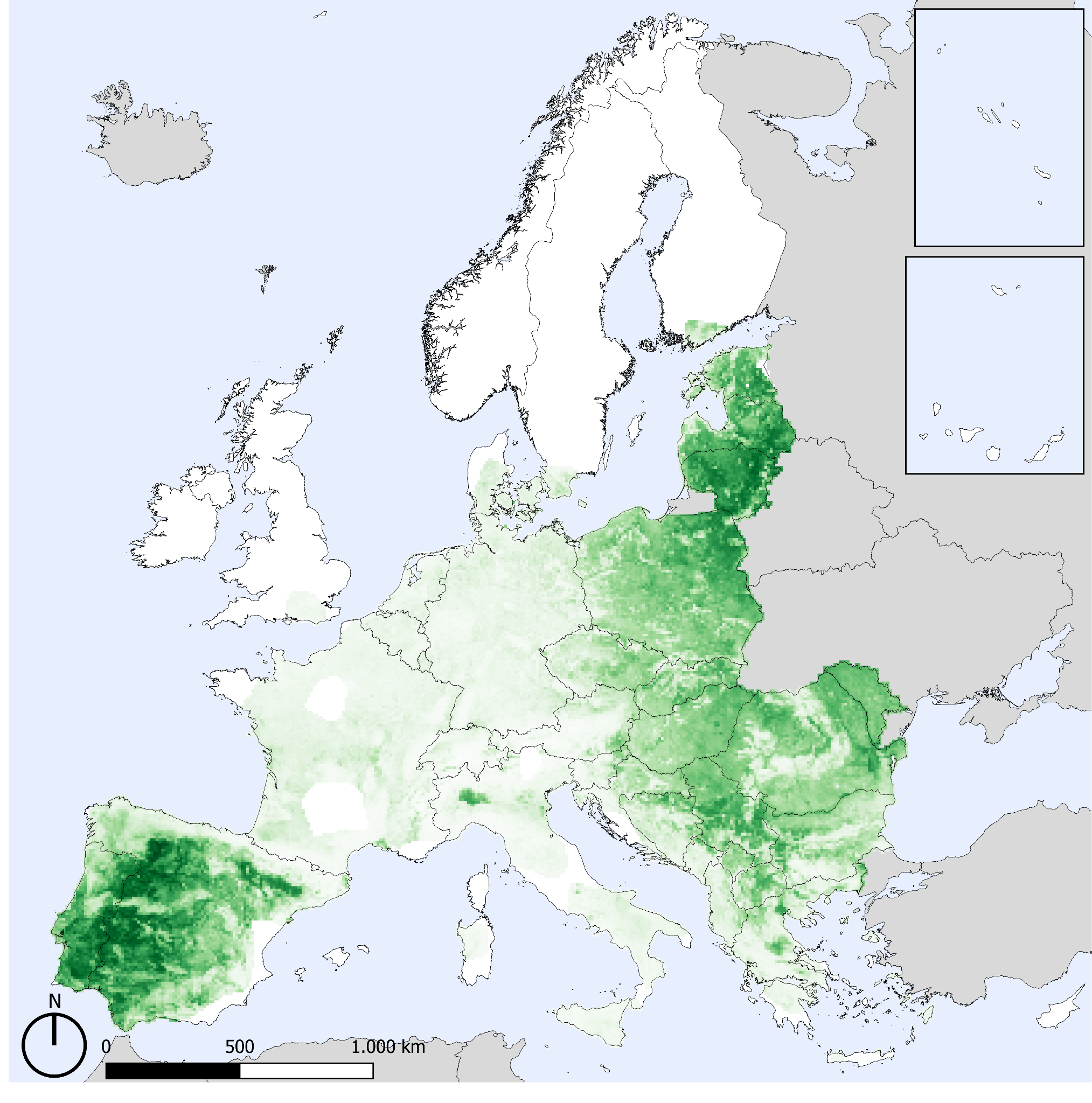* | *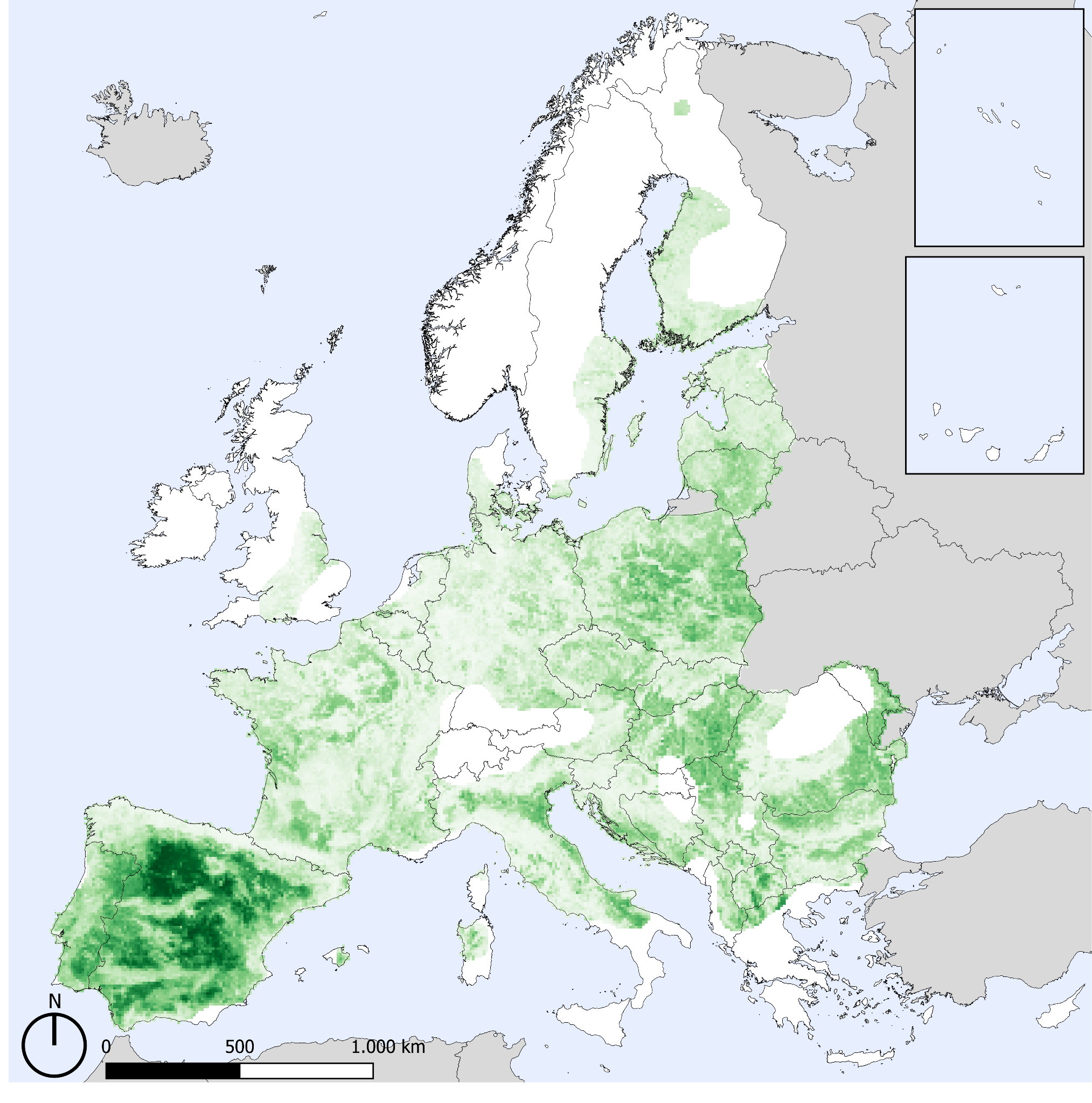* |
| *Ciconia ciconia* | *Circus pygargus* |
| *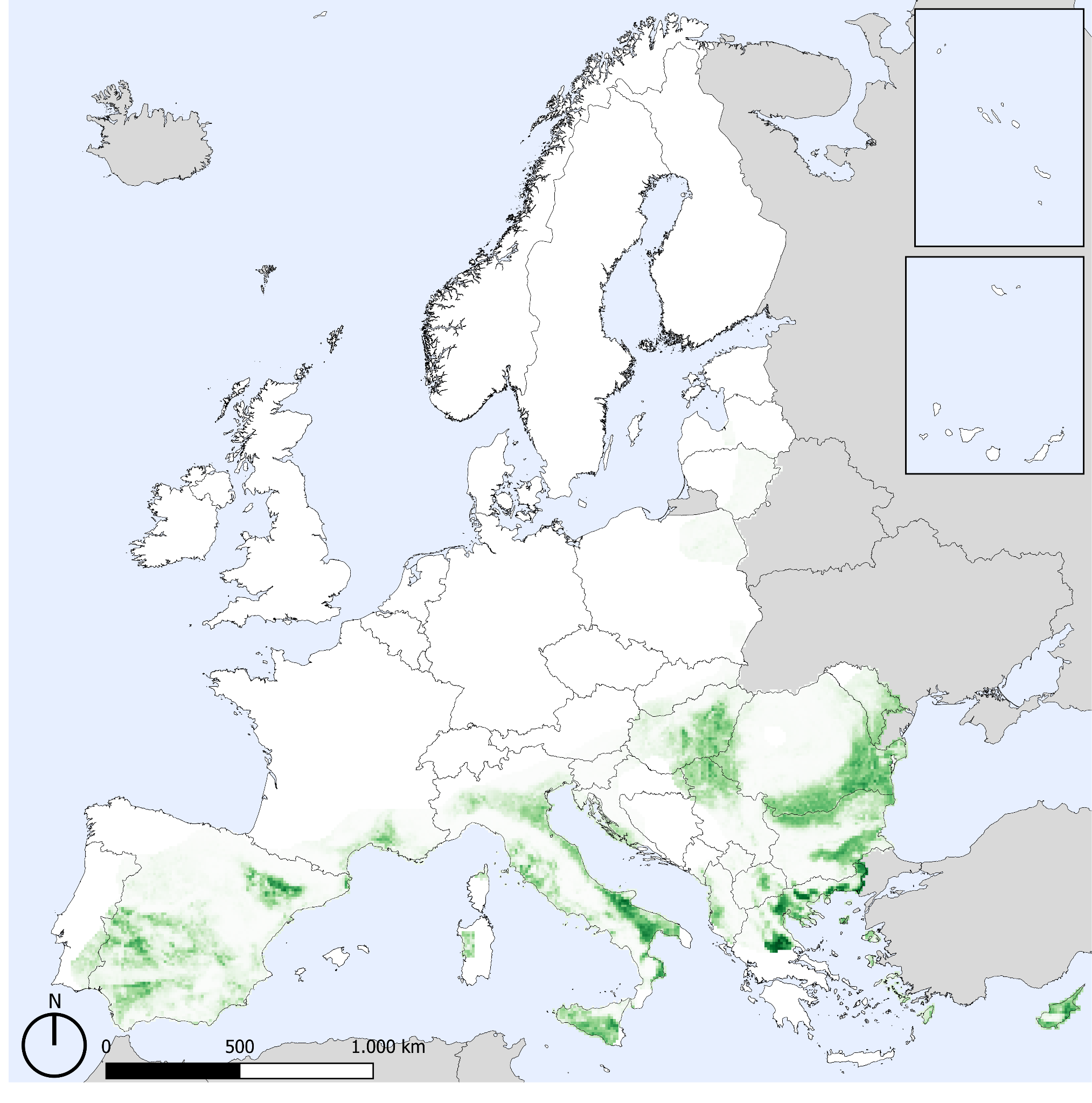* | *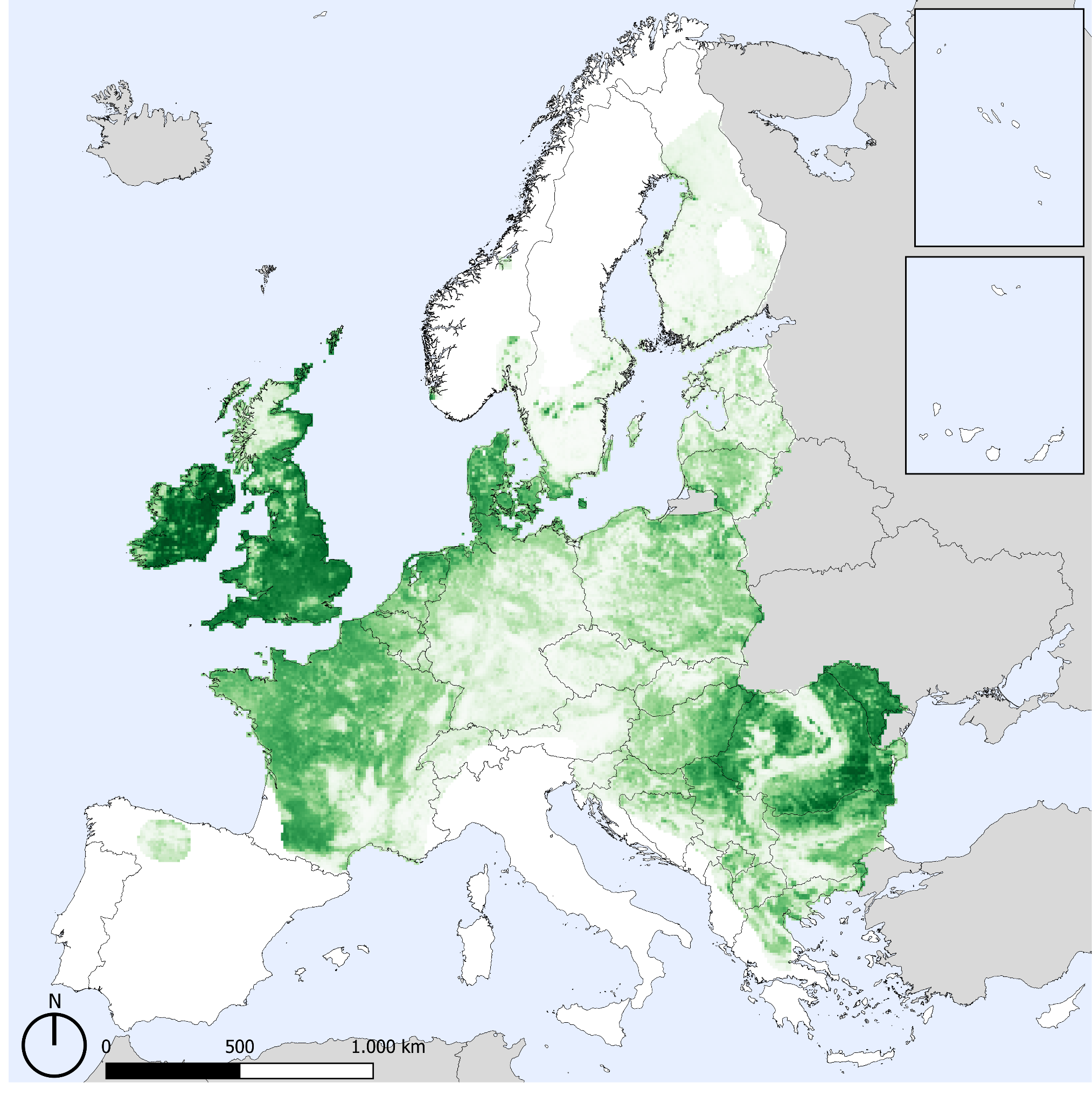* |
| *Coracias garrulus* | *Corvus frugilegus* |
| 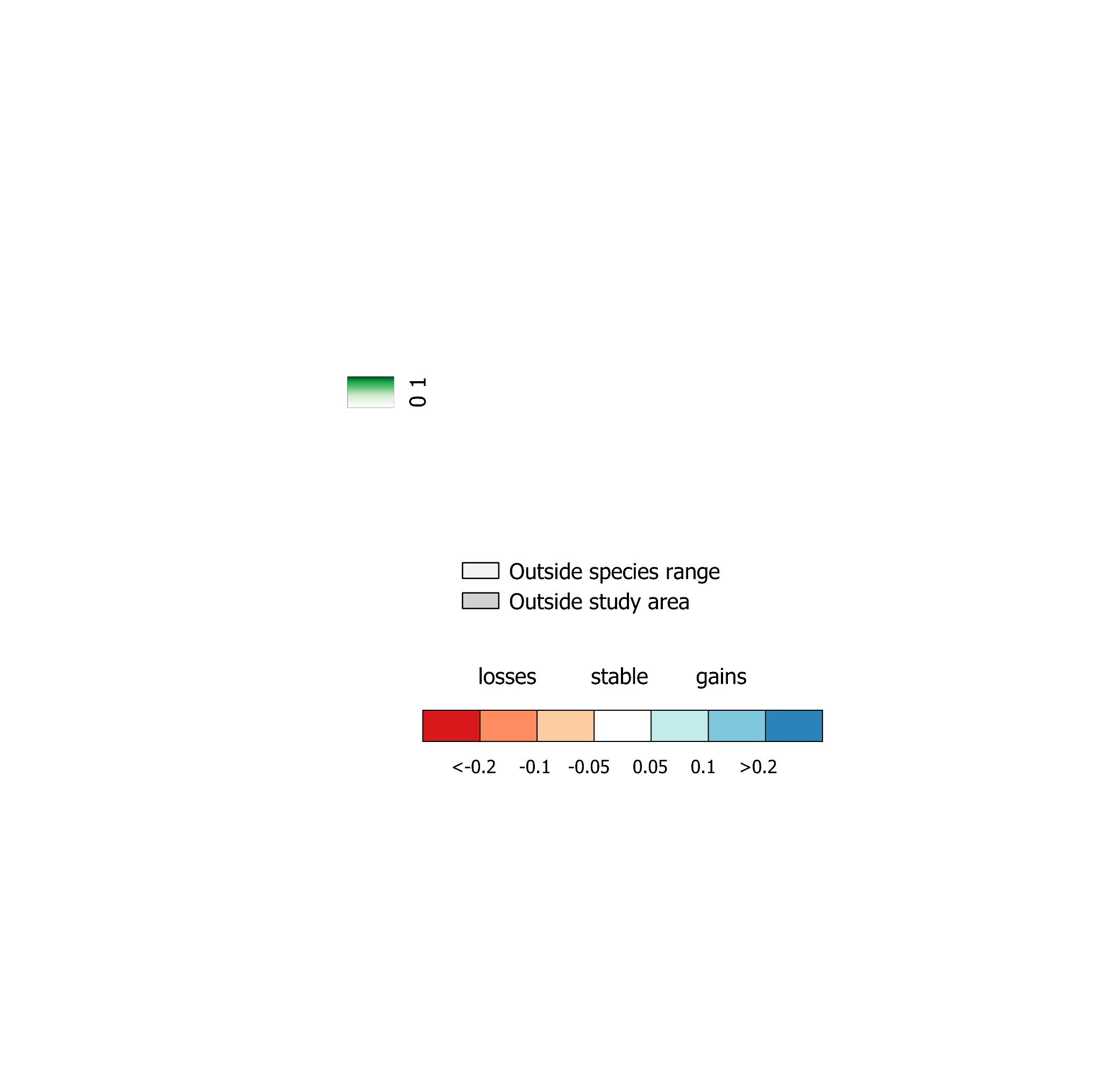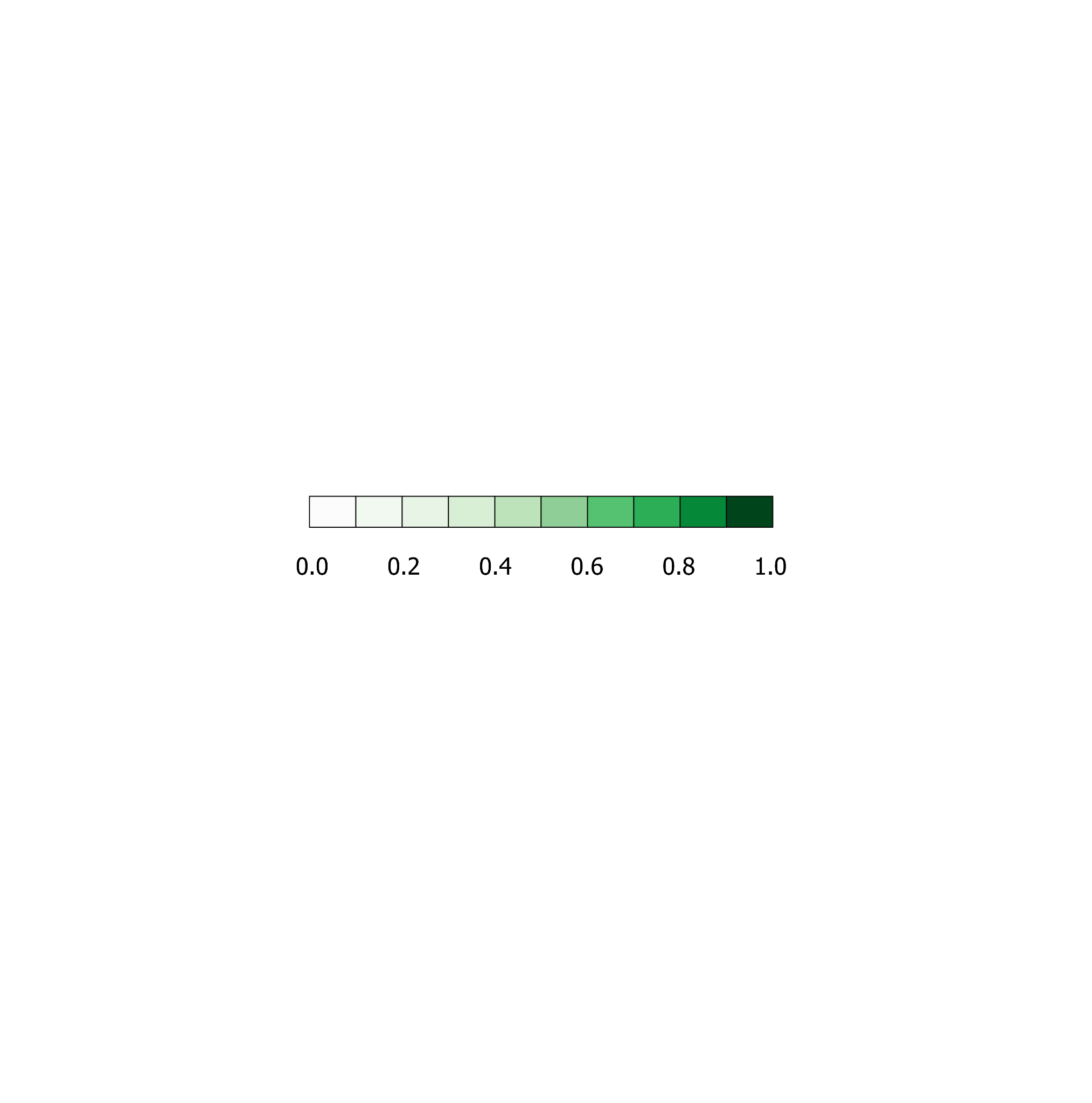  Occurrence probability | |
| *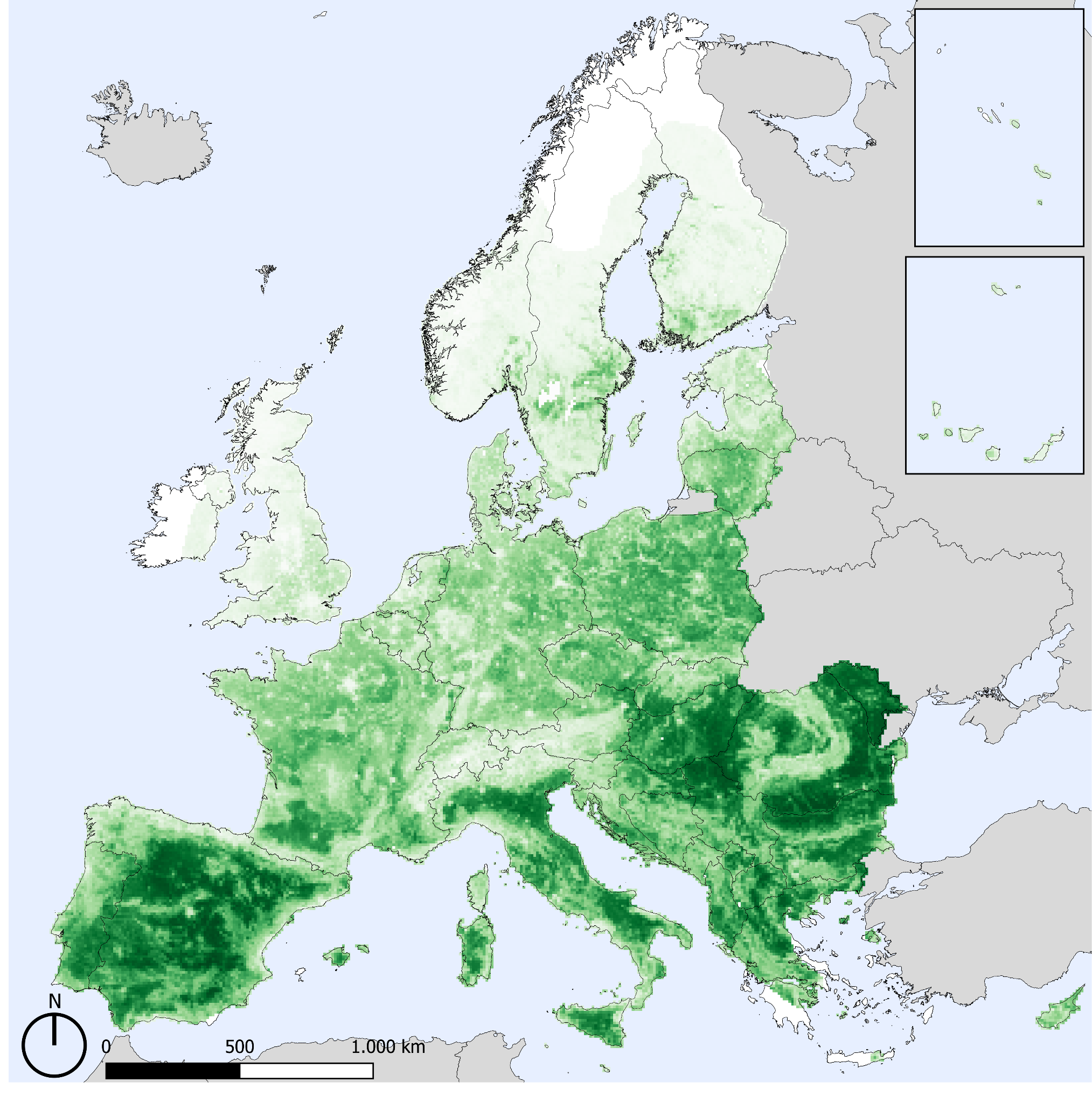* | *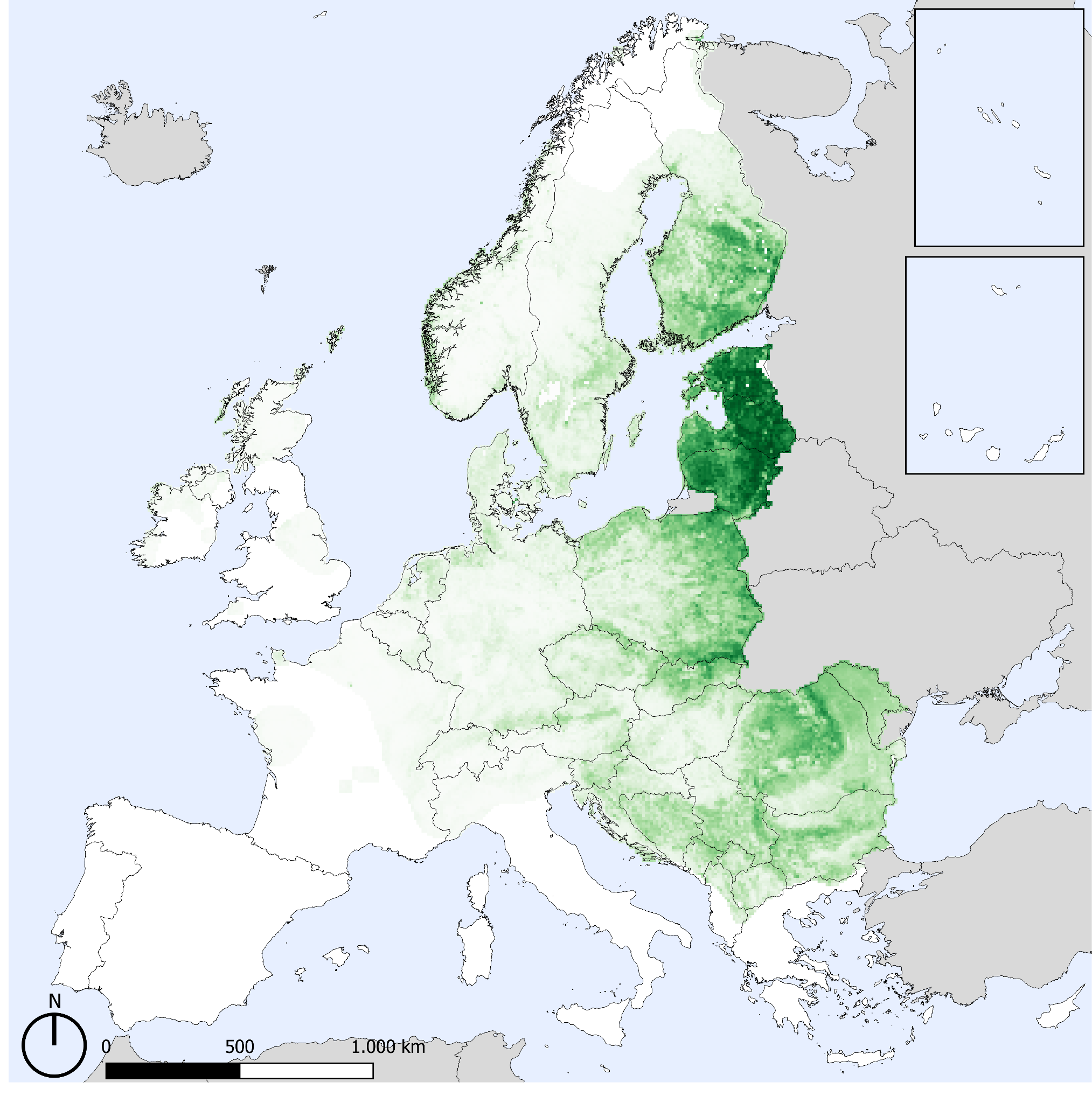* |
| *Coturnix coturnix* | *Crex crex* |
| *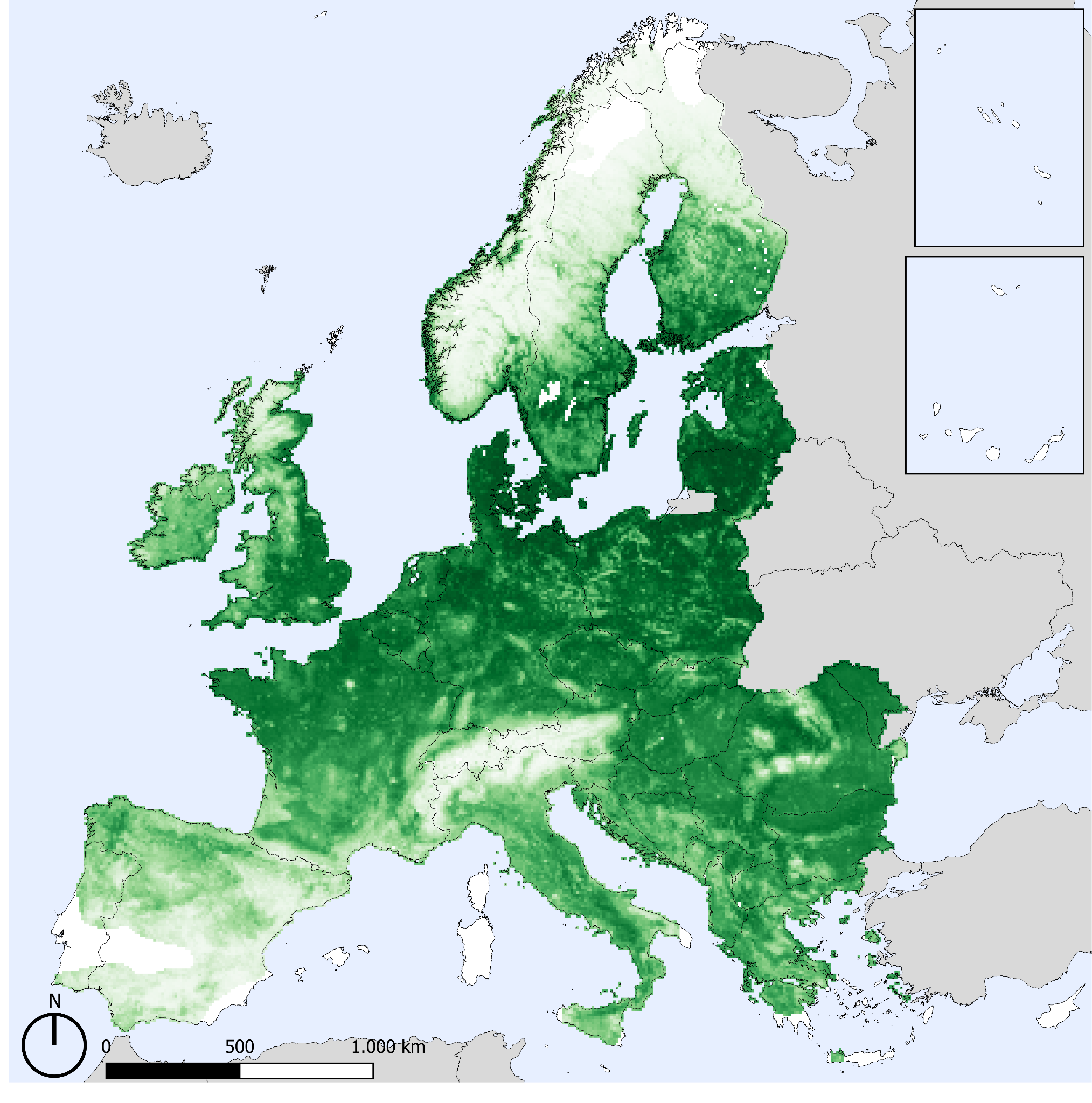* | *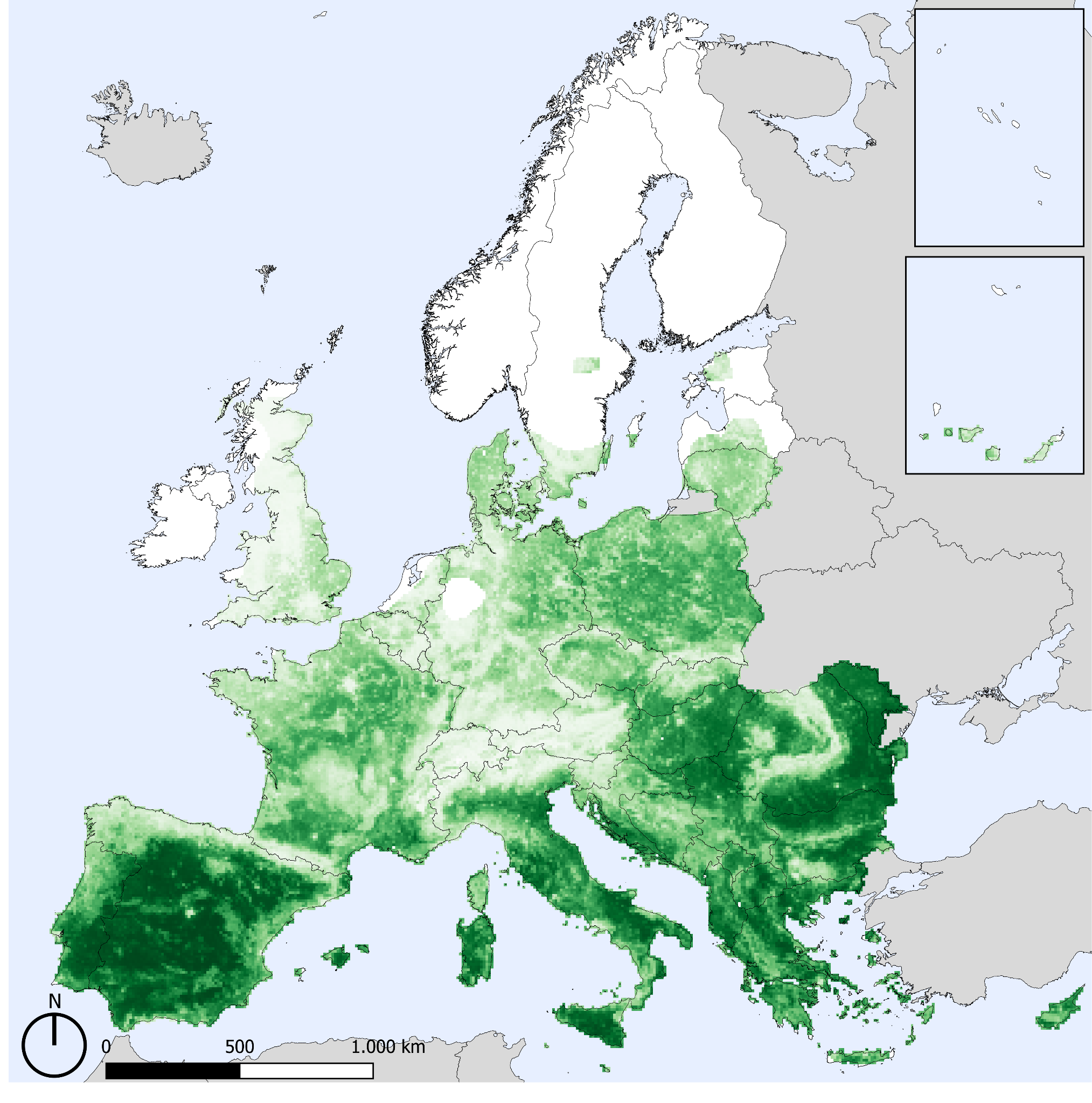* |
| *Curruca communis* | *Emberiza calandra* |
| 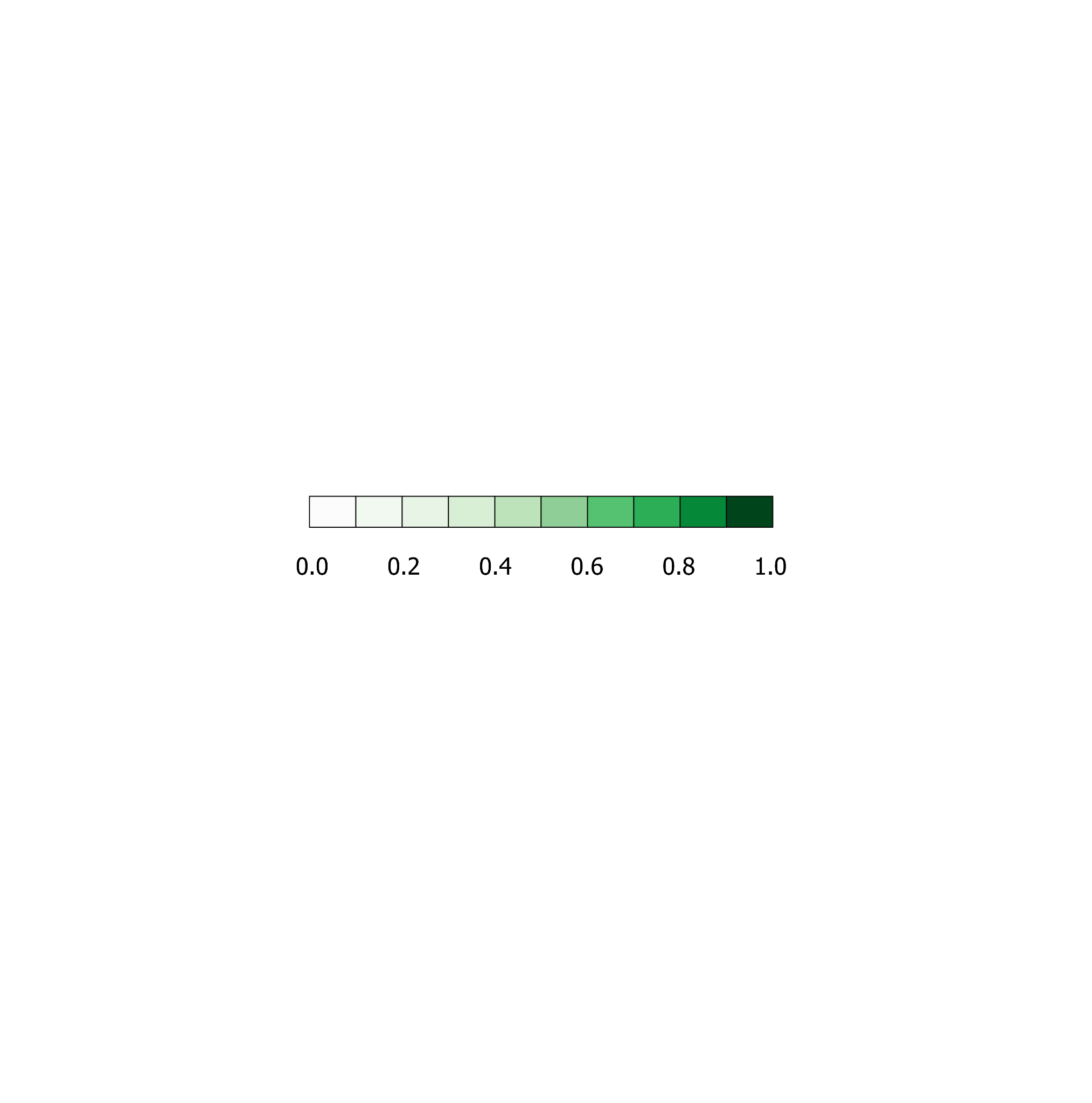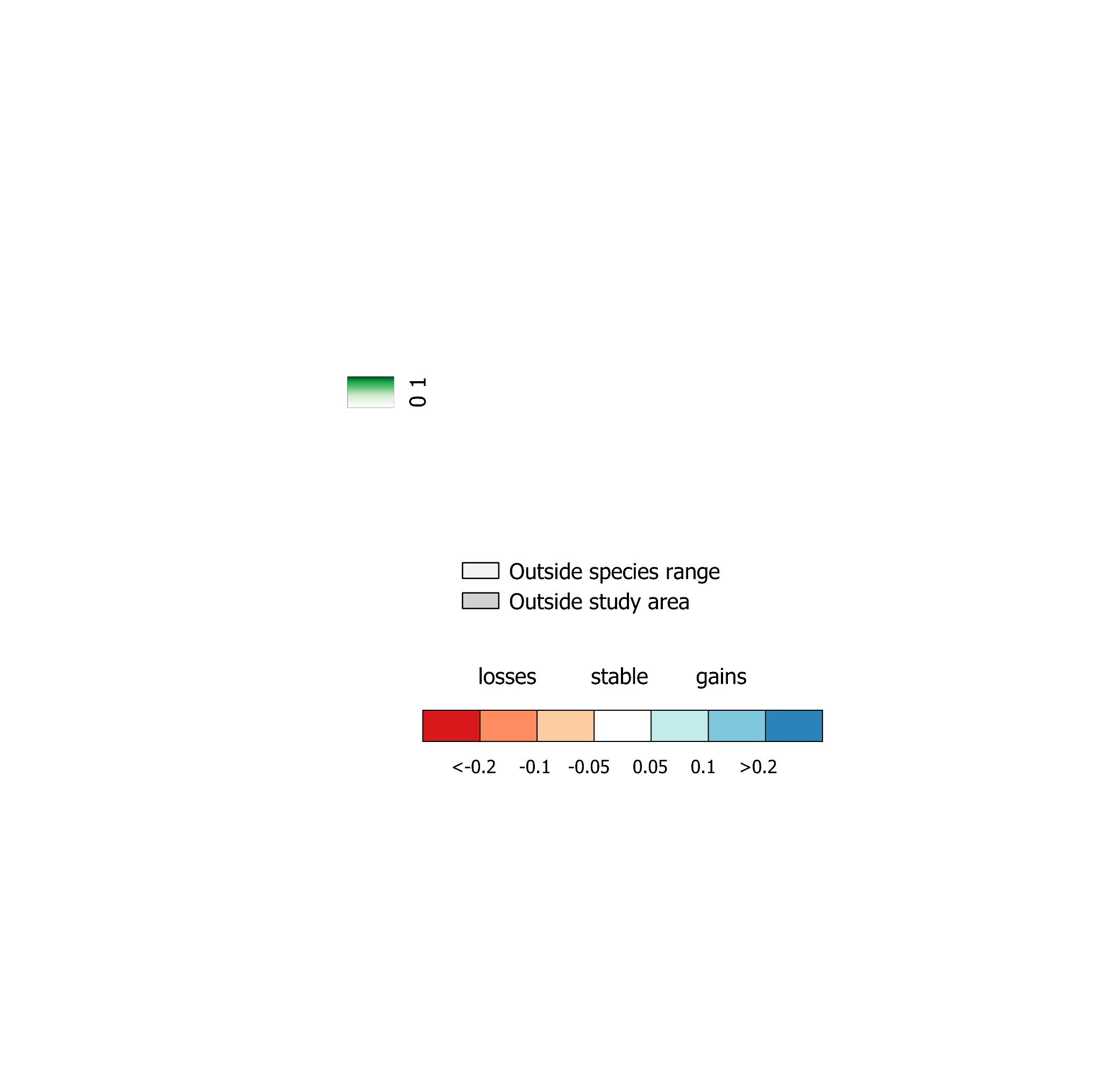  Occurrence probability | |
| *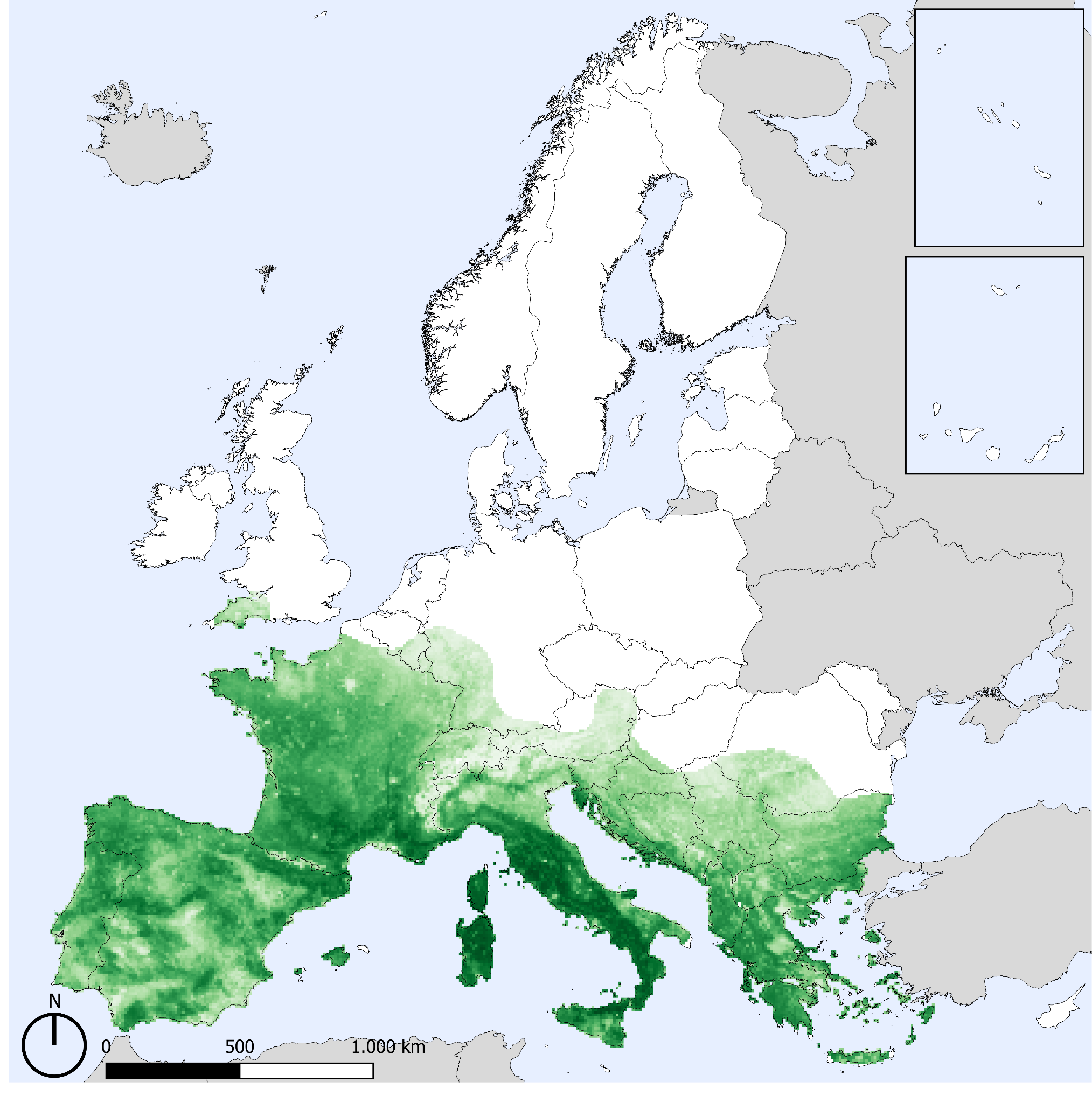* | *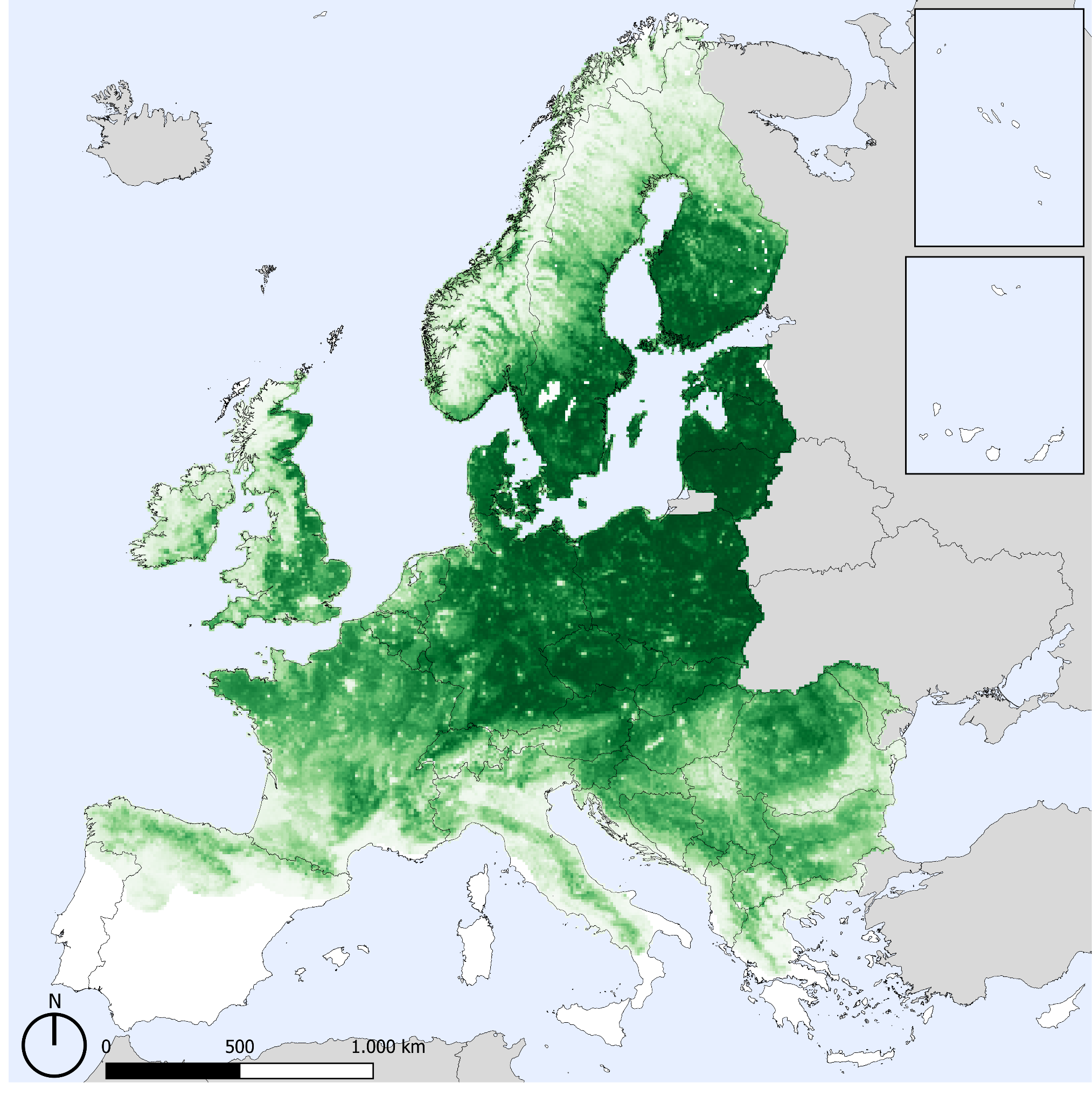* |
| *Emberiza cirlus* | *Emberiza citrinella* |
| *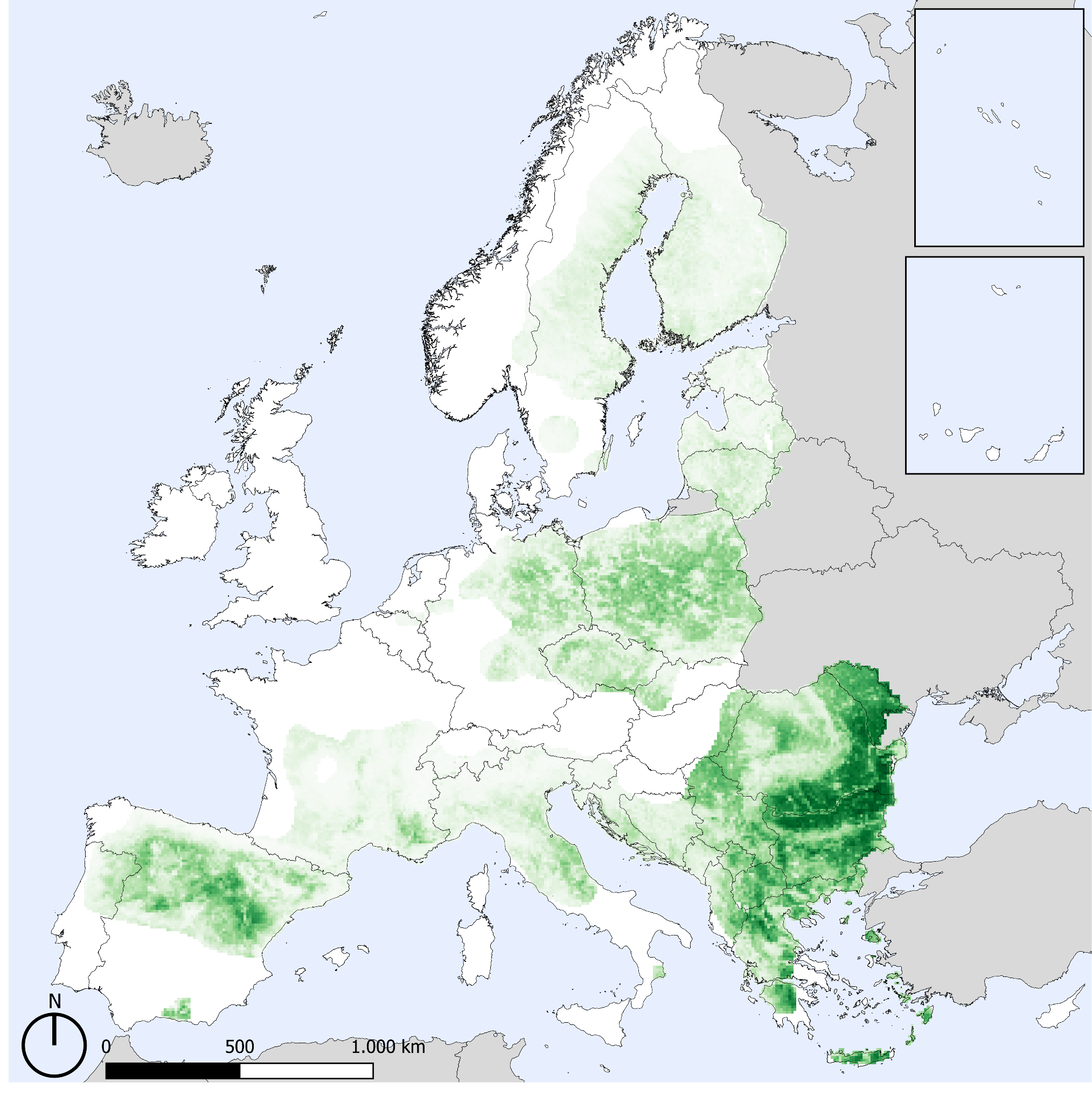* | *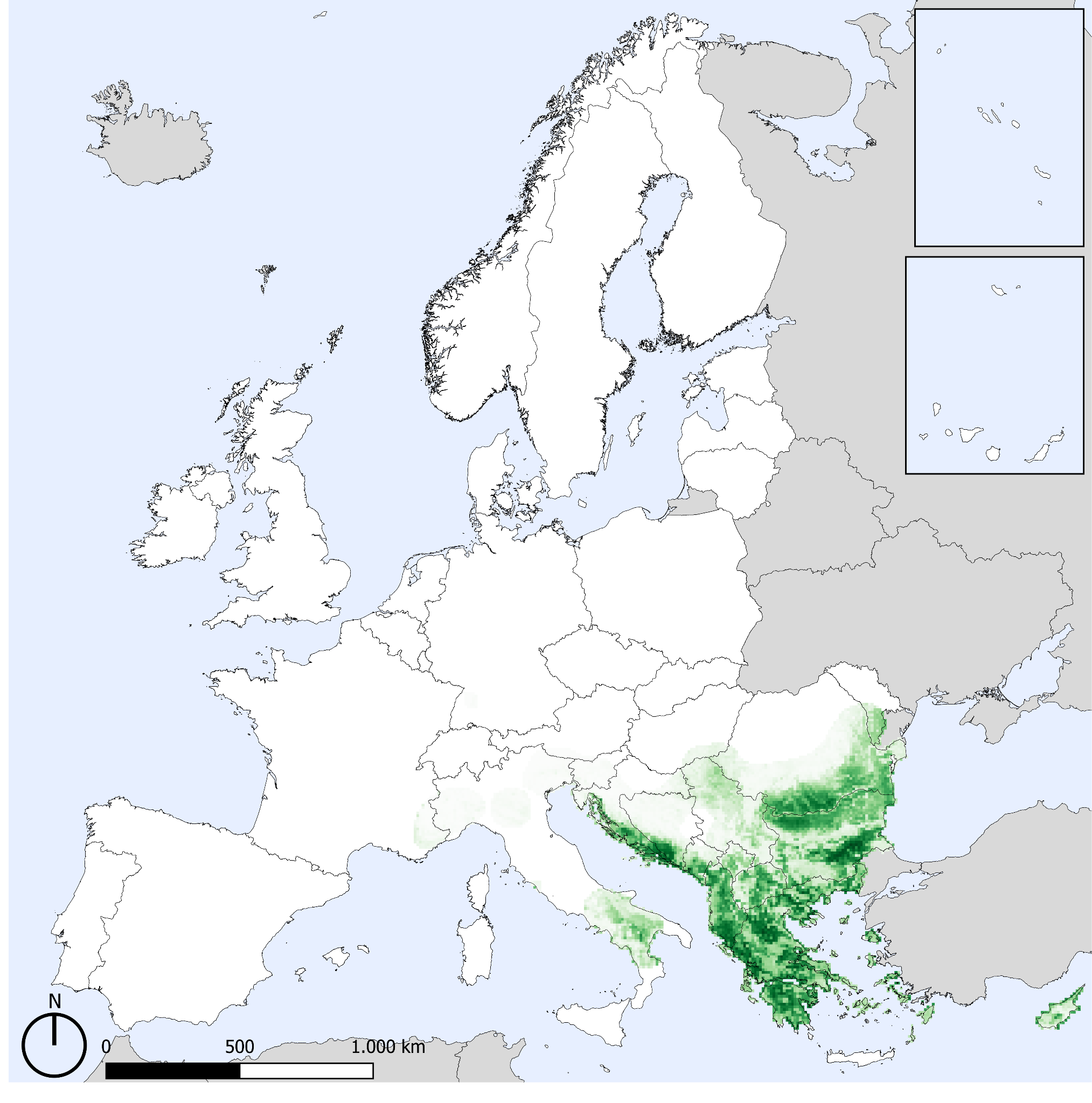* |
| *Emberiza hortulana* | *Emberiza melanocephala* |
| 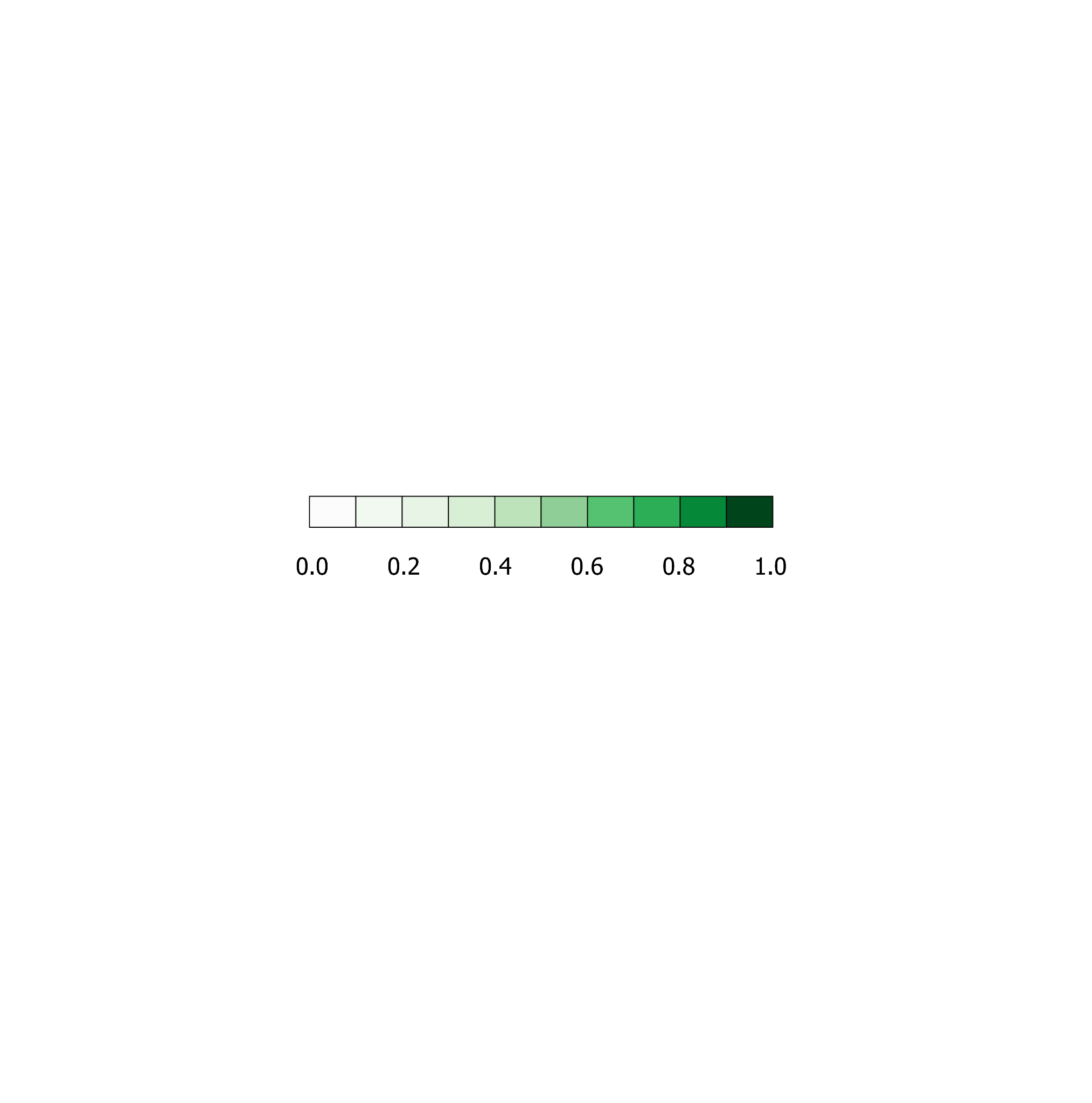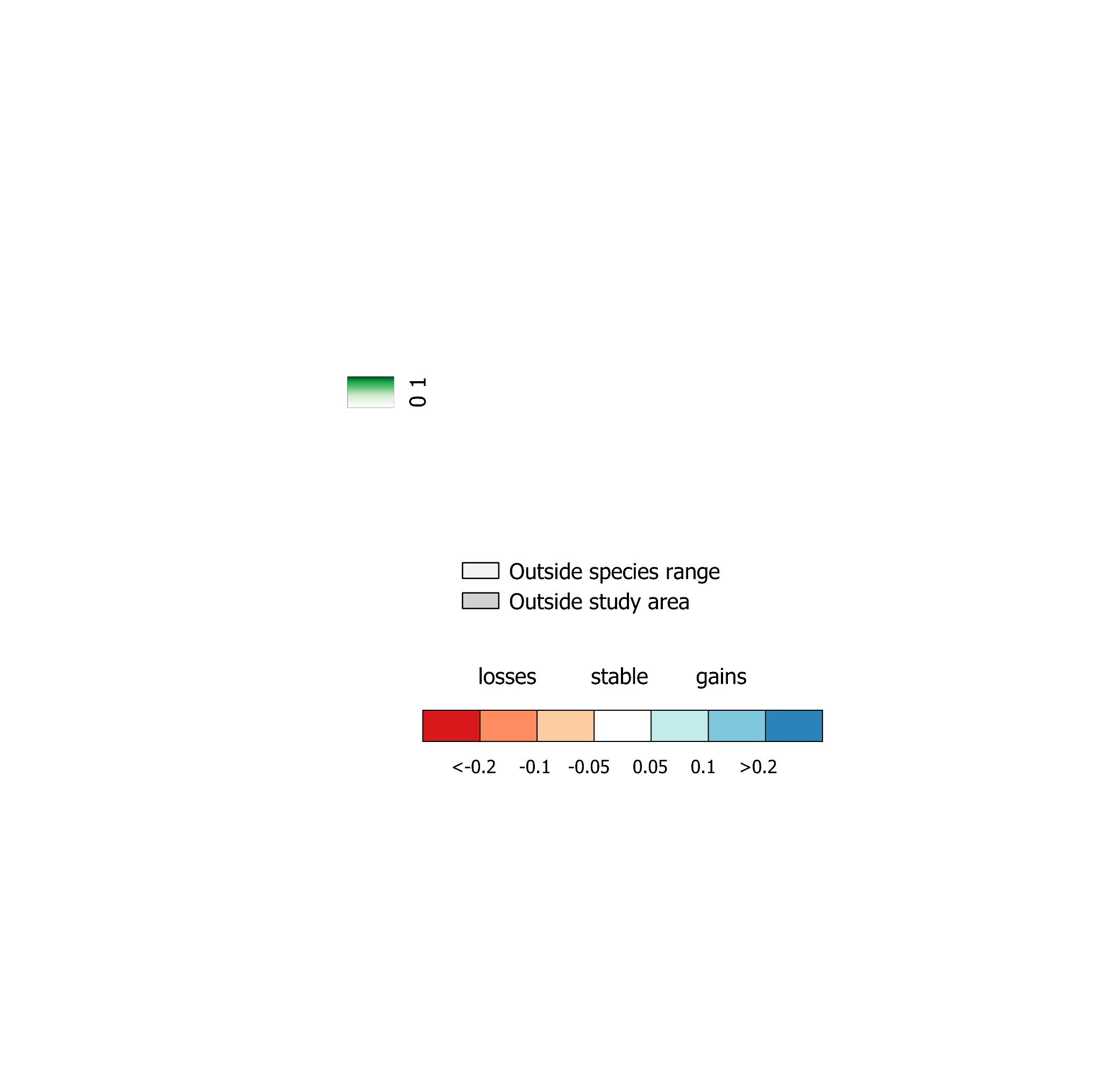  Occurrence probability | |
| *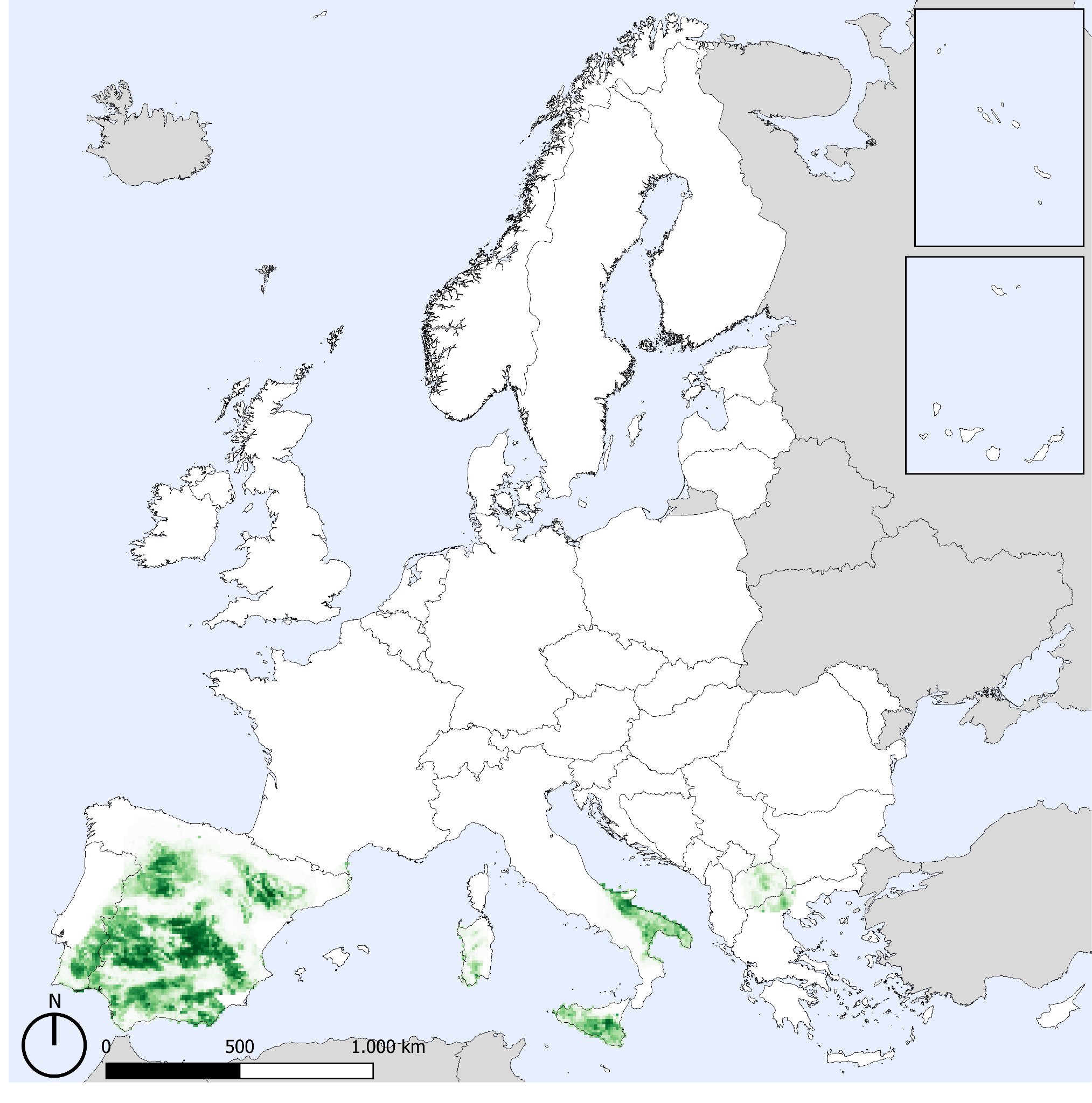* | *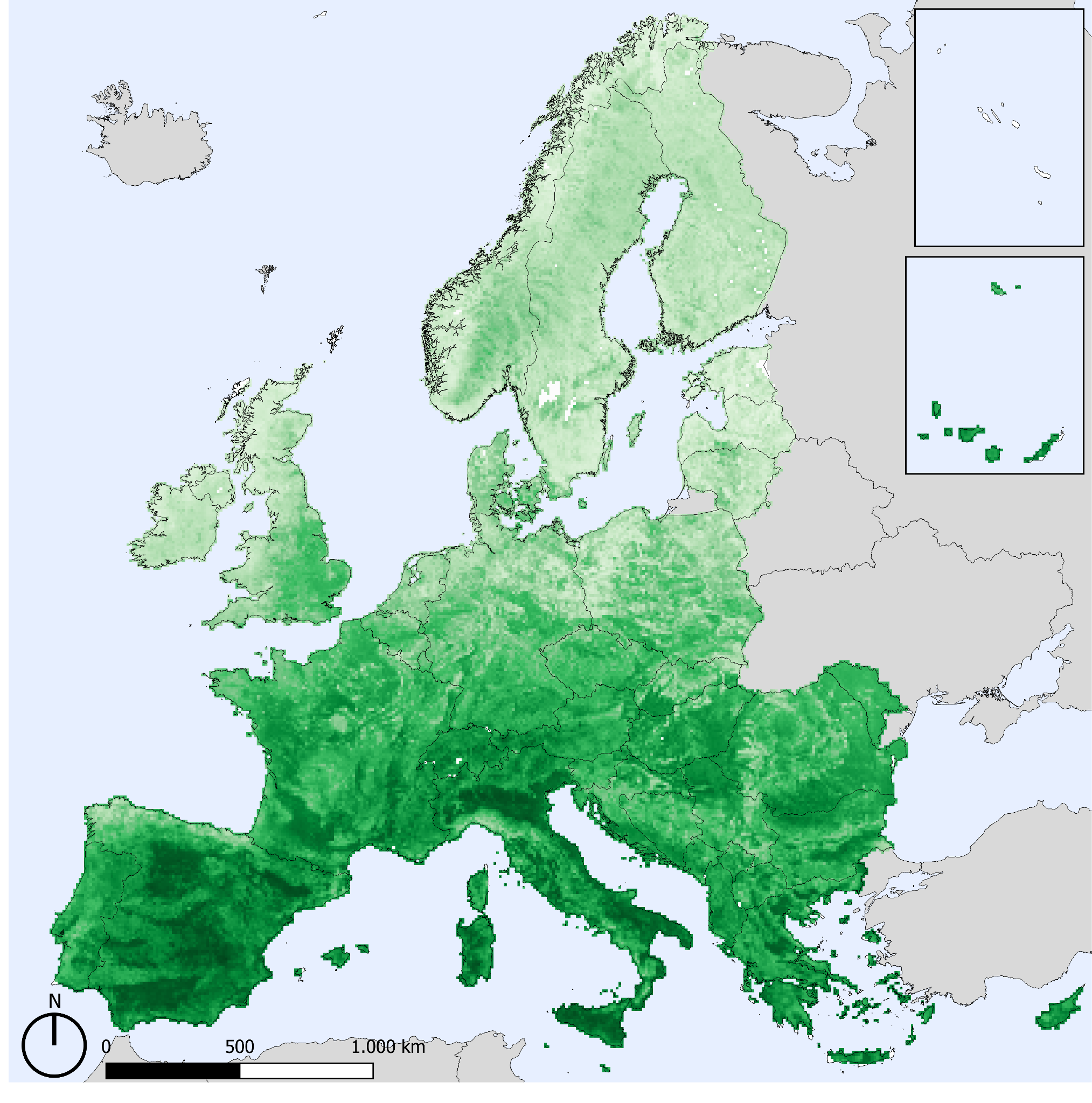* |
| *Falco naumanni* | *Falco tinnunculus* |
| *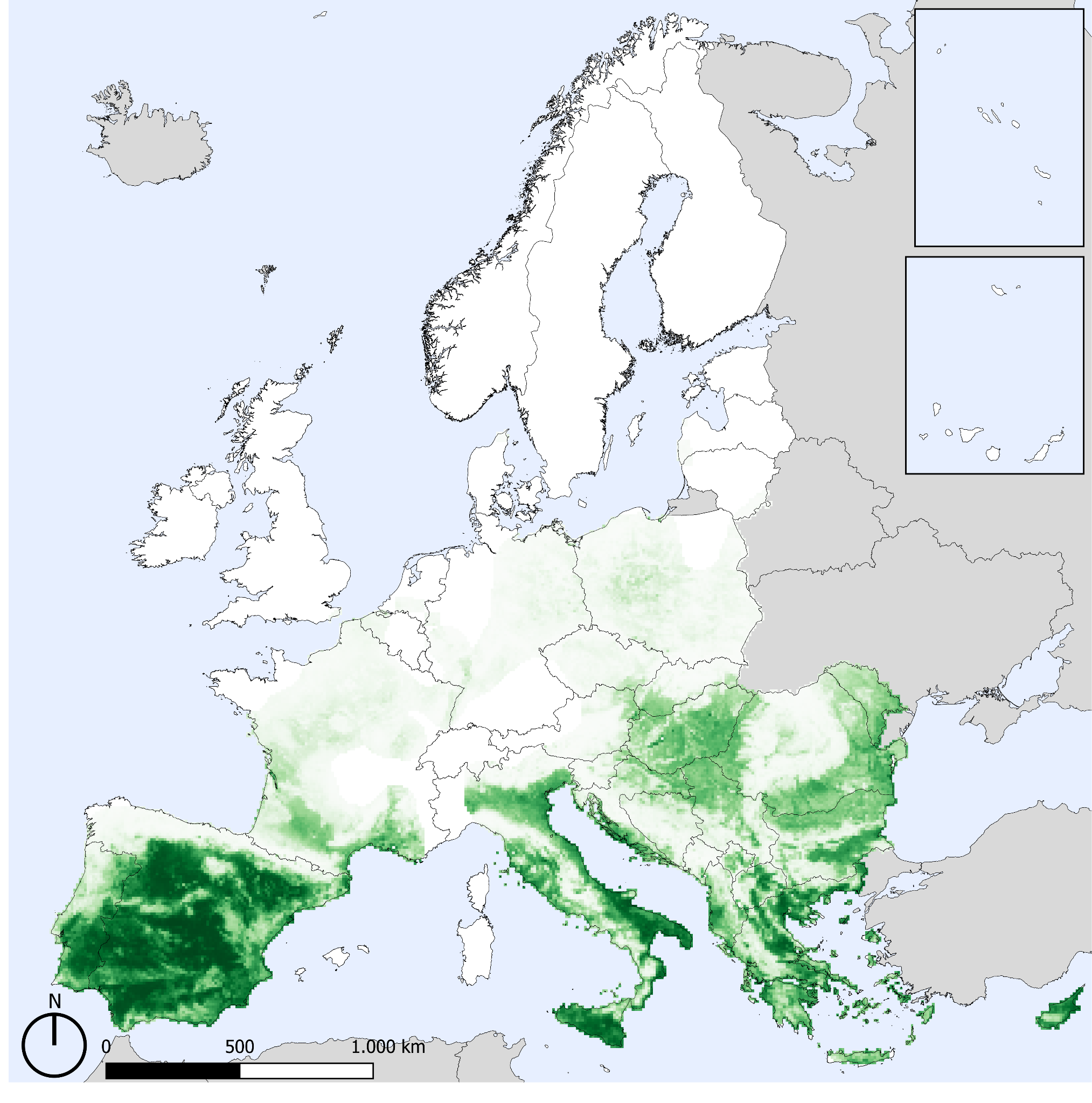* | *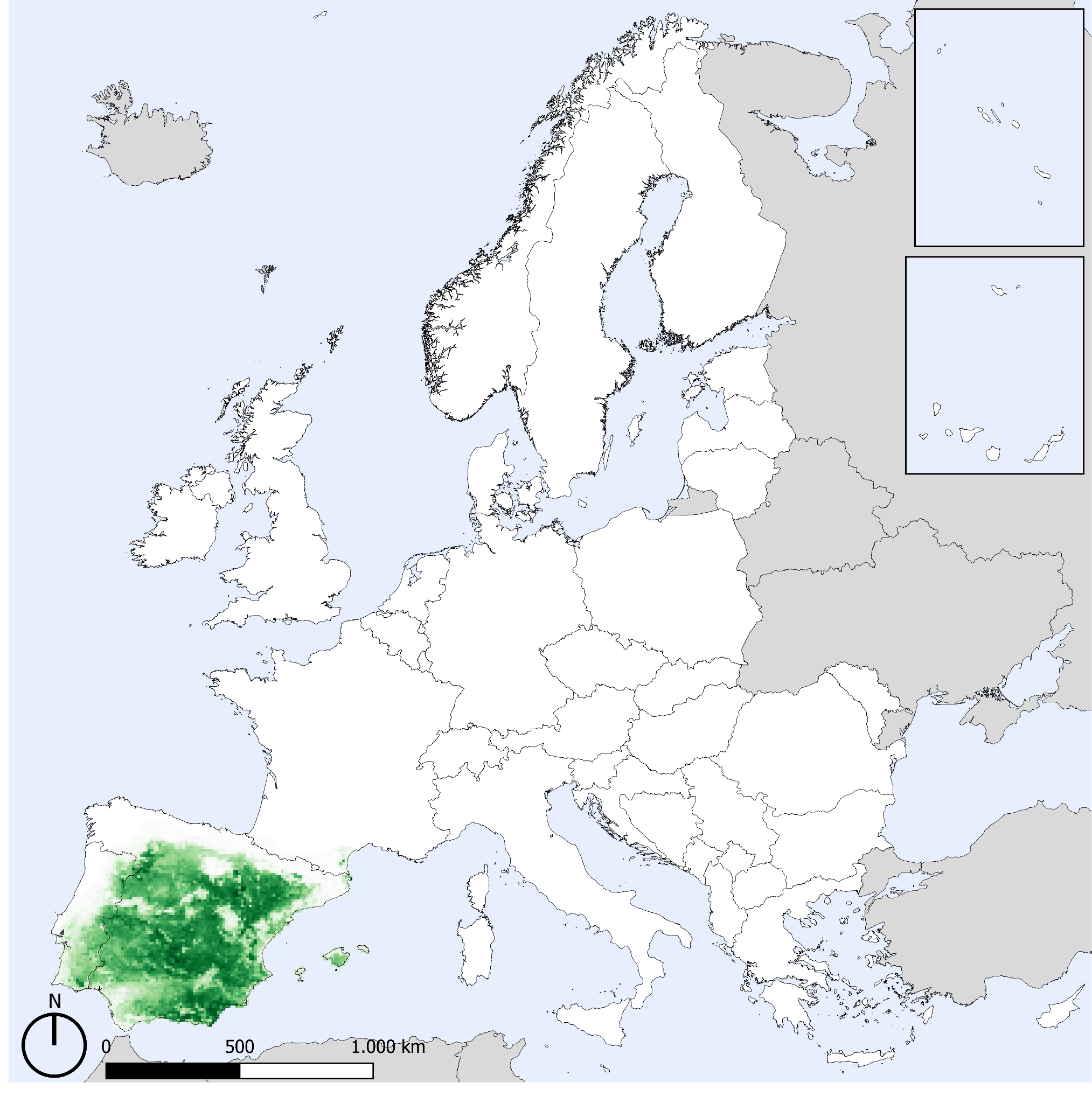* |
| *Galerida cristata* | *Galerida theklae* |
| 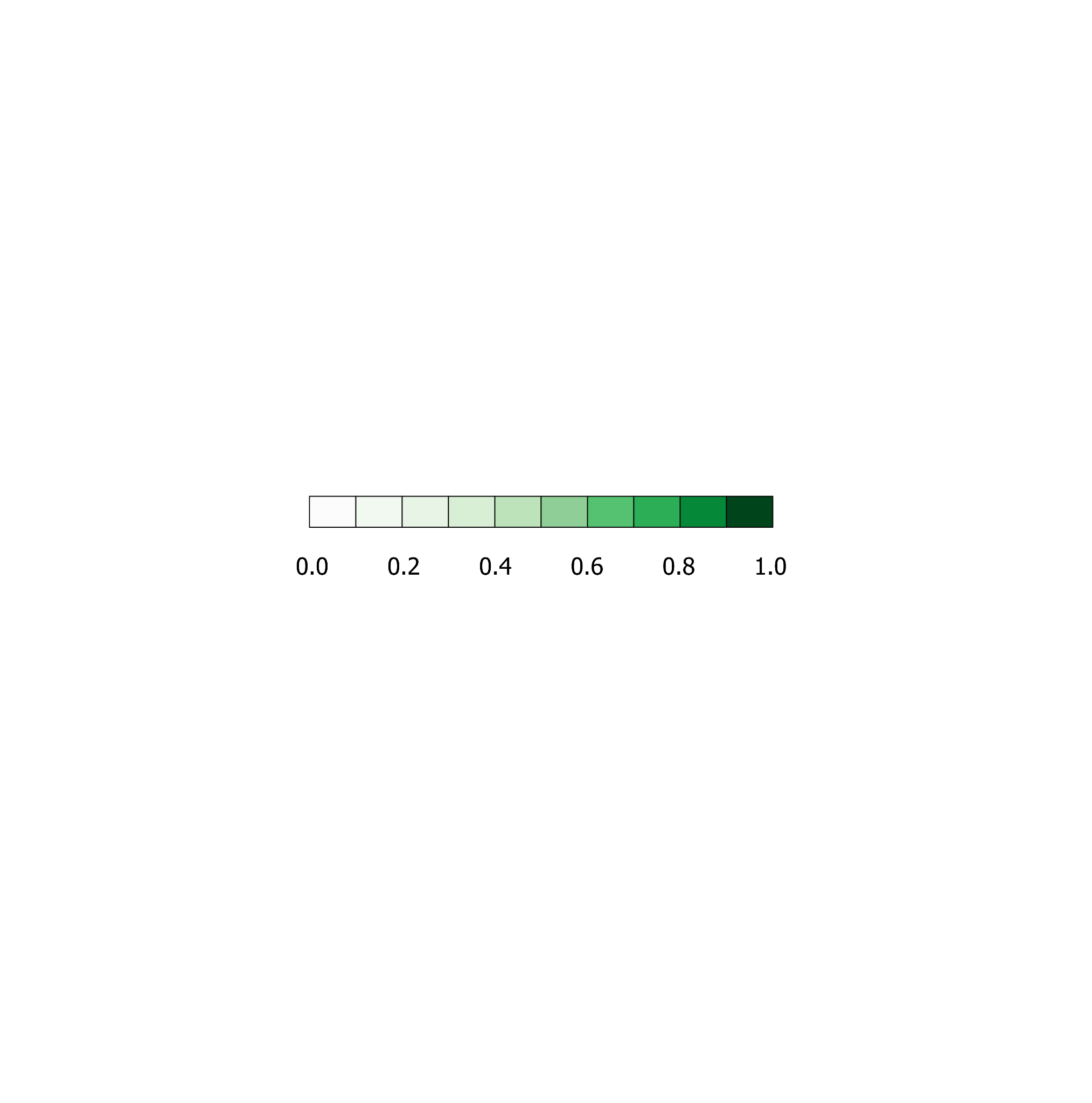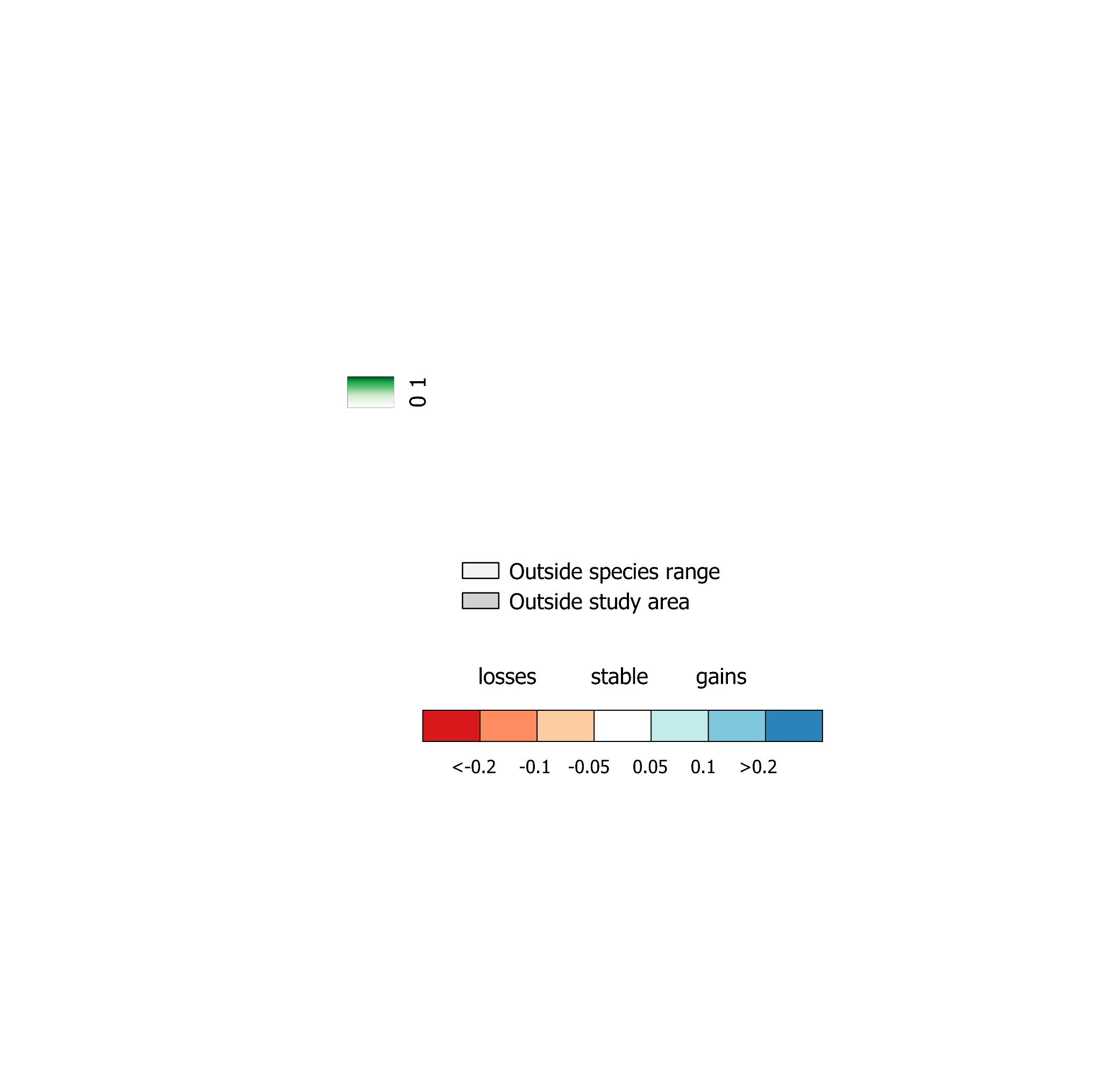  Occurrence probability | |
| *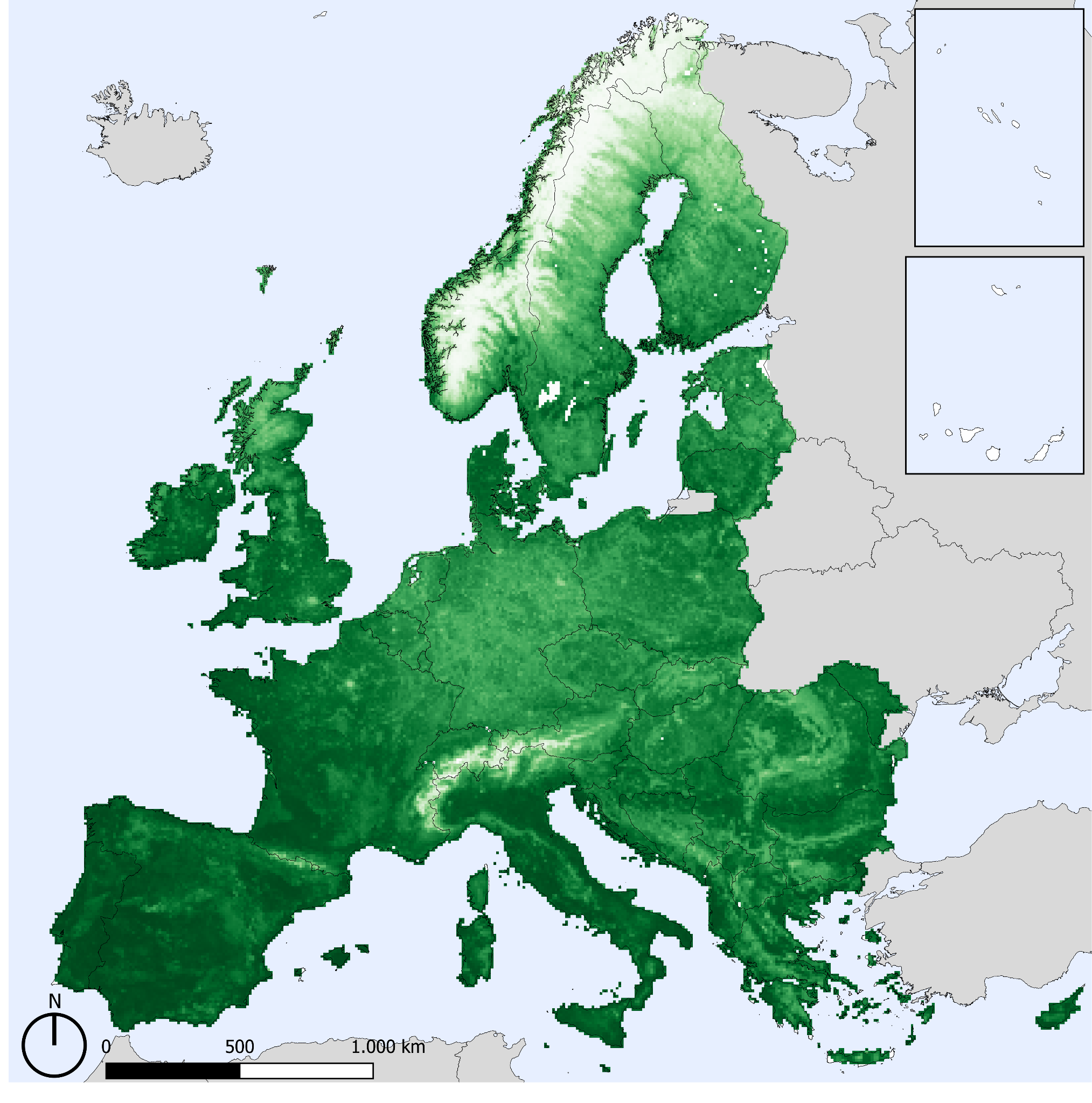* | *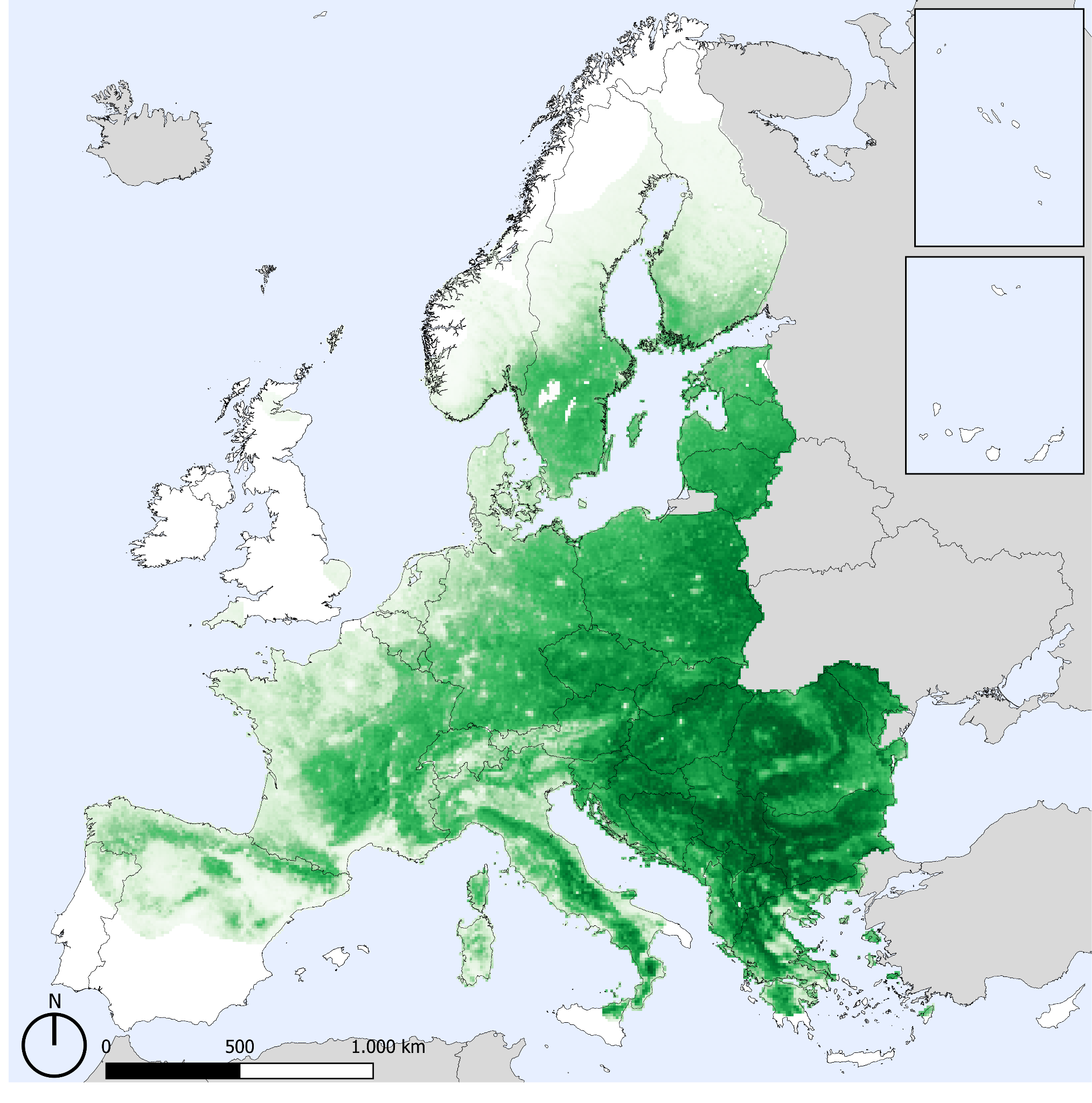* |
| *Hirundo rustica* | *Lanius collurio* |
| *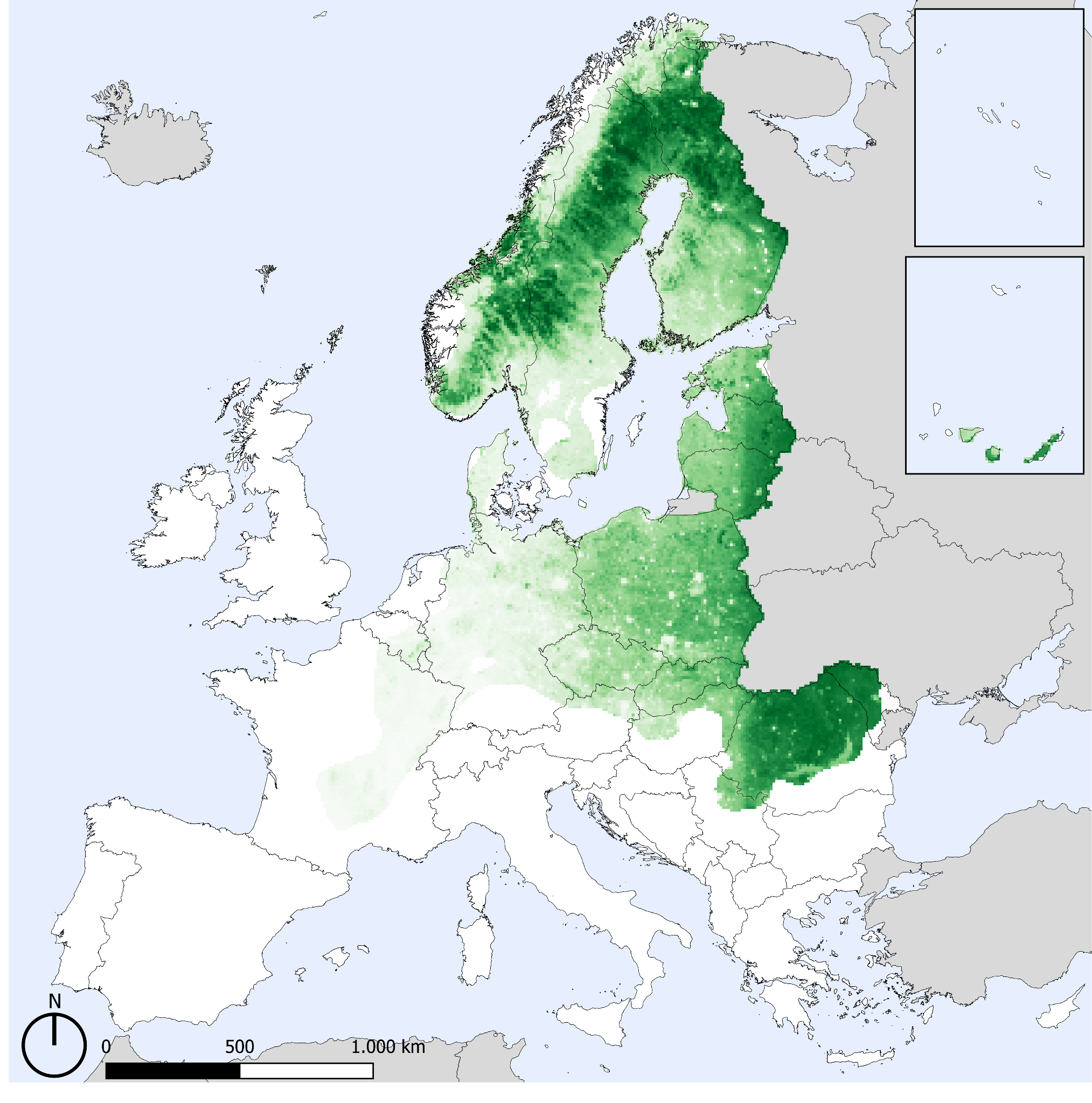* | *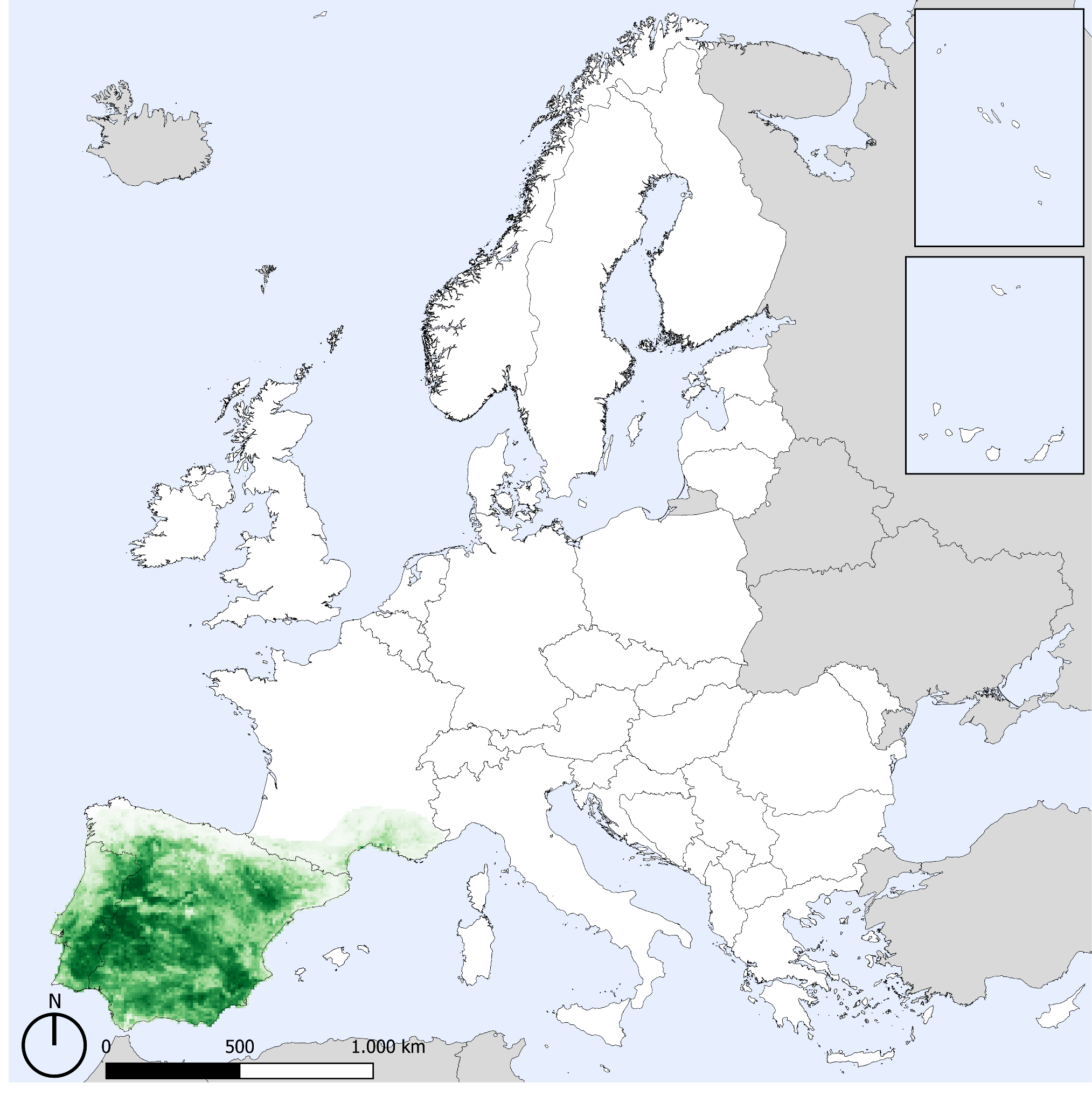* |
| *Lanius excubitor* | *Lanius meridionalis* |
| 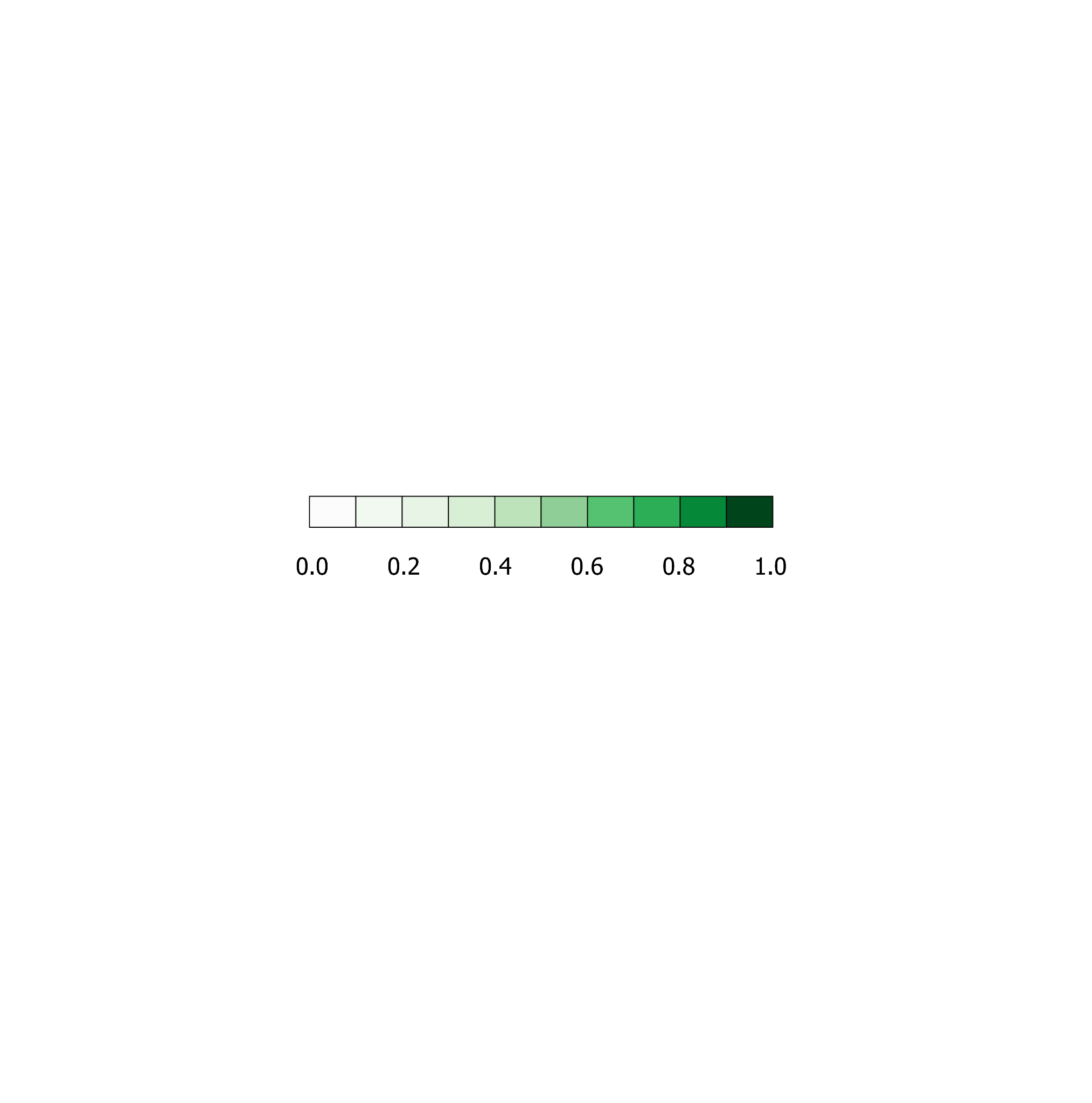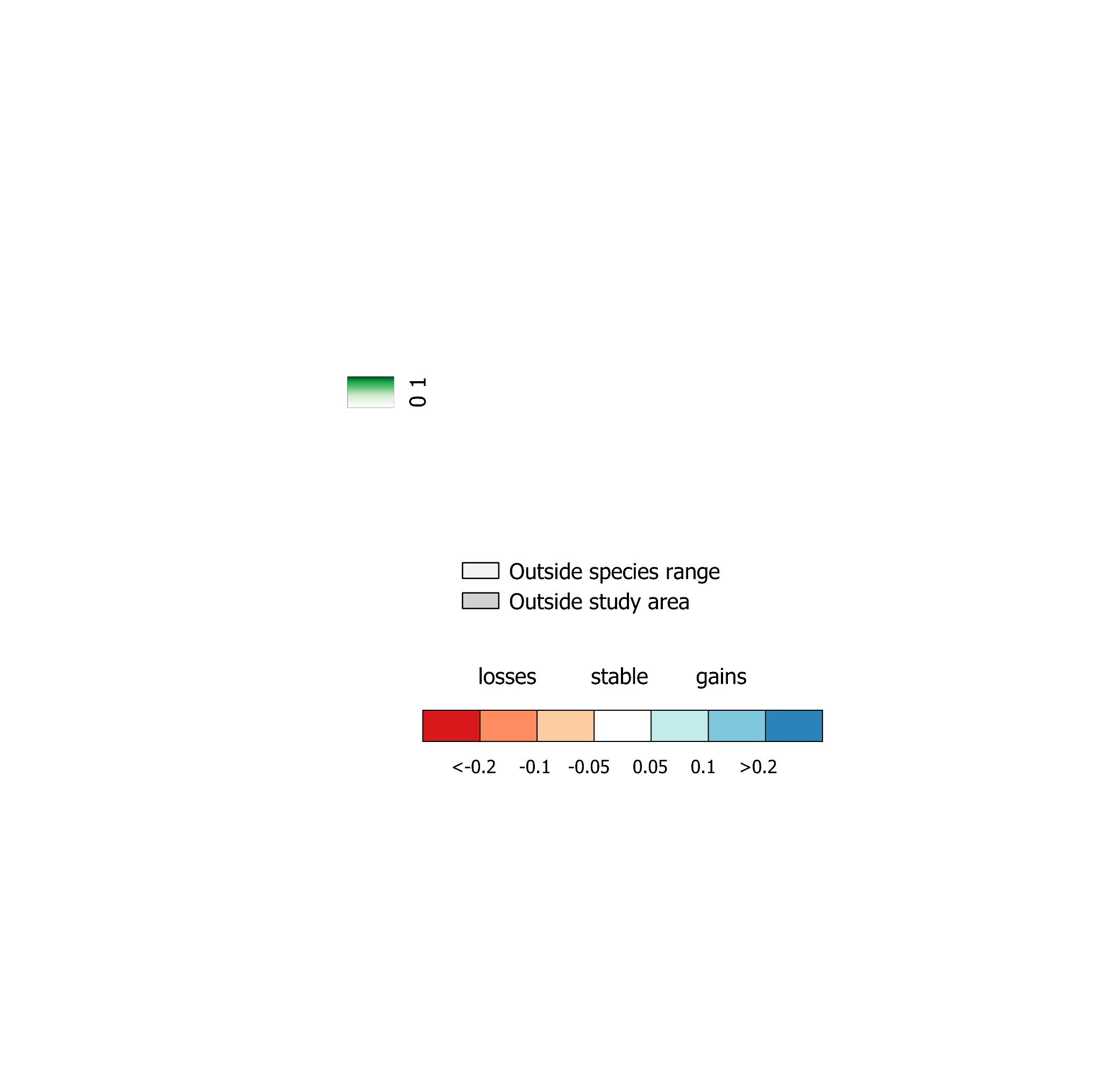  Occurrence probability | |
| *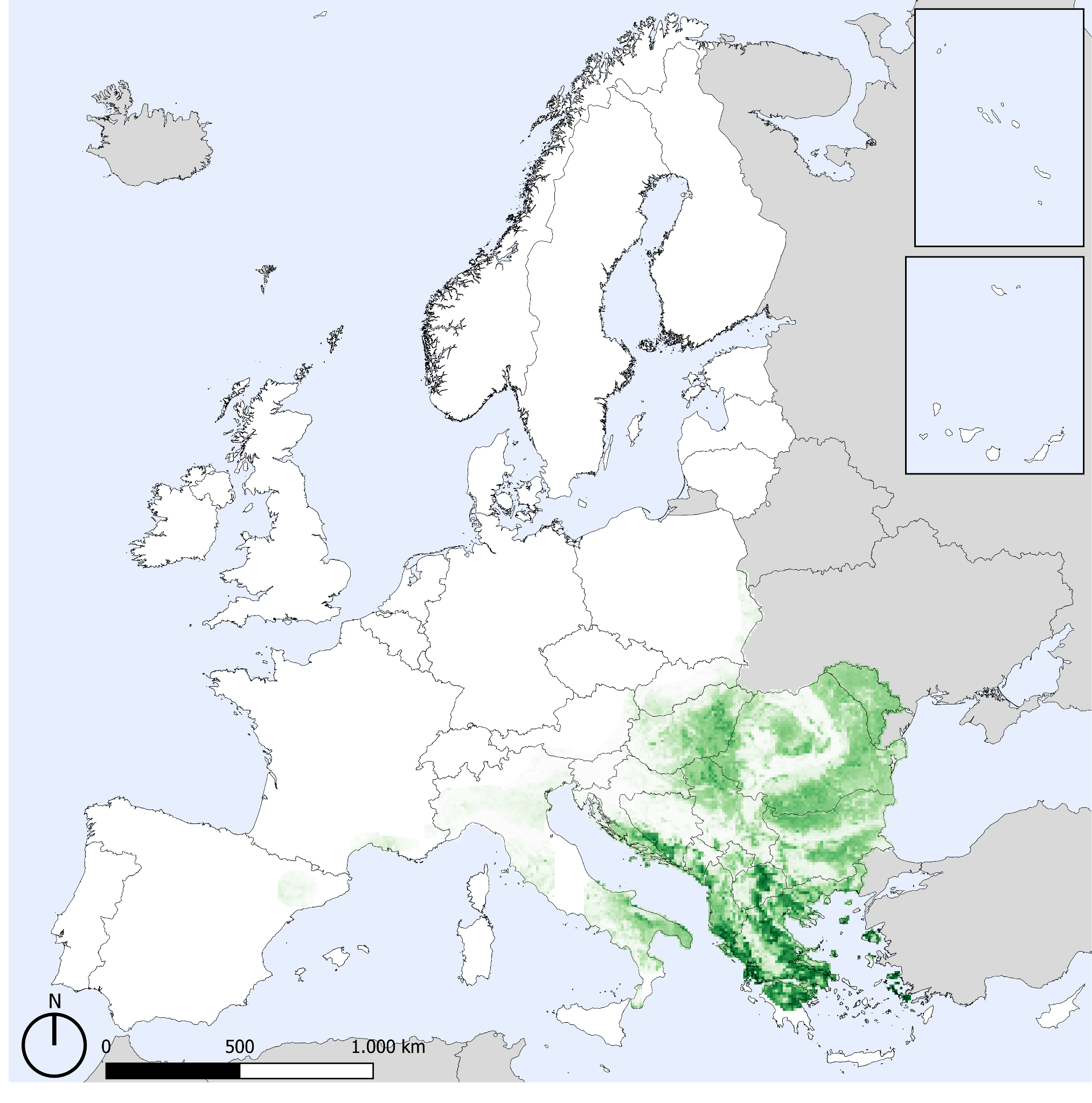* | *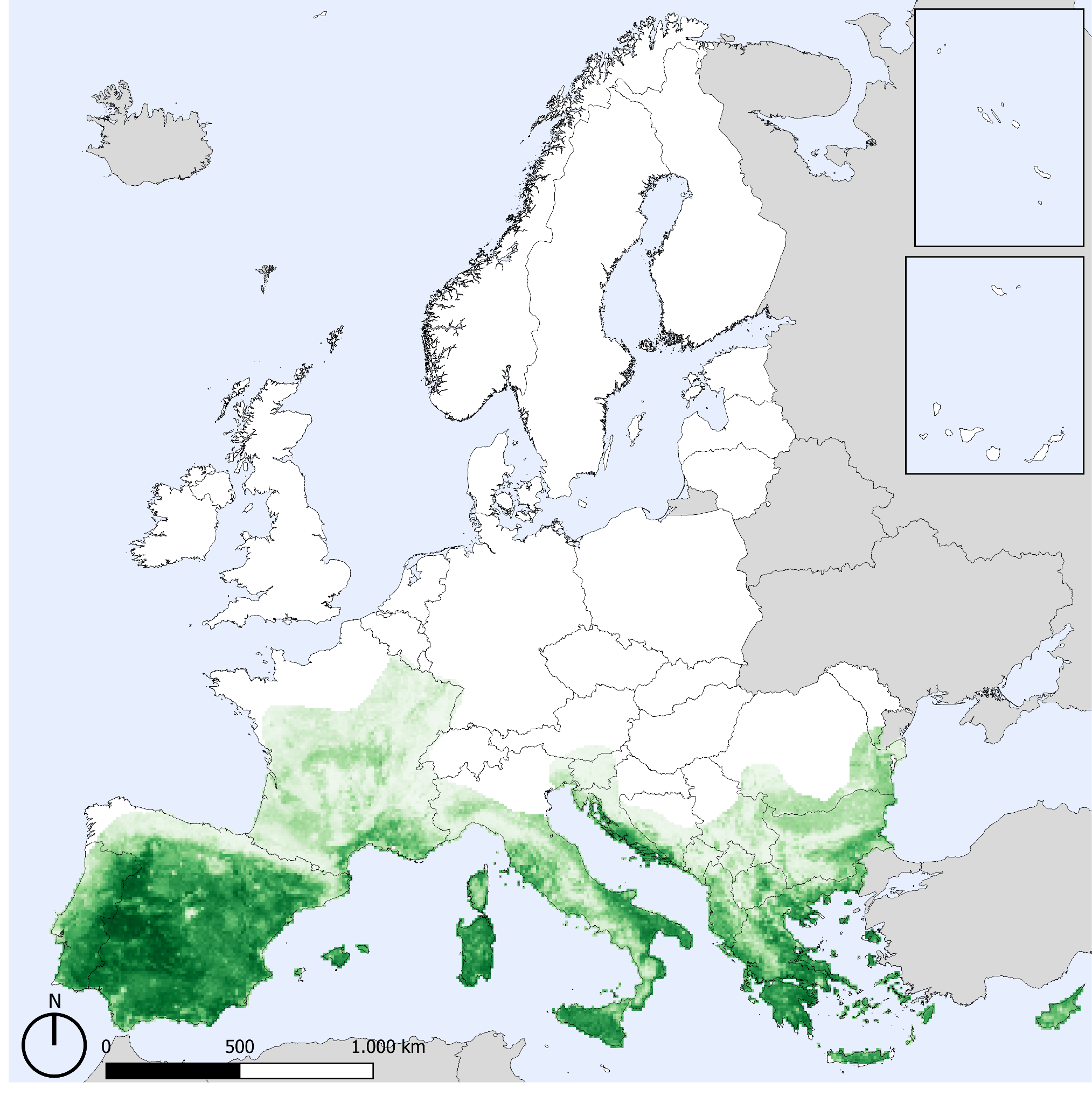* |
| *Lanius minor* | *Lanius senator* |
| *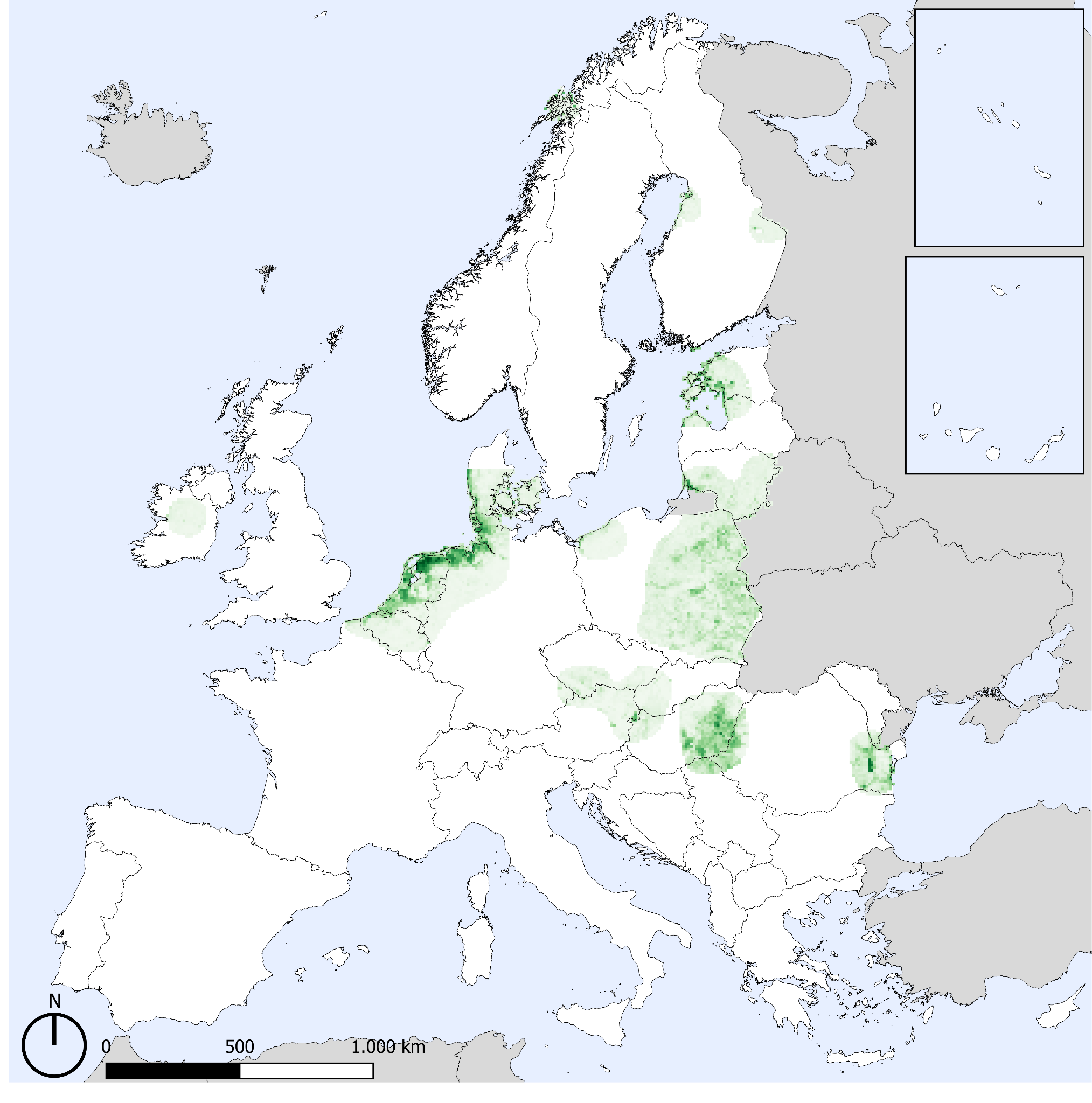* | *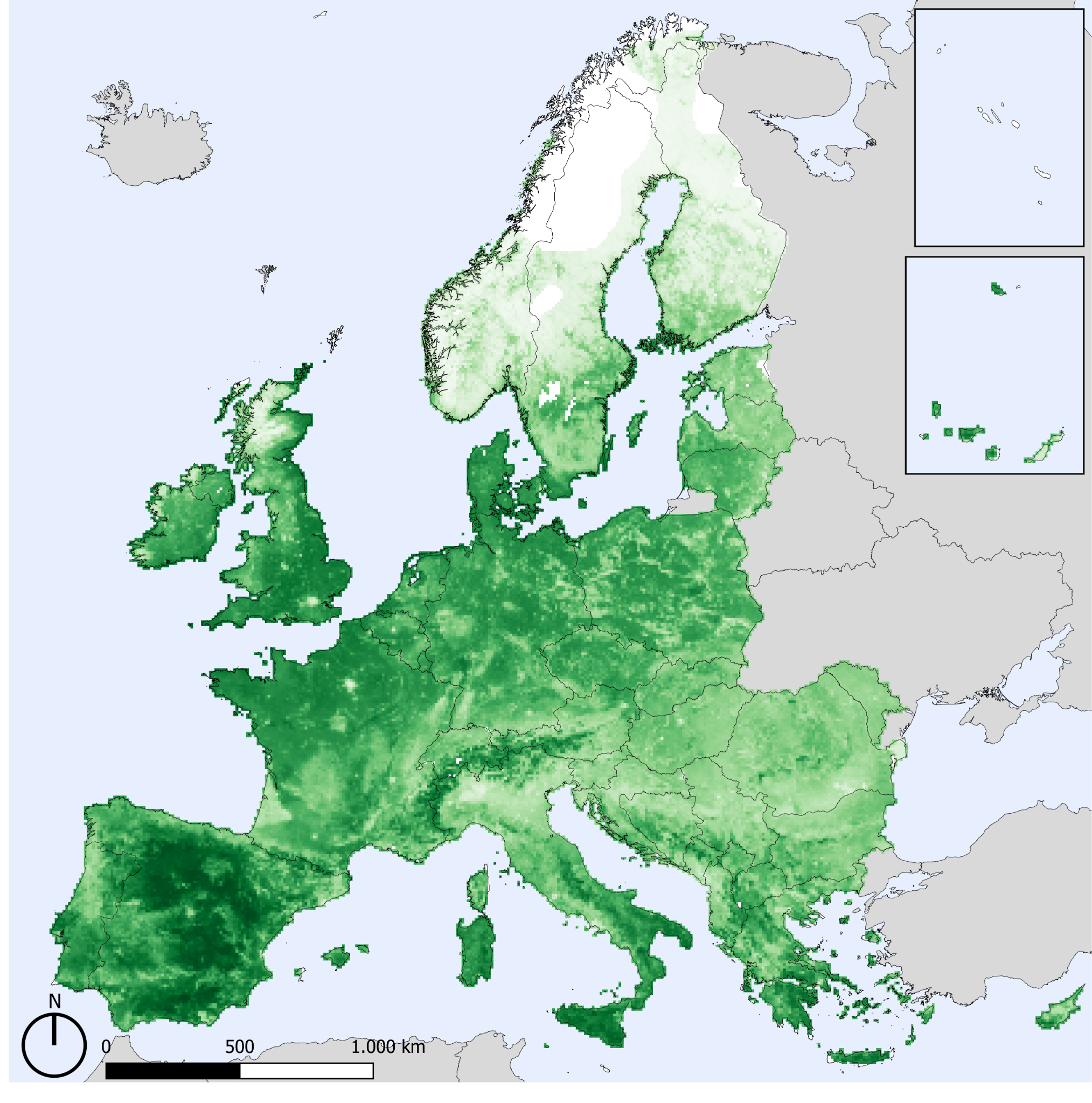* |
| *Limosa limosa* | *Linaria cannabina* |
| 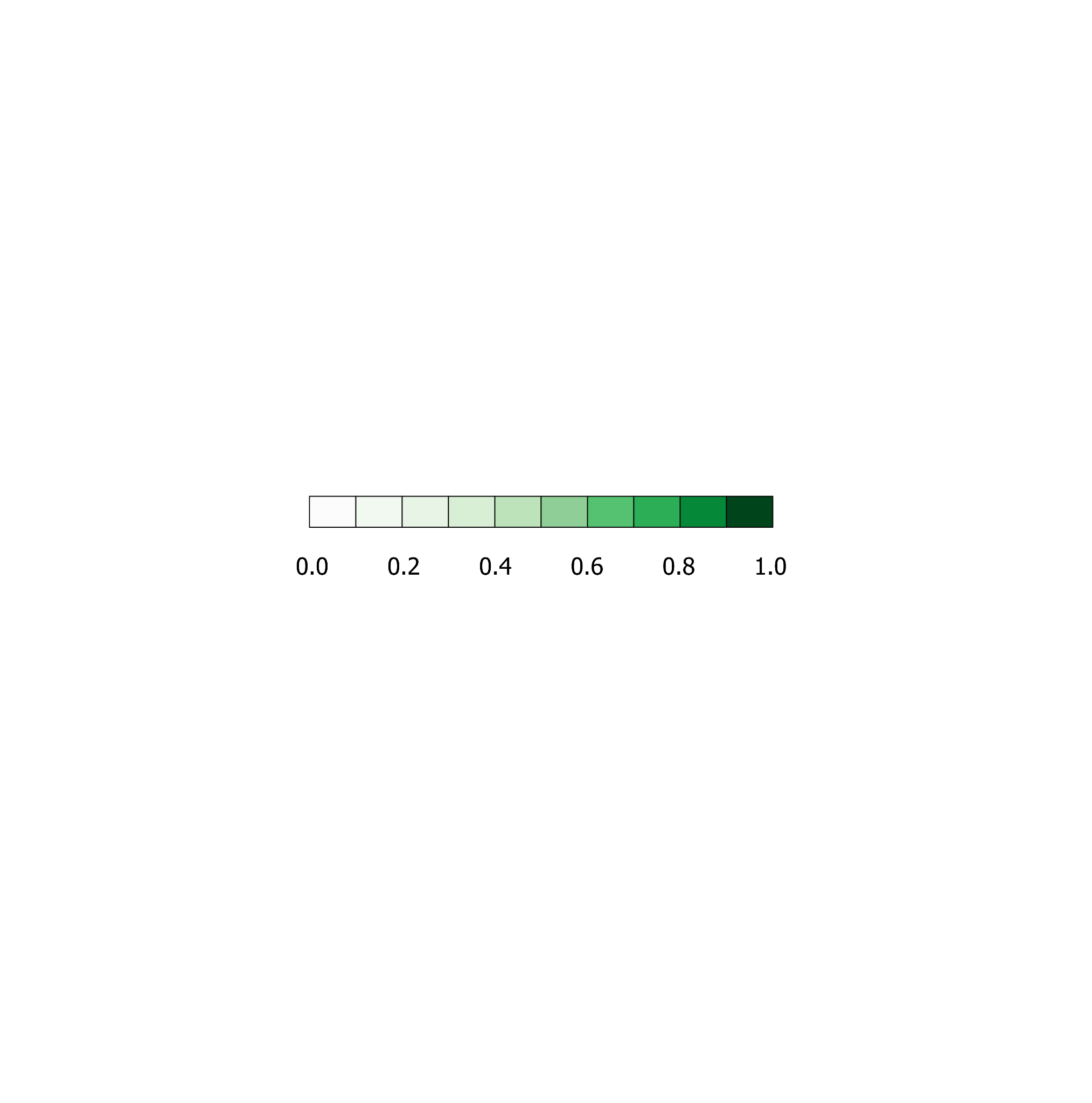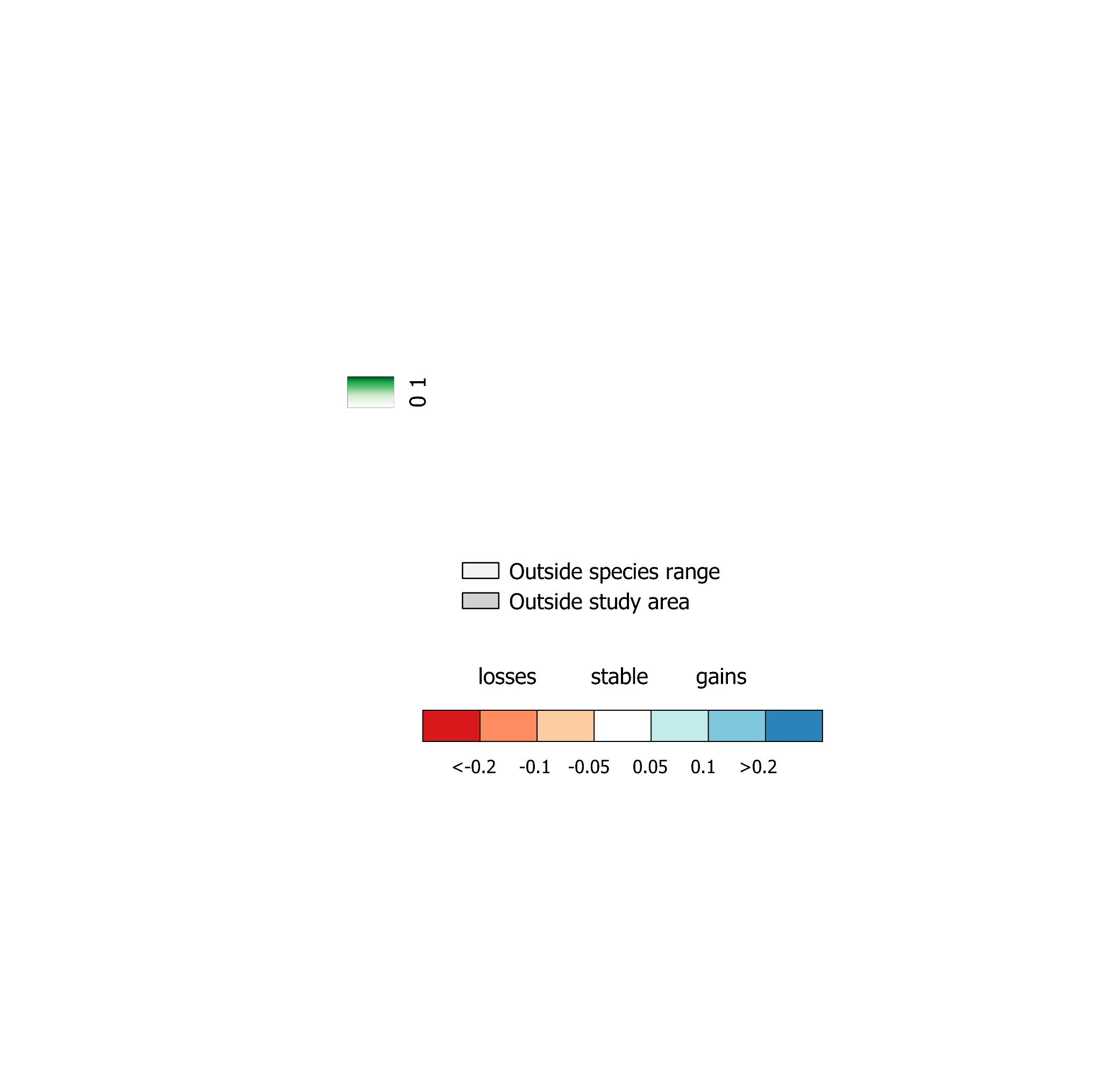  Occurrence probability | |
| *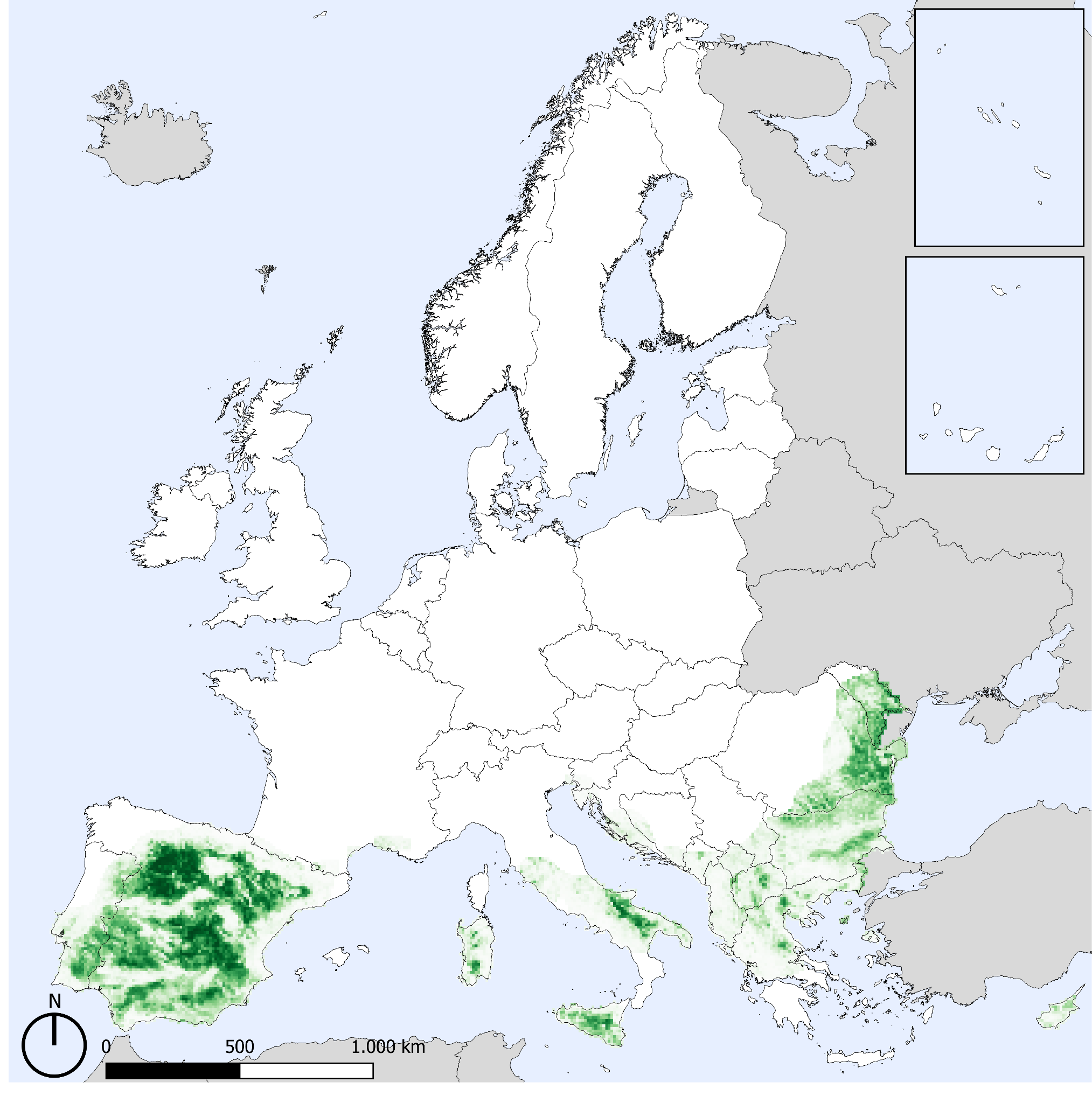* | *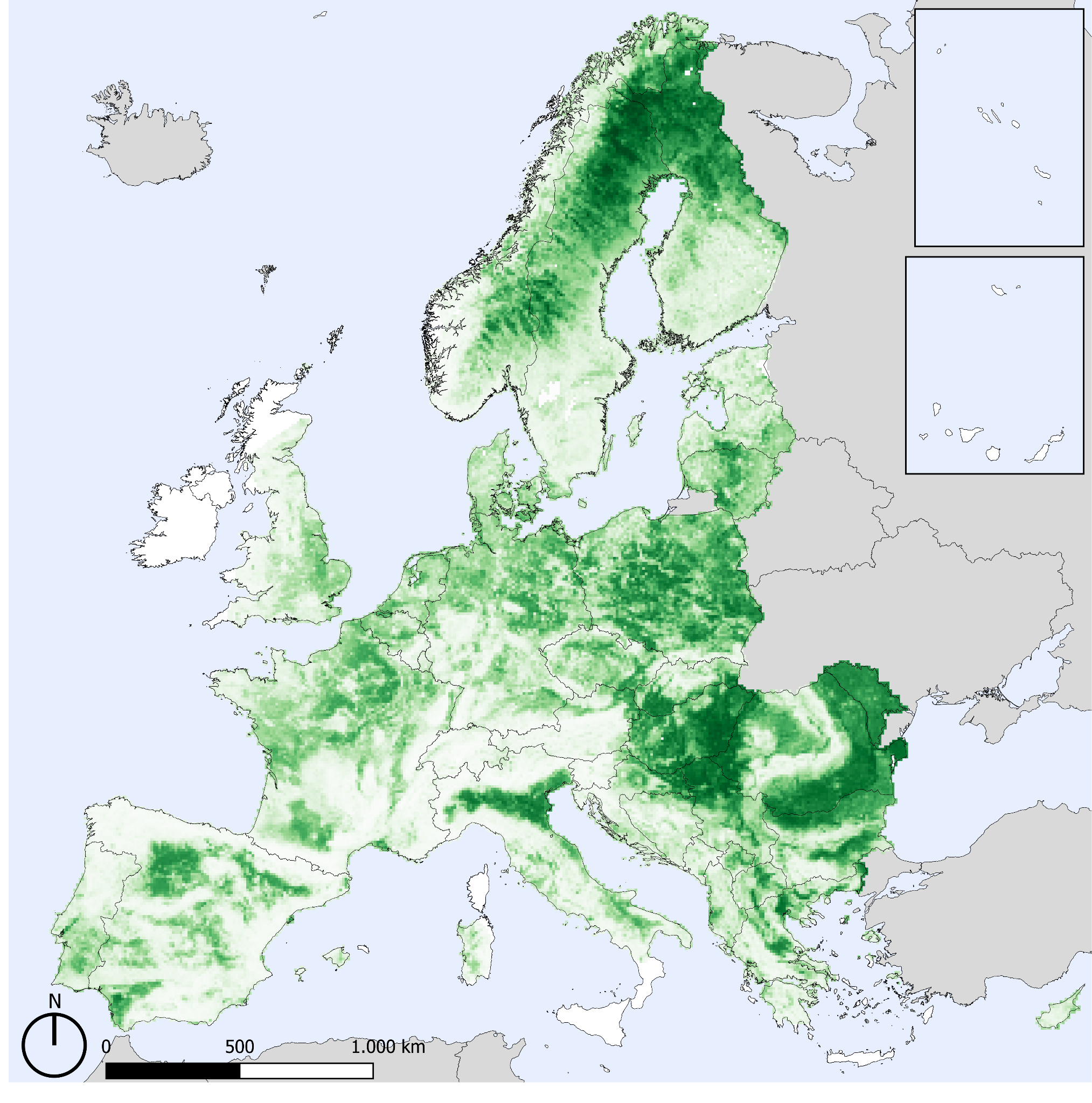* |
| *Melanocorypha calandra* | *Motacilla flava* |
| *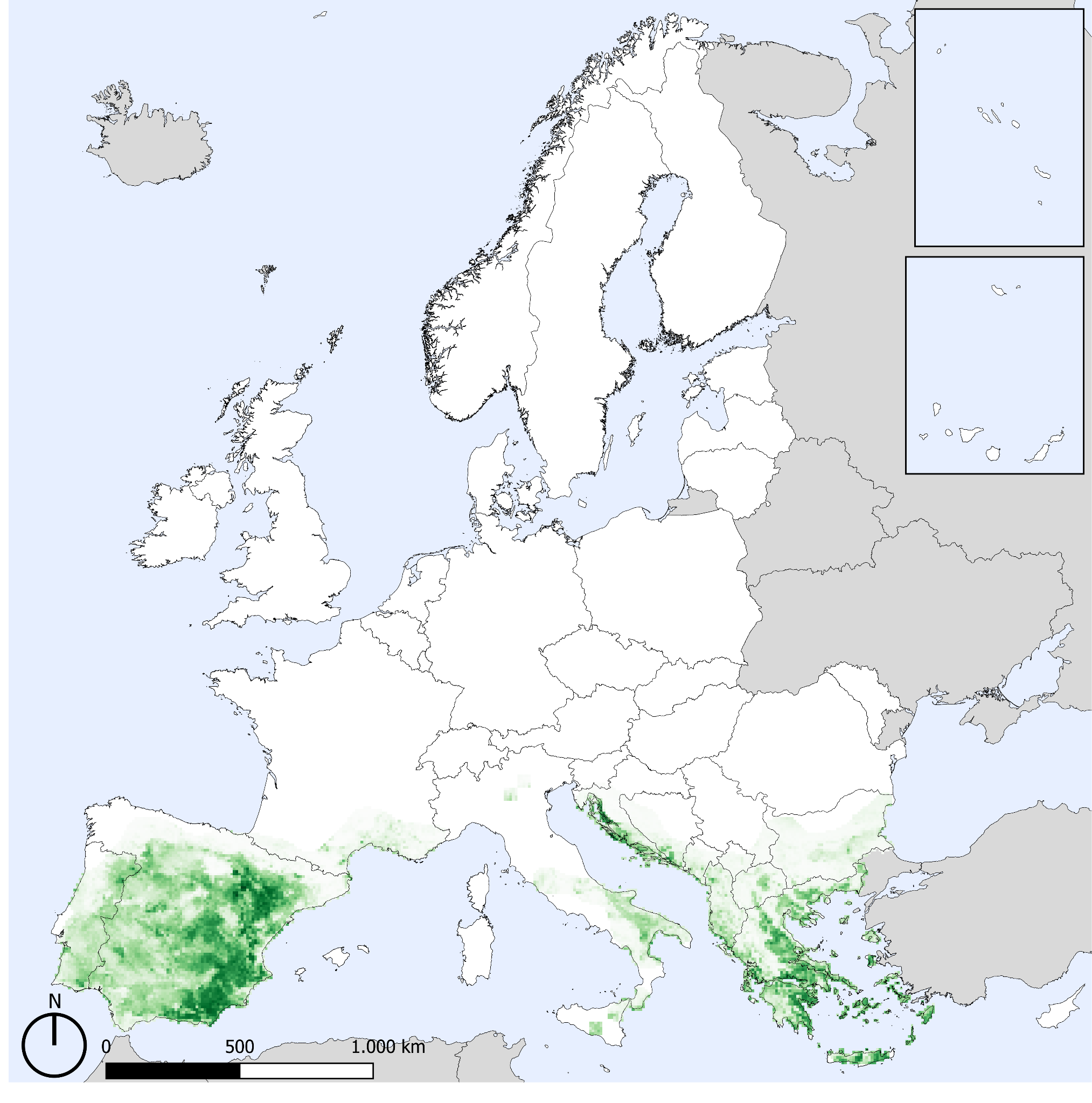* | *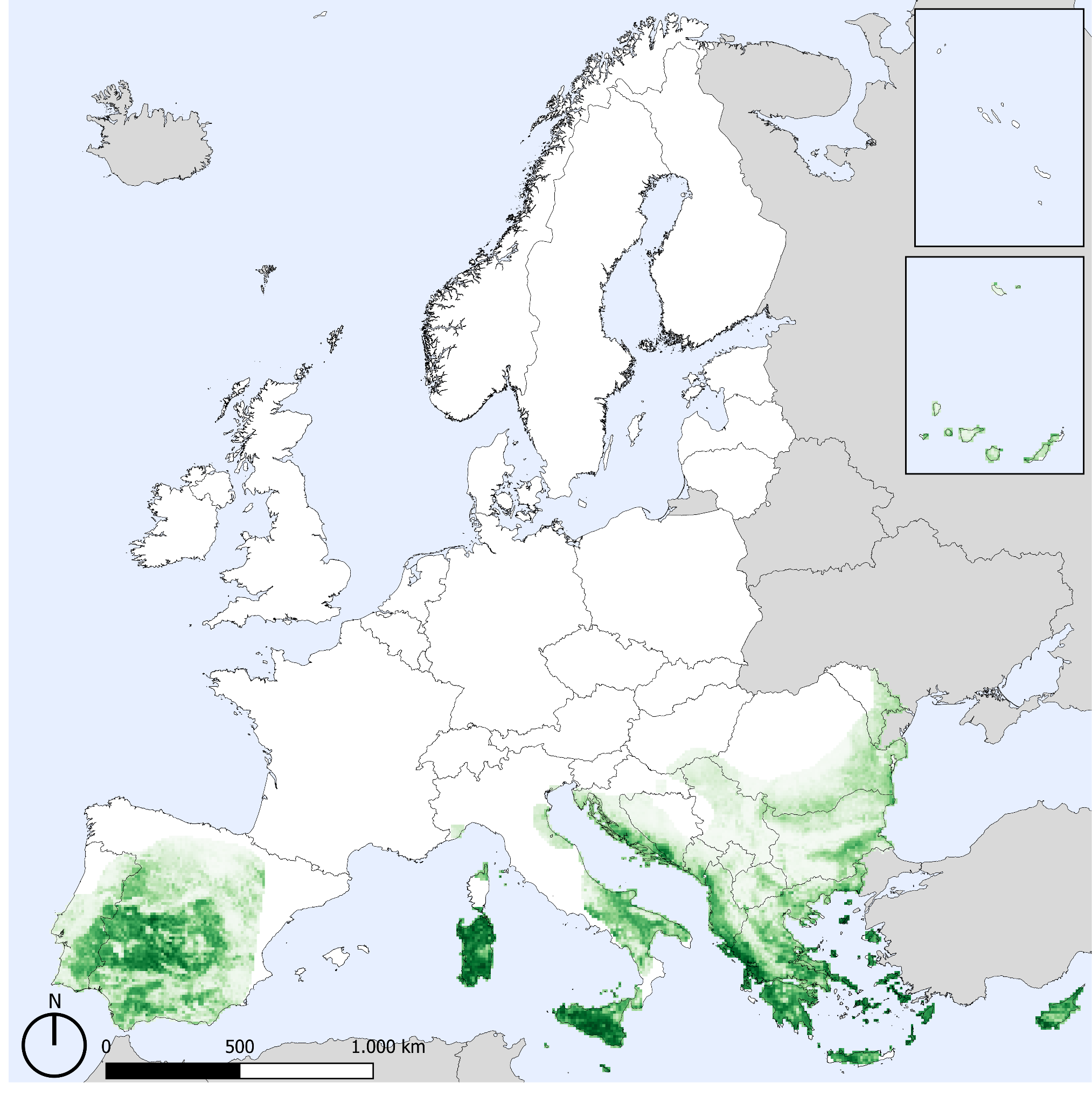* |
| *Oenanthe hispanica* | *Passer hispaniolensis* |
| 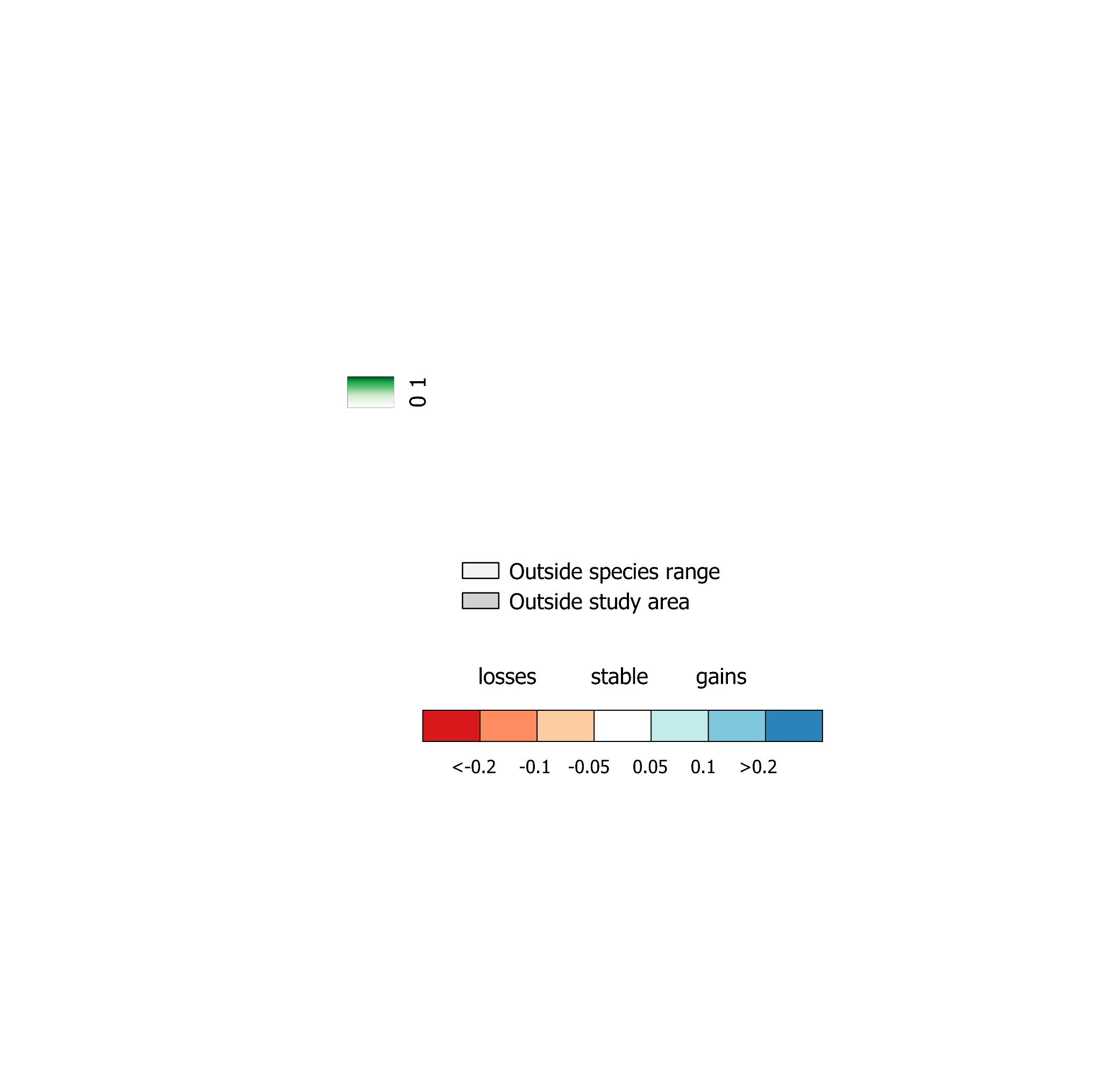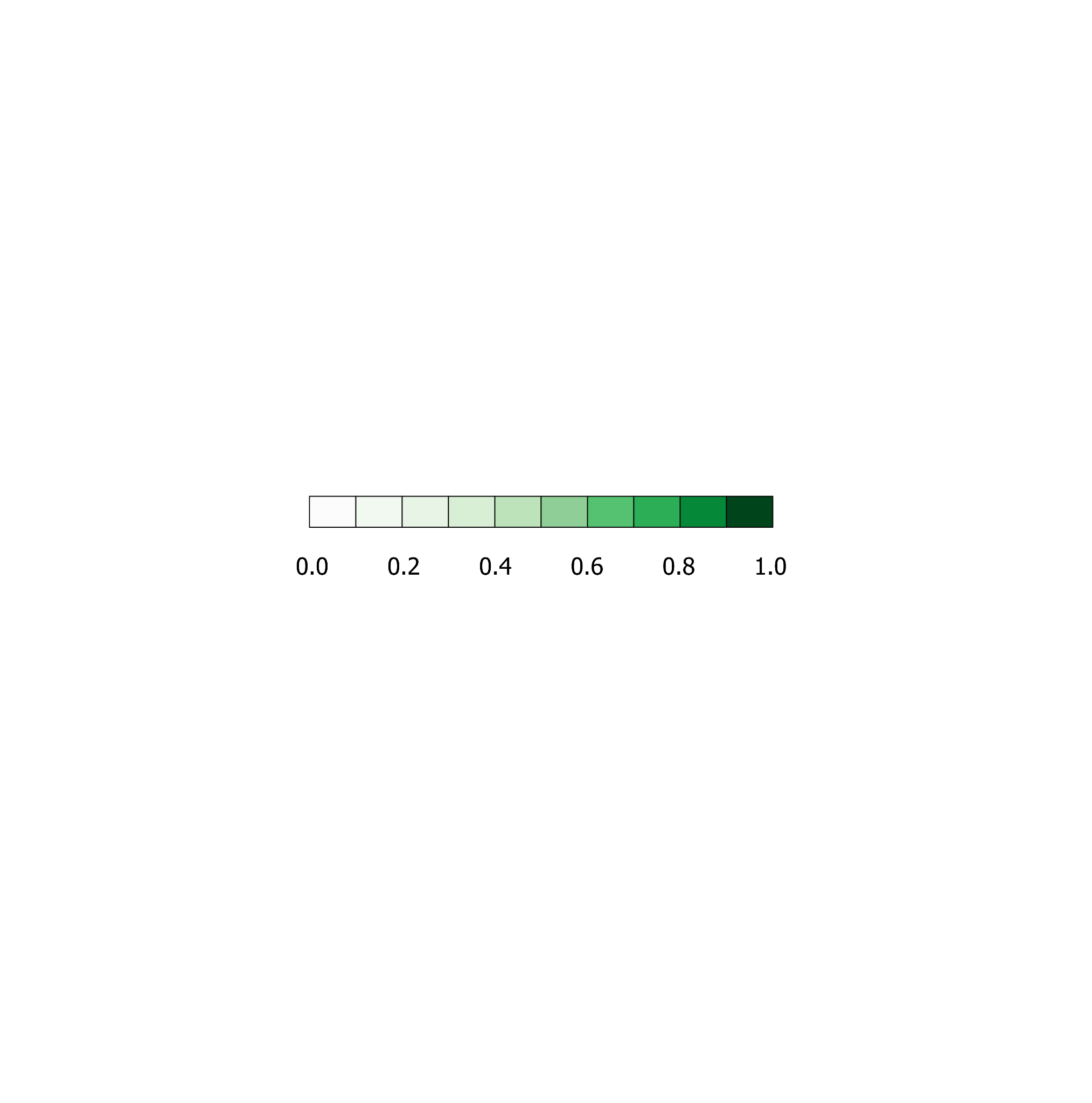  Occurrence probability | |
| *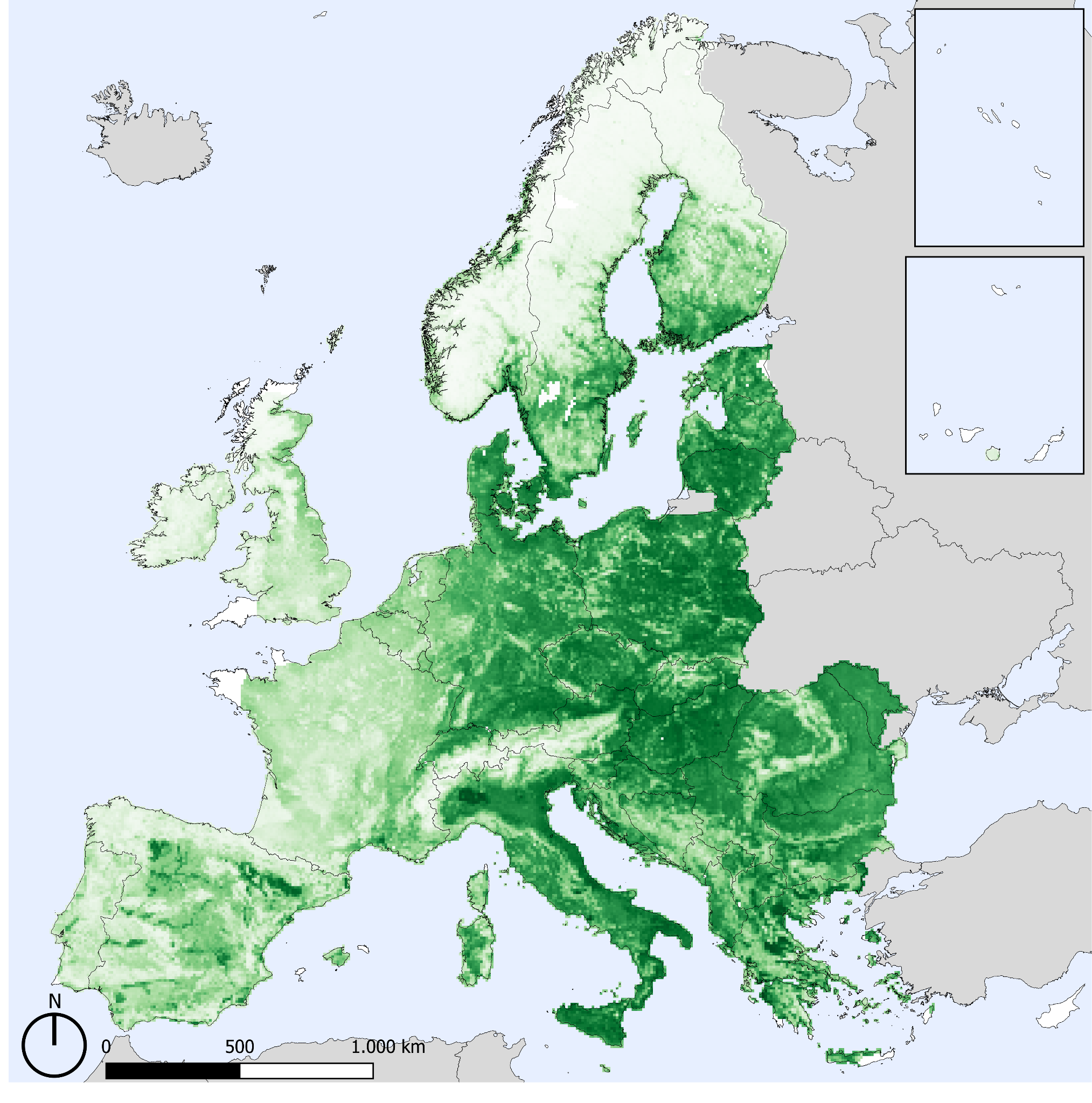* | *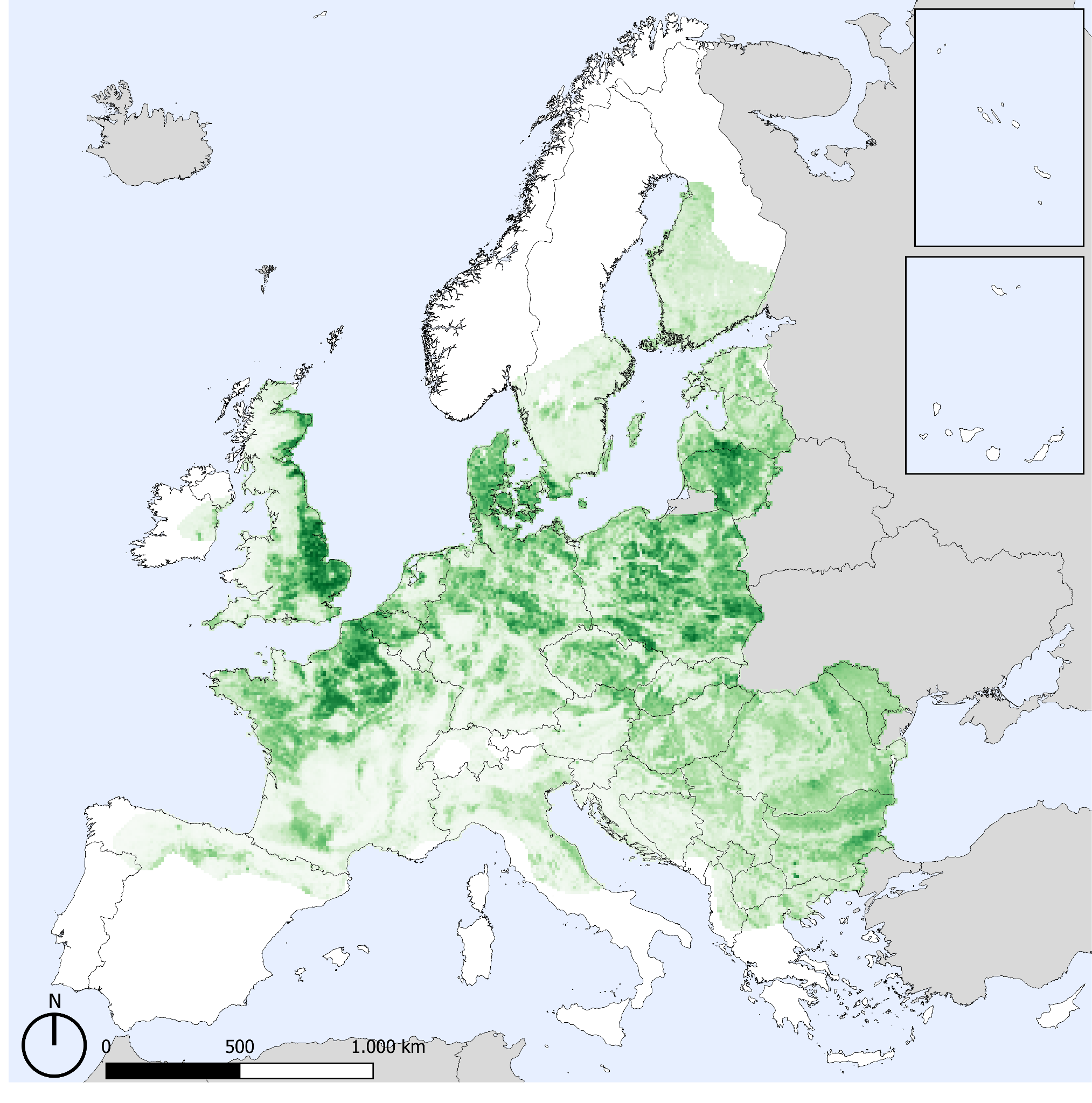* |
| *Passer montanus* | *Perdix perdix* |
| *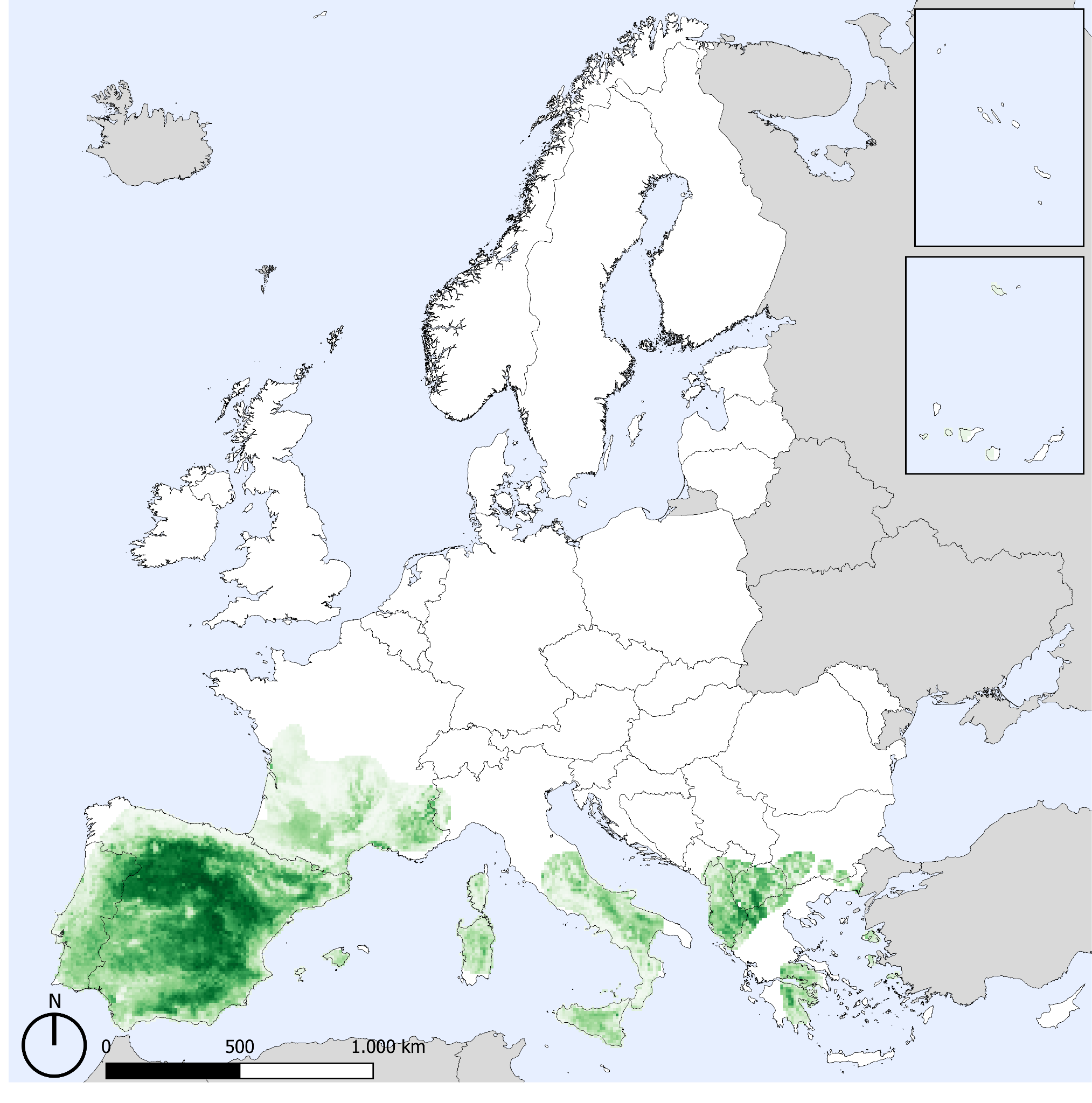* | *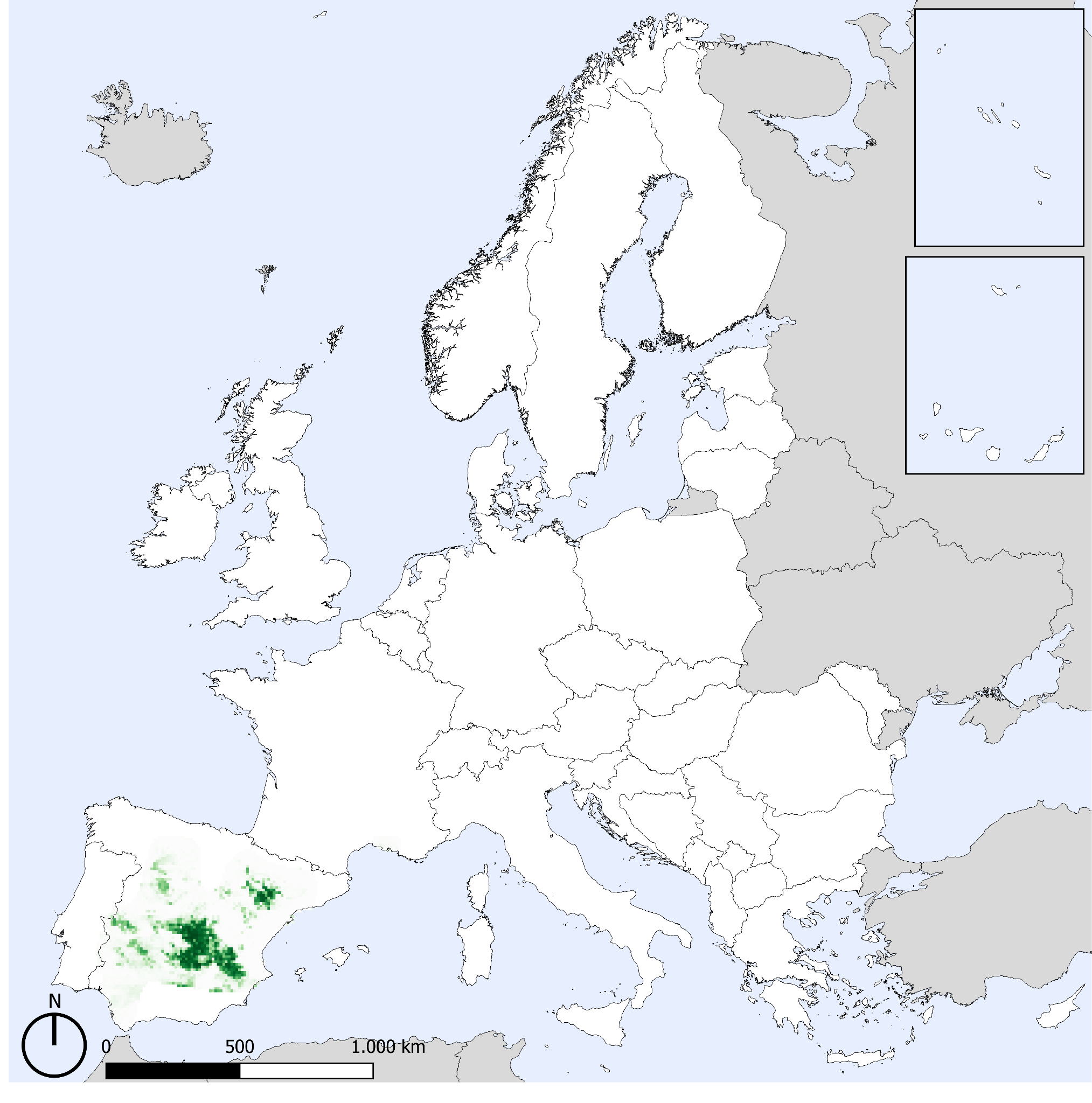* |
| *Petronia petronia* | *Pterocles alchata* |
| 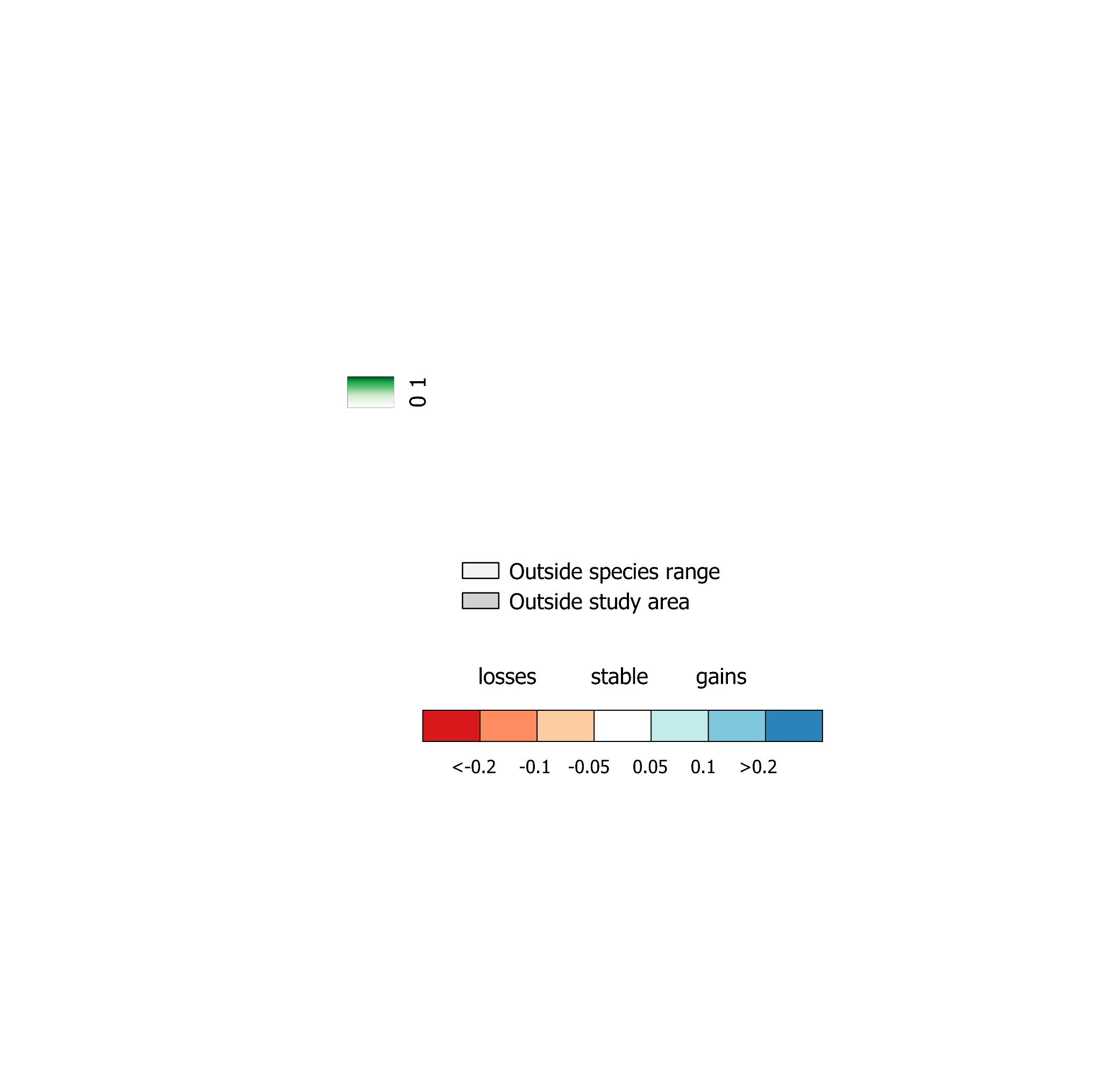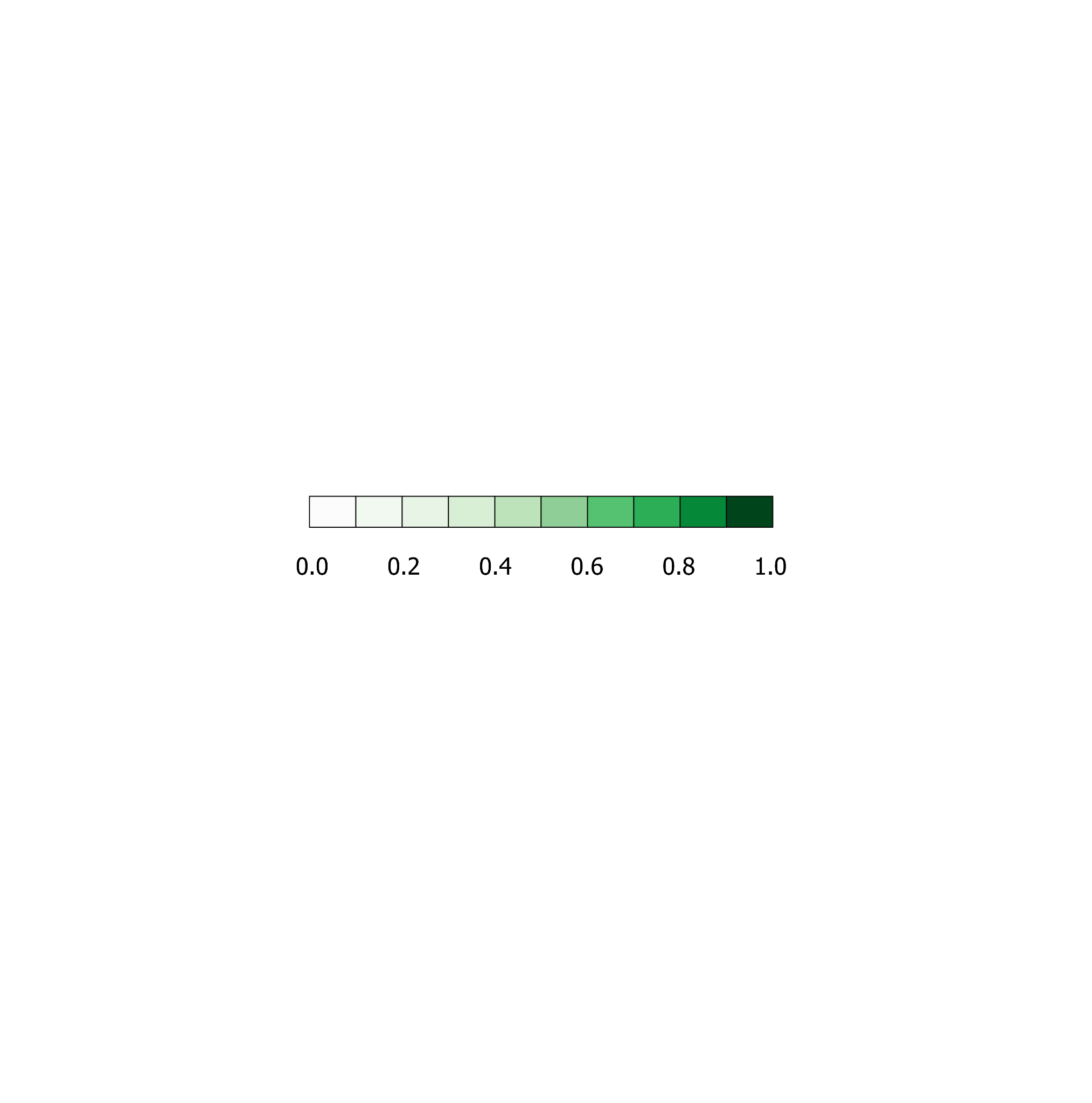  Occurrence probability | |
| *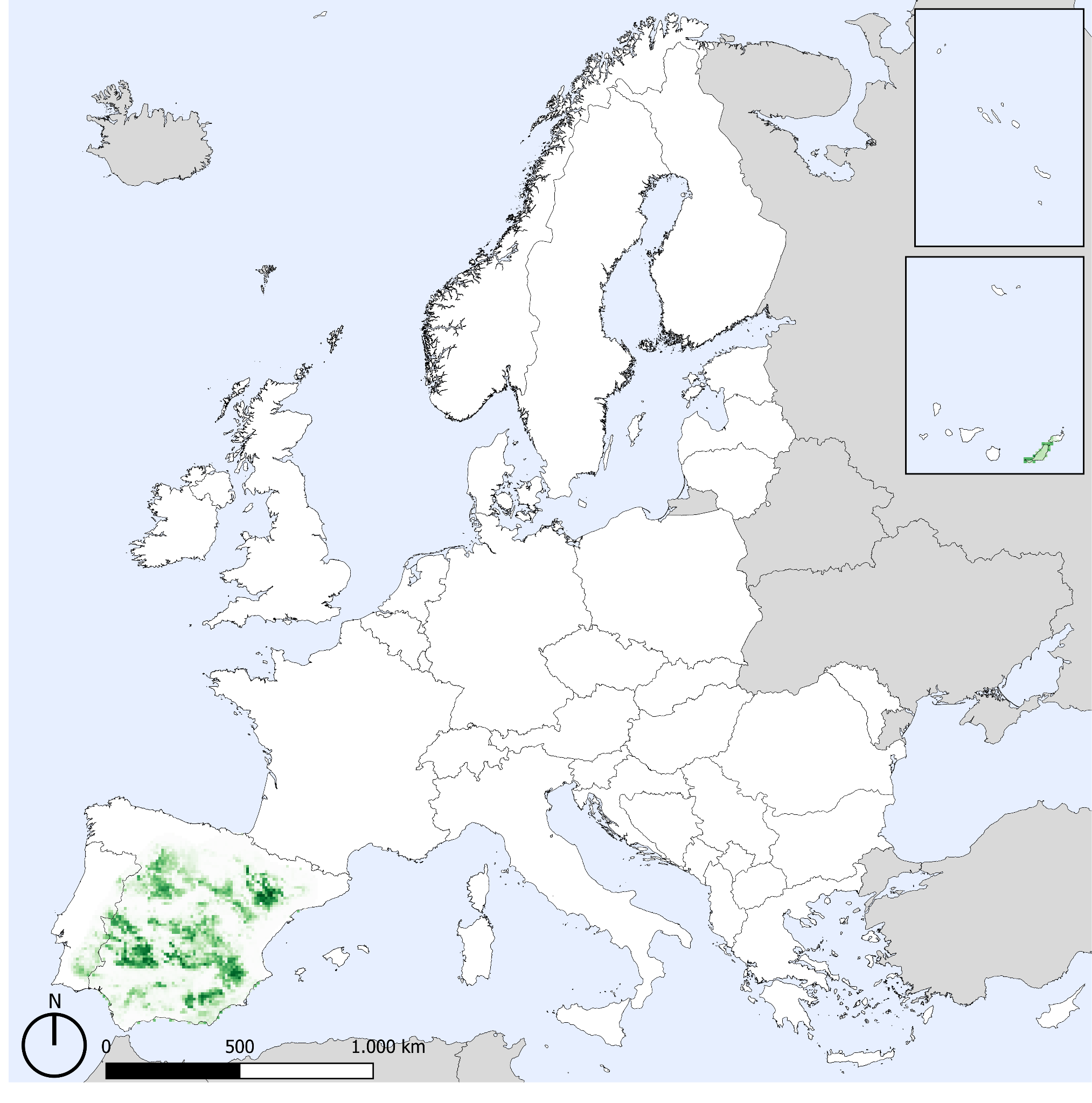* | *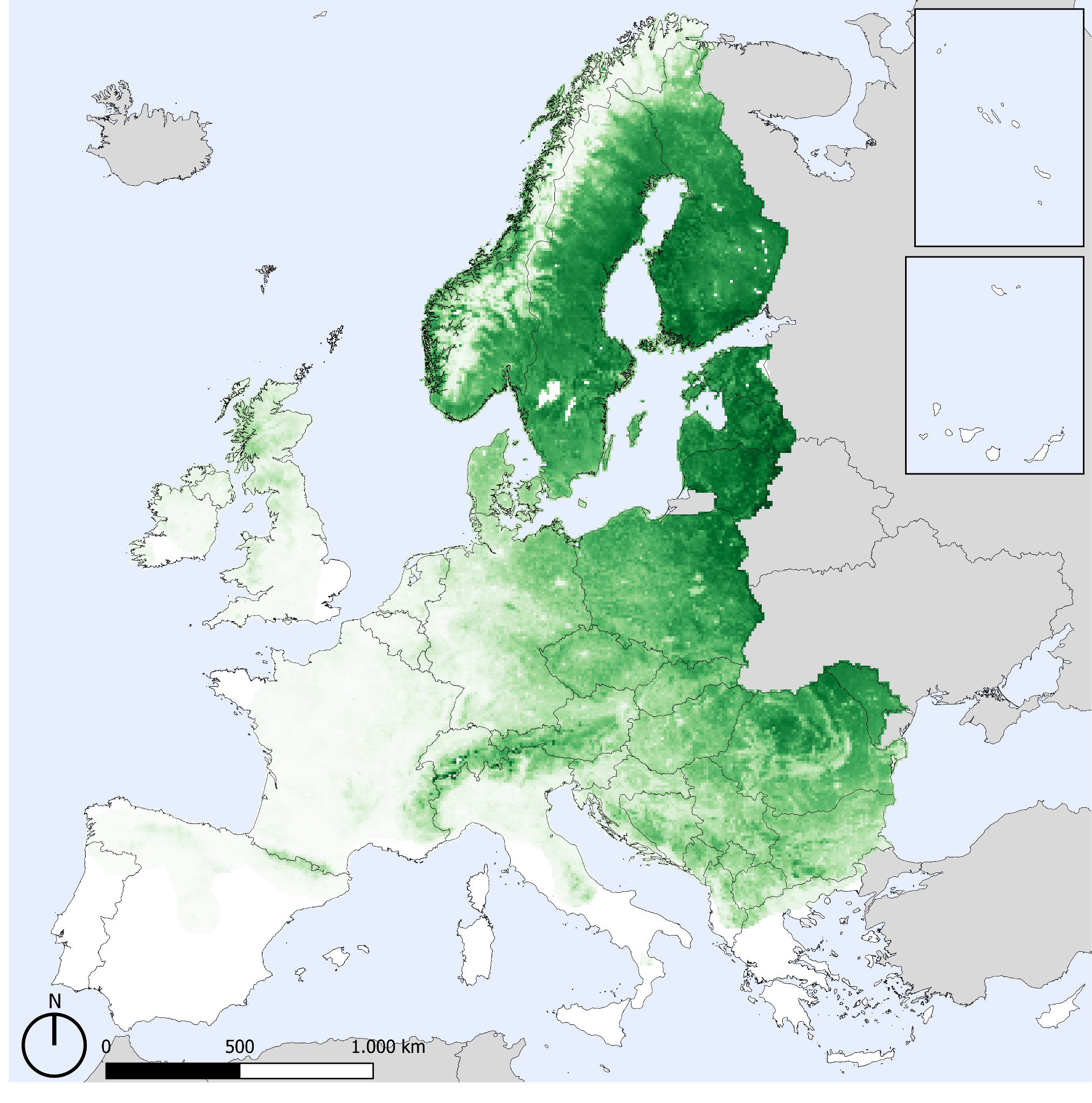* |
| *Pterocles orientalis* | *Saxicola rubetra* |
| *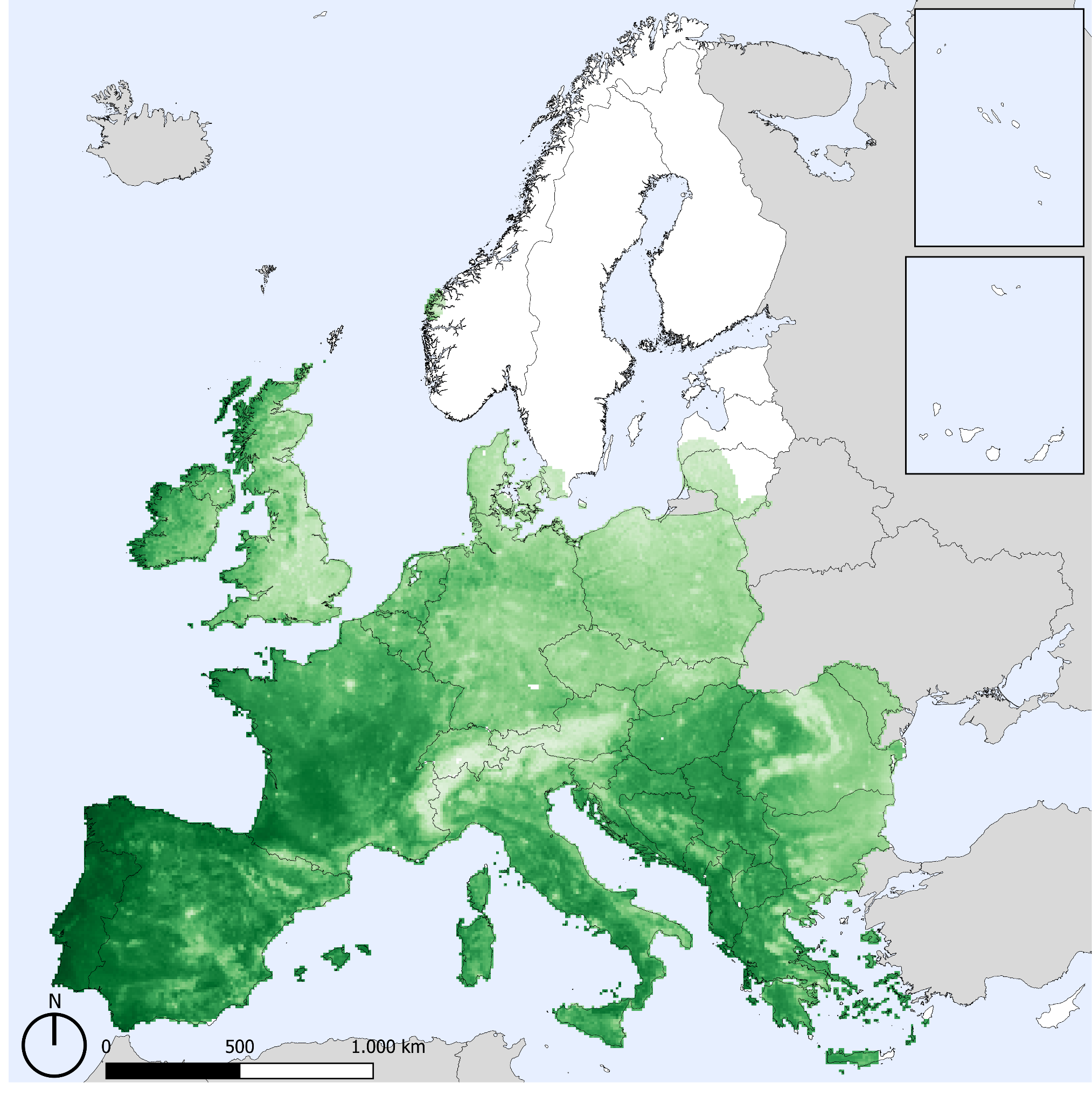* | *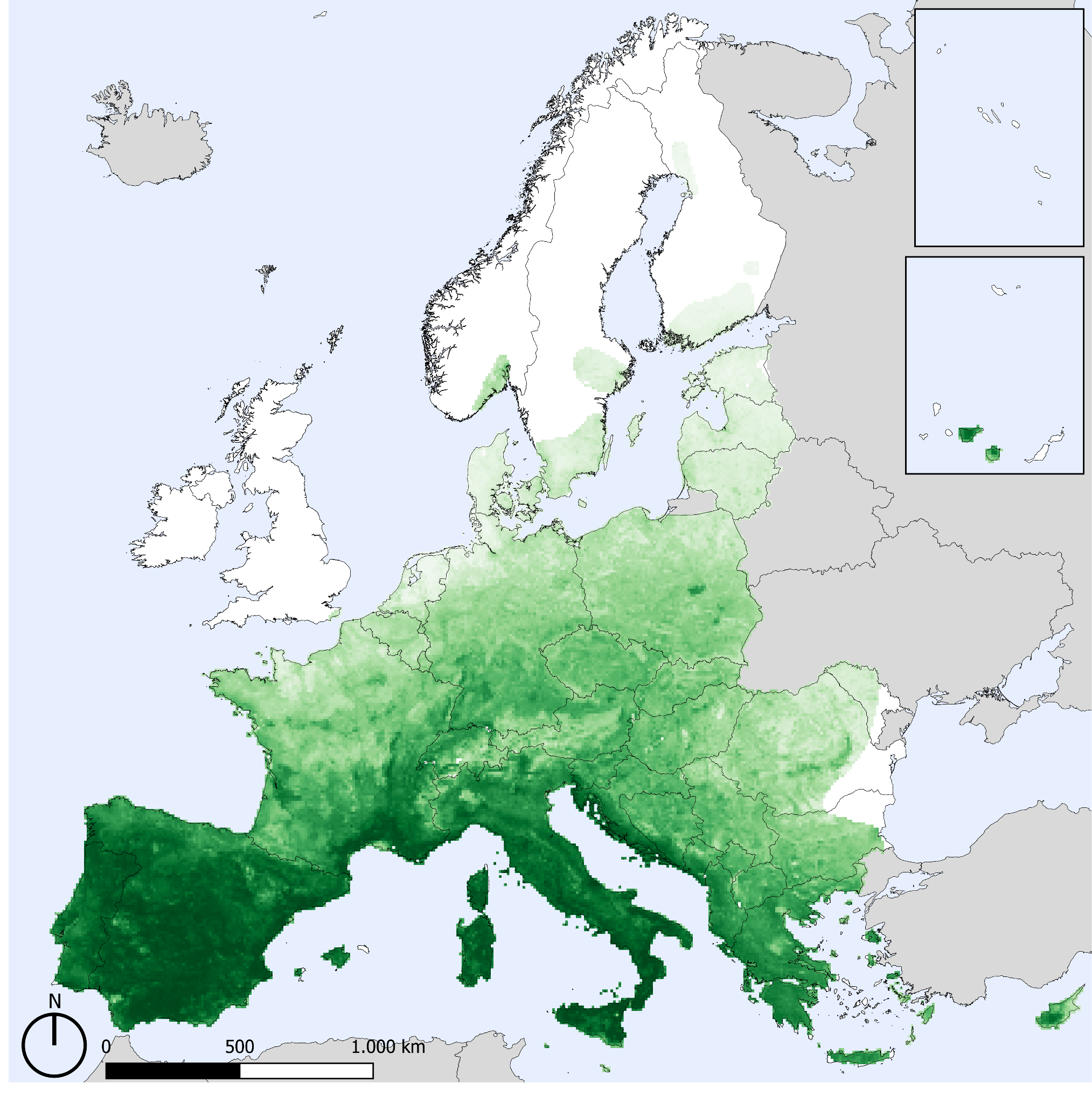* |
| *Saxicola torquatus* | *Serinus serinus* |
| 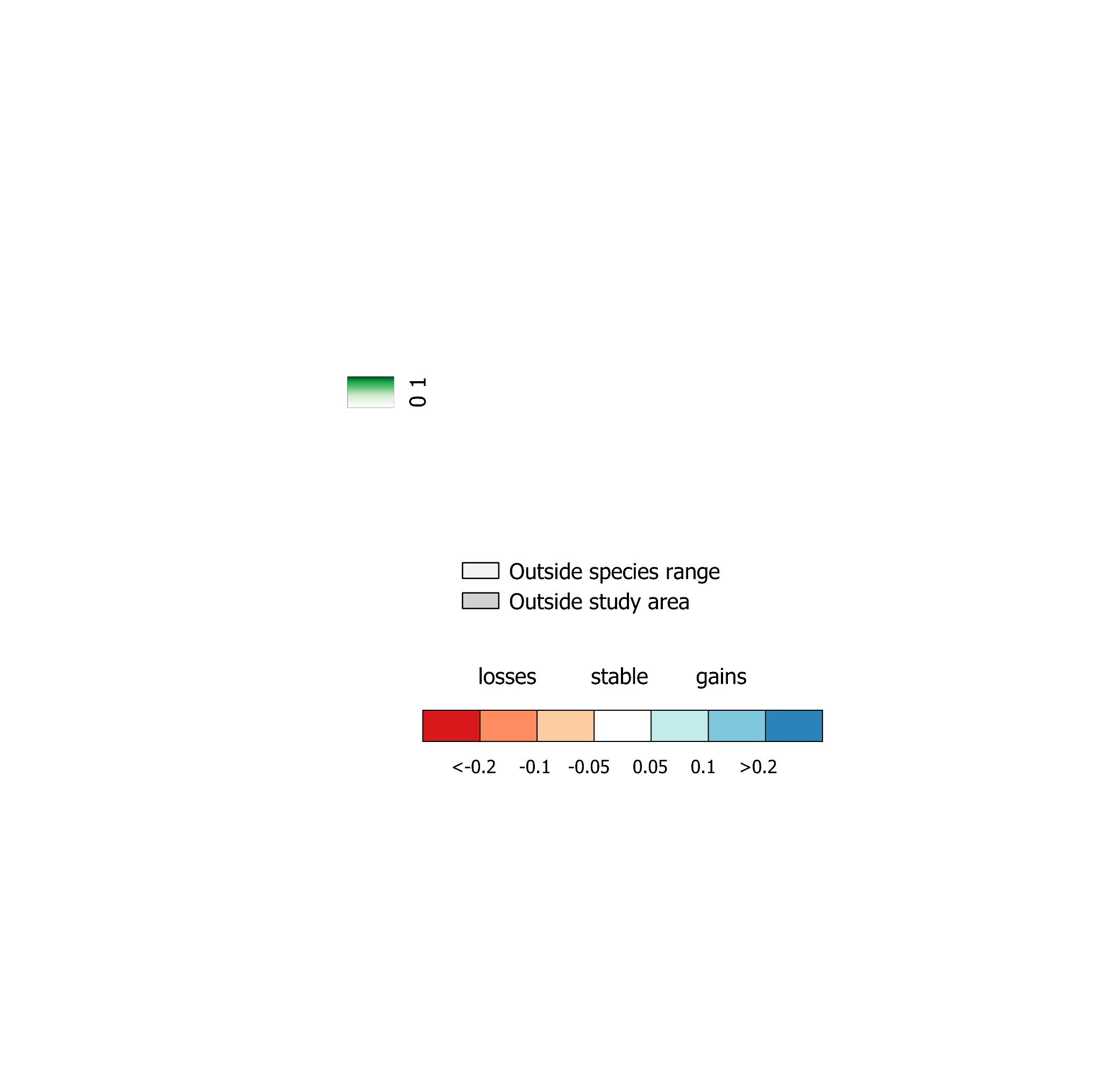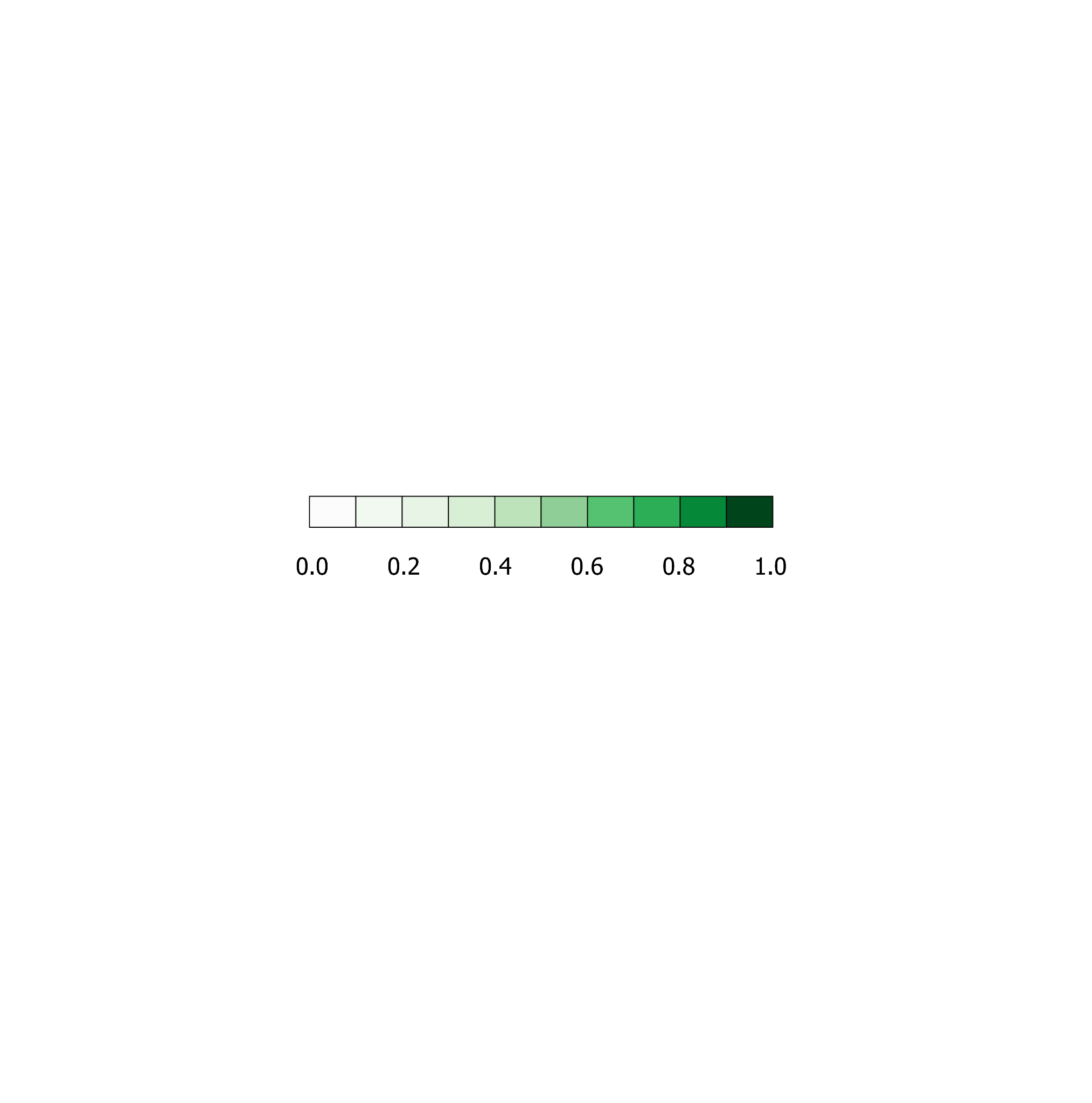  Occurrence probability | |
| *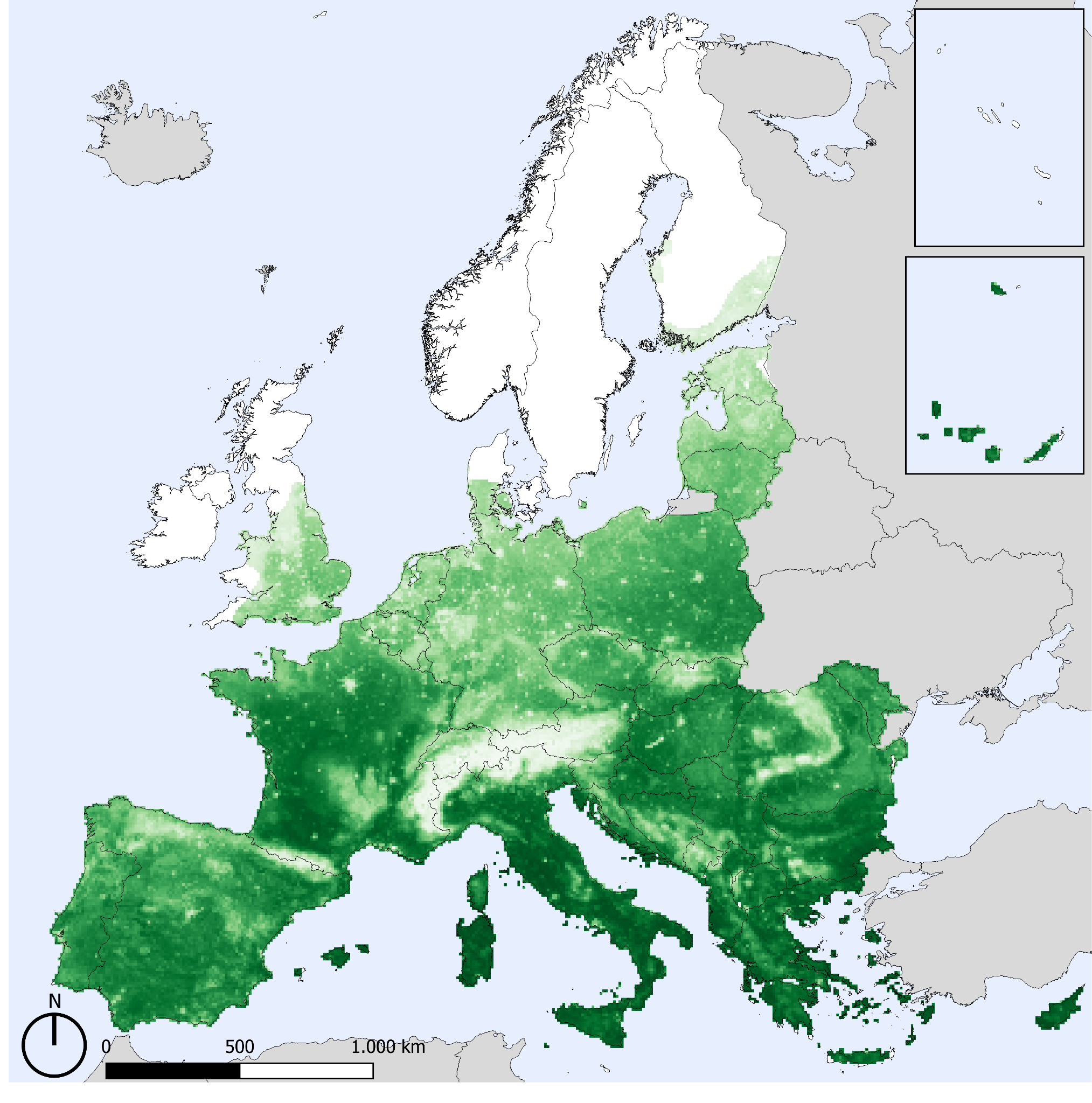* | *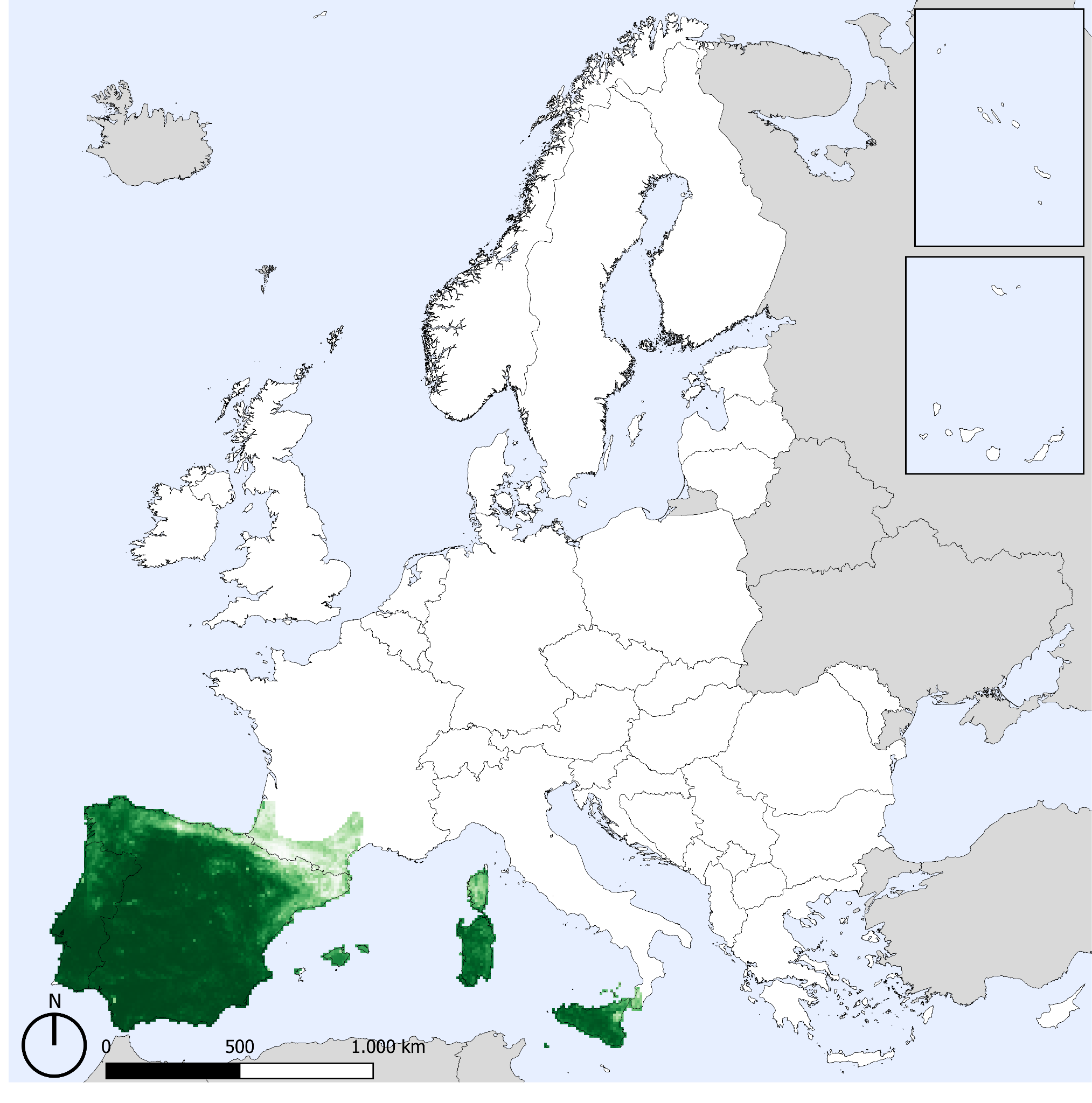* |
| *Streptopelia turtur* | *Sturnus unicolor* |
| *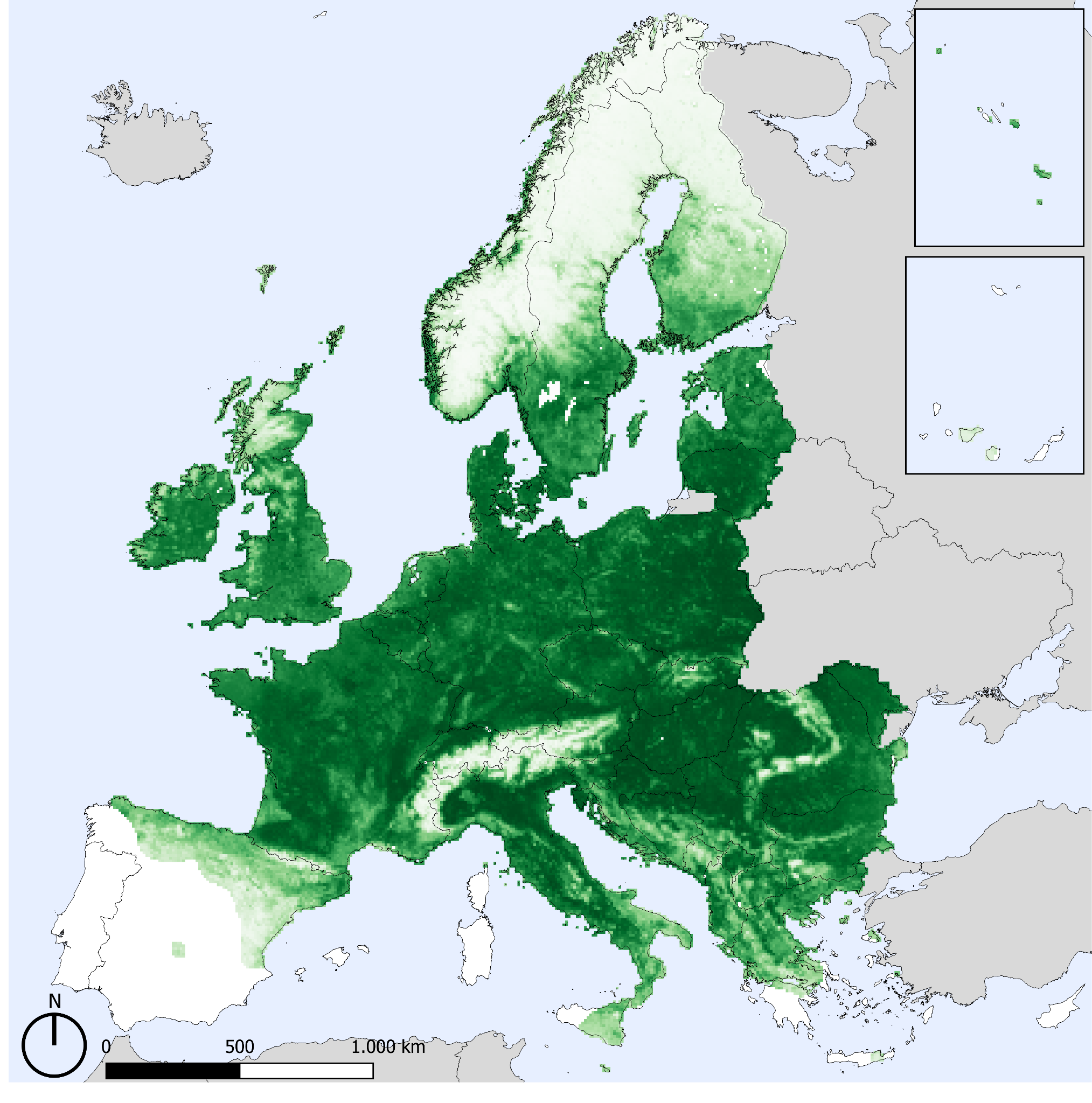* | *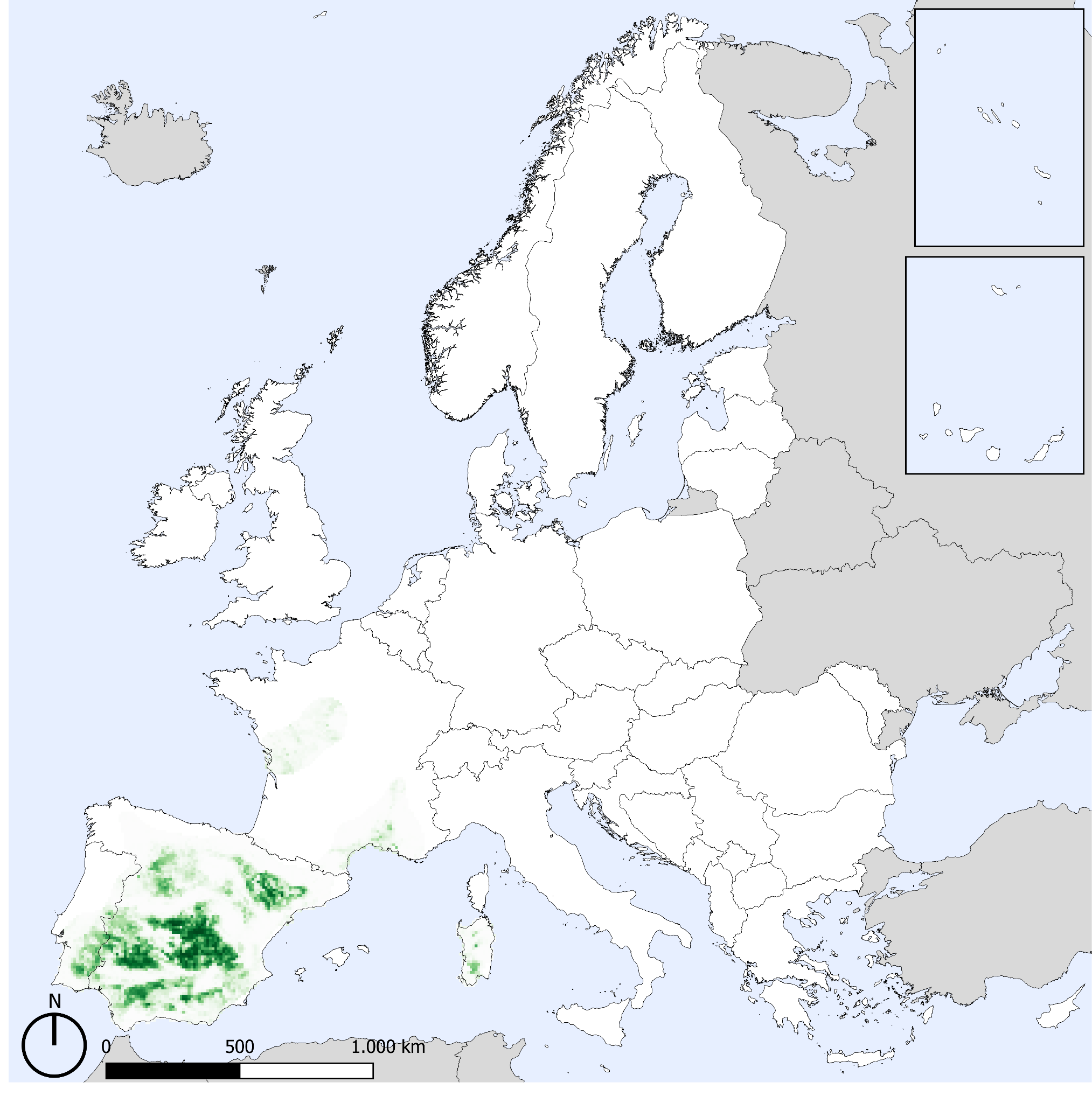* |
| *Sturnus vulgaris* | *Tetrax tetrax* |
| 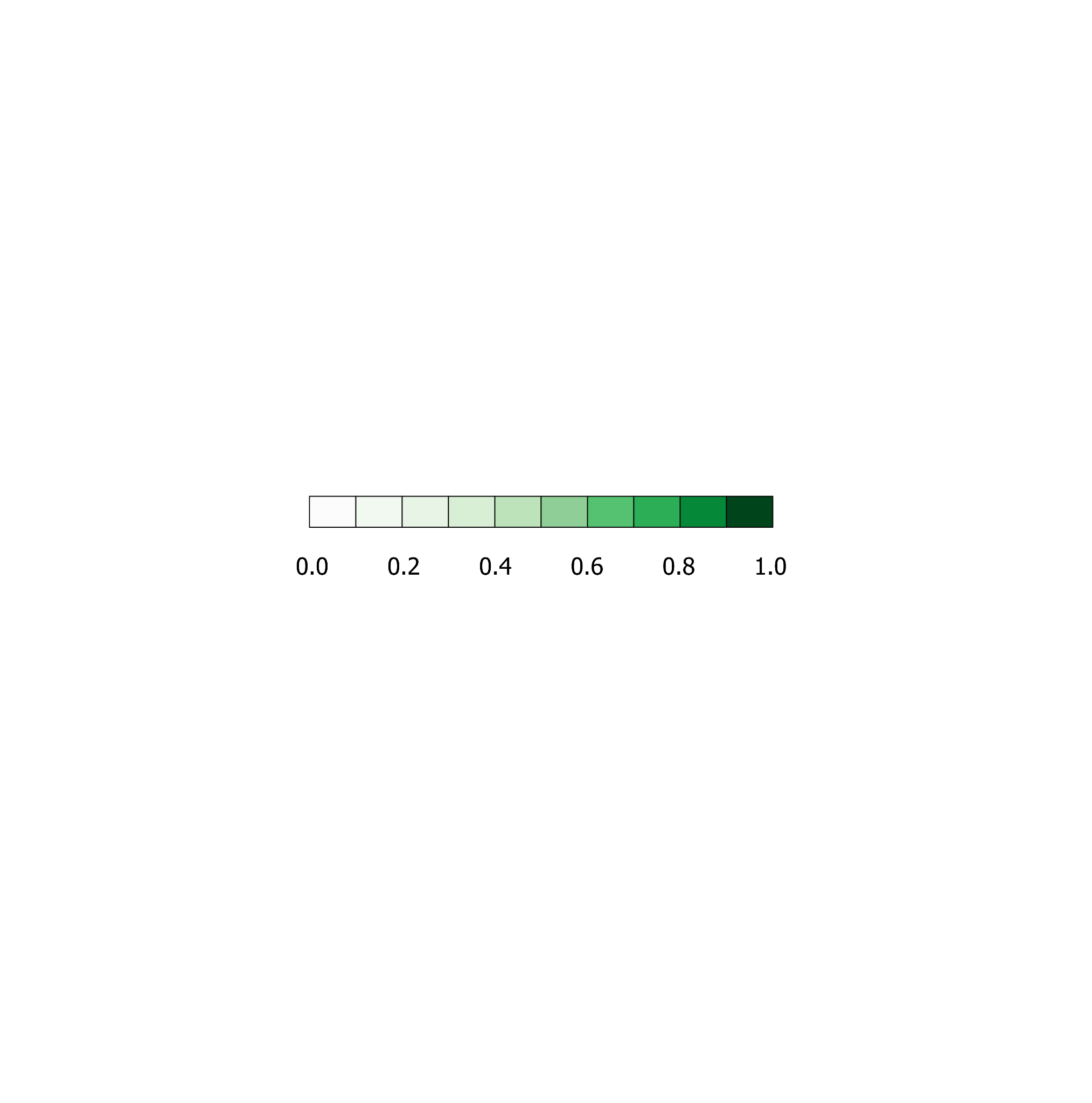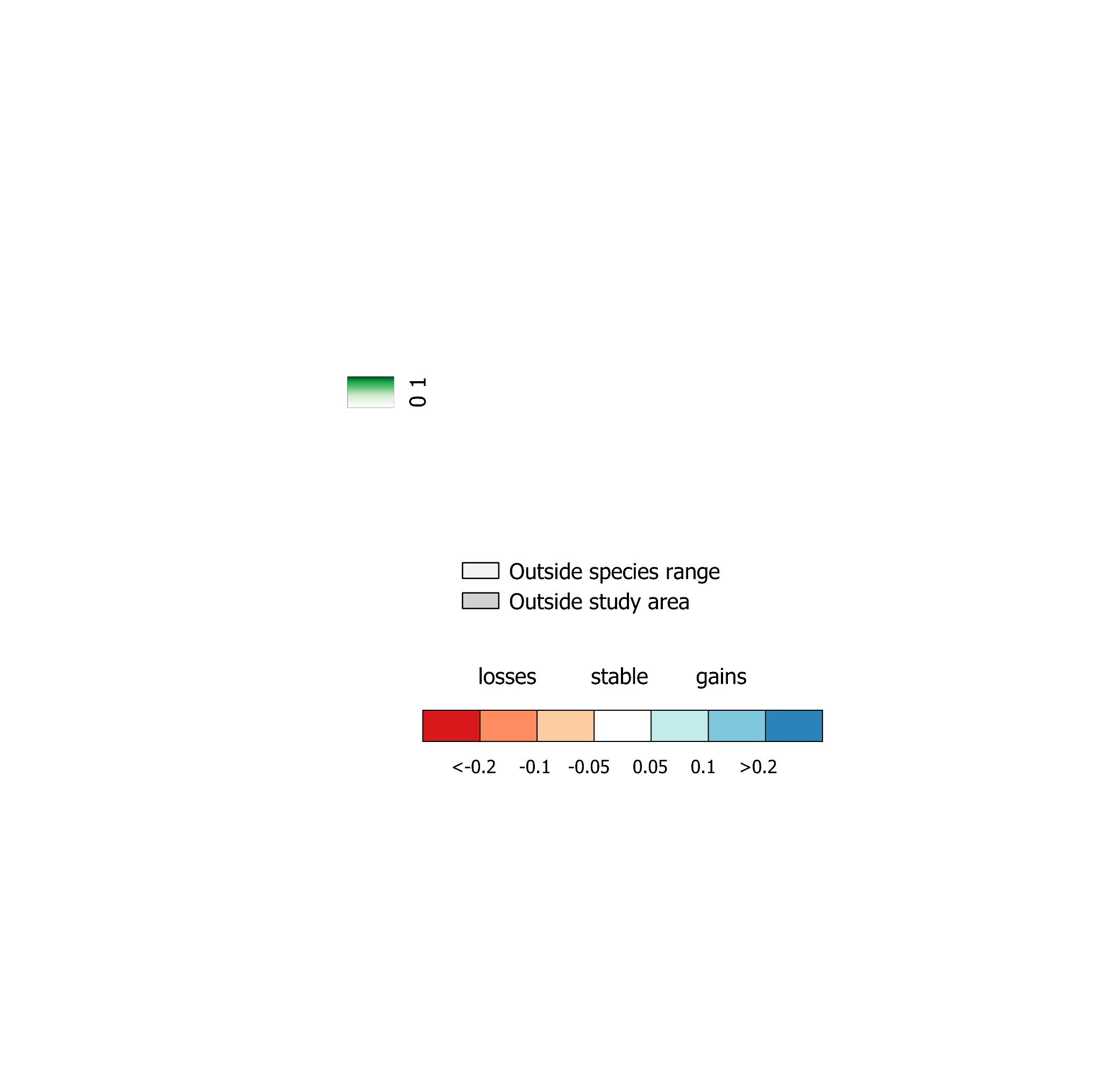  Occurrence probability | |
| *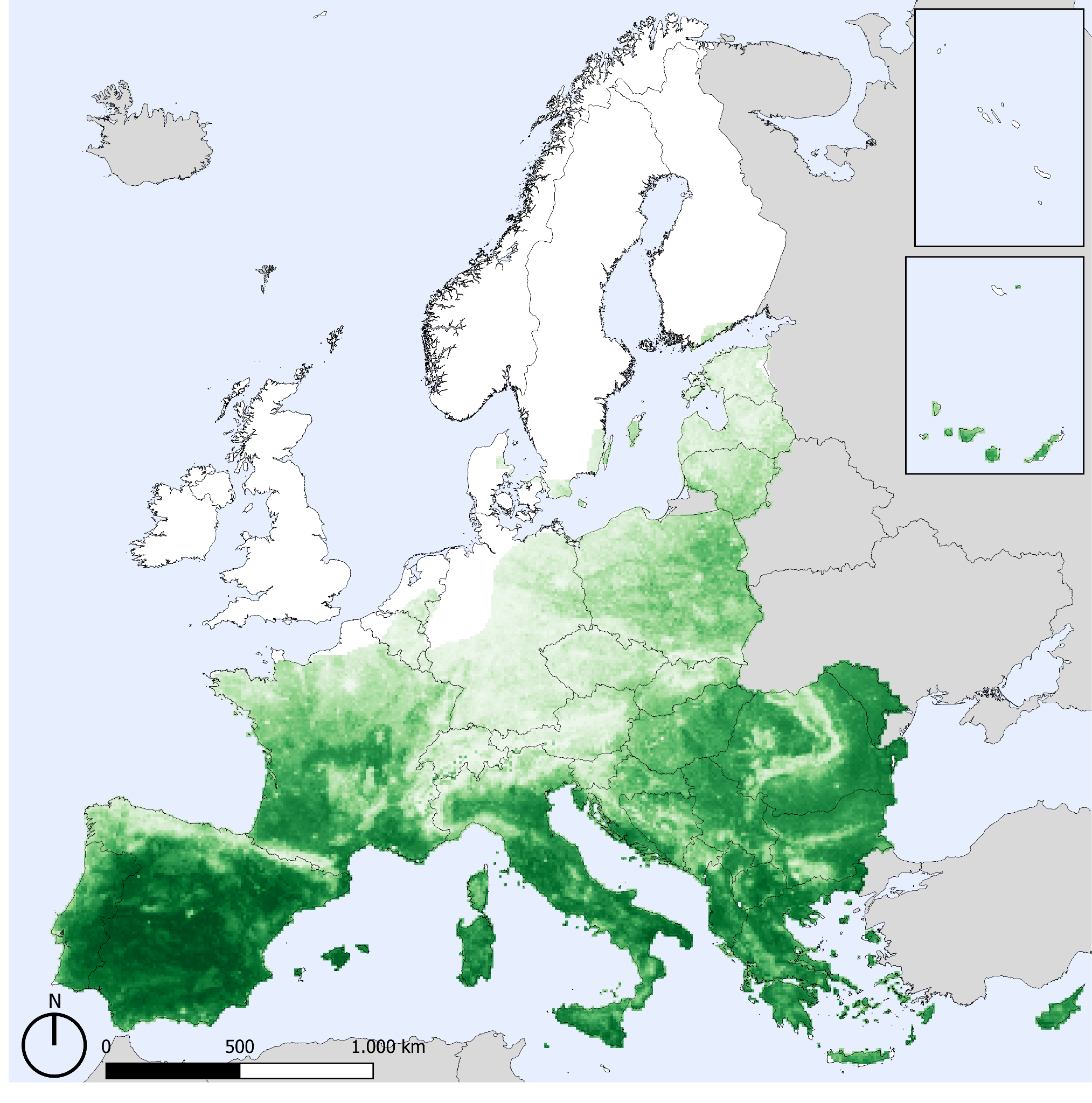* | *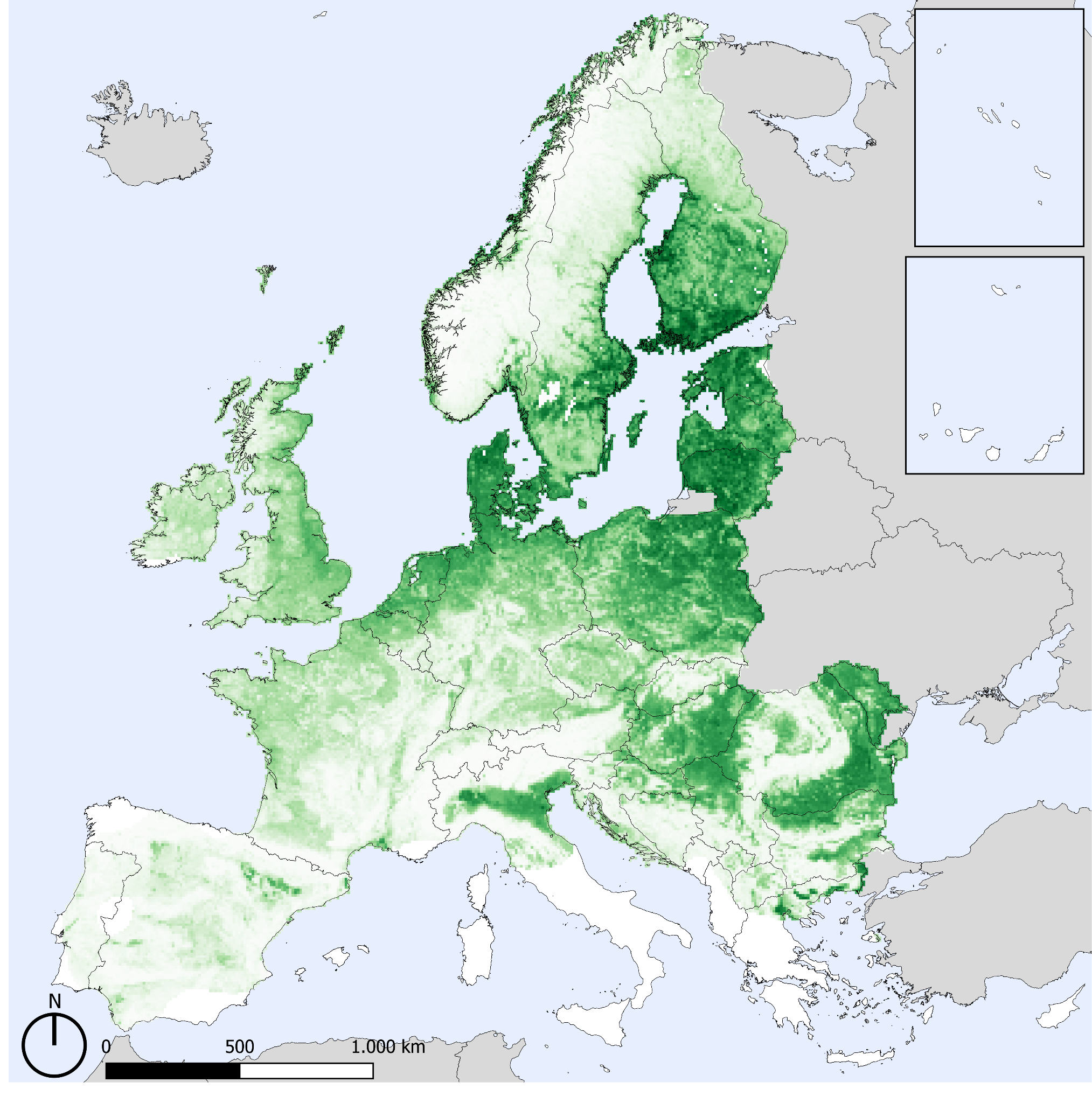* |
| *Upupa epops* | *Vanellus vanellus* |
| 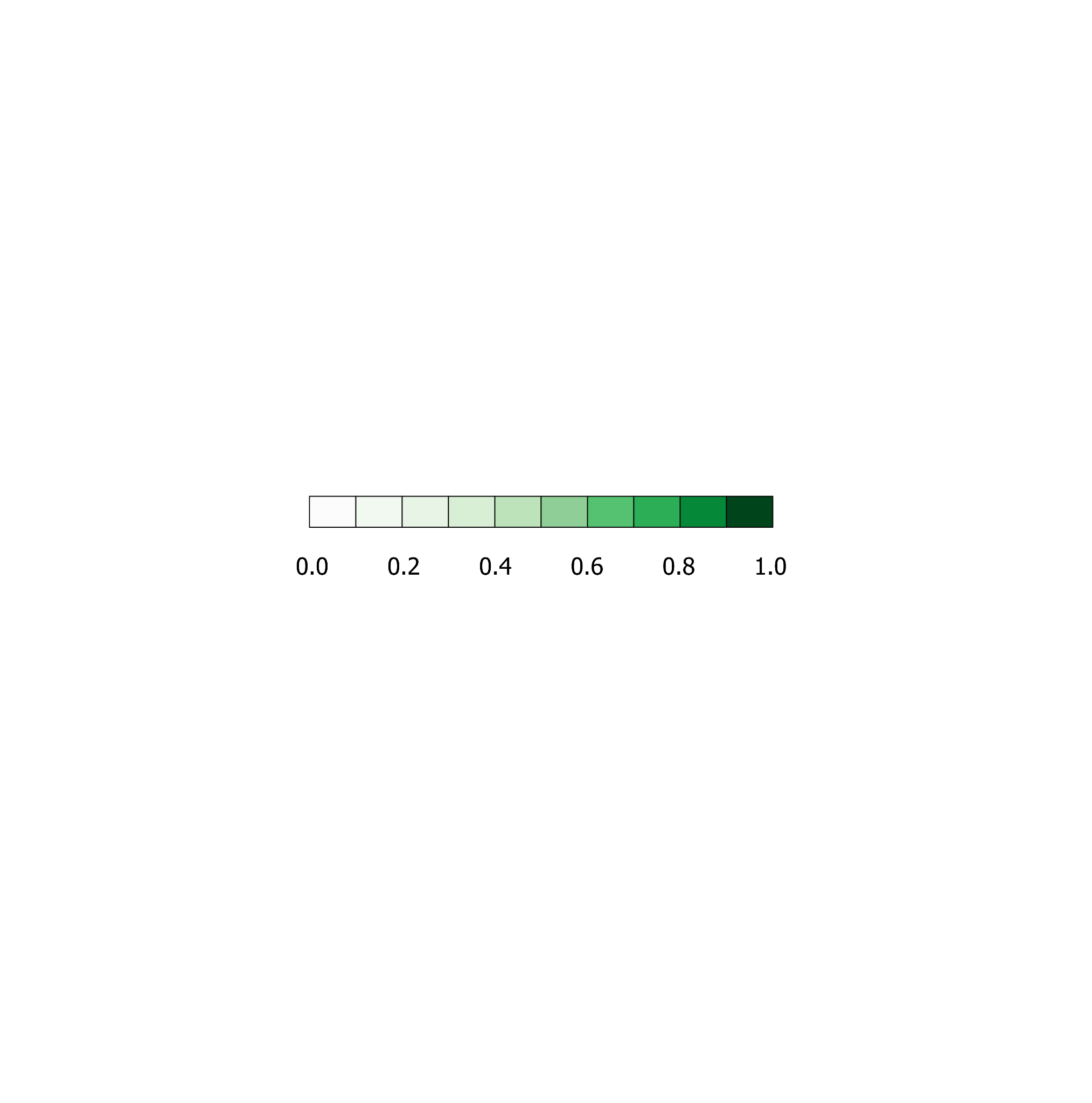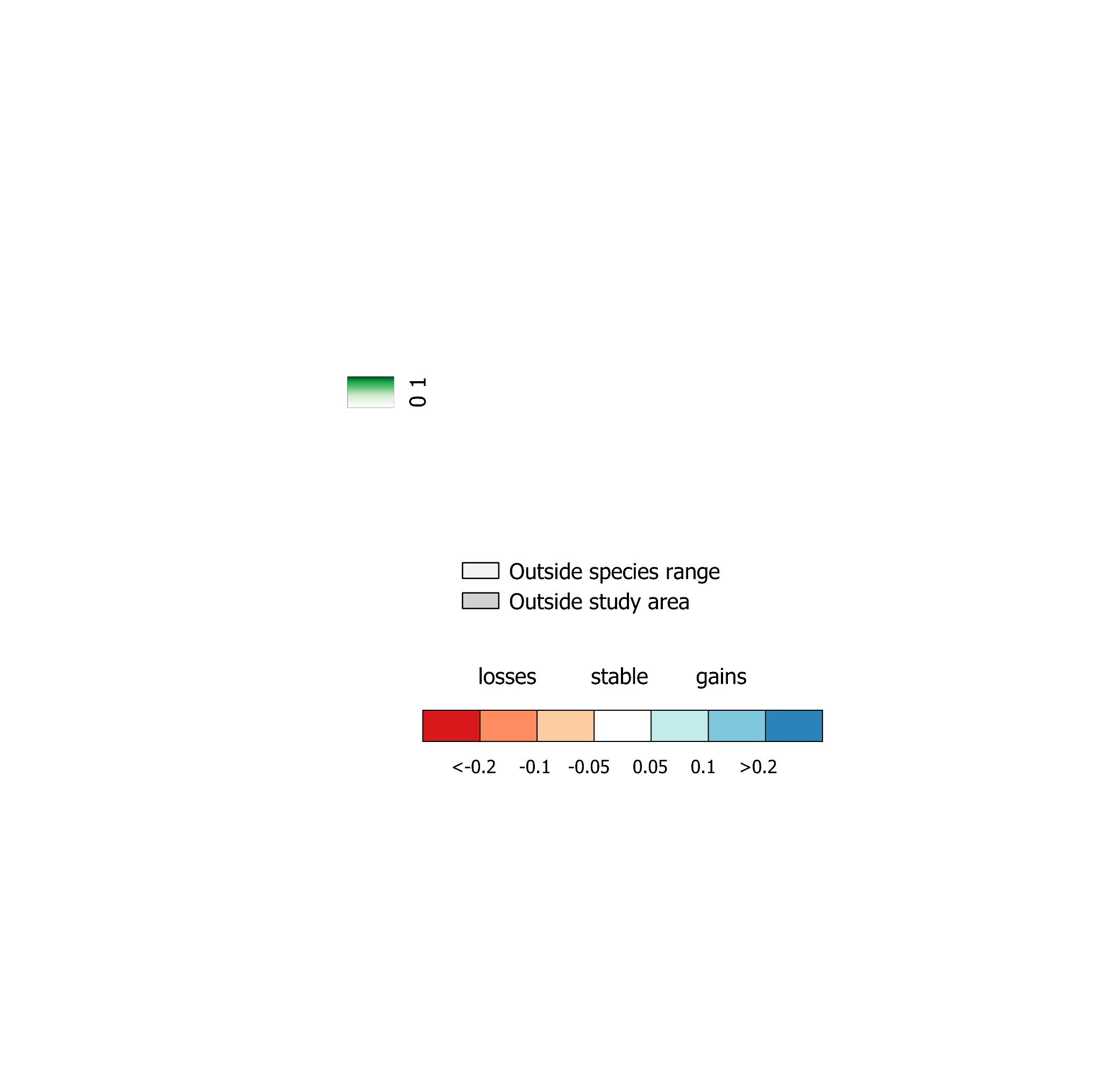  Occurrence probability | |

**Appendix S10. Change in distribution maps.** Changes in distribution maps show, for each 10-km square, the calibrated difference in the probability of occurrence between the second (2018–2022) and the first study period (2013–2017) (Appendix S12). It is crucial to emphasize that data gaps over large areas can significantly impact the spatial performance of models (Johnston et al. 2021; Moudrý et al. 2024; Oliver et al. 2021). We show that small increases in well-distributed data rapidly improve spatial performance at the European scale (Appendix S2), a pattern that also applies to the performance of change maps (Appendix S14).

The quality of the information on distribution substantially varied at the European level, and the lack of monitoring data for the study period in the Southeast generated less accurate predictions on change than those of the rest of Europe (Appendices S6-S8, Appendix S11). Although most change maps closely corresponded to the information and knowledge available at national level, for some species the change diverged substantially from local ornithological knowledge. For example, national data indicates that the Ortolan Bunting (*Emberiza hortulana*) is rapidly declining on farmland areas of Serbia (Radišić 2019), yet our change map did not properly show these patterns.

We explored the relationship between the observed change and the non-calibrated and calibrated change, respectively, for the 43 species whose predicted change maps showed satisfactory performance. For that, we built multiple transformations of the predictor (the observed change) to allow flexible model fitting. Model combinations included three different transformations (linear, polynomial and exponential) for each predictor (non-calibrated and calibrated change). The six models were compared using the Akaike Information Criterion with correction for small sample size (AICc; Burnham and Anderson 2002). The model with the lowest AICc was considered the most parsimonious (i.e., the model that better explained the observed change). Model selection revealed that the model that better explained the observed change was the one including the linear relationship with the calibrated change, followed by the polynomial transformation (AICc weights of the two best models: w_i_ = 0.592 and w_i_ = 0.242; difference in AICc between best and second-best model Δ_i_ < 2). This suggests that calibrated predictions provided greater explanatory power than non-calibrated change maps.

**Appendix S11. Accuracy and bias metrics of change maps.** Accuracy and bias metrics for the 50 study species across the two study periods used to measure the performance of change maps. Accuracy values appear before the slash, and bias values appear after the slash. Both metrics are calculated across the whole species range and at the regional level. Species with low change performance (defined as accuracy below 0.70 or absolute bias above 0.25) or species for which the change performance evaluation did not converge are marked with a cross (✝). SDs from cross-validation are not shown.

| **Scientific name** | **Acc./Bias Europe** | **Acc./Bias Central-Eastern** | **Acc./Bias North** | **Acc./Bias Southeast** | **Acc./Bias Southwest** | **Acc./Bias**  **West** |
| --- | --- | --- | --- | --- | --- | --- |
| *Alauda arvensis* | 0.94 / 0.04 | 0.93 / 0 | 0.88 / 0.09 | 0.90 / -0.01 | 0.93 / 0.02 | 0.94 / 0.04 |
| *Alectoris rufa* | 0.82 / 0.05 | ー | ー | ー | 0.82 / 0.01 | 0.79 / 0.11 |
| *Anthus campestris* | 0.8 / 0.23 | 0.84 / 0.04 | ー | 0.66 / 0.17 | 0.83 / 0.1 | ー |
| *Anthus pratensis* | 0.9 / 0.06 | 0.86 / 0.01 | 0.85 / 0.16 | ー | ー | 0.88 / -0.05 |
| *Athene noctua* | 0.84 / 0.15 | ー | ー | ー | 0.84 / 0.17 | ー |
| *Bubulcus ibis* | 0.88 / 0.04 | ー | ー | ー | 0.88 / 0.04 | ー |
| *Burhinus oedicnemus* | 0.79 / 0.08 | ー | ー | ー | 0.79 / 0.08 | ー |
| *Calandrella brachydactyla* | 0.82 / -0.01 | ー | ー | 0.64 / 0.02 | 0.81 / 0.07 | ー |
| *Ciconia ciconia* | 0.87 / 0.02 | 0.9 / 0.07 | ー | 0.78 / -0.05 | 0.85 / 0.04 | 0.7 / 0.13 |
| *Circus pygargus ✝* | 0.80 / 0.30 | 0.71 / 0.27 | ー | 0.76 / -0.01 | 0.78 / 0.11 | ー |
| *Coracias garrulus* | 0.76 / 0.13 | ー | ー | 0.62 / 0.09 | 0.77 / 0.1 | ー |
| *Corvus frugilegus* | 0.86 / 0.12 | 0.81 / 0.02 | ー | 0.8 / 0.07 | 0.72 / 0.23 | 0.89 / 0.01 |
| *Coturnix coturnix* | 0.83 / 0.12 | 0.77 / 0.09 | ー | 0.62 / 0.16 | 0.84 / 0.09 | 0.72 / 0.12 |
| *Crex crex* | 0.71 / 0.19 | 0.76 / 0.08 | 0.55 / 0.26 | ー | ー | 0.52 / 0.22 |
| *Curruca communis* | 0.92 / 0.06 | 0.88 / -0.07 | 0.88 / -0.06 | 0.82 / 0.03 | 0.91 / 0.07 | 0.87 / 0.1 |
| *Emberiza calandra* | 0.94 / -0.02 | 0.85 / 0.06 | ー | 0.85 / -0.02 | 0.92 / -0.02 | 0.88 / -0.01 |
| *Emberiza cirlus* | 0.9 / -0.01 | ー | ー | ー | 0.9 / -0.02 | ー |
| *Emberiza citrinella* | 0.94 / -0.08 | 0.83 / -0.08 | 0.88 / -0.04 | 0.81 / 0.04 | 0.87 / -0.05 | 0.93 / 0.04 |
| *Emberiza hortulana* | 0.81 / 0.1 | 0.76 / 0.19 | 0.44 / 0.17 | 0.82 / 0.03 | 0.74 / 0.01 | 0.76 / 0.05 |
| *Emberiza melanocephala ✝* | ー | ー | ー | ー | ー | ー |
| *Falco naumanni* | 0.8 / 0.14 | ー | ー | ー | 0.8 / 0.14 | ー |
| *Falco tinnunculus* | 0.83 / 0.03 | 0.79 / 0 | 0.81 / 0.07 | 0.86 / 0 | 0.83 / -0.02 | 0.83 / 0.05 |
| *Galerida cristata* | 0.86 / 0.08 | 0.8 / -0.02 | ー | 0.71 / 0.06 | 0.85 / 0.07 | ー |
| *Galerida theklae* | 0.81 / -0.01 | ー | ー | ー | 0.81 / -0.01 | ー |
| *Hirundo rustica* | 0.83 / -0.13 | 0.79 / -0.01 | 0.78 / -0.12 | 0.78 / -0.05 | 0.78 / -0.15 | 0.83 / -0.08 |
| *Lanius collurio* | 0.9 / 0.16 | 0.75 / 0.1 | 0.84 / 0.12 | 0.82 / -0.07 | 0.89 / 0.07 | 0.83 / 0.15 |
| *Lanius excubitor ✝* | 0.71 / 0.27 | 0.69 / 0.11 | ー | ー | ー | 0.56 / 0.07 |
| *Lanius meridionalis* | 0.77 / 0.22 | ー | ー | ー | 0.77 / 0.22 | ー |
| *Lanius minor* | 0.72 / 0.08 | 0.63 / 0.04 | ー | 0.69 / -0.02 | 0.8 / -0.05 | ー |
| *Lanius senator* | 0.83 / 0.22 | ー | ー | ー | 0.86 / 0.08 | ー |
| *Limosa limosa ✝* | ー | ー | ー | ー | ー | ー |
| *Linaria cannabina* | 0.91 / 0 | 0.9 / 0 | 0.84 / 0.03 | 0.77 / 0.08 | 0.86 / 0.04 | 0.89 / 0.01 |
| *Melanocorypha calandra* | 0.81 / -0.13 | ー | ー | ー | 0.84 / 0.03 | ー |
| *Motacilla flava* | 0.89 / -0.02 | 0.87 / -0.03 | 0.87 / 0.12 | 0.82 / 0.04 | 0.86 / 0 | 0.86 / 0.08 |
| *Oenanthe hispanica* | 0.76 / 0.18 | ー | ー | ー | 0.78 / 0.15 | ー |
| *Passer hispaniolensis* | 0.74 / 0.12 | ー | ー | 0.61 / 0.13 | 0.78 / 0.15 | ー |
| *Passer montanus* | 0.88 / 0.12 | 0.88 / -0.03 | 0.84 / -0.01 | 0.85 / 0.07 | 0.91 / 0.04 | 0.83 / 0.11 |
| *Perdix perdix* | 0.72 / 0.22 | 0.78 / 0.07 | ー | 0.61 / 0.12 | 0.69 / 0.19 | 0.69 / 0.17 |
| *Petronia petronia* | 0.84 / 0.19 | ー | ー | ー | 0.84 / 0.19 | ー |
| *Pterocles alchata ✝* | ー | ー | ー | ー | ー | ー |
| *Pterocles orientalis ✝* | ー | ー | ー | ー | ー | ー |
| *Saxicola rubetra* | 0.86 / 0.11 | 0.88 / 0.11 | 0.84 / 0.07 | 0.75 / -0.16 | 0.65 / 0.13 | 0.78 / 0.18 |
| *Saxicola torquatus* | 0.88 / 0.1 | 0.84 / 0.05 | ー | 0.8 / 0.04 | 0.92 / 0.05 | 0.85 / 0.11 |
| *Serinus serinus* | 0.85 / 0.07 | 0.86 / 0.06 | ー | 0.76 / 0.11 | 0.82 / -0.08 | 0.8 / 0.2 |
| *Streptopelia turtur* | 0.87 / -0.1 | 0.82 / -0.03 | ー | 0.78 / 0.03 | 0.9 / -0.07 | 0.65 / 0.06 |
| *Sturnus unicolor ✝* | 0.77 / -0.37 | ー | ー | ー | 0.77 / -0.37 | ー |
| *Sturnus vulgaris* | 0.9 / -0.03 | 0.83 / -0.03 | 0.83 / 0.03 | 0.71 / -0.09 | 0.93 / -0.01 | 0.88 / 0 |
| *Tetrax tetrax* | 0.78 / 0.24 | ー | ー | ー | 0.78 / 0.24 | ー |
| *Upupa epops* | 0.88 / 0.08 | 0.89 / -0.03 | ー | 0.72 / 0.11 | 0.88 / -0.02 | 0.7 / 0.13 |
| *Vanellus vanellus* | 0.86 / 0.02 | 0.87 / 0.09 | 0.84 / -0.04 | 0.85 / 0 | 0.72 / 0.08 | 0.88 / 0.05 |
| **Mean** | **0.83 / 0.08** | **0.82 / 0.04** | **0.79 / 0.06** | **0.76 / 0.03** | **0.83 / 0.05** | **0.80 / 0.08** |

**Appendix S12. Change maps.** Change in distribution maps for the 43 farmland bird species with satisfactory change performance included in this study, calculated as the calibrated difference in the probability of occurrence between the second (2018–2022) and the first period (2013–2017). Negative values (red) indicate a reduction in distribution, grey indicates no change, and positive values (blue) indicate an increase in distribution. See performance statistics in Appendix S11.

| *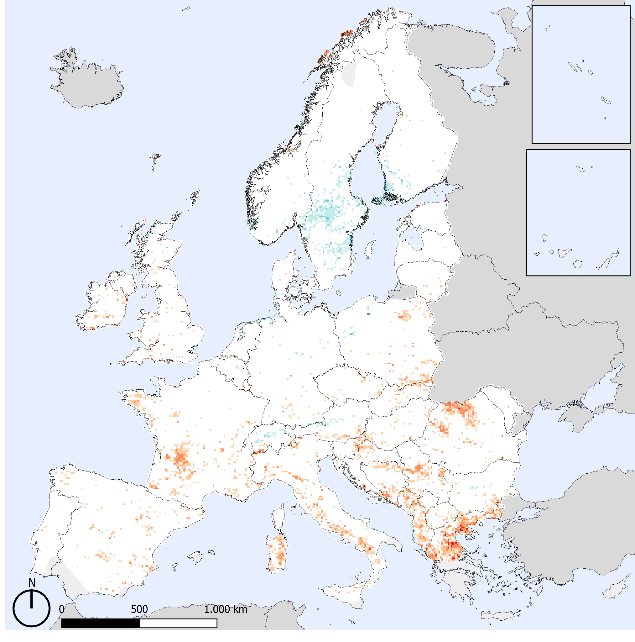* | *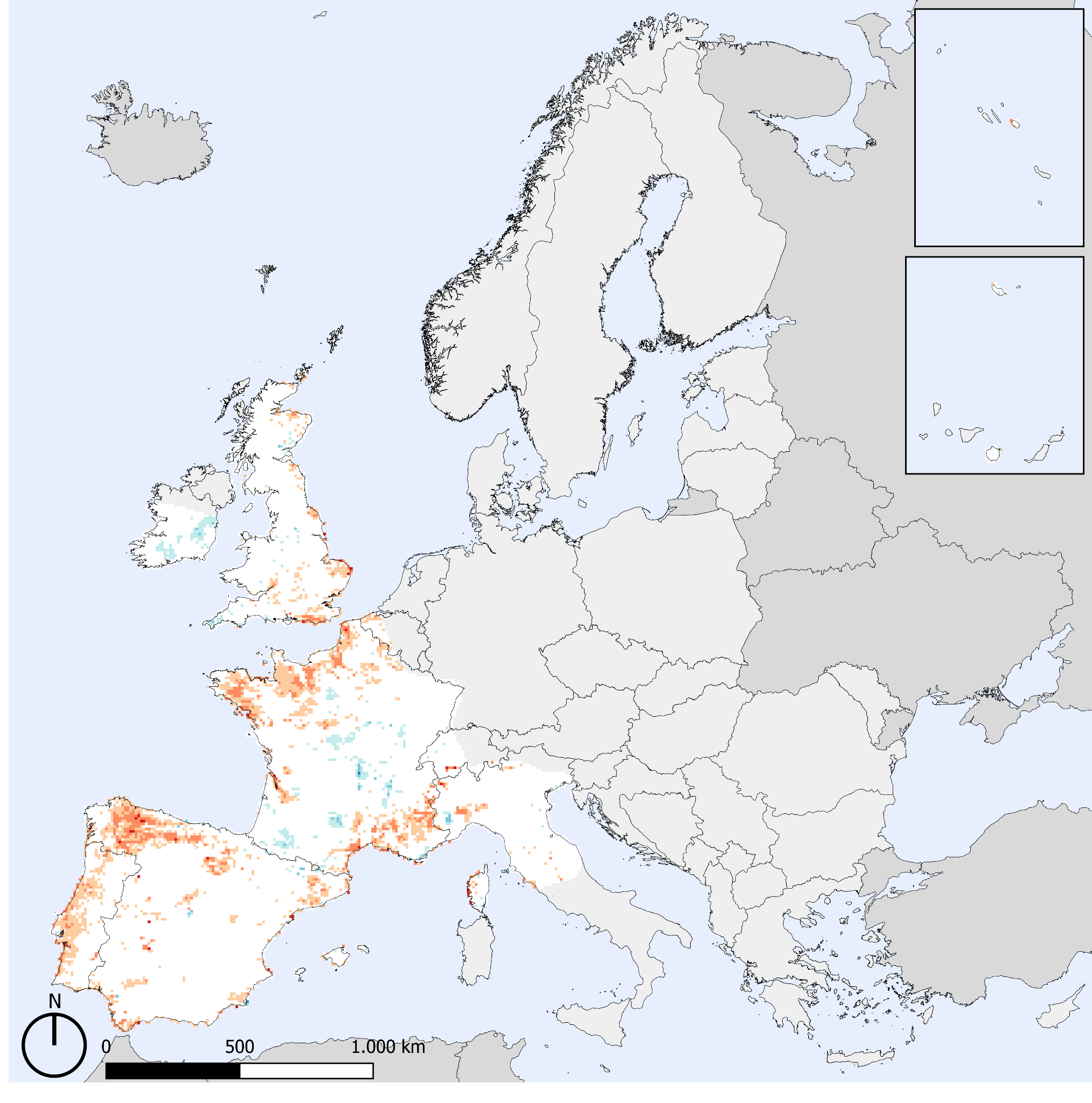* |
| --- | --- |
| *Alauda arvensis* | *Alectoris rufa* |
| *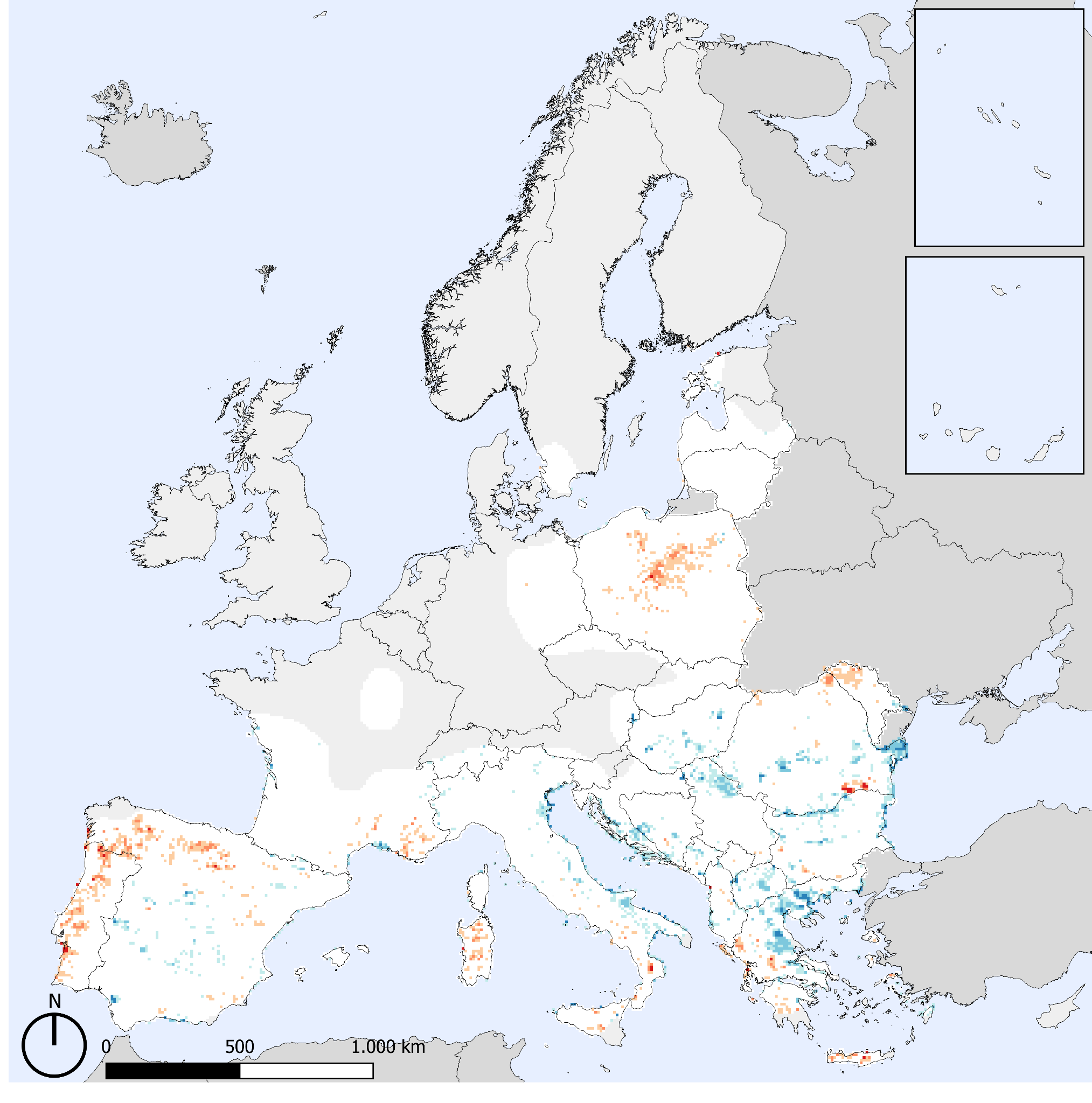* | *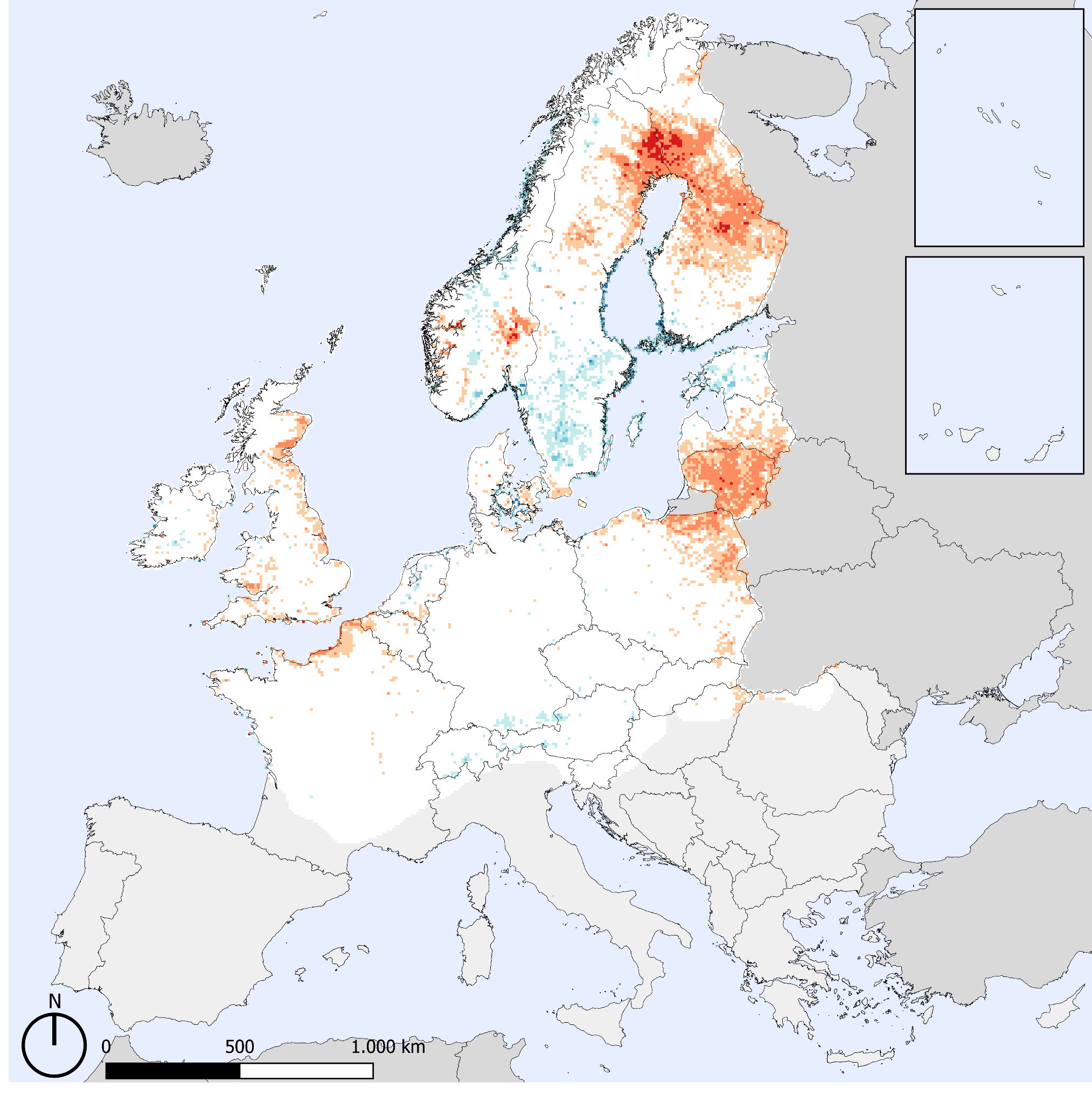* |
| *Anthus campestris* | *Anthus pratensis* |
| **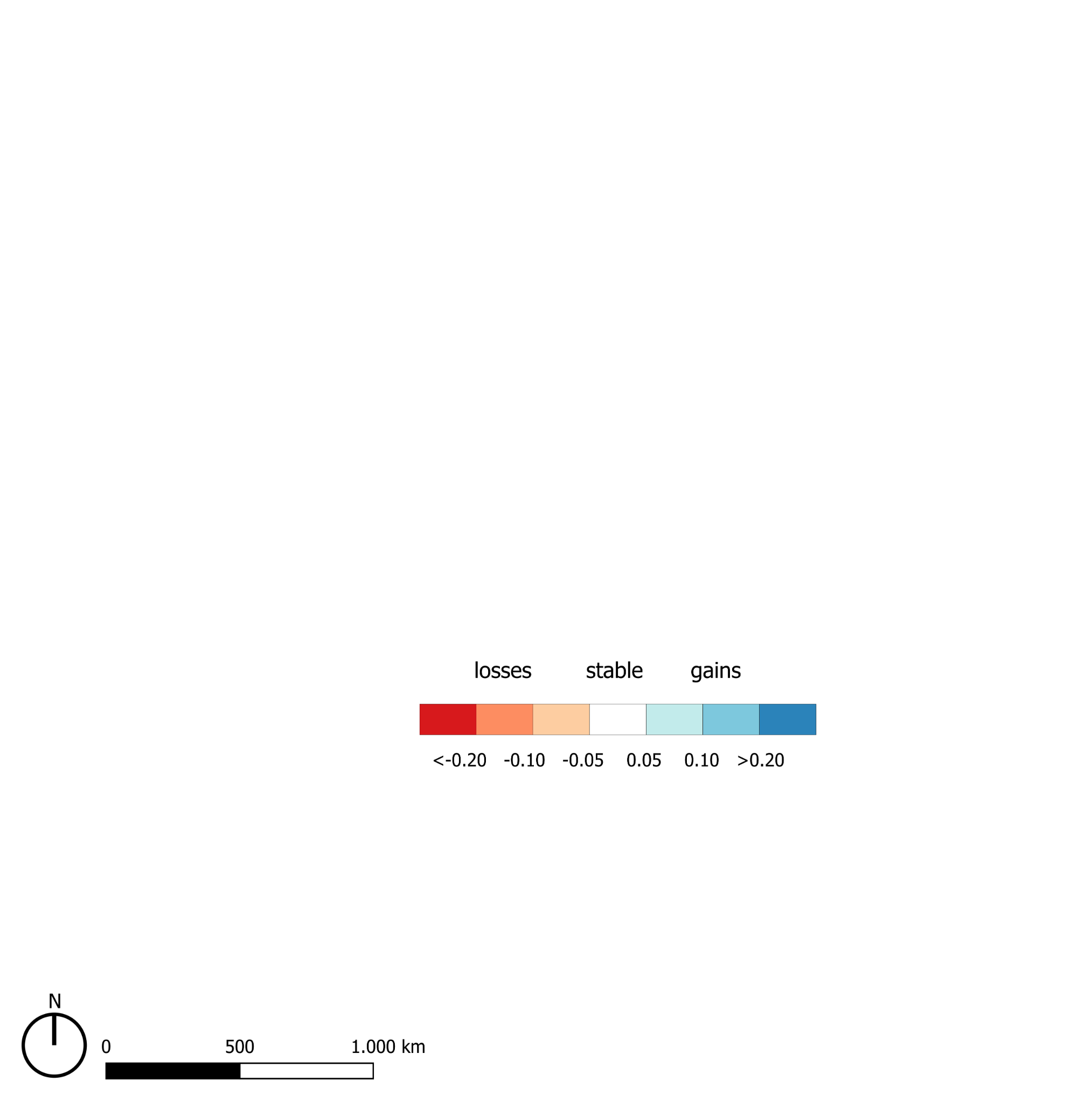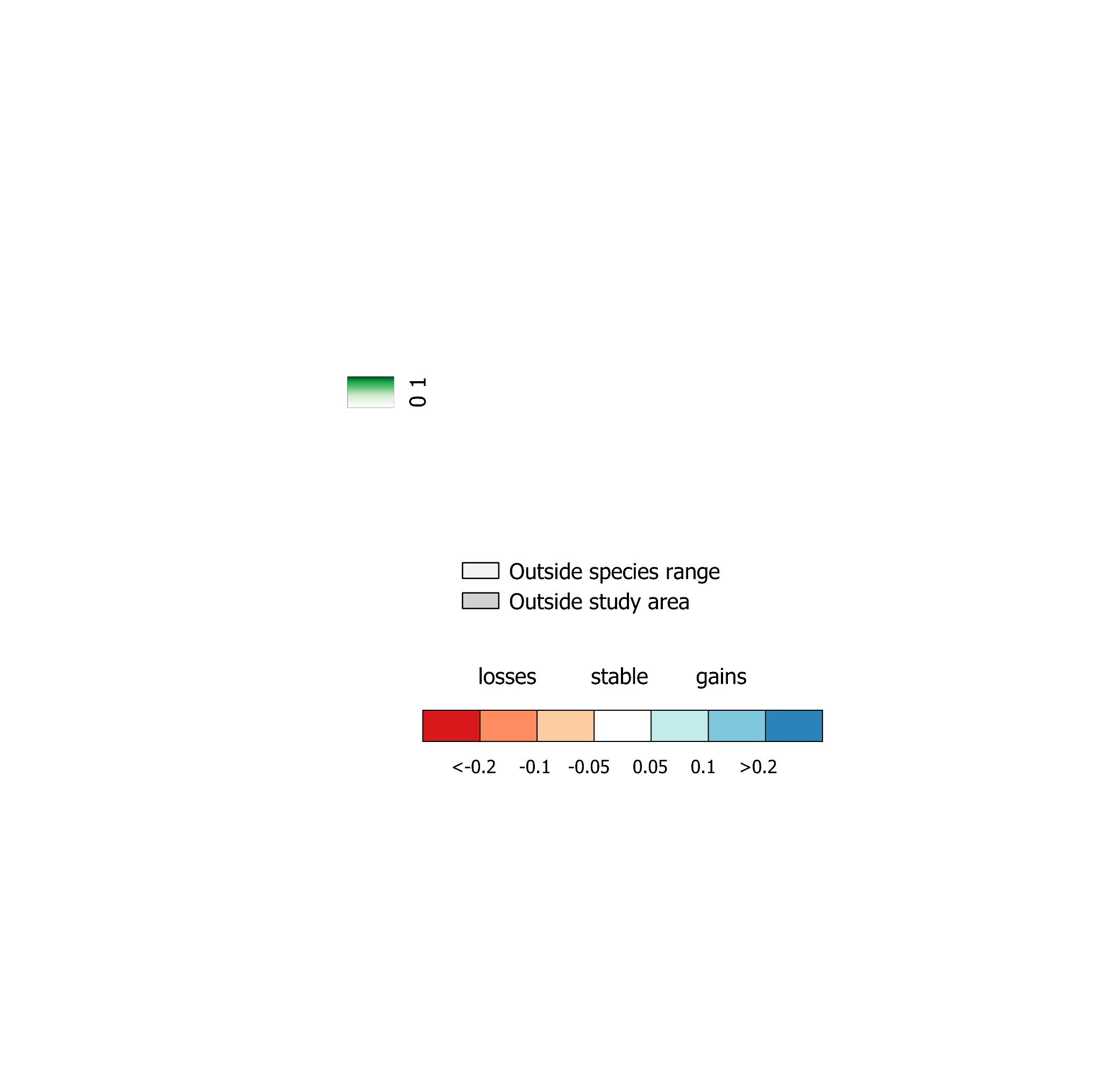**  Change in occurrence probability | |

| *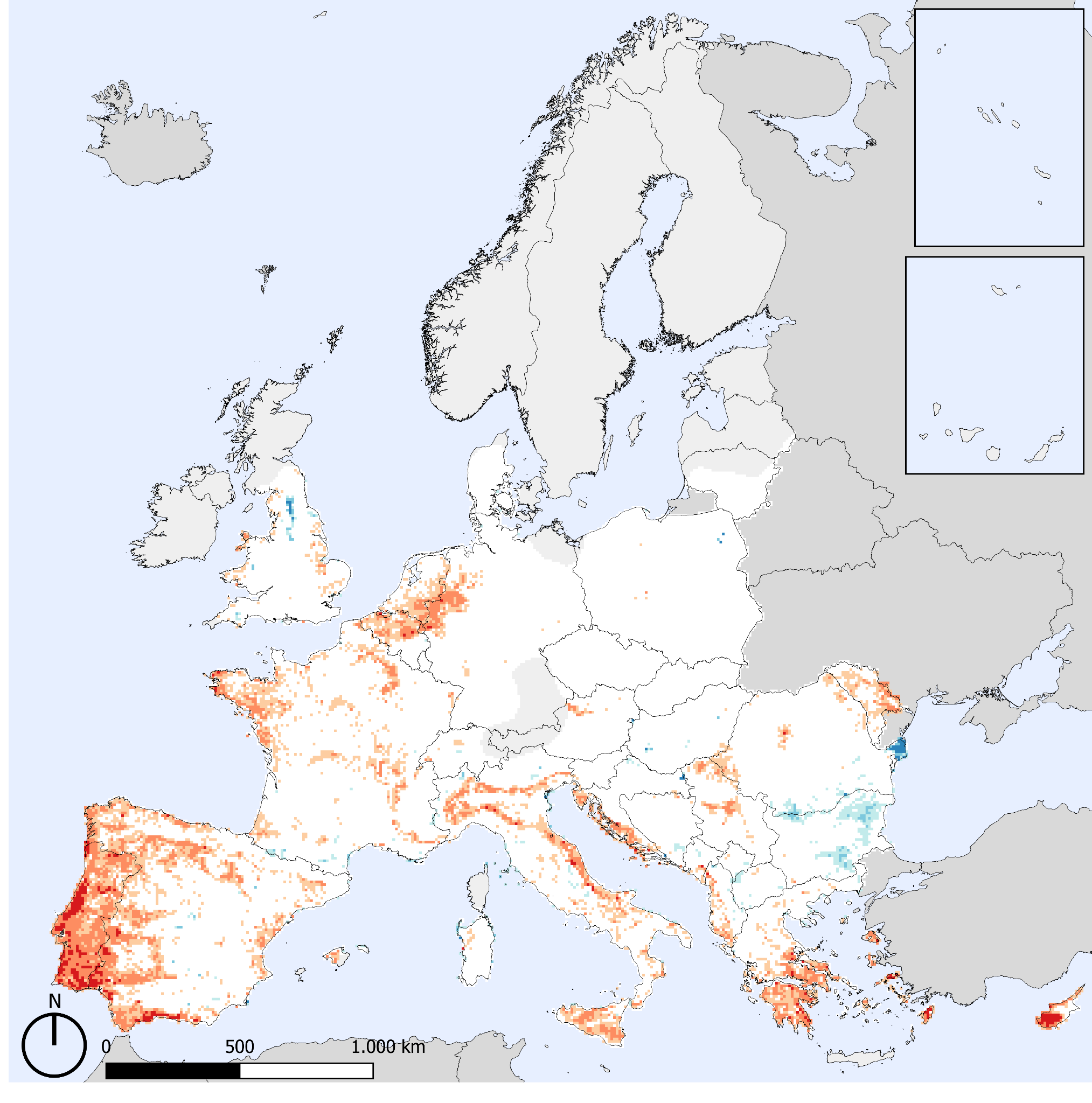* | *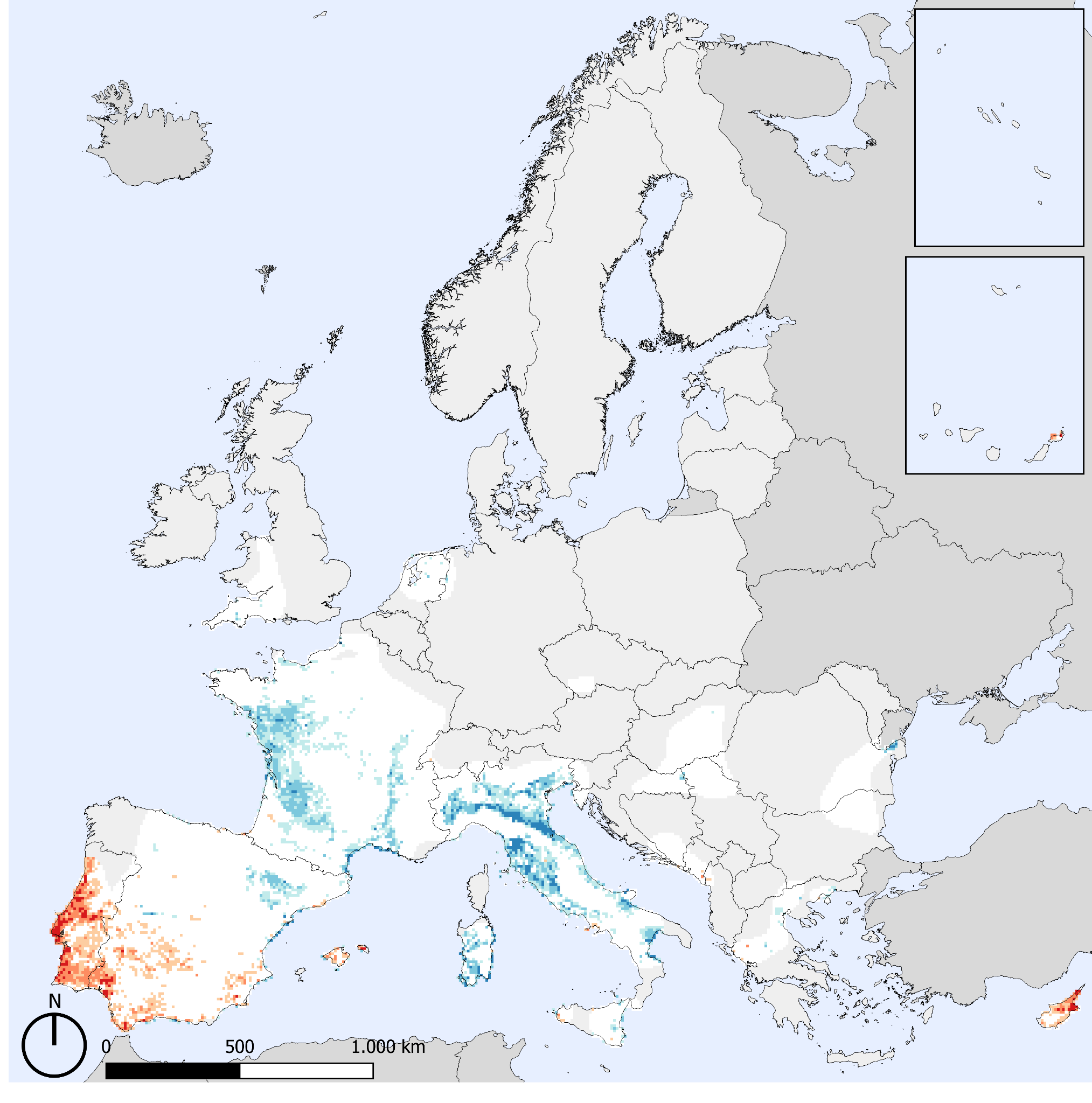* |
| --- | --- |
| *Athene noctua* | *Bubulcus ibis* |
| *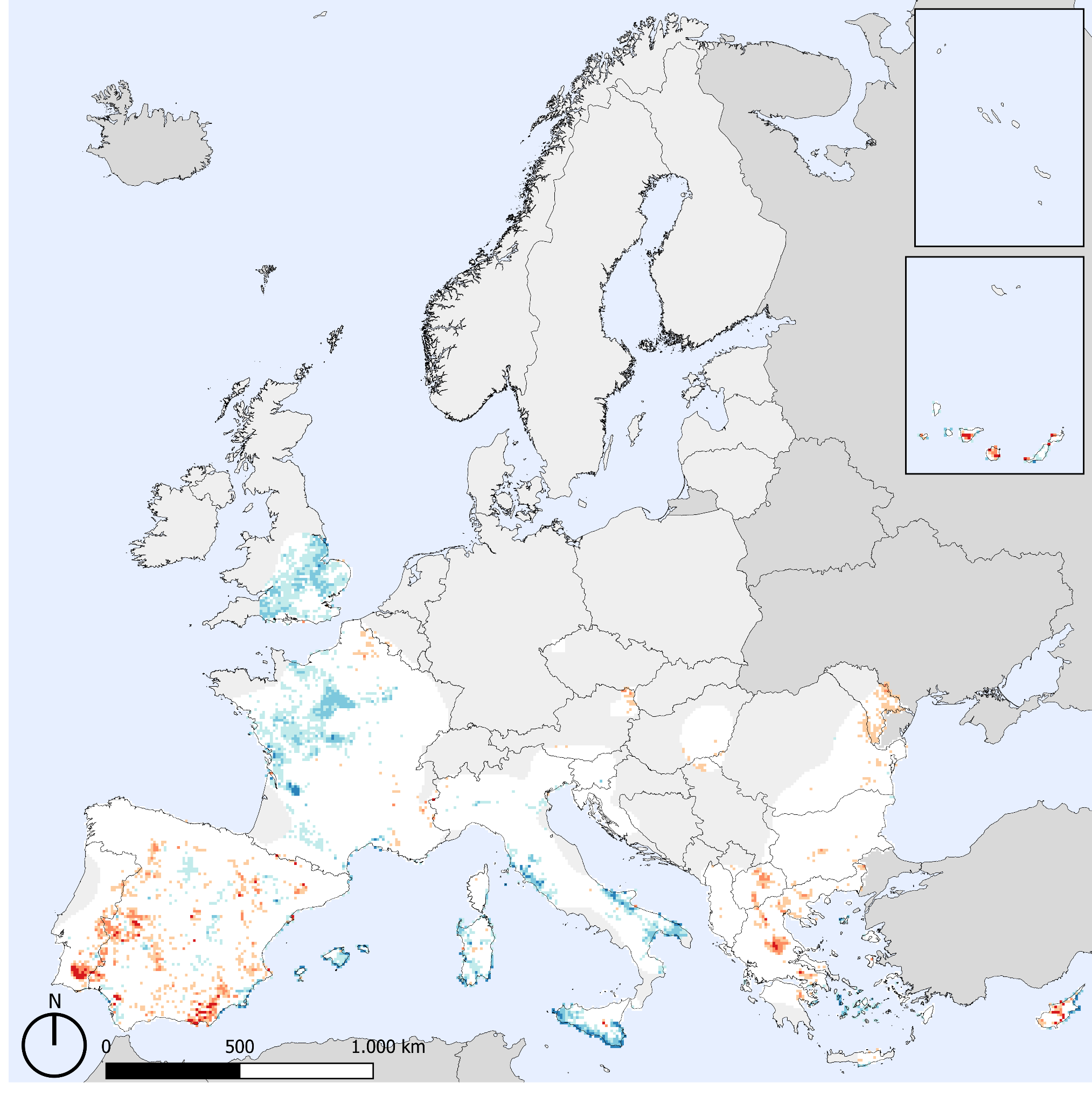* | *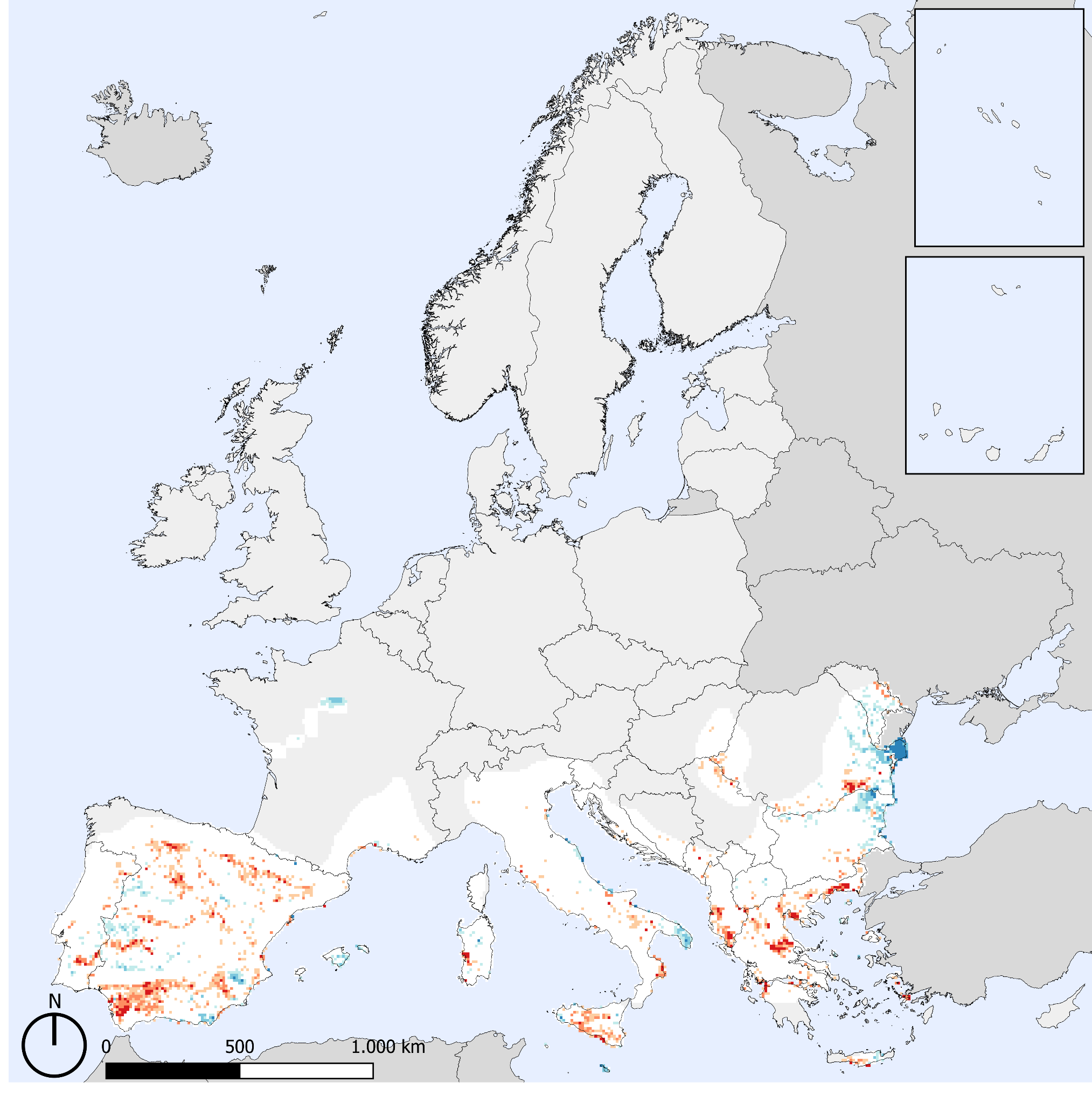* |
| *Burhinus oedicnemus* | *Calandrella brachydactyla* |
| **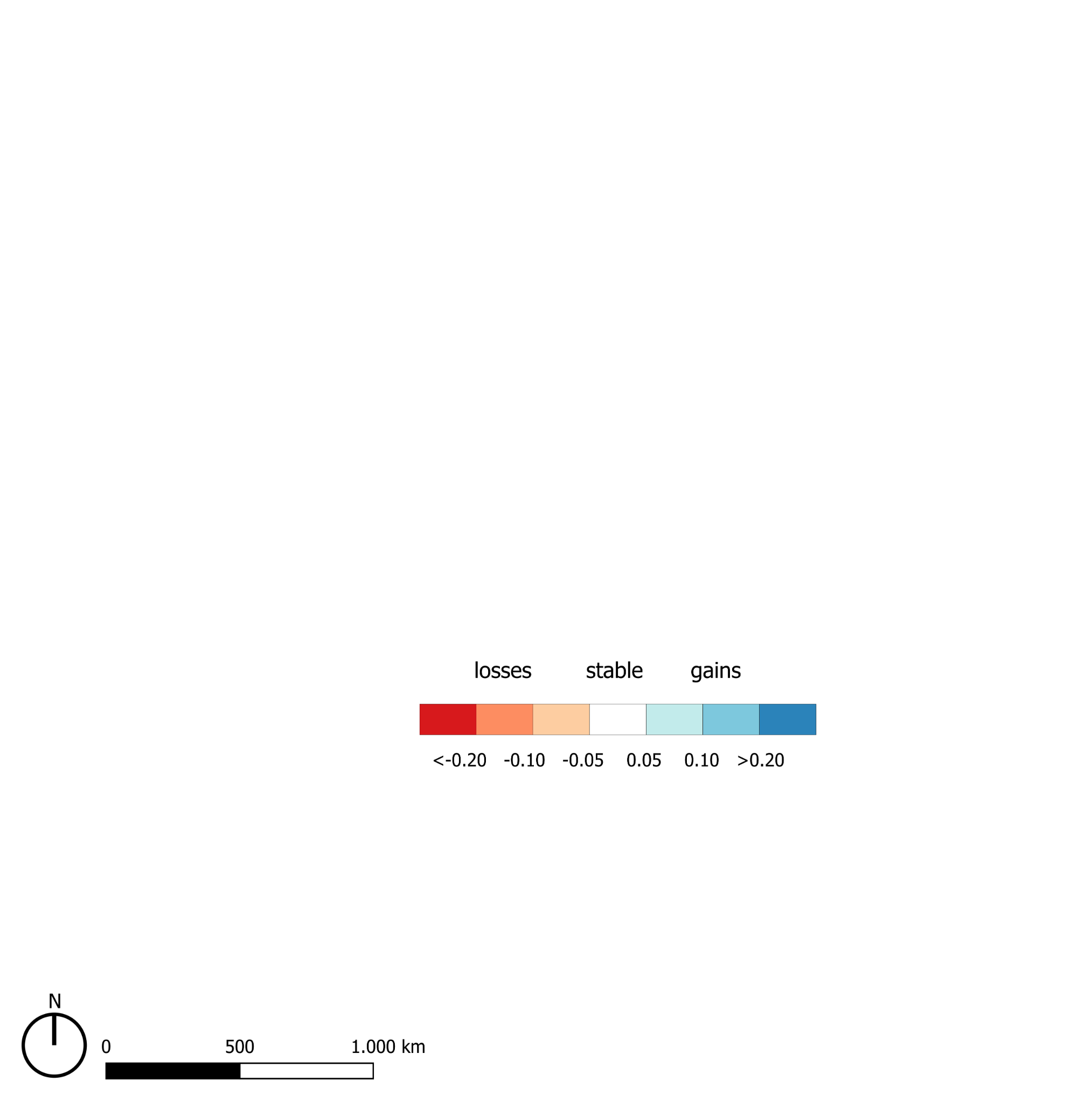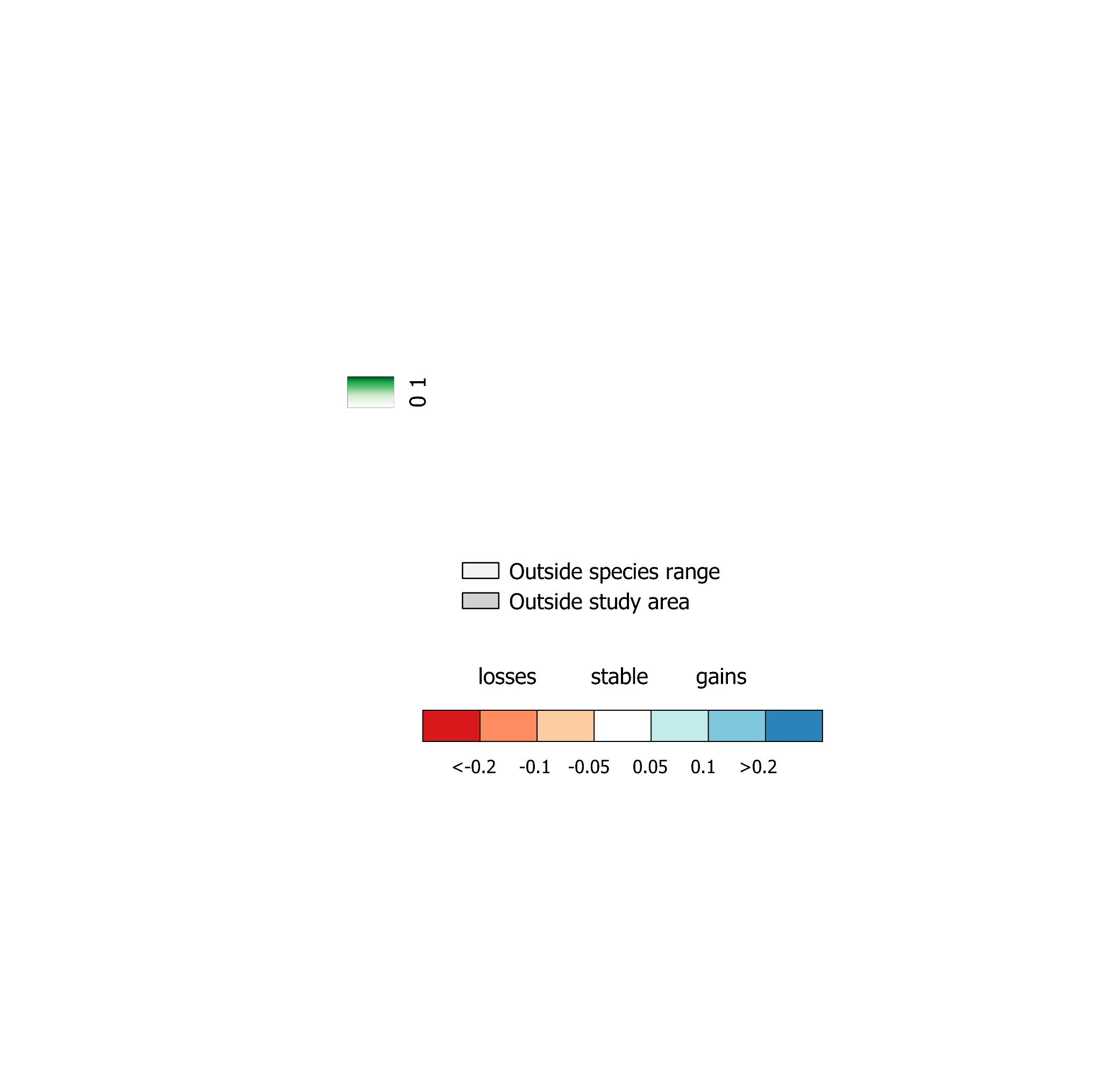**  Change in occurrence probability | |
| *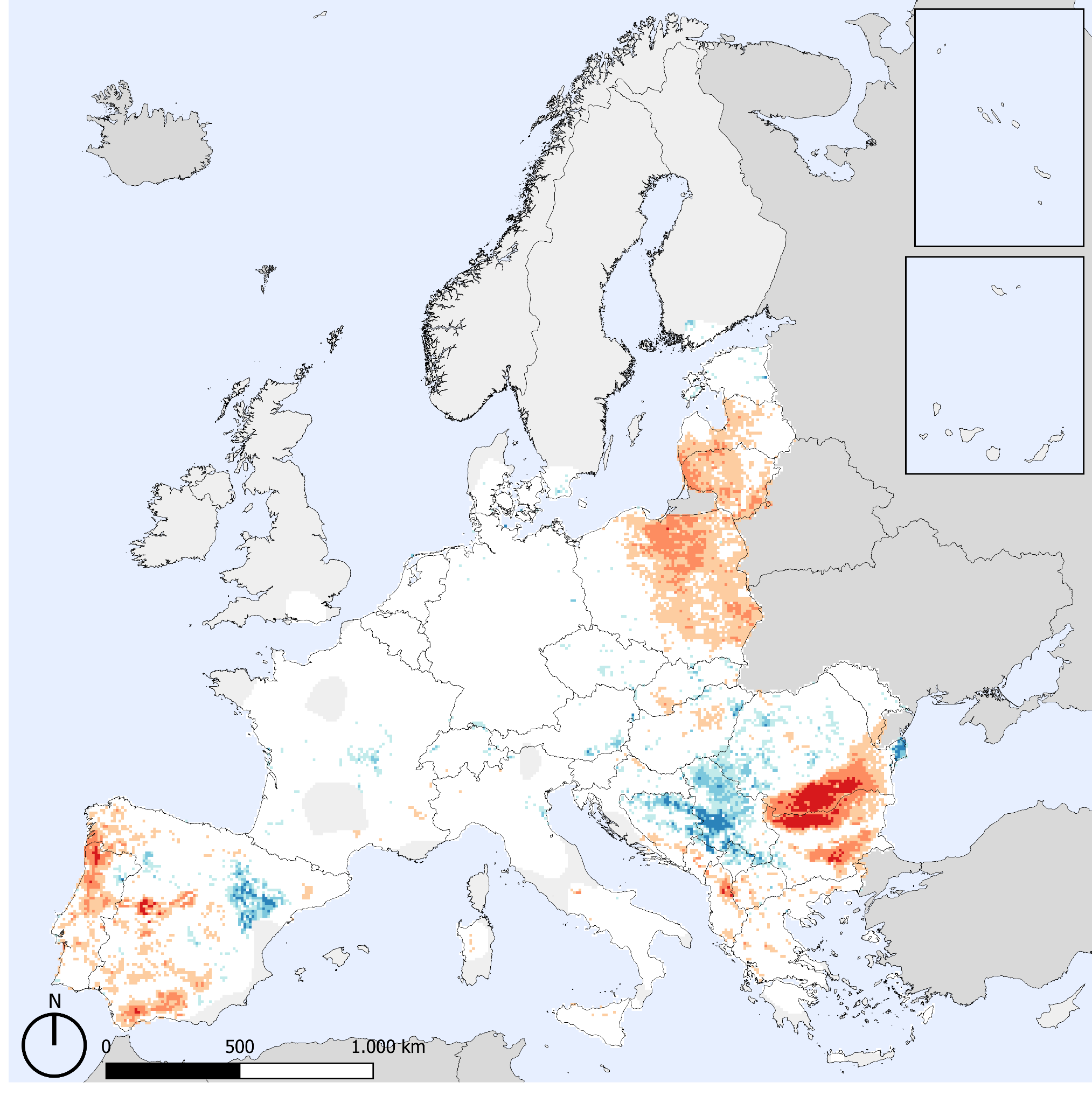* | *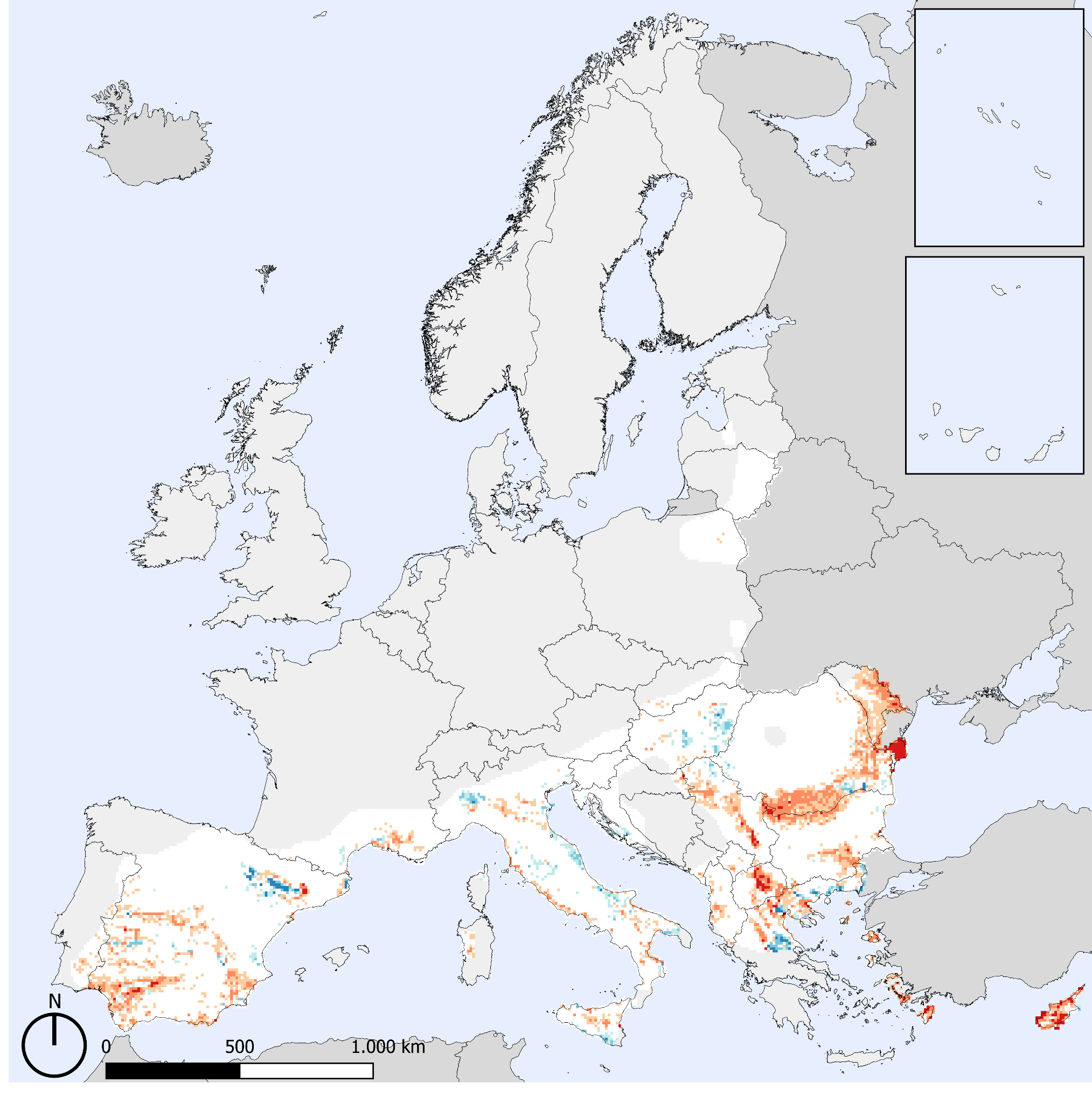* |
| *Ciconia ciconia* | *Coracias garrulus* |
| *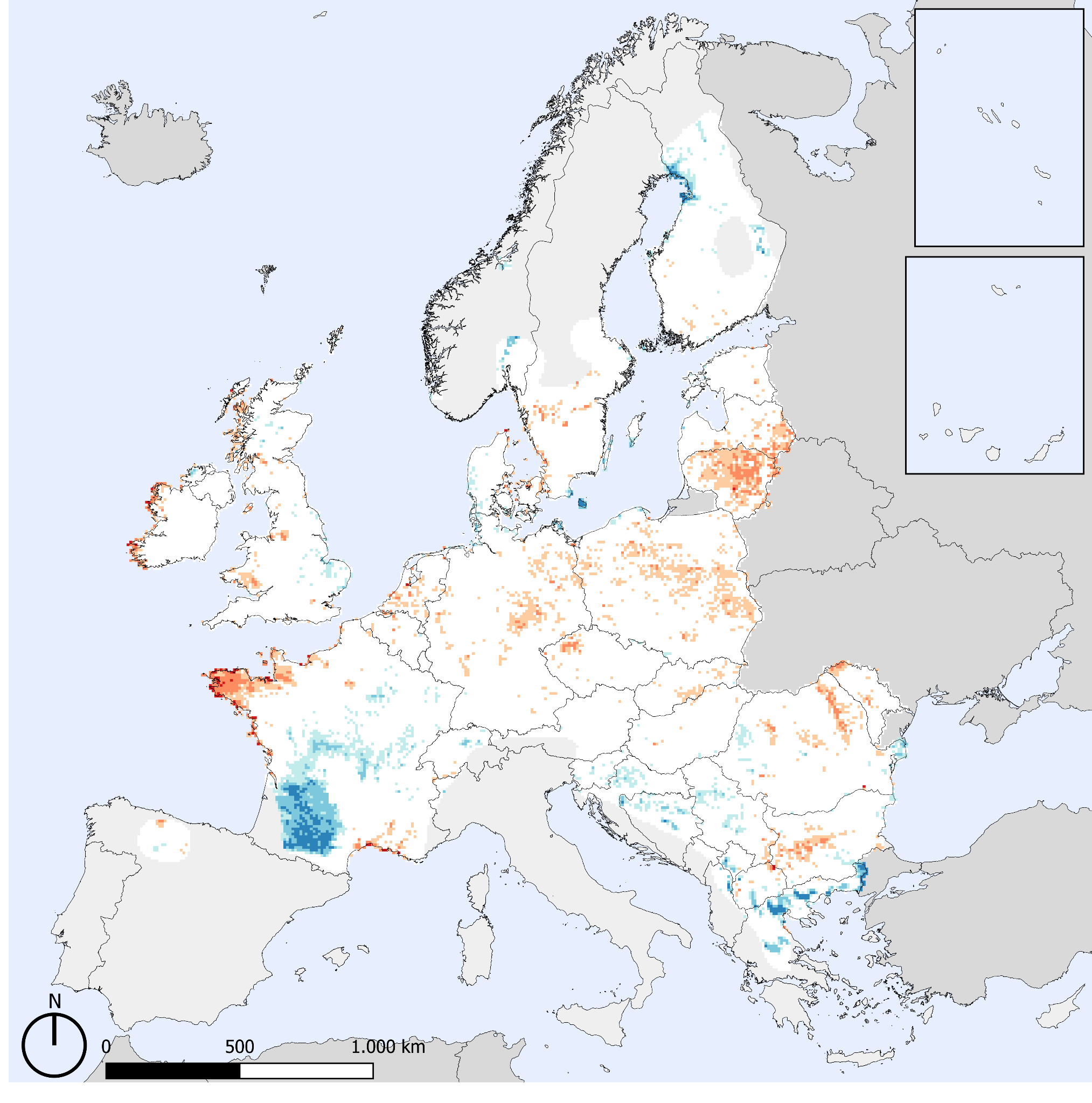* | *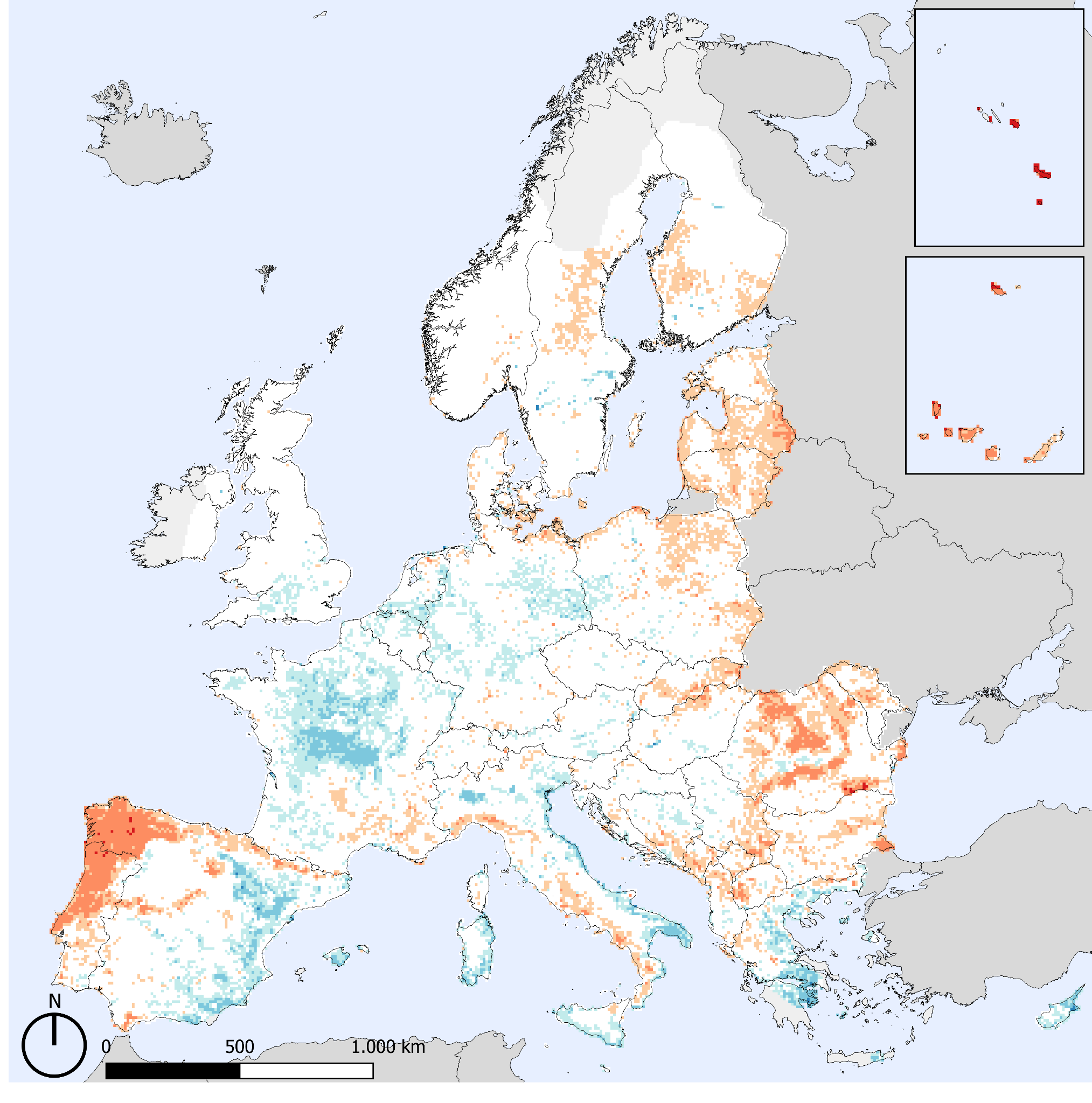* |
| *Corvus frugilegus* | *Coturnix coturnix* |
| **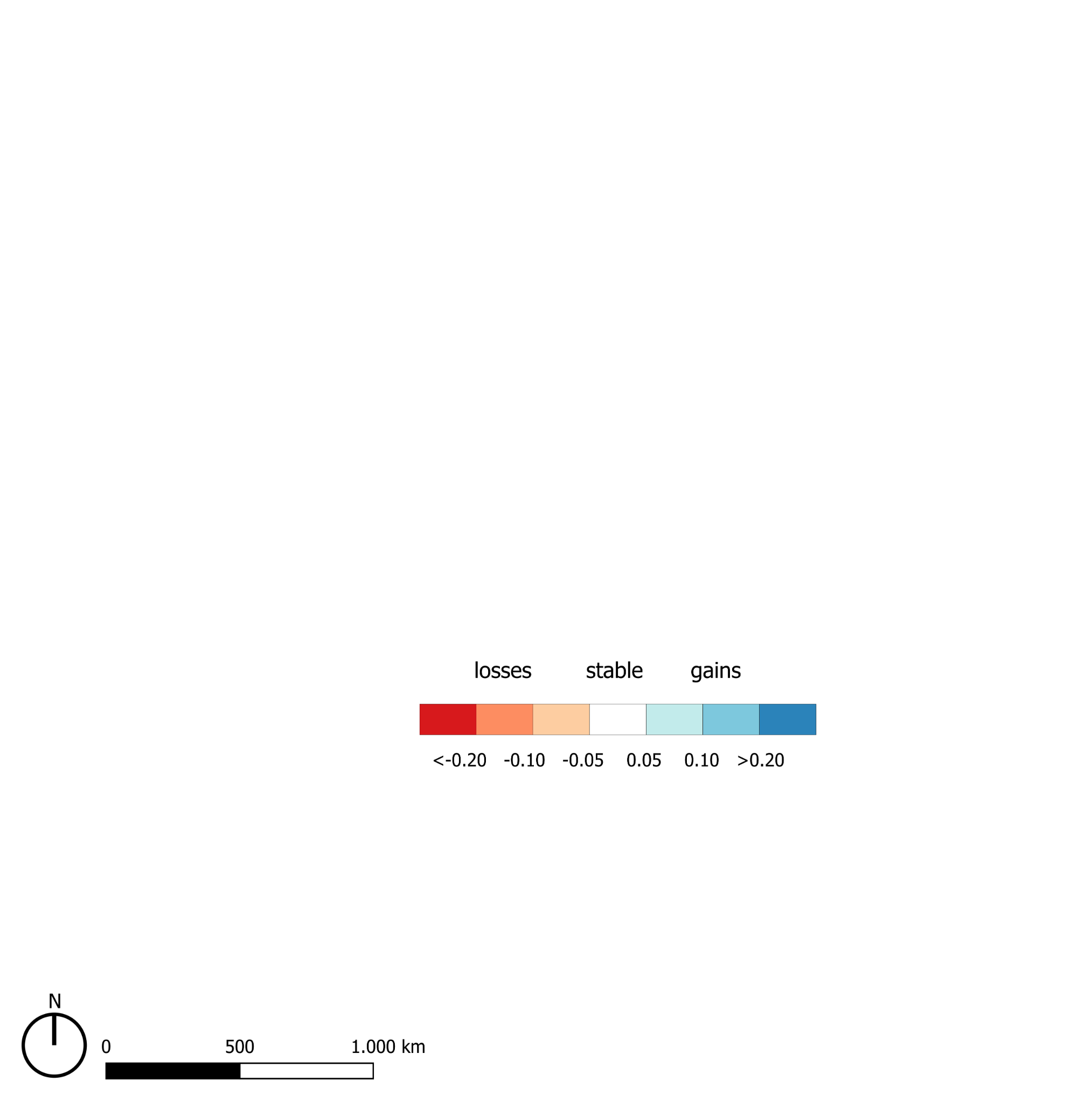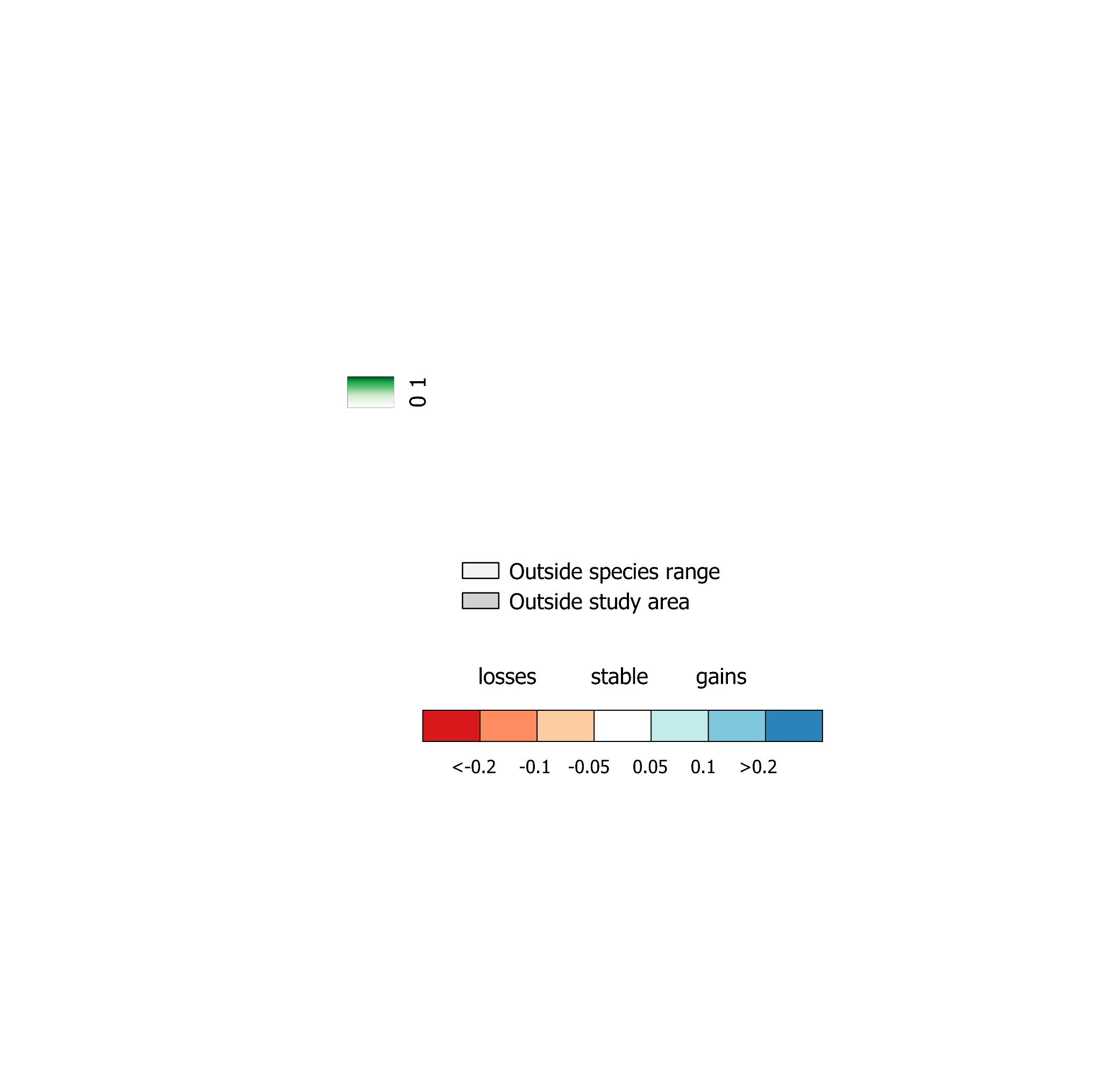**  Change in occurrence probability | |
| *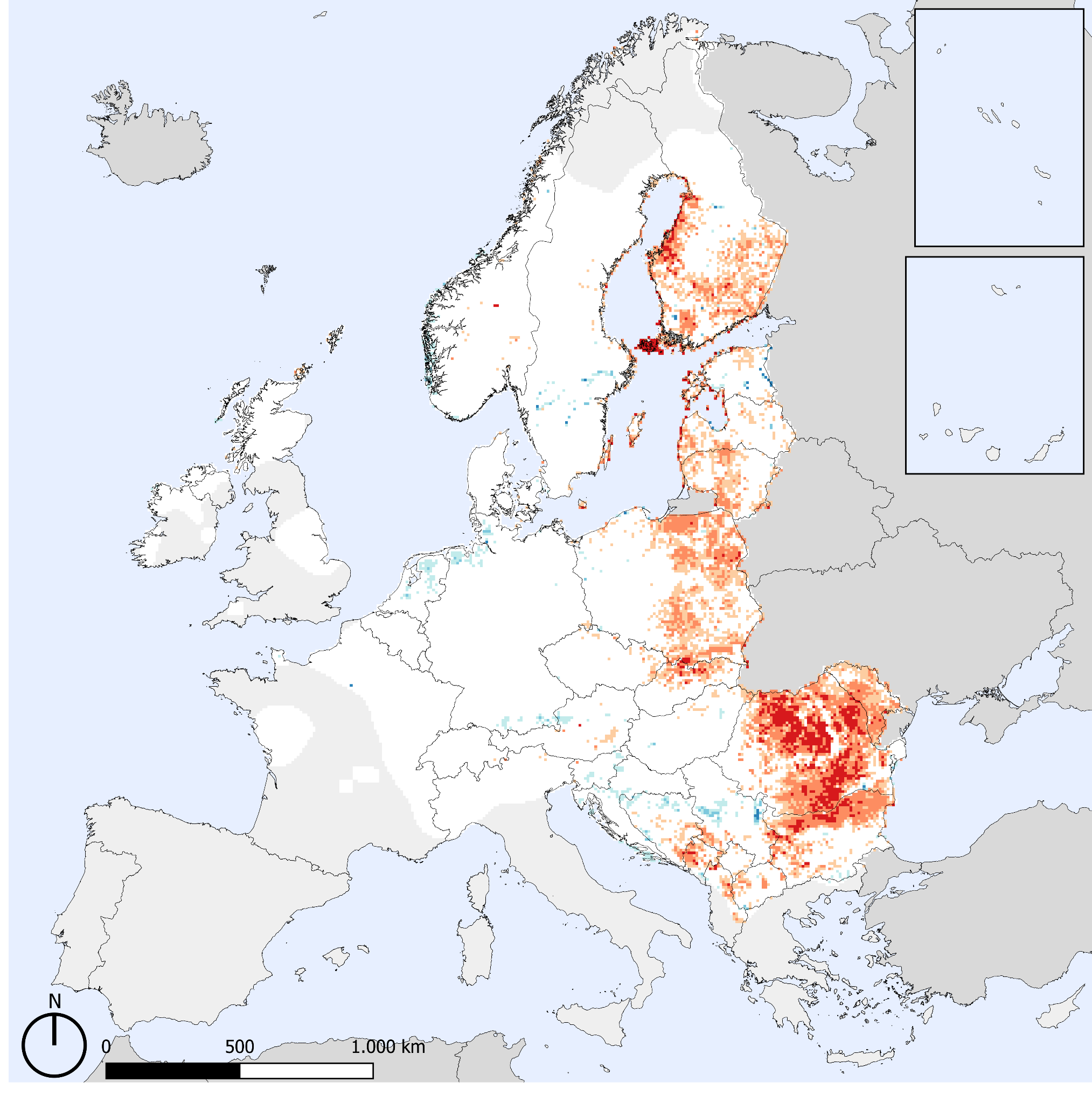* | *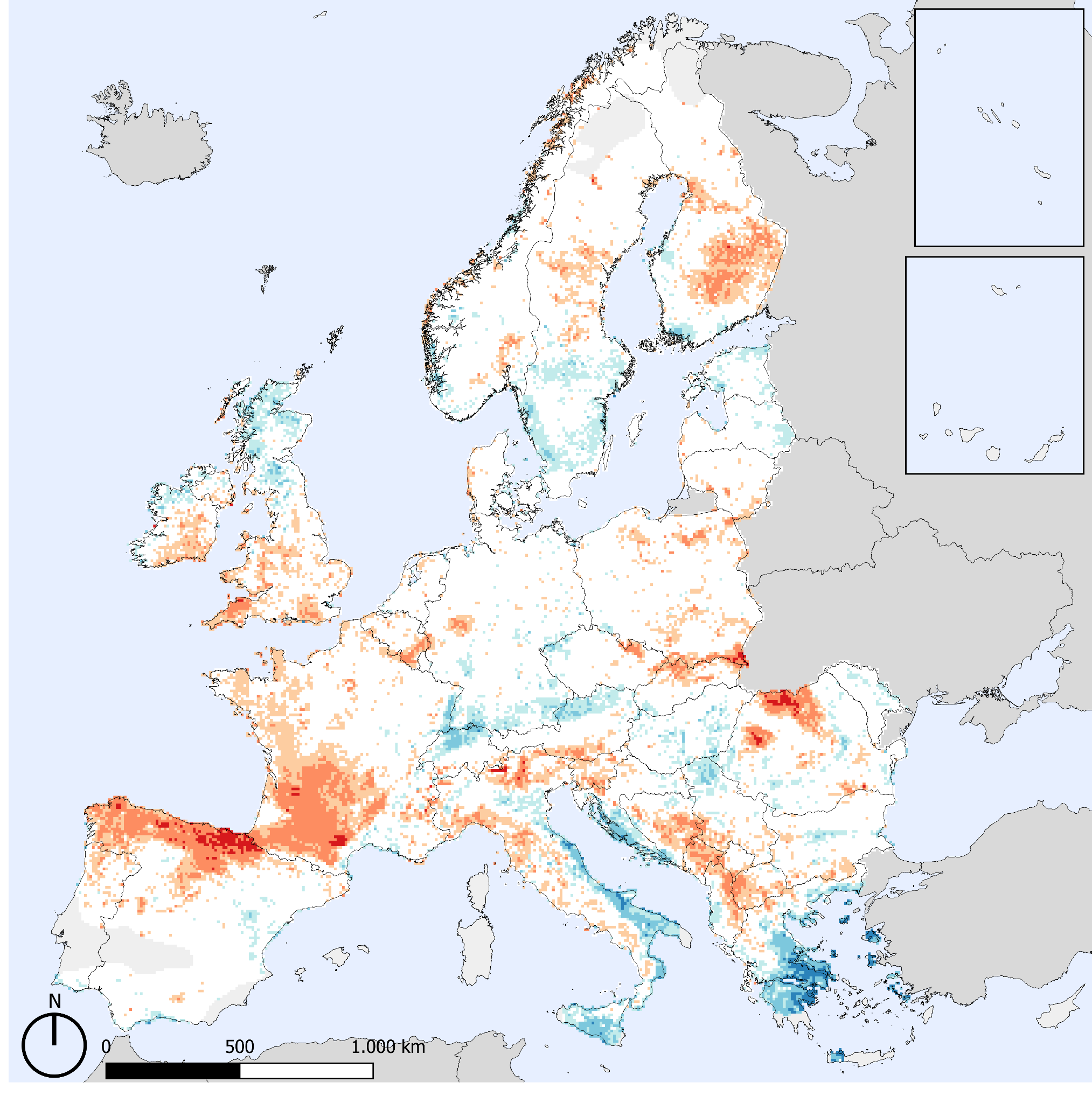* |
| *Crex crex* | *Curruca communis* |
|  |  |
| *Emberiza calandra* | *Emberiza cirlus* |
| Change in occurrence probability | |
|  |  |
| *Emberiza citrinella* | *Emberiza hortulana* |
|  |  |
| *Falco naumanni* | *Falco tinnunculus* |
| Change in occurrence probability | |
|  |  |
| *Galerida cristata* | *Galerida theklae* |
|  |  |
| *Hirundo rustica* | *Lanius collurio* |
| Change in occurrence probability | |
|  |  |
| *Lanius meridionalis* | *Lanius minor* |
|  |  |
| *Lanius senator* | *Linaria cannabina* |
| Change in occurrence probability | |
|  |  |
| *Melanocorypha calandra* | *Motacilla flava* |
|  |  |
| *Oenanthe hispanica* | *Passer hispaniolensis* |
| Change in occurrence probability | |
|  |  |
| *Passer montanus* | *Perdix perdix* |
|  |  |
| *Petronia petronia* | *Saxicola rubetra* |
| Change in occurrence probability | |
|  |  |
| *Saxicola torquatus* | *Serinus serinus* |
|  |  |
| *Streptopelia turtur* | *Sturnus vulgaris* |
| Change in occurrence probability | |

|  |  |
| --- | --- |
| *Tetrax tetrax* | *Upupa epops* |
|  |  |
| *Vanellus vanellus* |  |
| Change in occurrence probability | |

**Appendix S13. Observed change vs predicted change.** Relationship between observed change and calibrated predicted change in distribution for a total of 43 species whose predicted change maps showed satisfactory performance.

**Appendix S14. Performance on sample size.** Left: Change performance for accuracy for each species as a function of the mean sample size of test data. Open dots represent the mean, and black lines show the standard deviation from cross-validation. Pseudo-R² = 0.50; deviance = 0.09. Right: Change performance for the SD of the bias metric for each species from cross-validation, in relation to the mean sample size of test data. Each dot represents a species for which performance could be evaluated (n = 47). Pseudo-R² = 0.38; deviance = 0.13.

**Appendix S15. Percentage of species distribution in farmland habitats.** To assist interpretation of the predicted distributions and changes of farmland species, we calculated the percentage of species distribution occurring in farmland habitats, extracted from the binary farmland map (Appendix S16). Some species occur primarily in farmland, while others also use non-farmland habitats. Higher percentages indicate a greater overlap between the predicted distribution and farmland, whereas lower percentages suggest that non-farmland habitats also contribute. This analysis was conducted at the scale of the entire study area, rather than by region.

| **Scientific name** | **Percentage of distribution in farmland (%)** | **Scientific name** | **Percentage of distribution in farmland (%)** |
| --- | --- | --- | --- |
| *Alauda arvensis* | 71 | *Lanius collurio* | 53 |
| *Alectoris rufa* | 73 | *Lanius excubitor* | 44 |
| *Anthus campestris* | 65 | *Lanius meridionalis* | 84 |
| *Anthus pratensis* | 24 | *Lanius minor* | 78 |
| *Athene noctua* | 82 | *Lanius senator* | 60 |
| *Bubulcus ibis* | 81 | *Limosa limosa* | 78 |
| *Burhinus oedicnemus* | 88 | *Linaria cannabina* | 66 |
| *Calandrella brachydactyla* | 94 | *Melanocorypha calandra* | 96 |
| *Ciconia ciconia* | 79 | *Motacilla flava* | 66 |
| *Circus pygargus* | 90 | *Oenanthe hispanica* | 53 |
| *Coracias garrulus* | 96 | *Passer hispaniolensis* | 63 |
| *Corvus frugilegus* | 88 | *Passer montanus* | 62 |
| *Coturnix coturnix* | 87 | *Perdix perdix* | 99 |
| *Crex crex* | 43 | *Petronia petronia* | 73 |
| *Curruca communis* | 60 | *Pterocles alchata* | 96 |
| *Emberiza calandra* | 71 | *Pterocles orientalis* | 91 |
| *Emberiza cirlus* | 54 | *Saxicola rubetra* | 36 |
| *Emberiza citrinella* | 52 | *Saxicola torquatus* | 59 |
| *Emberiza hortulana* | 78 | *Serinus serinus* | 55 |
| *Emberiza melanocephala* | 61 | *Streptopelia turtur* | 66 |
| *Falco naumanni* | 91 | *Sturnus unicolor* | 58 |
| *Falco tinnunculus* | 61 | *Sturnus vulgaris* | 68 |
| *Galerida cristata* | 73 | *Tetrax tetrax* | 99 |
| *Galerida theklae* | 78 | *Upupa epops* | 64 |
| *Hirundo rustica* | 60 | *Vanellus vanellus* | 62 |

**Appendix S16. Map of farmland areas.** Yellow indicates 10-km squares with at least 50% of their area covered by farmland between 2018 and 2022. Farmland was defined using the following ESA CCI Land Cover categories: rainfed cropland, herbaceous cover, rainfed tree crops, irrigated crops, mosaic cropland–natural vegetation, and grassland (Appendix S4). Non-farmland squares within the study area are shown in white, and areas outside the study area are shown in grey.

**Appendix S17. List of participating countries and ornithological organizations.** Participating countries and ornithological organizations that provided data for the analyses presented in this study.

| **Country** | **Organization Name** |
| --- | --- |
| Andorra | Andorra Research + innovation |
| Austria | BirdLife Austria |
| Belgium | AVES/Natagora |
|  | Research Institute Nature and Forest |
| Bosnia and Herzegovina | Naše Ptice and Society for Research and Protection of Biodiversity |
| Bulgaria | Bulgarian Society for the Protection of Birds |
| Croatia | Ministry of Energy and Sustainable Development, Institute for Environmental and Nature Protection |
|  | Croatian Society for Protection of Birds and Nature |
| Cyprus | BirdLife Cyprus |
| Czechia | Institute of Environmental Studies, Faculty of Science, Charles University |
|  | Czech University of Life Sciences, Faculty of Environmental Sciences, Prague |
|  | Czech Society for Ornithology |
| Denmark | Danish Ornithological Association (BirdLife Denmark) |
| Estonia | Estonian Ornithological Society / BirdLife Estonia |
| Finland | Zoological Museum, Finnish Museum of Natural History |
| France | National Natural History Museum |
|  | Ligue pour la protection des oiseaux |
| Germany | Federation of German Avifaunists |
| Greece | Hellenic Ornithological Society |
| Hungary | University of Nyiregyhaza |
|  | Hungarian Ornithological and Nature Conservation Society (BirdLife Hungary) |
| Ireland | BirdWatch Ireland |
| Italy | Italian League for Bird Protection |
| Kosovo  Latvia | Kosovo Ornithological Society  Latvian Ornithological Society |
|  | Department of Zoology and Animal Ecology, Faculty of Biology, University of Latvia |
| Liechtenstein | Liechtenstein Botanical-Zoological Society |
| Lithuania | Lithuanian Ornithological Society |
| Luxembourg | Luxembourg Ornithological Center |
| Moldova | Society for Birds and Nature Protection |
| Montenegro | Center for Protection and Research of Birds |
| Netherlands | Sovon, Dutch Center for Field Ornithology |
| North Macedonia | Macedonian Ecological Society |
| Norway | Norwegian Institute for Nature Research |
|  | Norwegian Ornithological Society / BirdLife Norway |
| Poland | Museum and Institute of Zoology, Polish Academy of Sciences |
|  | Polish Society for the Protection of Birds (OTOP BirdLife Poland) |
| Portugal | University of Évora |
|  | Portuguese Society for the Study of Birds |
| Romania | Romanian Ornithological Society |
| Serbia | University of Novi Sad, Faculty of Sciences, Department of Biology and Ecology |
|  | Bird Protection and Study Society of Serbia / BirdLife Serbia |
| Slovakia | Slovak Ornithological Society / BirdLife Slovakia |
| Slovenia | DOPPS - BirdLife Slovenia |
| Spain | Spanish Society of Ornithology / SEO BirdLife |
|  | Catalan Ornithological Institute |
| Sweden | Lund University, Department of Biology |
|  | Swedish University of Agricultural Sciences, The Swedish Species Information Center |
| Switzerland | Swiss Ornithological Institute |
| United Kingdom | British Trust for Ornithology |

**References**

Burnham, K. P., and D. R. Anderson. 2002: “Model selection and multimodel inference: A Practical Information-Theoretic Approach”. Springer.

EBCC, European Bird Census Council. 2024. “European Bird Census Council website.”[https://ebcc.info](https://www.ebcc.info).

Johnston, A., W. H. Hochachka, M. E. Strimas-Mackey, et al. 2021. “Analytical guidelines to increase the value of community science data: an example using eBird data to estimate species distributions.” *Diversity and Distributions* 27, no. 7: 1265–1277.

Moudrý, V., M. Bazzichetto, R. Remelgado, et al. 2024. “Optimising occurrence data in species distribution models: sample size, positional uncertainty, and sampling bias matter.” *Ecography* 2024, no. 12: e07294.

Oliver, R. Y., C. Meyer, A. Ranipeta, K. Winner, and W. Jetz. 2021. “Global and national trends, gaps, and opportunities in documenting and monitoring species distributions.” *PLOS Biology* 19, no. 8: e3001336.

Radišić, D. 2019. “Procena efektivnosti zaštićenih područja i IBA mreže za odabrane vrsta ptica u Srbiji.” PhD diss., University of Novi Sad, Serbia.

Strimas-Mackey, M., W. M. Hochachka, V. Ruiz-Gutierrez, et al. 2023. “Best practices for using eBird data (Version 2.0).” Cornell Lab of Ornithology.<https://doi.org/10.5281/zenodo.3620739>.
